# Transcriptomic profiles of tumor-associated neutrophils reveal prominent roles in enhancing angiogenesis in liver tumorigenesis in zebrafish

**DOI:** 10.1038/s41598-018-36605-8

**Published:** 2019-02-06

**Authors:** Xiaojing Huo, Hankun Li, Zhen Li, Chuan Yan, Ira Agrawal, Sinnakaruppan Mathavan, Jianjun Liu, Zhiyuan Gong

**Affiliations:** 1 Department of Biological Sciences, National University of Singapore, Singapore, Singapore; 2 Genome Institute of Singapore, Singapore, Singapore

## Abstract

We have previously demonstrated the pro-tumoral role of neutrophils using a *kras*-induced zebrafish hepatocarcinogenesis model. To further illustrate the molecular basis of the pro-tumoral role, Tumor-associated neutrophils (TANs) were isolated by fluorescence-activated cell sorting (FACS) and transcriptomic analyses were carried out by RNA-Seq. Differentially expressed gene profiles of TANs from larvae, male and female livers indicate great variations during liver tumorigenesis, but the common responsive canonical pathways included an immune pathway (Acute Phase Response Signaling), a liver metabolism-related pathway (LXR/RXR Activation) and Thrombin Signaling. Consistent with the pro-tumoral role of TANs, gene module analysis identified a consistent down-regulation of Cytotoxicity module, which may allow continued proliferation of malignant cells. Gene Set Enrichment Analysis indicated up-regulation of several genes promoting angiogenesis. Consistent with this, we found decreased density of blood vessels accompanied with decreased oncogenic liver sizes in neutrophil-depleted larvae. Collectively, our study has indicated some molecular mechanisms of the pro-tumoral roles of TANs in hepatocarcinogenesis, including weakened immune clearance against tumor cells and enhanced function in angiogenesis.

## Introduction

Hepatocellular carcinoma (HCC) is the most common type of primary liver cancer with high malignancy and mortality^1^. HCC is frequently caused by chronic inflammation in the liver, where the immune cells create an unresolved, chronic inflammation by initiating and maintaining infiltrating immune cells and producing cytokines in the liver^2,3^. Among these immune cells, neutrophils, the most abundant immune cells in human, have been proved to play a role in a variety of tumors^4,5^. In recent studies, various roles of tumor-associated neutrophils (TANs) have been identified, including the existence of N1 (anti-tumoral) and N2 (pro-tumoral) tumor-associated neutrophils in tumor development and progression^6^.

We have previously developed several inducible HCC models by transgenic expression of selected driver oncogenes^7–11^. In these transgenic models, the driver oncogene can be temporally activated to initiate liver carcinogenesis and histologically proven HCC are usually produced in a few weeks. Thus, these transgenic models provide a powerful tool for investigation of liver tumor initiation. In previous studies, we have found a prominent role of immune response during liver cancer progression^12^. In particular, we found a rapid migration of neutrophils towards oncogenic liver in a *kras*-induced zebrafish HCC model^13^. By manipulation of neutrophil numbers and activities through pharmaceutical treatments and genetic knockdown, we have demonstrated that tumor-associated neutrophils (TANs) promoted hepatocarcinogenesis during the initiation of *kras*-induced oncogenesis. We found that increased TAN activity accelerated the proliferation of oncogenic hepatocytes and deterred their apoptosis, thus contributing to the pathological malignancy. In that study, although the pro-tumor roles of TANs have been demonstrated, these roles remain to be elucidated at the molecular level. The purpose of this study was to use a transcriptomic approach to provide molecular insights into TANs and their roles in hepatocarcinogenesis. Thus, neutrophils were isolated from larvae, adult males and adult females following oncogenic *kras* activation and used for RNA-seq analyses. By comparing transcriptomic profiles of TANs and matched naïve neutrophils (NNs), we observed prominent roles of TANs in loss of cytotoxicity and pro-angiogenesis, both of which apparently favor tumor initiation and progression.

## Results and Discussion

### Validation of neutrophil identity by transcriptomic profiling

TANs were isolated from *kras*+*/lyz*+ fish (see Methods) and NNs isolated from *lyz*+ fish by FACS. The fluorescent microscope images and flow cytometry dot-plots of the isolated cells showed an obvious enrichment of DsRed+ neutrophils in comparison to the cell suspension before sorting (Supplementary Fig. S1). The purity of the sorted neutrophils (DsRed+) is above 90%. The neutrophil samples from larva (L), male (M) and female (F) adult fish were collected with biological duplicates or triplicates, as summarized in Table S1. RNAs were isolated from these neutrophil samples and sequenced to a depth of 23.4–98.9 × 10^6^ reads for each library. In total, 15 RNA samples were sequenced, including three TAN_L and three matched NN_L from larvae, three TAN_M and two NN_M from male adults, and two TAN_F and two NN_F from female adults. All the sequence reads were then mapped to the zebrafish genome reference, danRer7, after removing low-quality reads. By annotating the mapped reads, a total of 8.8–13.5 × 10^3^ transcript entries were identified with at least one mapped read, constituting 60–90% of total known zebrafish transcript entries in the danRer7 zebrafish genome database (14,868 RefSeq transcript entries in total). To minimize the effect from potentially leaky expression, a cut-off of 30 reads was used to retain the robustly expressed transcripts. As a result, 6.2–10.7 × 10^3^ transcript entries were identified for each pool of neutrophils and used for subsequent analyses (Supplementary Table S1), representing 42–72% of the danRer7 database.

**Figure 1:**
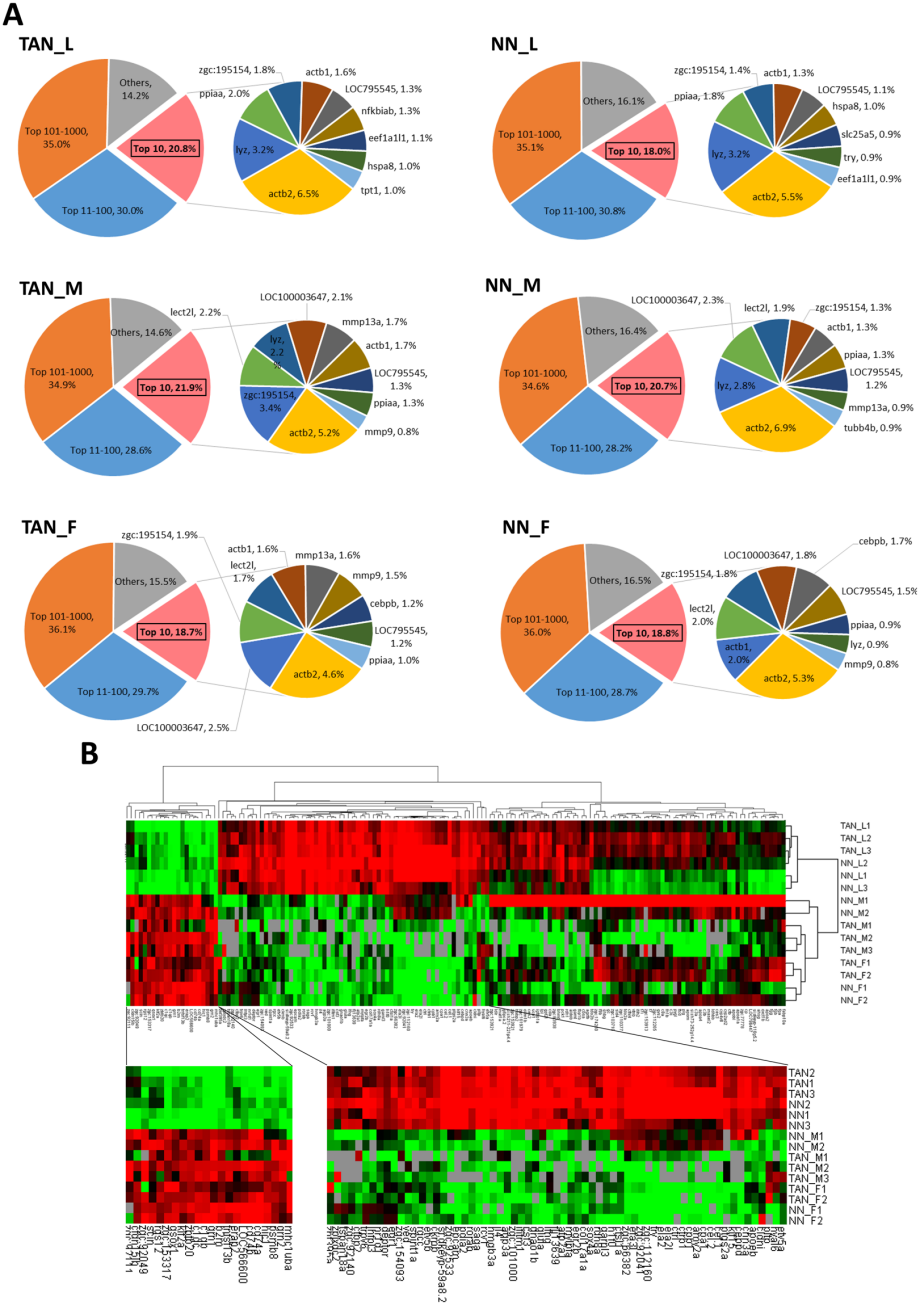
Transcript abundance in neutrophils and hierarchical clustering of neutrophil samples and transcript abundance. (**A**) Transcript abundance distribution in neutrophil populations. The main pie charts of TAN_L, NN_L, TAN_M, NN_M, TAN_F, and NN_F are composed of four slices based on abundance ranking: top 10, 11–100, 101–1,000 and the rest (1,001 and the rest up to 12,000). Gene symbols and relative abundance of the top 10 transcripts are indicated in a subpie in each group. (**B**) Hierarchical clustering of the 15 neutrophil samples. The red and green colors indicate the high and low abundance, respectively.

The distribution of transcript entries and total counts over different transcript abundance categories showed similar profiles for all neutrophil samples (Fig. 1A). There were only a few transcripts which had high abundance while the majority of transcripts were at the very low abundance, which was the typical pattern of transcriptomic profiles of essentially all tissues and cell types^14,15^. The top 10 transcripts accounted for about 20% transcriptome body and the top 100 transcripts constituted around 50% of the transcriptome body. In contrast, the lowest expressed ~11,000 transcript entries (ranked after 1000 and categorized in others) contributed only about 15% of the transcriptome body. In the sub-pie of the top 10 abundant transcripts, the most abundant and common genes in all neutrophil groups were *actb1, actb2, lyz* and *lect2l*. Both *actb1* and *actb2* encodes β-actin, which are commonly known as housekeeping genes and play critical roles in cell mobility. Their crucial roles in neutrophil functions have long been recognized as mutation of these genes in neutrophils could result in abnormalities in chemotaxis, superoxide production and membrane potential response^16^. Both *lyz* and *lect2l* are well-known as neutrophil-specific genes. *lyz* encodes a specific enzyme to hydrolyze specific linkages in bacterial cell wall^17^ while *lect21* is a leukocyte-derived chemotactic factor gene^18^. Both of them play significant roles against pathogen infection in neutrophils. Thus, these top abundantly expressed genes are consistent with the function of neutrophils and validate the neutrophil identities of the cell population we isolated.

To examine the similarities between the RNA-seq samples, we performed hierarchical clustering across all the 15 samples based on gene expression abundance. These samples were clearly clustered into two branches, larva and adult (Fig. 1B). Thus, there was an overwhelming influence of developmental stages on neutrophil transcriptomes. Two distinct gene clusters were enriched in larvae and adults respectively. Among the larva enriched genes, a number of neutrophil developmental regulators were identified, such as *etv5a, hyal6*, *ctrp, ctrl* and *ctbp2*. Among them, c*tbp2* encodes a C-terminal binding protein, which co-activates neutrophil differentiation with a zinc finger transcription factor^19^, indicating an active development of neutrophils in the larval stage (8 dpf), in which the adaptive immune system has not been fully developed^20^. In the adult enriched gene cluster, *mhc1uha, cd74a* and *cd74b*, which encode major histocompatibility complex (MHC) molecules, may indicate the maturity of the adaptive immune system in adult fish. In addition, *psmb8* and *b2m* are important components of MHC I molecules^21,22^ while *grn1, grn2, rgs12, and tnfsfl3b* are associated with neutrophil responses to chemokines and cytokines^23,24^.

### Distinct transcriptomes between tumor associated and naïve neutrophils

**Figure 2:**
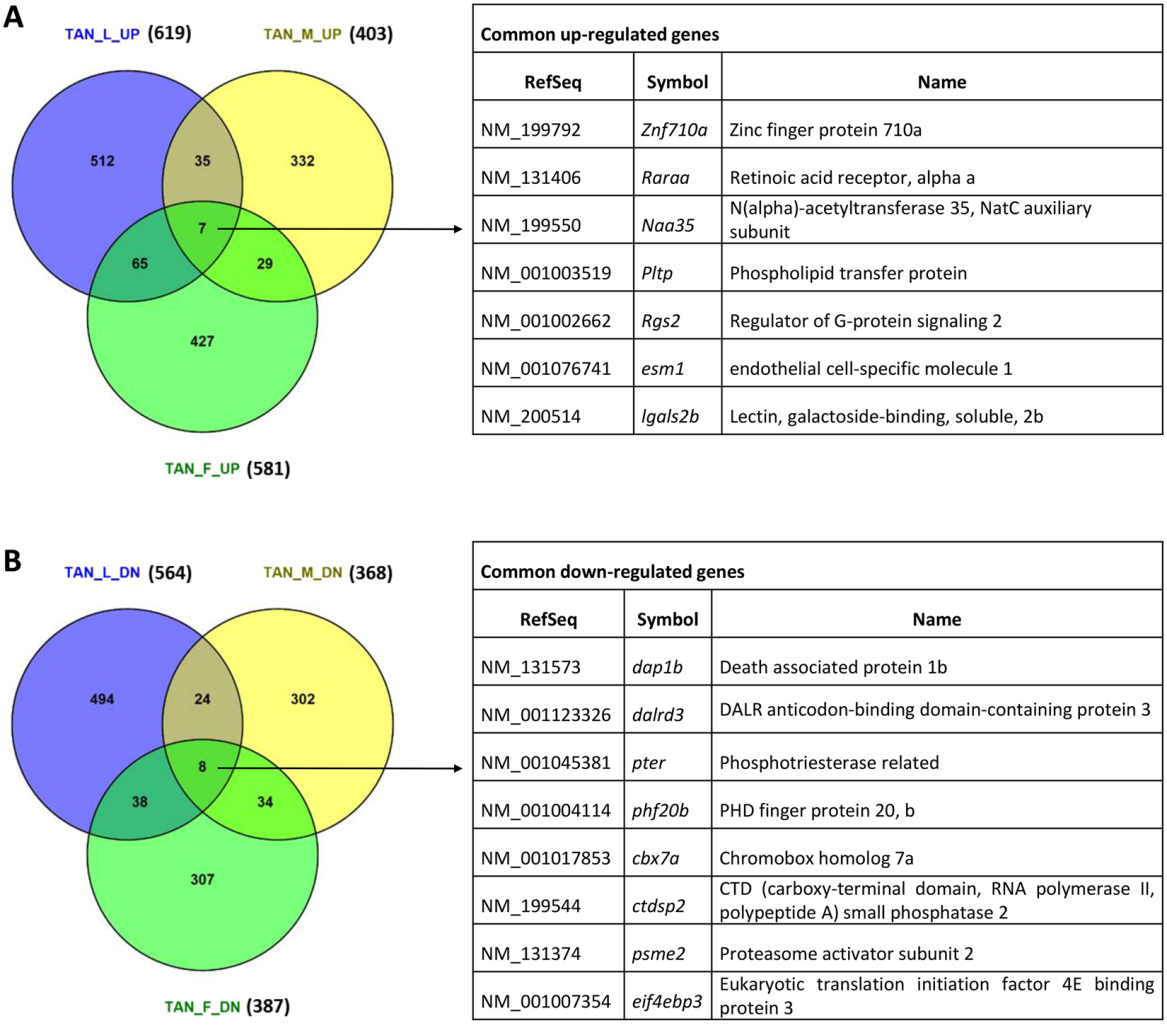
Venn diagrams of DEGs in the three TAN groups: TAN_L, TAN_M and TAN-F. (**A**) Overlaps of up-regulated genes in the three TAN groups. (**B**) Overlaps of down-regulated genes in the three TAN groups. The commonly deregulated genes among all the three TAN groups are listed on the right of each Venn diagram.

In order to extract specific transcriptomic features for TANs, DEGs were identified by comparison of matched TANs and NNs. By using selection criteria of fold change >1.25 and p-value <0.05, DEGs were selected from the three TAN/NN groups. The numbers of up- and down-regulated genes in the larva, male and female TANs are shown in Venn diagrams in Fig. 2 and the list of these genes are shown in Supplementary Tables S2–S4, including 619 up- and 564 down-regulated genes in larva TANs (Supplementary Table S2), 403 up- and 368 down-regulated genes in male TANs (Supplementary Table S3), and 581 up- and 387-down-regulated genes in female TANs (Supplementary Table S4). However, deregulated genes from the three TAN groups had relatively small overlaps with only seven commonly up-regulated genes and eight commonly down-regulated genes (Fig. 2), indicating that both developmental stages and genders affected the response of neutrophils greatly during hepatocarcinogenesis. This is consistent with a previous study on gender difference in neutrophils in responses to cytokine stimulations and malignant growth^25^. Moreover, the aged neutrophils have also been demonstrated to be different from the young ones in human^26^. To verify the dynamic range of gene expression, reverse-transcription quantitative PCR (RT-qPCR) was performed for the fifteen common DEGs. As shown in Supplementary Fig. S2, there was a good correlation between the RNA-seq and RT-qPCR results for up- and down-regulation of these common DEGs (Supplementary Fig. S2).

The seven commonly up-regulated genes are involved in various biological activities, including regulation of G-protein signaling (*rgs3*), lipid transport (*pltp*), transcriptional regulation (*raraa* and *znf710a*) and vascularization (*lgals2b, naa35* and *esm1*). Among these genes, *esm1* has been reported to be associated with cancer and it is involved in cell survival, cell cycle progression, migration, angiogenesis, invasion and epithelial-mesenchymal transition during tumor invasion in colorectal cancer^27^. The eight commonly down-regulated genes also have multiple functional implications, including apoptosis (*dap1b*), phosphatase activity (*ctdsp2*) and chromatin-associated protein (*cbx7a*), ion binding and metal transition (*pter* and *phf20b*), translation (*dalrd3, eif4ebp3*), and antigen presenting and processing (*psme2*). Notably, *psme2* encodes a proteinase in immunoproteasome, which plays a role in the processing of class I MHC peptides^28^. The consistent down-regulation of this gene in all the three TAN groups might indicate their suppressed antigen presenting function during hepatocarcinogenesis.

### Commonly upregulated canonical pathways in TANs as revealed by IPA

**Figure 3:**
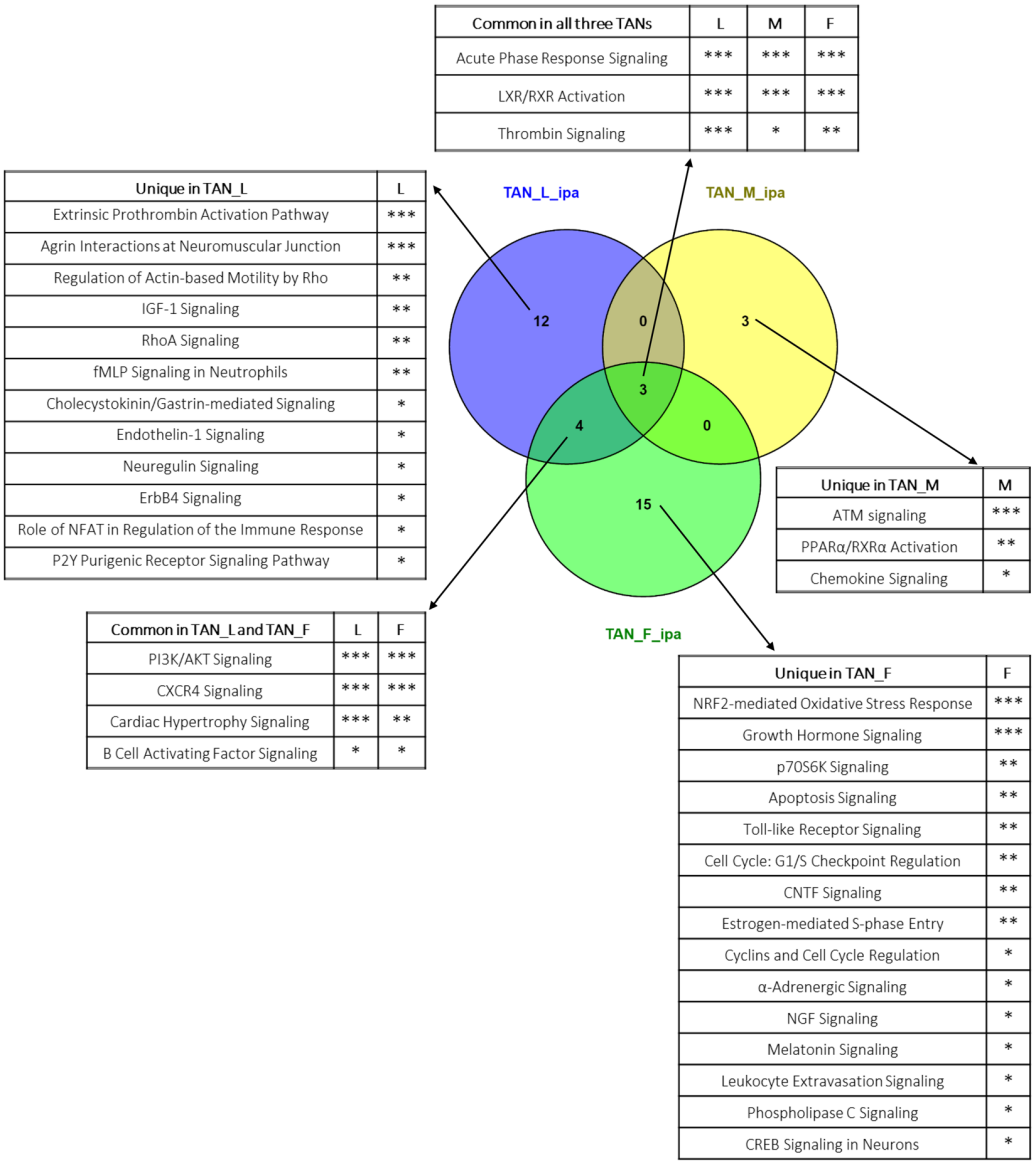
Overlap of IPA canonical pathways in the three TAN groups: TAN_L, TAN_M and TAN-F. The Venn diagram shows the overlap among the TAN groups. The pathways from each component of the Venn diagram are listed in the tables with indication of their statistical significance in canonical pathway analysis (***p < 0.001, **p < 0.01, *p < 0.05).

To further understand biological properties of TANs, we identified canonical pathways enriched in DEGs in TANs via IPA. As shown in Fig. 3, the overlapping pathways among the three TAN groups were few. The common pathways enriched in all TAN groups were Acute Phase Response Signaling, LXR/RXR Activation, and Thrombin Signaling. The first two pathways were highly robust and appeared to be highly significant (p-value < 0.001) at the top of the lists from all three TAN groups. Acute Phase Response Signaling is associated with cytokine production for innate immune cells in response to stimuli, such as malignant growth^29^. LXR/RXR Activation is involved in lipid metabolism and it has been reported to be activated in HCC^30,31^. It is interesting to note that LXR activation has been demonstrated to impair neutrophil motility in an infection model, and the inhibition of chemokine-induced RhoA activation has been identified as a putative underlying mechanism^32,33^. The functional implication of this pathway is consistent with our earlier observation that TANs becomes more stagnant after infiltrating into the tumor microenvironment^13^. In addition, Thrombin Signaling plays an enhancing role in cell adhesion and has long been found to promote tumor in metastasis initiation^34,35^. Thus, these data revealed crucial responses of neutrophil towards the oncogenic transformation of hepatocytes and tend to indicate their pro-tumor roles.

### Impairment of cytotoxicity function in TANs as revealed by immune module analysis

**Figure 4:**
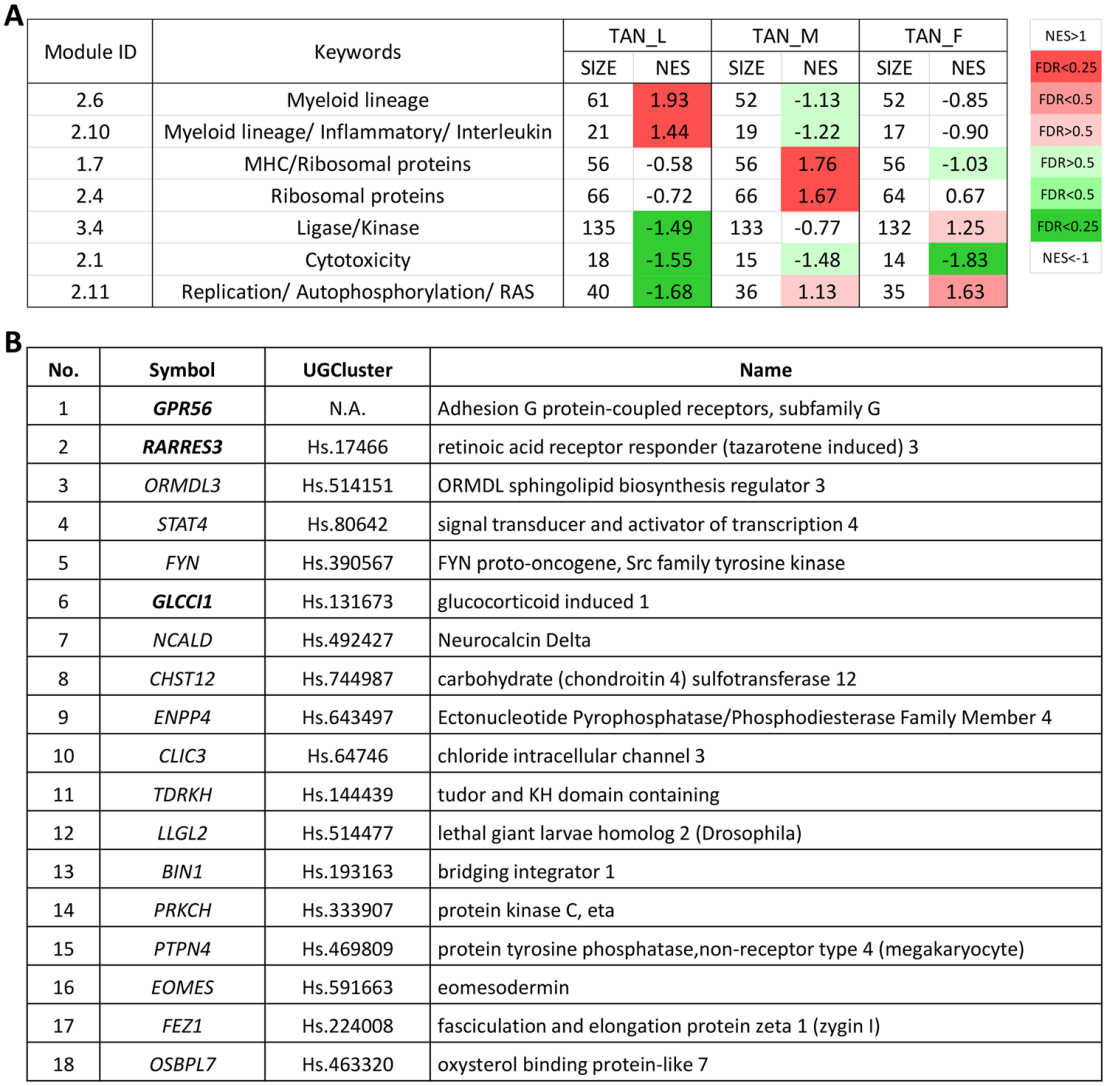
Immune module analysis of TAN transcriptomes by GSEA. (**A**) List of enriched immune modules in TAN groups. Significant normalized enrichment scores (NES) are highlighted based on False Discovery Rates (FDR), which are shown in color as indicated by a color scale on the right. Red indicates positive values of NES and green indicates the negative values. FDR values below 0.25 are regarded as statistical significance. (**B**) List of leading edge genes in Module 2.1 Cytotoxicity. Gene No. 1–14 are common among all the TAN groups. No. 15 is common in TAN_L and TAN_M. No. 16–18 are only found in TAN_L. The bold highlighted genes are discussed in the paragraph. N.A., not available.

To further characterize immune response in the three TAN groups, gene module analysis was conducted. All gene modules significantly up- and down-regulated are shown in Fig. 4A. These gene modules revealed distinct enrichments in the three TAN group; however, Module 2.1 (Cytotoxicity) was consistently down-regulated among the three TAN groups. In this module, TAN_L, TAN_M and TAN_F contained 18, 15 and 14 leading edge genes and 14 of them were common in all the three TANs (Fig. 4B). The top down-regulated genes included tumor suppressors such as *GPR56, RARRES3*, and *GLCCI1. GPR56* encodes a non-classical adhesion receptor and has been identified as a new type of adhesion receptor that binds to extracellular matrix proteins^36^. Mediated by this transmembrane receptor, the cell adhesion has formed a critical inhibitory process during cancer progression; for example, *GPR56* has been reported to suppress tumor growth and metastasis in a melanoma model^37^. *RARRES3* has been identified as a class II tumor suppressor in B cell chronic lymphocytic leukemia and colorectal cancer^38,39^. It is also down-regulated in human HCC tumors and its overexpression in hepatoma cells promotes apoptosis^40^. *GLCCI1* is an early marker of glucocorticoid-induced apoptosis, involving in breast cancer and colorectal cancer^41–44^. Consistent with the roles of *RARRES3* and *GLCCI1* in apoptosis, we also observed previously that TANs inhibited apoptosis of oncogenic hepatocytes in zebrafish and thus promoted hepatocarcinogenesis^13^. Thus, the transcriptomic data further support the pro-tumor role of TANs by suppression of tumor-killing capability through inhibition of apoptosis.

### Resemblance of zebrafish TAN signature to mouse TAN transcriptome

**Figure 5:**
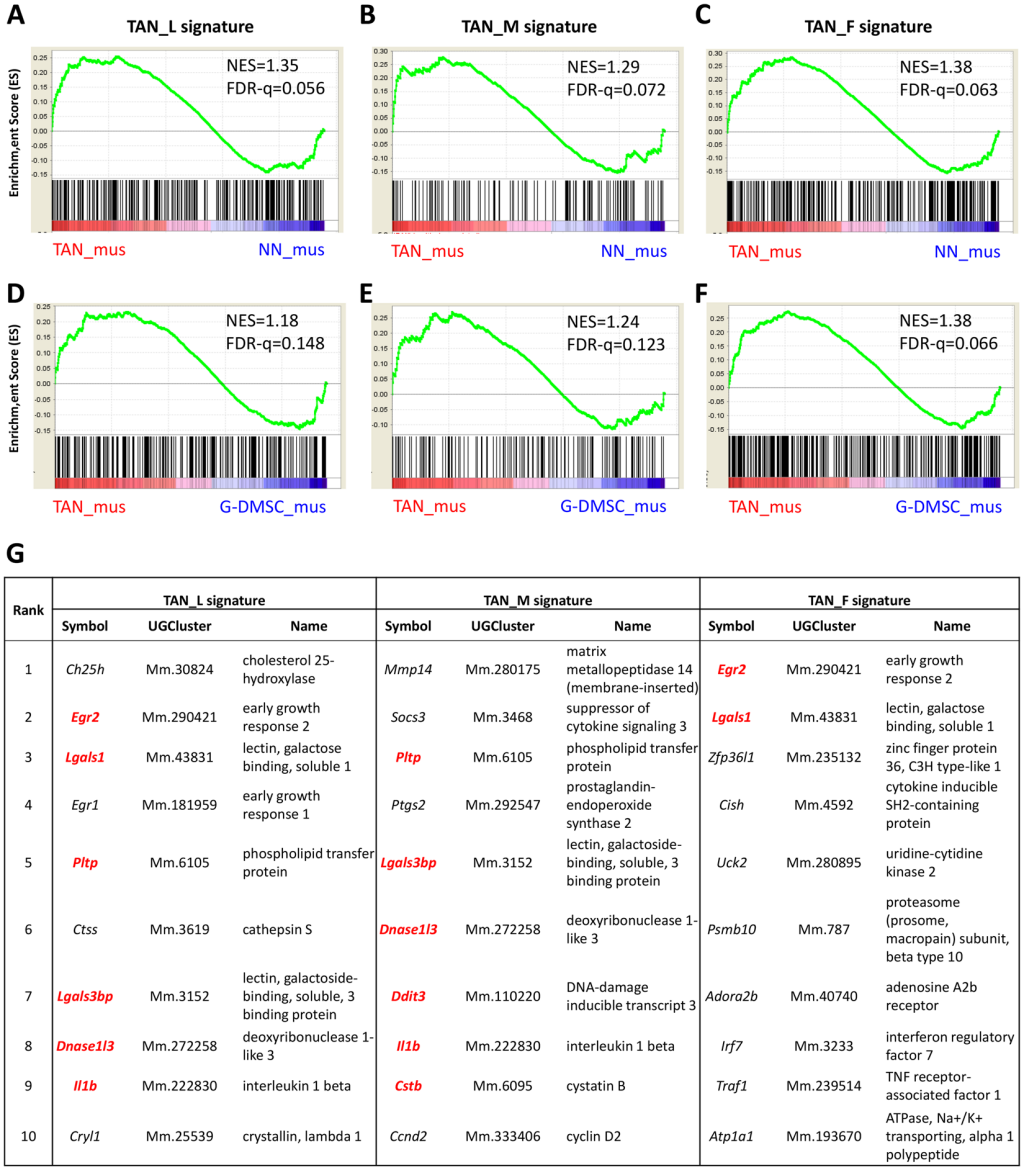
Enrichment of TAN signatures in comparison to mouse TAN dataset. (**A**–**F**) Enrichment plots by GSEA in comparison of zebrafish TANs with mouse TAN (**A**–**C**) or G-MDSC (**D**–**F**). Up-regulated genes from TAN_L (**A**,**D**), TAN_M (**B**,**E**), and TAN_F (**C**,**F**) as signatures were compared to a mouse TAN microarray dataset (GSE43254) by GSEA. The similarity between zebrafish TAN groups and mouse TAN (TAN_mus) was separately compared against mouse NN (NN_mus, **A**–**C**) and against mouse G-MDSC (G-MDSC_mus, **D**–**F**). NES indicate the correlation between two dataset and a positive value refers to positive correlation. FDR measures the statistical significance of NES, and the value below 0.25 is regarded as significance. (**G**) List of the top 10 leading edge genes between zebrafish TAN groups and TAN_mus basedon mouse gene symbols. The leading edge genes which also emerged in other TAN groups are labeled in red.

To further validate the isolated zebrafish TANs and their potential functional conservation across species, zebrafish TAN transcriptomic data were compared by GSEA with the transcriptomic data from mouse TANs, which were isolated from *Kras*-driven melanoma and were so far the best matched TAN data for our *kras*-driven liver tumor in zebrafish^45^. The dataset also contained microarray data of NNs isolated from mouse bone marrow and the granulocyte-like myeloid derived suppressor cells (G-MDSC) isolated from melanoma respectively as non-tumor control and parallel tumor associated granulocytes. The up-regulated gene list of each zebrafish TAN group was used to represent its transcriptomic signature. As shown in Fig. 5A–F, based on normalized enrichment scores (NES) and false discovery rate (FDR), the zebrafish TAN groups show significantly high resemblance to the mouse TANs when they were compared against both NNs and G-MDSC. Thus, there is a significant similarity between zebrafish TAN transcriptomes and mouse TAN transcriptome, which are highly distinguishable from either NN or G-MDSC.

To gain biological insights of the similarity between zebrafish and mouse TANs, the top leading edge genes between them are presented in Fig. 5G and Supplementary Tables S5–S7, and their strong association with tumors were noted. The list contains pro-angiogenic genes (*Lgals1, Lgals3bpb, Il1b, Vegfa*), pro-tumor cytokines (*Il1b, Il10*, and *Il10ra*), and tumor-related transcriptional factors (*Egr1* and *Egr2*), indicating that zebrafish and mouse TANs may have similar pro-tumor characteristics in these aspects. These example genes could be potential biomarkers for diagnosis and therapy. For example, *IL10* and *IL10RA* have been utilized as potential therapeutic targets in a mouse HCC model, as the use of anti-IL10 and anti-IL10RA oligodeoxynucleotide in the treatment has enhanced the anti-tumor activity of macrophages^46^.

### Pro-angiogenic role of TANs

**Figure 6:**
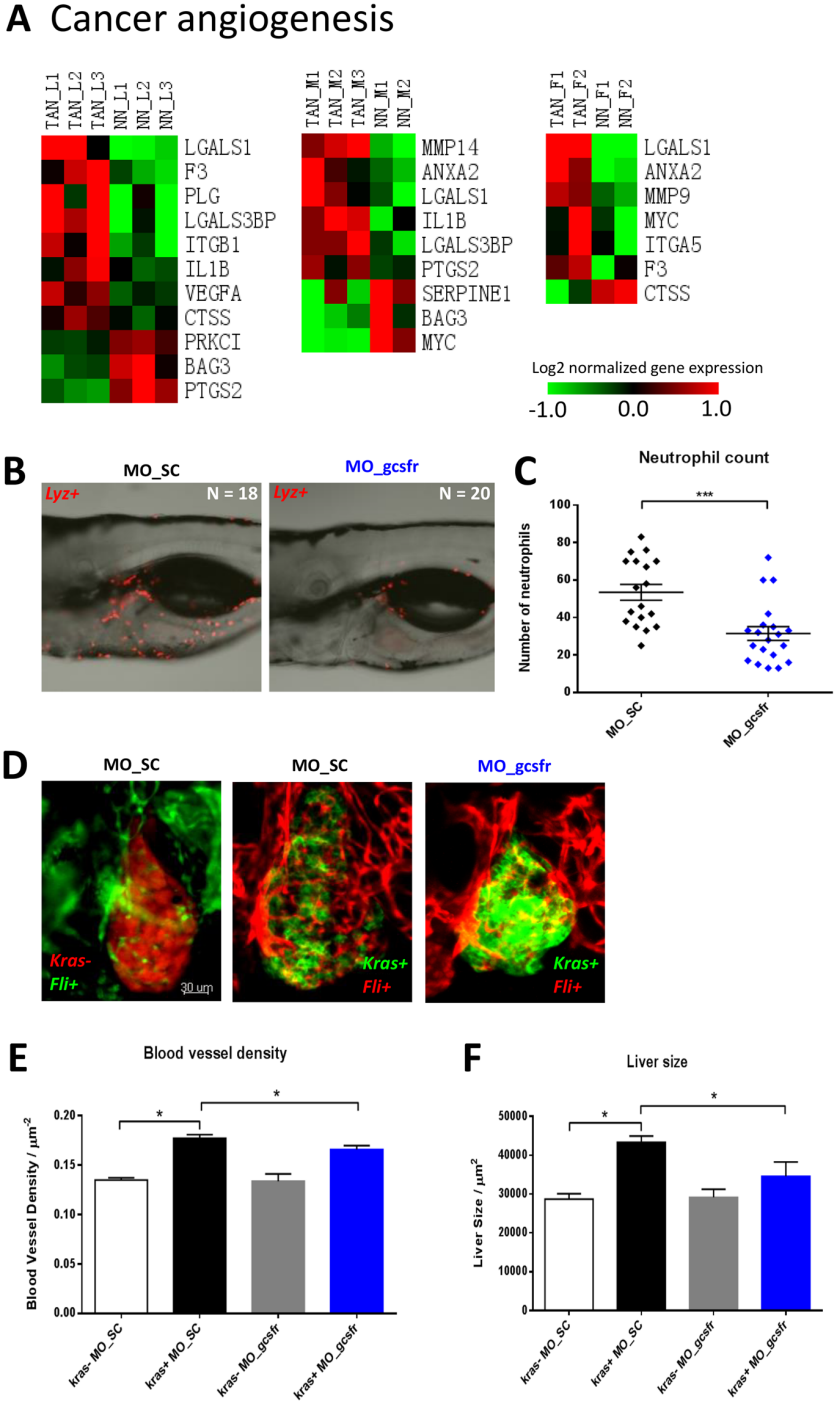
Expression of cancer angiogenesis genes in TANs and effects of neutrophil depletion on liver tumorigenesis and angiogenesis. (**A**) Heatmap representation of expression of cancer angiogenesis genes extracted from DEGs of TANs. The color codes represent fold changes of expression of each gene in each sample relative to the mean expression of all samples. The list of cancer angiogenesis genes was obtained based on IPA knowledge datbase. (**B**) Representative confocal images of 6-dpf *lyz*+ larvae injected with different morpholinos: MO_SC (left, n = 18) or MO_gcsfr (right, n = 20). (**C**) Numbers of neutrophils in the middle body after injection of morpholinos. Neutrophils were counted in the the middle body surrounding the liver region (from the posterior edge of eye to posterior edge of swimbladder). (**D**) Representative 3D confocal images of 6-dpf *kras−/fli*+ and *kras*+*/fli*+ larvae injected with MO_SC or MO_gcsfr. For *fli*+ larvae, both *Tg*(*fli1a::GFP*) (left) and *Tg*(*fli1a::RFP*) (middle and right) were used. *Kras-* larvae were LiPan strain (left) with DsRed expression in the liver. (**C** and **D**) Quantification of blood vessel density in the liver area (**E**) and liver size (**F**) after morpholino injection. N >20 in each group. Statistical significance: *p < 0.05.

There was initial evidence suggesting that TANs can affect tumor angiogenesis^47^. However, the mechanism remains unclear. As shown in Fig. 6A, we found that many pro-angiogenic genes were differentially expressed in all three TAN groups. These pro-angiogenic genes included *vegfa* (vascular endothelial growth factor), *il1b* (pro-angiogenic interleukin), *itgb1* (integrin), *mmp14* and *mmp9* (matrix metallopeptidases) and gene encoding other cell adhesion molecules (*ilgals1* and *igals3bp*). The overwhelming up-regulation of these pro-angiogenesis genes in TANs has indicated a prominent role of neutrophils in promoting tumor angiogenesis during tumor initiation. Notably, *lgals3bpb* was found to be up-regulated in TANs from both mouse melanoma and early zebrafish liver tumorigenesis based on the leading edge genes of cross-species transcriptomic comparison between zebrafish and mice (Fig. 5G). Interestingly, *LGALS3BP* is also up-regulated in human HCC and cirrhosis tissue^48^. Lgals3bp extensively interacts with extracellular matrix components including fibronectin and β1-integrin, both of which are up-regulated in oncogenic hepatocytes based on our unpublished hepatocyte transcriptomic data. This interaction involved in fibrosis and angiogenesis in the development of malignant liver tumors^49^.

To evaluate the effect of neutrophils on tumor angiogenesis, the number of neutrophils was manipulated by suppression of neutrophil differentiation with MO_gcsfr^50^ in *kras*+*/fli*+ double transgenic embryos. The effects of these morpholino oligonucleotides have been previously validated in earlier reports^50^ as well as in our laboratory^13,51^. In this study, as shown in Fig. 6B,C, we found that the number of neutrophils in the liver region in MO_gcsfr injected larva was significantly lower than that in the larvae injected with the control MO_SC morpholino. Next, we injected mopholino oligonucleotides into the *kras*+*/fli*+ and *kras−/fli*+ embryos at one-cell stage and analyzed under a confocal microscope at 6 dpf with Dox induction from 3 dpf. As shown in Fig. 6D, in the MO_SC injected groups, blood vessels were obviously increased in *kras*+*/fli*+ larvae (middle) compared to *kras−/fli*+ larvae after Dox induction (left). The increased blood vessels as well as increased liver size were confirmed by quantification of *fli*+ blood vessel area (Fig. 6E) and the 2D liver size (Fig. 6F), which were consistent with our observation of increased blood vessels in the *Myc* transgenic zebrafish model following the induction of *Myc*-mediated liver tumorigenesis^52^. Thus, MO_gcsfr injection suppressed neutrophil differentiation and resulted in significant decreases in both the density of blood vessels and liver size. These observations made at two different dpf (RNA-seq at 8 dpf and morpholino knockdown at 6 dpf) are consistent for a pro-angiogenic role of neutrophils in the initial stage of hepatocarcinogenesis and should add more confidence on the conclusion, which is also consistent with a prominent pro-angiogenic role of TANs in hepatocarcinogenesis as previously suggested for human HCC^53^.

## Methods

### Zebrafish husbandry and induction of liver tumors

All zebrafish experiments were carried out in accordance with the recommendations in the Guide for the Care and Use of Laboratory Animals of the National Institutes of Health and the protocol was approved by the Institutional Animal Care and Use Committee (IACUC) of the National University of Singapore (Protocol Number: 096/12). Five transgenic lines were used in this study: *Tg*(*fabp10::rtTA2s-M2; TRE2::EGFP-kras*^*G12V*^*)*, shorted as *kras*+, which was generated using a Tet-On system to have liver-specific expression of oncogenic kras^G12V 7^; *Tg*(*lyz::DsRed)*, shorted as *lyz*+. in which neutrophils are labeled with DsRed under the control of neutrophil-specific *lyz* (lysozyme C) promotor^54^; *Tg*(*fli1a::GFP)*^55^ and *Tg*(*fli1a::RFP*)^56^ with GFP- and RFP-labeled blood vessels respectively, both of which are shorted as *fli*+ in this report; LiPan transgenic zebrafish, *Tg*(*fabp10::DsRed; ela3l::EGFP*) with DsRed expression in the liver and EGFP expression in exocrine pancreas^57^. To induce the expression of Kras^G12V^-EGFP in *kras*+ fish, 20 µg/ml doxycycline (Dox; Sigma, D9891) was used for larvae from 3 day post fertilization (dpf) to 8 dpf and for adult fish from 6 month post fertilization for 5 days.

### Isolation of neutrophils

Neutrophils were isolated from both 8-dpf larvae (n > 50 each sample) after removal of head and tail parts to enrich the liver portion) and adult livers (4–7 fish pooled for each sample) through fluorescence-activated cell sorting (FACS) using a cell sorter (BD Aria) following a previously described protocol^58^. TANs were isolated based on DsRed expression cells from Dox-treated kras+/lyz+ double transgenic fish and NNs were isolated based on DsRed expression from *lyz*+ larvae. The purity of FACS isolated neutrophils was above 90%, and the number of cells collected from each sample was above 10,000.

### Morpholino knockdown and confocal imaging

Morpholino knockdown was performed on *kras*+*/fli*+ or *kras−/fli*+ as previously described^13^. Two morpholino oligonucleotides were designed and synthesized by GeneTools (Philomath, OR). A previously validated morpholino oligonucleotides for reduction (MO_gcsfr, 5′-GAAGCACAAGCGAGACGGATGCCAT-3′)^50^ of neutrophil population, as well as a standard control morpholino (MO_SC, 5′-CCTCTTACCTCAGTTACAATTTATA-3′) that targeted a human beta-globin intron, were used in this study. These morpholinos were injected into zebrafish embryos at one-cell stage and the larvae developed from injected embryos were imaged at 6 dpf using a confocal microscope (Carl Zeiss LSM510) for examination of their angiogenesis in livers. Measurement of neutrophil number, liver sizes and blood vessels was performed using ImageJ as previously described^9,59^ and >20 larva for each group were analyzed in this study. The difference between comparing groups was evaluated by student’s t-test using Graphpad Prism 6 (statistical significance: *p < 0.05, **p < 0.01, ***p < 0.001).

### RNA extraction and library preparation

Following FACS sorting, the viability and purity of isolated neutrophils was tested immediately once the cell sorting was finished. Only the sorted samples with over 98% DAPI negative (living cells) and over 90% DsRed positive (neutrophil marker) were processed for the RNA extraction using the RNeasy Micro Kit (Qiagen, 74004). The quality and quantity of isolated RNA were examined with Bioanalyzer by using Agilent RNA 6000 Pico Kit (Agilent Technologies, 5067-1513) and all RNA samples in this study had RNA integrity values above 7. These RNAs were processed to NGS (next generation sequencing)-qualified cDNA by using SMARTer Ultra Low Input RNA Kit for Sequencing (Clontech Laboratories, Inc., 634848). The cDNAs were examined with Bioanalyzer by using Agilent High Sensitivity DNA Kit (Agilent Technologies, 5067-4626). DNA shearing was performed on Covaris AFA system and the resulting DNA was in the 200–500 bp range. The sheared cDNA was prepared for constructing multiplex sequencing libraries using NEB DNA Library Prep Kits (New England Biolabs Inc., E7370L and E7335S). Each multiplex occupied one lane and was sequenced on Illumina HiSeq NGS platform with the sequencing read length of 100 bp.

### RNA-seq data processing and annotation

Sequence reads were assembled by aligning to the zebrafish reference genome sequence (Danio rerio, UCSC version danRer7, 2010) using TopHat2.0 (http://ccb.jhu.edu/software/tophat/index.shtml). All the zebrafish genes were annotated to human and mouse orthologous/homologous genes by retrieving from the Genome Institute of Singapore Zebrafish Annotation Database (http://giscompute.gis.a-star.edu.sg/~govind/unigene_db/) as previously described^60^. The quantification of reads mapped to each gene was performed using a python package, HT-Seq^61^ with ambiguous and non-unique alignments depleted.

### RNA-seq data analyses

Based on sequence count of each gene, which has been normalized against the total sequence counts, hierarchical clustering was performed using Cluster 3.0 (http://bonsai.hgc.jp/~mdehoon/software/cluster/software.htm) across all neutrophil samples to examine their similarities. Differentially expressed genes (DEGs) between TANs and NNs were identified by using DESeq2, an R package, which normalizes the count data and models with negative binomial distribution^62^, with cut-off p-value at 0.05 and fold change at 1.25. The biological insights of the DEGs were mined by using IPA (Ingenuity Pathway Analysis, Qiagen). The fold change and p-value of DEGs were input for the establishment of causal networks and the overlap p-value for each pathway was calculated using one-sided Fisher’s exact test. The overlap p-value < 0.05 was considered to be significant. Gene module analysis was conducted based on coordinated gene expression pattern from multiple disease conditions according to Chaussabel *et al*.^63^. The comparison between zebrafish TAN signatures (defined by significantly up-regulated genes against NNs) and mouse TAN microarray dataset (GSE43254) obtained from GEO (Gene Expression Omnibus) was performed by Gene Set Enrichment Analysis (GSEA)^64^. The enrichment score of the signatures was estimated by using an empirical phenotype-based permutation test and statistical significance of enrichment score was estimated by FDR < 0.25.

## Electronic supplementary material

Supplementary Figures and Tables
