# Supplementary material for: Transcriptomic profiles of tumor-associated neutrophils reveal prominent roles in enhancing angiogenesis in liver tumorigenesis in zebrafish
Source: Sci Rep. 2019 Feb 6;9:1509. doi: 10.1038/s41598-018-36605-8 (PMC6365535; doi:10.1038/s41598-018-36605-8)
Supplement: Supplementary file 1 — Supplementary Figures and Tables [file 41598_2018_36605_MOESM1_ESM.docx]

Supplementary Information

**Transcriptomic profiles of tumor-associated neutrophils reveal prominent roles in enhancing carcinogenesis and angiogenesis in zebrafish**

Xiaojing Huo^1^, Hankun Li^1^, Zhen Li^2^, Chuan Yan^1^, Ira Agrawal^1^, Sinnakaruppan Mathavan^2^, Jianjun Liu^2^ and Zhiyuan Gong^1*^

^1^Department of Biological Sciences, National University of Singapore, Singapore

^2^Genome Institute of Singapore, Singapore

*Corresponding author: Zhiyuan Gong, Department of Biological Sciences, National University of Singapore, Singapore, 117543. E-mail: [dbsgzy@nus.edu.sg](mailto:dbsgzy@nus.edu.sg); phone: (65)-65162860; fax: (65)-67792486.


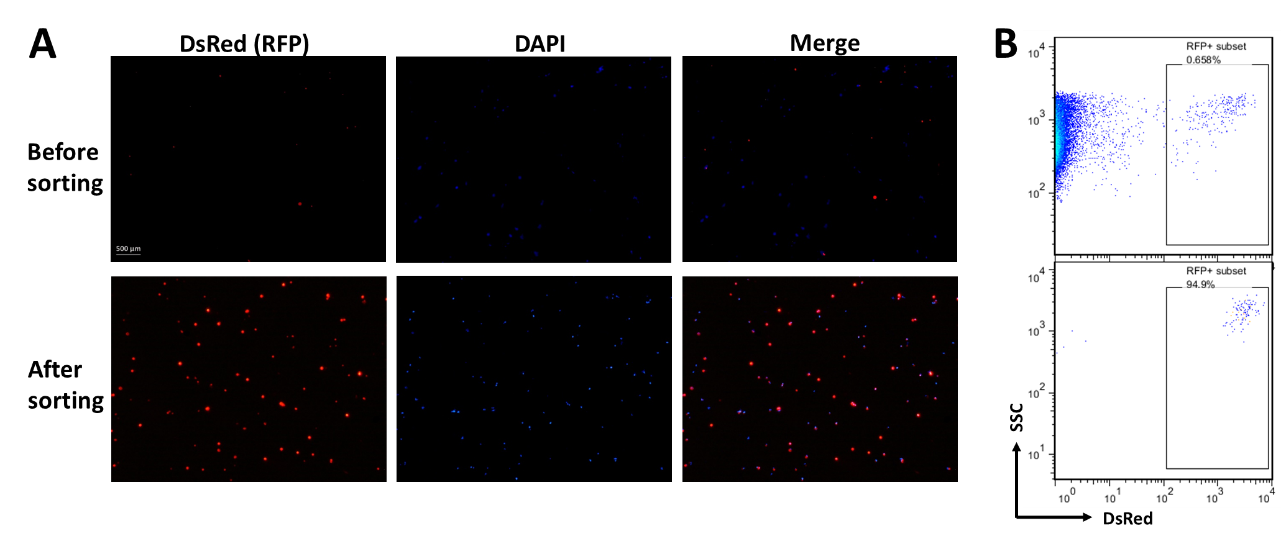


**Supplementary Figure S1. Neutrophils isolated by FACS.** (A) Examples of DsRed+ neutrophils isolated from *kras+/lyz+* larvea by FACS. (B) Flow cytometry dot-plots of dissociated cells of *lyz+* larvae in the measure of side scatter (SSC) and DsRed+ cells before sorting (up) and after sorting (down).


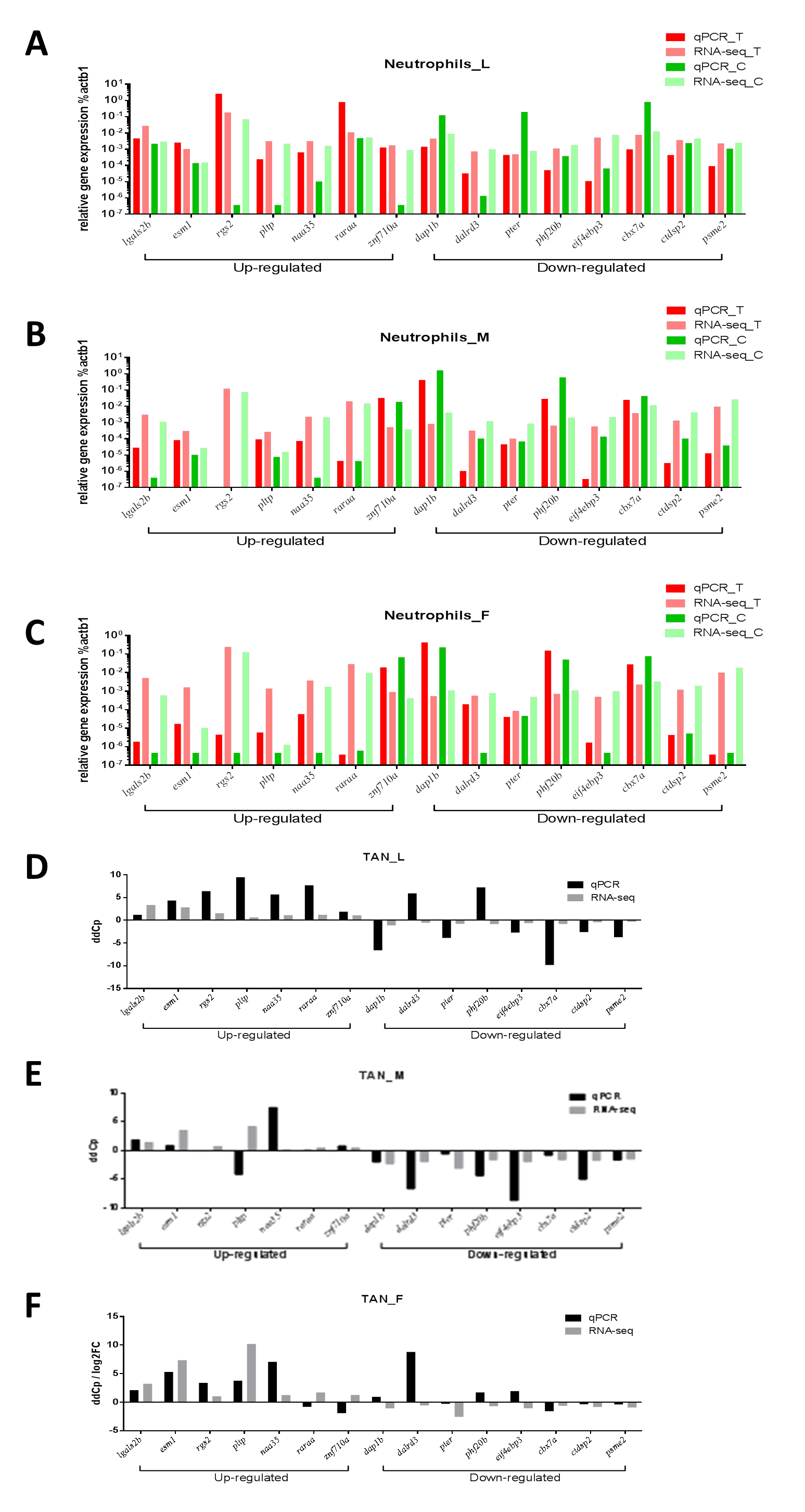
**Supplementary Figure S2. RT-qPCR validation of RNA-seq results using different batch of isolated neutrophil samples.** (A-C) The RT-qPCR and RNA-seq data are presented in relative gene expression value ($2^{- normalized Cp}$) from the qPCR results, and relative RPKM values from RNA-seq. Red bars are tumor samples and green bars are control samples. Both Cp and normalized RPKM values are normalized against the ΔCp and RPKM of housekeeping gene *actb1*. (D-E) The RT-qPCR and RNA-seq data are presented in ΔΔCp and log2 fold change of tumor samples against control. Both ΔCp and normalized RPKM values are normalized against the Cp and RPKM of housekeeping gene *actb1*.

**Supplementary tables**

**Table S1. Summary of RNA-seq data**

| **Sample Name** | **Total Reads Sequenced** | **Total Mapped Reads** | **Uniquely Mapped Reads** | **Total Transcript Entries** | **Transcript entries of read count >30** |
| --- | --- | --- | --- | --- | --- |
| TAN1 | 27,355,811 | 19,682,648 | 12,851,664 | 13,413 | 8,321 |
| TAN2 | 25,295,794 | 15,616,163 | 8,018,616 | 11,941 | 7,474 |
| TAN3 | 58,867,060 | 39,150,274 | 26,362,124 | 12,377 | 8,688 |
| NN1 | 33,422,323 | 25,984,799 | 17,033,500 | 10,797 | 8,125 |
| NN2 | 42,458,748 | 28,822,946 | 17,811,214 | 13,472 | 10,716 |
| NN3 | 61,135,086 | 35,219,223 | 23,975,276 | 9,288 | 7,912 |
| TAN_M1 | 32,480,636 | 17,290,308 | 9,915,618 | 8,789 | 6,194 |
| TAN_M2 | 61,412,539 | 39,115,847 | 24,326,829 | 9,157 | 7,481 |
| TAN_M3 | 23,719,133 | 16,932,403 | 10,888,289 | 8,968 | 6,826 |
| NN_M1 | 98,935,776 | 67,736,931 | 43,028,097 | 12,255 | 9,075 |
| NN_M2 | 23,413,799 | 16,863,794 | 10,584,762 | 9,777 | 6,652 |
| TAN_F1 | 39,690,577 | 24,983,549 | 16,018,345 | 10,526 | 7,146 |
| TAN_F2 | 26,318,814 | 17,472,937 | 11,181,900 | 10,242 | 6,894 |
| NN_F1 | 47,190,404 | 31,025,638 | 18,240,611 | 10,204 | 7,601 |
| NN_F2 | 29,251,976 | 18,904,987 | 11,341,344 | 10,626 | 7,087 |

**Table S2. DEGs in larva TANs**

| Refseq | Unigene ID | Gene Symbol | Hs Gene Symbol | log2FC | pValue |
| --- | --- | --- | --- | --- | --- |
| NM_001015057 | Dr.75658 | *tfa* | *TF* | 1.929 | 5.99E-15 |
| NM_199785 | Dr.26626 | *ercc4* | *ERCC4* | 1.695 | 9.35E-12 |
| NM_001013259 | Dr.150316 | *serpina1l* | *SERPINA1* | 1.474 | 7.21E-08 |
| NM_001080587 | Dr.82221 | *dytn* | *DYTN* | 1.460 | 2.04E-07 |
| NM_200514 | Dr.84923 | *lgals2b* | *LGALS1* | 1.444 | 3.81E-08 |
| NM_001130586 | Dr.104720 | *apoa2* | *APOA2* | 1.424 | 2.30E-07 |
| NM_001006079 | Dr.13499 | *osr1* | *OSR1* | 1.349 | 1.22E-06 |
| NM_200154 | Dr.82434 | *sepsecs* | *SEPSECS* | 1.285 | 3.05E-08 |
| NM_001198745 | Dr.87529 | *LOC100002960* | *N.A.* | 1.280 | 5.00E-06 |
| NM_001077758 | Dr.75688 | *serpina1* | *SERPINA1* | 1.253 | 2.49E-06 |
| NM_212774 | Dr.8505 | *fgb* | *FGB* | 1.228 | 1.19E-05 |
| NM_131242 | Dr.77174 | *c3a* | *C3* | 1.202 | 1.66E-05 |
| NM_001006045 | Dr.40045 | *cited2* | *CITED2* | 1.190 | 1.23E-05 |
| NM_213054 | Dr.77175 | *fgg* | *FGG* | 1.190 | 2.36E-05 |
| NM_213390 | Dr.26855 | *f2* | *F2* | 1.179 | 4.95E-06 |
| NM_201050 | Dr.10580 | *nek2* | *NEK2* | 1.164 | 1.30E-06 |
| NM_213025 | Dr.75972 | *ndufa4* | *NDUFA4* | 1.163 | 1.68E-07 |
| NM_001080010 | Dr.85614 | *serpinf2b* | *SERPINF2* | 1.159 | 3.38E-05 |
| NM_001007151 | Dr.32788 | *shbg* | *SHBG* | 1.150 | 4.35E-05 |
| NM_001114915 | Dr.79754 | *crp3* | *APCS* | 1.128 | 5.97E-05 |
| NM_001194989 | Dr.33271 | *fga* | *FGA* | 1.124 | 5.04E-05 |
| NM_001002568 | Dr.2050 | *gc* | *GC* | 1.116 | 7.30E-05 |
| NM_001099248 | Dr.84654 | *ankdd1a* | *ANKDD1A* | 1.099 | 9.24E-05 |
| NM_198063 | Dr.3585 | *agt* | *AGT* | 1.097 | 8.32E-05 |
| NM_001030244 | Dr.76679 | *vsg1* | *VSG1* | 1.097 | 7.53E-05 |
| NM_200327 | Dr.24982 | *zgc:56585* | *N.A.* | 1.082 | 1.19E-04 |
| NM_001044936 | Dr.132299 | *ces3* | *CES2* | 1.065 | 1.52E-04 |
| NM_001030232 | Dr.75730 | *zgc:114123* | *N.A.* | 1.062 | 1.50E-04 |
| NM_001020588 | Dr.48047 | *zgc:110377* | *ITIH3* | 1.062 | 1.11E-04 |
| NM_001139461 | Dr.13466 | *vtnb* | *VTN* | 1.061 | 1.45E-04 |
| NM_001013261 | Dr.24233 | *fn1b* | *FN1* | 1.060 | 1.65E-04 |
| NM_001144786 | Dr.79872 | *si:ch1073-126c3.2* | *N.A.* | 1.059 | 1.49E-04 |
| NM_001002133 | Dr.150441 | *trim63* | *TRIM63* | 1.057 | 1.09E-04 |
| NM_131243 | Dr.21006 | *c3b* | *C3* | 1.056 | 1.71E-04 |
| NM_001076741 | Dr.87157 | *zgc:153027* | *ESM1* | 1.050 | 1.75E-04 |
| NM_001100029 | Dr.105072 | *si:ch73-252g14.4* | *N.A.* | 1.049 | 1.57E-04 |
| NM_001077612 | Dr.118084 | *phkg1a* | *PHKG1A* | 1.046 | 4.51E-08 |
| NM_001040310 | Dr.83709 | *f9b* | *F9* | 1.034 | 2.35E-04 |
| NM_001089577 | Dr.115641 | *rhcga* | *RHCG* | 1.031 | 1.44E-04 |
| NM_001024435 | Dr.51148 | *c9* | *C9* | 1.025 | 2.42E-04 |
| NM_001083542 | Dr.2132 | *hao1* | *HAO1* | 1.022 | 2.79E-04 |
| NM_001005981 | Dr.80177 | *kng1* | *KNG1* | 1.010 | 3.26E-04 |
| NM_001144804 | Dr.75830 | *wnt11* | *WNT11* | 1.007 | 1.57E-04 |
| NM_201118 | Dr.76990 | *ambpl* | *AMBP* | 1.000 | 3.33E-04 |
| NM_001115054 | Dr.154459 | *si:dkey-25o1.6* | *N.A.* | 0.999 | 3.83E-04 |
| NM_001017728 | Dr.90156 | *f3b* | *F3* | 0.998 | 1.27E-05 |
| NM_200589 | Dr.24063 | *abcc2* | *ABCC2* | 0.998 | 3.72E-04 |
| NM_131338 | Dr.75096 | *cfb* | *CFB* | 0.993 | 3.11E-04 |
| NM_001089517 | Dr.85158 | *zgc:162611* | *N.A.* | 0.988 | 3.09E-04 |
| NM_001040297 | Dr.78286 | *crp4* | *APCS* | 0.986 | 4.56E-04 |
| NM_001123063 | Dr.41082 | *dpys* | *DPYSL3* | 0.986 | 3.85E-04 |
| NM_001076710 | Dr.81338 | *zgc:152891* | *ALOX15B* | 0.986 | 2.54E-04 |
| NM_001199366 | Dr.104840 | *cfhl4* | *CFHL4* | 0.977 | 3.44E-04 |
| NM_001114472 | Dr.111456 | *zgc:171422* | *N.A.* | 0.973 | 3.17E-04 |
| NM_001044900 | Dr.86250 | *ighmbp2l* | *IGHMBP2* | 0.973 | 2.20E-04 |
| NM_001037673 | Dr.78705 | *prg4b* | *PRG4B* | 0.969 | 5.30E-04 |
| NM_152960 | Dr.104721 | *fabp10a* | *FABP10A* | 0.966 | 5.59E-04 |
| NM_001002307 | Dr.89689 | *rbp2b* | *RBP2B* | 0.961 | 6.26E-04 |
| NM_001004546 | Dr.27305 | *zgc:92111* | *N.A.* | 0.949 | 6.33E-04 |
| NM_001001949 | Dr.67637 | *grna* | *GRN* | 0.942 | 2.57E-05 |
| NM_001003448 | Dr.87085 | *zgc:92113* | *N.A.* | 0.936 | 7.85E-04 |
| NM_001025533 | Dr.11422 | *mcm6l* | *MCM6* | 0.936 | 5.51E-04 |
| NM_001100089 | Dr.85308 | *setd8b* | *C12orf65* | 0.932 | 1.96E-04 |
| NM_001105117 | Dr.85476 | *si:ch211-137i24.10* | *N.A.* | 0.930 | 9.35E-04 |
| NM_001083580 | Dr.114598 | *zgc:162885* | *XRCC6BP1* | 0.927 | 1.81E-04 |
| NM_001100020 | Dr.78847 | *si:ch211-140f21.1* | *N.A.* | 0.921 | 7.92E-04 |
| NM_131563 | Dr.8472 | *tnnc2* | *TNNC2* | 0.920 | 6.74E-04 |
| NM_001199728 | Dr.21117 | *apom* | *APOM* | 0.915 | 9.62E-04 |
| NM_182940 | Dr.105480 | *hbae1* | *HBZ* | 0.913 | 5.46E-04 |
| NM_183341 | Dr.86414 | *egr2a* | *EGR2* | 0.912 | 2.43E-05 |
| NM_001195614 | Dr.55459 | *stab1l* | *STAB1* | 0.906 | 1.01E-03 |
| NM_001034182 | Dr.77654 | *sid4* | *HSPG2* | 0.904 | 9.51E-04 |
| NM_001003496 | Dr.77437 | *c8a* | *C8* | 0.903 | 8.82E-04 |
| NM_001003999 | Dr.29999 | *tmem144a* | *TMEM144* | 0.899 | 1.23E-03 |
| NM_001004634 | Dr.79923 | *gpx1b* | *GPX1* | 0.897 | 1.30E-03 |
| NM_001077454 | Dr.74514 | *zgc:153629* | *KRT15* | 0.895 | 1.45E-03 |
| NM_001002662 | Dr.39062 | *rgs2* | *RGS2* | 0.890 | 1.15E-15 |
| NM_001076602 | Dr.83274 | *slc25a38a* | *SLC25A38* | 0.886 | 1.09E-03 |
| NM_199738 | Dr.79928 | *scrn3* | *SCRN3* | 0.883 | 2.38E-04 |
| NM_001126436 | Dr.17352 | *pigx* | *PIGX* | 0.882 | 7.90E-05 |
| NM_173275 | Dr.76526 | *lamb1a* | *LAMB1* | 0.878 | 1.79E-03 |
| NM_001079996 | Dr.80050 | *cyp4v7* | *KLKB1* | 0.877 | 1.60E-03 |
| NM_001025478 | Dr.82288 | *zgc:114121* | *CCDC77* | 0.876 | 1.73E-03 |
| NM_001037236 | Dr.88584 | *c3c* | *C3* | 0.875 | 1.79E-03 |
| NM_001044321 | Dr.87374 | *pglyrp5* | *PGLYRP1* | 0.873 | 1.37E-06 |
| NM_199856 | Dr.80748 | *tdo2b* | *TDO2* | 0.871 | 1.35E-03 |
| NM_001098183 | Dr.141500 | *LOC565548* | *ANKRD34B* | 0.871 | 1.02E-03 |
| NM_001002363 | Dr.87300 | *cd36* | *CD36* | 0.870 | 1.57E-03 |
| NM_001017814 | Dr.87820 | *spata6* | *SPATA6* | 0.869 | 1.61E-03 |
| NM_001114704 | Dr.114900 | *zgc:174260* | *SERPINA9* | 0.864 | 1.84E-03 |
| NM_001003428 | Dr.87252 | *crygn2* | *CRYGN* | 0.862 | 1.65E-03 |
| NM_201065 | Dr.79220 | *surf6* | *SURF6* | 0.862 | 9.06E-04 |
| NM_199798 | Dr.80392 | *rrad* | *RRAD* | 0.862 | 1.68E-03 |
| NM_131672 | Dr.133025 | *csf1ra* | *CSF1RA* | 0.853 | 2.34E-03 |
| NM_001007357 | Dr.79443 | *lrrc20* | *LRRC20* | 0.852 | 2.38E-03 |
| NM_200617 | Dr.6341 | *rars2* | *RARS2* | 0.851 | 2.09E-03 |
| NM_183066 | Dr.1450 | *hbae3* | *HBAE3* | 0.851 | 1.34E-03 |
| NM_173228 | Dr.77123 | *f7i* | *F7* | 0.849 | 1.76E-03 |
| NM_001076730 | Dr.36660 | *zgc:153610* | *EXOC6B* | 0.847 | 2.35E-03 |
| NM_001089448 | Dr.16479 | *zgc:162946* | *N.A.* | 0.840 | 2.47E-03 |
| NM_001002446 | Dr.82532 | *cldn15lb* | *CLDN15LB* | 0.836 | 1.70E-03 |
| NM_001104942 | Dr.132687 | *zgc:171223* | *CCDC99* | 0.831 | 1.46E-03 |
| NM_200113 | Dr.78669 | *agps* | *AGPS* | 0.828 | 4.43E-06 |
| NM_130997 | Dr.75080 | *egr2b* | *EGR2* | 0.827 | 1.35E-03 |
| NM_200144 | Dr.83717 | *upp2* | *UPP2* | 0.825 | 2.75E-03 |
| NM_001170835 | Dr.42191 | *pex1* | *PEX1* | 0.821 | 3.46E-03 |
| NM_001009901 | Dr.132245 | *tnni2a.4* | *TNNI2* | 0.816 | 1.98E-03 |
| NM_001110296 | Dr.85128 | *brip1* | *BRIP1* | 0.810 | 3.63E-03 |
| NM_200156 | Dr.14749 | *nup155* | *NUP155* | 0.806 | 4.26E-04 |
| NM_001109705 | Dr.108155 | *zgc:173593* | *FTMT* | 0.802 | 1.71E-03 |
| NM_001144818 | Dr.158051 | *ggcta* | *GGCTA* | 0.802 | 1.05E-03 |
| NM_131005 | Dr.268 | *epd* | *EPD* | 0.800 | 3.78E-03 |
| NM_199631 | Dr.105341 | *racgap1* | *RACGAP1P* | 0.797 | 1.95E-04 |
| NM_001001407 | Dr.32964 | *mre11a* | *MRE11A* | 0.797 | 3.86E-03 |
| NM_212907 | Dr.25810 | *foxq1b* | *FOXQ1* | 0.797 | 4.23E-03 |
| NM_001004016 | Dr.133666 | *st3gal5l* | *ST3GAL5L* | 0.796 | 4.66E-03 |
| NM_001017610 | Dr.85122 | *ascc1* | *ASCC1* | 0.795 | 9.62E-09 |
| NM_131760 | Dr.1047 | *tyms* | *TYMS* | 0.794 | 6.10E-05 |
| NM_001122761 | Dr.110984 | *slc6a13l* | *SLC6A13L* | 0.794 | 2.52E-03 |
| NM_131516 | Dr.460 | *pvalb2* | *PVALB2* | 0.790 | 4.21E-03 |
| NM_001111148 | Dr.81676 | *zgc:171416* | *TONSL* | 0.790 | 4.52E-03 |
| NM_001044340 | Dr.94434 | *mov10b.2* | *MOV10* | 0.786 | 5.13E-03 |
| NM_200786 | Dr.83748 | *xrcc4* | *XRCC4* | 0.784 | 4.11E-03 |
| NM_201193 | Dr.26666 | *tln2* | *TLN2* | 0.781 | 3.18E-03 |
| NM_205643 | Dr.80068 | *pnp4b* | *PNP* | 0.779 | 5.44E-03 |
| NM_001008652 | Dr.82220 | *ch25h* | *CH25H* | 0.779 | 8.69E-04 |
| NM_001171066 | Dr.151469 | *si:ch211-283h6.6* | *N.A.* | 0.778 | 5.09E-03 |
| NM_001040332 | Dr.14207 | *zyg11* | *ZYG11B* | 0.778 | 2.53E-09 |
| NM_001017707 | Dr.33948 | *arf4a* | *ARF5* | 0.778 | 4.34E-03 |
| NM_198338 | Dr.114267 | *slc4a1a* | *SLC4A1A* | 0.776 | 2.90E-03 |
| NM_001002615 | Dr.161828 | *mterfd1* | *MTERF3* | 0.775 | 6.51E-04 |
| NM_001079999 | Dr.69837 | *zgc:154088* | *SLC38A9* | 0.774 | 4.62E-03 |
| NM_001030233 | Dr.120310 | *zgc:113423* | *N.A.* | 0.772 | 4.84E-03 |
| NM_001045415 | Dr.82533 | *lcn15* | *PTGDS* | 0.771 | 5.42E-03 |
| NM_001113590 | Dr.90160 | *tnfsf13b* | *TNFSF13B* | 0.769 | 5.53E-03 |
| NM_214795 | Dr.84644 | *tph2* | *TPH2* | 0.769 | 5.09E-03 |
| NM_200238 | Dr.80438 | *vil1l* | *VIL1* | 0.768 | 6.11E-03 |
| NM_205679 | Dr.29769 | *cda* | *CDA* | 0.768 | 5.67E-03 |
| NM_001128338 | Dr.16338 | *si:dkey-18l1.1* | *KIAA0564* | 0.764 | 1.26E-04 |
| NM_001118897 | Dr.77557 | *evpla* | *EVPL* | 0.759 | 3.06E-03 |
| NM_001006008 | Dr.84765 | *zgc:103759* | *NUDT16* | 0.758 | 5.00E-03 |
| NM_001013574 | Dr.79930 | *zgc:113516* | *ETNK2* | 0.751 | 6.25E-03 |
| NM_001017701 | Dr.84739 | *mppe1* | *MPPE1* | 0.751 | 6.09E-03 |
| NM_131819 | Dr.77162 | *f7* | *F7* | 0.749 | 5.97E-03 |
| NM_178298 | Dr.8516 | *sepp1b* | *SEPP1B* | 0.749 | 7.79E-03 |
| NM_001030286 | Dr.83995 | *serac1* | *SERAC1* | 0.748 | 2.43E-04 |
| NM_001123308 | Dr.65762 | *ogg1* | *OGG1* | 0.746 | 6.41E-03 |
| NM_200616 | Dr.12563 | *zgc:63674* | *C1orf27* | 0.742 | 1.47E-03 |
| NM_212770 | Dr.1128 | *cpn1* | *CPN1* | 0.740 | 7.35E-03 |
| NM_001017603 | Dr.80506 | *acer1* | *ACER1* | 0.740 | 8.49E-03 |
| NM_001077468 | Dr.13693 | *zgc:153377* | *MRPL21* | 0.738 | 1.65E-03 |
| NM_131682 | Dr.8180 | *alas2* | *ALAS1* | 0.735 | 8.33E-03 |
| NM_200159 | Dr.81400 | *manba* | *MANBA* | 0.734 | 1.78E-04 |
| NM_001017867 | Dr.81730 | *gnpda1* | *GNPDA1* | 0.733 | 2.74E-03 |
| NM_001003556 | Dr.78016 | *dimt1l* | *DIMT1* | 0.733 | 6.26E-03 |
| NM_001076663 | Dr.138610 | *rabggta* | *RABGGTA* | 0.732 | 8.12E-03 |
| NM_001017648 | Dr.91734 | *dnajc28* | *DNAJC28* | 0.730 | 5.11E-03 |
| NM_001115060 | Dr.113696 | *cxcl-c1c* | *CXCL-C1C* | 0.730 | 8.96E-03 |
| NM_199812 | Dr.77648 | *zgc:66472* | *N.A.* | 0.729 | 7.69E-03 |
| NM_001114586 | Dr.27189 | *abca1b* | *NIPSNAP3B* | 0.729 | 1.54E-03 |
| NM_001080174 | Dr.83781 | *sirt3* | *RIC8A* | 0.727 | 1.29E-03 |
| NM_212620 | Dr.20277 | *acta2* | *ACTA1* | 0.726 | 5.84E-03 |
| NM_001007030 | Dr.80418 | *synj1* | *SYNJ1* | 0.721 | 1.03E-02 |
| NM_001018679 | Dr.33913 | *gys2* | *GYS2* | 0.721 | 8.02E-03 |
| NM_001077787 | Dr.89616 | *zgc:153638* | *DNAJB14* | 0.720 | 1.54E-03 |
| NM_201008 | Dr.24973 | *pfkfb2a* | *PFKFB3* | 0.719 | 8.82E-04 |
| NM_001003464 | Dr.83937 | *cldnk* | *CLDNK* | 0.715 | 1.04E-02 |
| NM_001001840 | Dr.117553 | *nfkb2* | *NFKB2* | 0.713 | 1.47E-07 |
| NM_199712 | Dr.79270 | *pigp* | *PIGP* | 0.712 | 2.93E-04 |
| NM_131591 | Dr.75552 | *actc1b* | *ACTA1* | 0.712 | 2.63E-03 |
| NM_001089536 | Dr.12523 | *cep128* | *CEP128* | 0.710 | 1.01E-02 |
| NM_200875 | Dr.76348 | *myof* | *MYOF* | 0.710 | 1.14E-02 |
| NM_182880 | Dr.57073 | *serpind1* | *SERPIND1* | 0.709 | 1.17E-02 |
| NM_201100 | Dr.86246 | *minpp1b* | *MINPP1* | 0.709 | 4.36E-03 |
| NM_001003513 | Dr.81587 | *traf3* | *TRAF3* | 0.709 | 1.39E-03 |
| NM_001163831 | Dr.22265 | *prdm14* | *PRDM14* | 0.708 | 1.14E-02 |
| NM_001007437 | Dr.77353 | *zgc:101663* | *N.A.* | 0.707 | 7.71E-05 |
| NM_001020626 | Dr.77673 | *c1qtnf3* | *AMACR* | 0.703 | 9.95E-03 |
| NM_001004118 | Dr.34264 | *gdpd1* | *GDPD1* | 0.700 | 8.65E-03 |
| NM_001025164 | Dr.159500 | *zgc:112265* | *ITIH4* | 0.698 | 8.54E-03 |
| NM_200335 | Dr.19506 | *med18* | *MED18* | 0.697 | 5.96E-03 |
| NM_001045127 | Dr.33839 | *si:dkey-44g23.5* | *FAM195A* | 0.696 | 2.05E-03 |
| NM_213007 | Dr.852 | *itih2* | *ITIH2* | 0.695 | 7.61E-03 |
| NM_001114415 | Dr.91374 | *si:ch211-245p7.3* | *HYDIN* | 0.694 | 1.02E-02 |
| NM_001256683 | Dr.140875 | *LOC100151273* | *N.A.* | 0.693 | 1.30E-02 |
| NM_001113641 | Dr.82576 | *si:ch211-170d8.5* | *N.A.* | 0.691 | 1.24E-02 |
| NM_199879 | Dr.20491 | *mgrn1a* | *MGRN1* | 0.688 | 7.71E-03 |
| NM_001204157 | Dr.33003 | *zdhhc6* | *ZDHHC6* | 0.686 | 8.00E-03 |
| NM_001004496 | Dr.75489 | *ofd1* | *OFD1* | 0.686 | 2.22E-03 |
| NM_001004566 | Dr.80590 | *manea* | *MANEAL* | 0.680 | 1.38E-02 |
| NM_200449 | Dr.106780 | *agxt2l1* | *AGXT2L2* | 0.678 | 1.16E-02 |
| NM_131619 | Dr.31421 | *mylz3* | *MYL1* | 0.678 | 6.21E-03 |
| NM_001040380 | Dr.75556 | *parpbp* | *PARPBP* | 0.677 | 1.61E-02 |
| NM_200627 | Dr.118384 | *cdkal1* | *CDKAL1* | 0.677 | 1.29E-03 |
| NM_131772 | Dr.77854 | *cldn11b* | *CLDN11B* | 0.675 | 1.58E-02 |
| NM_001002713 | Dr.30980 | *mfsd5* | *MFSD5* | 0.675 | 4.23E-03 |
| NM_201472 | Dr.77342 | *plg* | *PLG* | 0.673 | 1.55E-02 |
| NM_001003578 | Dr.152161 | *lpar2a* | *LPAR3* | 0.673 | 8.19E-04 |
| NM_001008582 | Dr.77047 | *zgc:103710* | *C3* | 0.672 | 1.43E-02 |
| NM_198072 | Dr.75737 | *mtr* | *MTR* | 0.672 | 1.67E-02 |
| NM_001045488 | Dr.75873 | *zgc:161979* | *N.A.* | 0.671 | 1.69E-02 |
| NM_001045232 | Dr.84312 | *sco2* | *SCO2* | 0.670 | 1.35E-02 |
| NM_001005963 | Dr.88686 | *cyp2k16* | *Cyp2ac1* | 0.669 | 1.55E-02 |
| NM_001135129 | Dr.115368 | *zgc:194768* | *UROC1* | 0.668 | 1.44E-02 |
| NM_001042775 | Dr.52856 | *abcg2a* | *ABCG2* | 0.668 | 1.77E-02 |
| NM_001024813 | Dr.40391 | *mcm10* | *MCM10* | 0.667 | 1.69E-02 |
| NM_001002312 | Dr.19771 | *ift172* | *IFT172* | 0.664 | 1.44E-02 |
| NM_001102616 | Dr.105118 | *tdo2a* | *TDO2* | 0.663 | 1.35E-02 |
| NM_001103111 | Dr.111174 | *mntb* | *MNT* | 0.662 | 2.58E-03 |
| NM_001100100 | Dr.105880 | *tsnaxip1* | *TSNAXIP1* | 0.661 | 1.05E-02 |
| NM_001126463 | Dr.24976 | *igfbp5a* | *IGFBP5* | 0.661 | 1.88E-02 |
| NM_001013341 | Dr.37497 | *ywhae2* | *YWHAE* | 0.660 | 1.71E-02 |
| NM_001243045 | Dr.78258 | *lamb2* | *LAMB1* | 0.659 | 1.11E-02 |
| NM_201319 | Dr.85558 | *pms1* | *PMS1* | 0.659 | 2.87E-03 |
| NM_200531 | Dr.132746 | *mfsd4a* | *MFSD4* | 0.658 | 1.68E-02 |
| NM_001083009 | Dr.80962 | *zgc:158782* | *MTO1* | 0.657 | 1.36E-03 |
| NM_212846 | Dr.87218 | *hbbe2* | *HBE1* | 0.657 | 1.40E-02 |
| NM_001013327 | Dr.17323 | *zgc:110805* | *METTL10* | 0.657 | 1.65E-02 |
| NM_001114440 | Dr.24856 | *slc4a2b* | *SLC4A2* | 0.655 | 2.77E-04 |
| NM_001077150 | Dr.74231 | *oxct1b* | *OXCT1* | 0.655 | 1.28E-02 |
| NM_001002586 | Dr.29899 | *cryba1b* | *CRYBA1* | 0.654 | 1.46E-02 |
| NM_001083823 | Dr.40360 | *cd22* | *SIGLEC1* | 0.653 | 3.31E-06 |
| NM_212859 | Dr.89727 | *tnfa* | *TNFA* | 0.652 | 1.70E-03 |
| NM_001030193 | Dr.133636 | *zgc:114081* | *FBXO6* | 0.650 | 2.09E-02 |
| NM_212893 | Dr.161770 | *dpydb* | *DPYD* | 0.649 | 1.86E-02 |
| NM_213418 | Dr.76941 | *nudt1* | *NUDT1* | 0.647 | 1.37E-02 |
| NM_001256177 | Dr.79676 | *cyp2x7* | *CYP2X7* | 0.647 | 1.56E-02 |
| NM_213224 | Dr.159616 | *gnl2* | *GNL2* | 0.644 | 1.59E-03 |
| NM_001076707 | Dr.80092 | *abi1b* | *ABI1* | 0.643 | 6.83E-03 |
| NM_212947 | Dr.121066 | *rsrc1* | *RSRC1* | 0.642 | 1.91E-02 |
| NM_001079979 | Dr.152360 | *zgc:153913* | *CPN2* | 0.639 | 1.77E-02 |
| NM_001282050 | N.A. | *N.A.* | *N.A.* | 0.638 | 1.33E-03 |
| NM_131248 | Dr.144146 | *egr1* | *EGR1* | 0.637 | 3.03E-03 |
| NM_001017750 | Dr.75118 | *acta1a* | *ACTA1* | 0.637 | 2.21E-02 |
| NM_001002182 | Dr.83427 | *zgc:91890* | *N.A.* | 0.636 | 2.32E-03 |
| NM_001044976 | Dr.106682 | *kif14* | *KIF14* | 0.635 | 2.40E-02 |
| NM_001030241 | Dr.47462 | *snrnp35* | *SNRNP35* | 0.634 | 2.40E-02 |
| NM_001044853 | Dr.80453 | *klhl26* | *KLHL26* | 0.633 | 1.97E-02 |
| NM_001214910 | Dr.7577 | *pogza* | *POGZA* | 0.633 | 1.95E-02 |
| NM_199859 | Dr.150659 | *abi3bp* | *ABI3BP* | 0.633 | 1.64E-02 |
| NM_200352 | Dr.16642 | *rtn4ip1* | *RTN4IP1* | 0.632 | 1.90E-02 |
| NM_213525 | Dr.6655 | *ciz1b* | *CIZ1* | 0.632 | 2.15E-02 |
| NM_001020785 | Dr.135567 | *il10* | *IL10* | 0.631 | 4.25E-03 |
| NM_001076556 | Dr.78718 | *slc35f2* | *SLC35F2* | 0.631 | 1.70E-02 |
| NM_001004568 | Dr.36552 | *cdc42ep3* | *CDC42EP3* | 0.631 | 1.38E-02 |
| NM_001172627 | Dr.37813 | *itga11a* | *ITGA11* | 0.631 | 2.20E-02 |
| NM_001004512 | Dr.81717 | *si:busm1-57f23.1* | *SI:BUSM1-57F23.1* | 0.630 | 2.45E-02 |
| NM_001079676 | Dr.84028 | *crfb8* | *IL20RA* | 0.630 | 2.34E-02 |
| NM_200396 | Dr.12674 | *coro6* | *CORO1B* | 0.629 | 2.09E-02 |
| NM_001083867 | Dr.84698 | *zgc:162301* | *YRDC* | 0.628 | 1.63E-02 |
| NM_001007405 | Dr.119922 | *rbm41* | *RBM41* | 0.628 | 1.97E-02 |
| NM_001005598 | Dr.134263 | *ttr* | *TTR* | 0.628 | 2.11E-02 |
| NM_001002748 | Dr.77360 | *zgc:100919* | *N.A.* | 0.627 | 3.80E-03 |
| NM_199788 | Dr.75667 | *fam212ab* | *FAM212A* | 0.627 | 1.43E-02 |
| NM_001017802 | Dr.133630 | *zgc:110329* | *N.A.* | 0.625 | 2.21E-02 |
| NM_001080619 | Dr.155217 | *slc39a3* | *SLC39A3* | 0.625 | 2.16E-02 |
| NM_001002621 | Dr.79889 | *gsto1* | *GSTO1* | 0.624 | 2.15E-02 |
| NM_212709 | Dr.75708 | *ppp1r3b* | *PPP1R3B* | 0.624 | 2.55E-02 |
| NM_200599 | Dr.81354 | *cxxc1l* | *CXXC1* | 0.623 | 1.67E-04 |
| NM_182863 | Dr.4199 | *serpinc1* | *SERPINC1* | 0.620 | 1.66E-02 |
| NM_001077744 | Dr.41325 | *otud7b* | *OTUD7B* | 0.620 | 2.34E-02 |
| NM_001040306 | Dr.79892 | *kmo* | *KMO* | 0.617 | 2.76E-02 |
| NM_001044978 | Dr.150777 | *ddx59* | *DDX59* | 0.616 | 1.67E-02 |
| NM_001082812 | Dr.90264 | *cmklr1* | *CMKLR1* | 0.616 | 1.12E-08 |
| NM_001045393 | Dr.47954 | *zgc:153044* | *N.A.* | 0.615 | 2.88E-02 |
| NM_001126446 | Dr.86116 | *piga* | *PIGA* | 0.615 | 2.72E-02 |
| NM_001008538 | Dr.37123 | *pygl* | *PYGM* | 0.615 | 2.24E-02 |
| NM_001139479 | Dr.75455 | *a2ml* | *A2M* | 0.614 | 2.14E-02 |
| NM_001005978 | Dr.80301 | *glipr2l* | *GLIPR2* | 0.613 | 2.66E-02 |
| NM_001002741 | Dr.14523 | *rnpep* | *RNPEP* | 0.612 | 2.78E-02 |
| NM_001040304 | Dr.49103 | *gmpr2* | *GMPR2* | 0.611 | 3.32E-04 |
| NM_200614 | Dr.85225 | *zgc:63663* | *HSPA1A* | 0.610 | 2.70E-02 |
| NM_001256223 | Dr.88768 | *im:6903726* | *PTGR2* | 0.608 | 4.18E-03 |
| NM_001017640 | Dr.83969 | *akr1a1b* | *AKR1A1B* | 0.608 | 1.15E-02 |
| NM_001170836 | Dr.118434 | *znf574* | *ZNF574* | 0.607 | 2.92E-02 |
| NM_001173406 | Dr.150159 | *LOC798080* | *PROKR1* | 0.607 | 7.17E-03 |
| NM_131520 | Dr.51894 | *fn1* | *FN1* | 0.607 | 3.11E-02 |
| NM_001083123 | Dr.75914 | *ar* | *AR* | 0.606 | 3.84E-04 |
| NM_001100137 | Dr.76551 | *zgc:165344* | *MYBPH* | 0.606 | 1.94E-02 |
| NM_001082940 | Dr.83037 | *mafa* | *MAFA* | 0.605 | 1.38E-02 |
| NM_131188 | Dr.75115 | *mylpfa* | *MYLPF* | 0.603 | 3.15E-02 |
| NM_001098386 | Dr.115281 | *wu:fc61g08* | *USP13* | 0.603 | 1.58E-02 |
| NM_001082982 | Dr.91948 | *si:ch211-89p1.1* | *F11R* | 0.602 | 2.52E-02 |
| NM_001130597 | Dr.134880 | *zgc:193538* | *N.A.* | 0.601 | 3.26E-02 |
| NM_001018125 | Dr.75113 | *cryba4* | *CRYBA1* | 0.600 | 2.75E-02 |
| NM_212583 | Dr.30340 | *slc39a1* | *SLC39A1* | 0.599 | 3.12E-02 |
| NM_131186 | Dr.22 | *nr2f5* | *NR2F5* | 0.599 | 1.94E-02 |
| NM_001079981 | Dr.79345 | *isoc1* | *ISOC1* | 0.598 | 8.65E-04 |
| NM_001007400 | Dr.22212 | *cyp3c1l2* | *CYP3A7* | 0.598 | 3.01E-02 |
| NM_001114895 | Dr.52137 | *zgc:171534* | *N.A.* | 0.597 | 2.81E-02 |
| NM_152980 | Dr.118210 | *mst1* | *MST1* | 0.597 | 3.40E-02 |
| NM_001089458 | Dr.87867 | *cyp24a1* | *CYP24A1* | 0.596 | 3.41E-02 |
| NM_001017837 | Dr.107611 | *zgc:110182* | *FAM84B* | 0.596 | 1.35E-02 |
| NM_001014357 | Dr.84239 | *zgc:112970* | *N.A.* | 0.596 | 1.54E-02 |
| NM_001100437 | Dr.113769 | *crybb1l2* | *CRYBB1L2* | 0.595 | 2.58E-02 |
| NM_200893 | Dr.81416 | *ampd1* | *AMPD1* | 0.592 | 3.19E-02 |
| NM_001045360 | Dr.77055 | *cyb5r2* | *CYB5R2* | 0.590 | 2.96E-02 |
| NM_001017734 | Dr.76505 | *steap4* | *STEAP4* | 0.589 | 3.01E-02 |
| NM_001017807 | Dr.1945 | *capn2l* | *CAPN2* | 0.588 | 1.92E-02 |
| NM_001045845 | Dr.83024 | *zgc:153293* | *N.A.* | 0.588 | 3.10E-03 |
| NM_131525 | Dr.75139 | *sptb* | *SPTB* | 0.588 | 2.94E-02 |
| NM_001159970 | Dr.110802 | *s1pr2* | *S1PR2* | 0.588 | 9.47E-03 |
| NM_001017602 | Dr.33948 | *arf4a* | *ARF5* | 0.585 | 2.89E-02 |
| NM_001083553 | Dr.85263 | *txlnbb* | *TXLNB* | 0.584 | 3.32E-02 |
| NM_001003519 | Dr.33963 | *pltp* | *PLTP* | 0.581 | 3.61E-02 |
| NM_001277130 | N.A. | *N.A.* | *N.A.* | 0.578 | 3.64E-02 |
| NM_001076622 | Dr.89419 | *itln1* | *ITLN1* | 0.578 | 3.96E-02 |
| NM_001017892 | Dr.75751 | *zgc:113208* | *N.A.* | 0.577 | 2.08E-02 |
| NM_001045410 | Dr.159102 | *tmem150a* | *TMEM150A* | 0.577 | 7.41E-03 |
| NM_200345 | Dr.26462 | *zdhhc16b* | *ZDHHC16* | 0.576 | 2.36E-02 |
| NM_001044965 | Dr.43652 | *si:ch211-260p9.6* | *KIAA1609* | 0.575 | 2.25E-02 |
| NM_001017900 | Dr.89885 | *zgc:110602* | *SLC35A3* | 0.573 | 3.77E-02 |
| NM_001025519 | Dr.40488 | *zgc:113426* | *N.A.* | 0.573 | 4.10E-02 |
| NM_212873 | Dr.106930 | *lgals3bpb* | *LGALS3BP* | 0.573 | 3.20E-02 |
| NM_001077155 | Dr.40163 | *zgc:153218* | *NARS2* | 0.573 | 4.14E-02 |
| NM_200915 | Dr.82331 | *rbm17* | *RBM17* | 0.572 | 1.88E-02 |
| NM_201500 | Dr.79367 | *elovl6l* | *ELOVL6* | 0.572 | 3.07E-02 |
| NM_200370 | Dr.150526 | *zgc:64043* | *TMEM254* | 0.572 | 3.77E-02 |
| NM_213371 | Dr.78785 | *tmem161b* | *TMEM161B* | 0.572 | 4.95E-04 |
| NM_001006009 | Dr.12697 | *zgc:103755* | *CLIP4* | 0.571 | 1.35E-02 |
| NM_001001838 | Dr.79693 | *top1mt* | *TOP1MT* | 0.570 | 4.05E-02 |
| NM_001098388 | Dr.76547 | *zcchc24* | *ZCCHC24* | 0.570 | 3.07E-02 |
| NM_001115059 | Dr.120512 | *tgfbr1b* | *TGFBR1* | 0.570 | 2.41E-03 |
| NM_201175 | Dr.132376 | *oxsr1b* | *OXSR1* | 0.570 | 4.11E-02 |
| NM_001162847 | Dr.78676 | *si:ch211-225p5.3* | *N.A.* | 0.570 | 4.10E-02 |
| NM_001002492 | Dr.90598 | *cmtm4* | *CMTM4* | 0.570 | 4.15E-02 |
| NM_001034971 | Dr.134213 | *itgb1a* | *ITGB1* | 0.569 | 3.85E-02 |
| NM_153662 | Dr.31925 | *st8sia2* | *ST8SIA2* | 0.569 | 2.17E-02 |
| NM_001029957 | Dr.47530 | *chac2* | *CHAC2* | 0.568 | 4.28E-02 |
| NM_130932 | Dr.75117 | *ckma* | *CKM* | 0.567 | 1.01E-02 |
| NM_001114572 | Dr.90327 | *zgc:175274* | *PIGW* | 0.566 | 4.03E-02 |
| NM_001144055 | Dr.15176 | *si:dkey-69o16.5* | *N.A.* | 0.566 | 2.22E-02 |
| NM_214807 | Dr.89723 | *fabp7b* | *FABP7* | 0.566 | 3.71E-02 |
| NM_001002647 | Dr.88155 | *zgc:92020* | *EGR3* | 0.566 | 6.99E-05 |
| NM_001113377 | Dr.142587 | *csf3r* | *CSF3R* | 0.566 | 2.71E-05 |
| NM_001204123 | Dr.36658 | *gpatch1* | *GPATCH1* | 0.563 | 6.25E-03 |
| NM_001002176 | Dr.117370 | *tha1* | *THA1* | 0.563 | 1.84E-02 |
| NM_001143751 | Dr.154668 | *erbb4a* | *ERBB4* | 0.563 | 2.59E-02 |
| NM_199550 | Dr.77236 | *naa35* | *NAA35* | 0.563 | 5.54E-05 |
| NM_001083570 | Dr.132661 | *errfi1* | *ERRFI1* | 0.562 | 2.96E-02 |
| NM_001128774 | Dr.113445 | *zgc:195152* | *N.A.* | 0.562 | 4.28E-02 |
| NM_001039639 | Dr.40859 | *abcg2c* | *ABCG2C* | 0.562 | 3.03E-03 |
| NM_001256719 | Dr.90214 | *dhrsx* | *DHRSX* | 0.562 | 4.28E-02 |
| NM_131110 | Dr.32297 | *cahz* | *CA13* | 0.561 | 3.85E-02 |
| NM_001113638 | Dr.76192 | *zgc:171551* | *ZNF568* | 0.559 | 3.90E-02 |
| NM_001100144 | Dr.3564 | *apoa1b* | *APOA1B* | 0.559 | 2.32E-02 |
| NM_001002752 | Dr.88142 | *yeats4* | *YEATS4* | 0.559 | 1.11E-03 |
| NM_212820 | Dr.104315 | *cnr1* | *CNR1* | 0.558 | 3.64E-02 |
| NM_001166353 | Dr.104637 | *amdhd1* | *AMDHD1* | 0.557 | 4.40E-02 |
| NM_001003830 | Dr.76891 | *noc2l* | *NOC2L* | 0.557 | 2.13E-02 |
| NM_205605 | Dr.18243 | *wdr91* | *WDR91* | 0.557 | 1.20E-03 |
| NM_001017539 | Dr.77336 | *efha1* | *EFHA1* | 0.556 | 1.68E-03 |
| NM_001080644 | Dr.128545 | *trappc12* | *TRAPPC12* | 0.556 | 3.73E-02 |
| NM_001044331 | Dr.78570 | *siae* | *SIAE* | 0.555 | 1.76E-02 |
| NM_001045341 | Dr.79547 | *cryl1* | *CRYL1* | 0.555 | 1.06E-03 |
| NM_001130783 | Dr.78912 | *plekhm2* | *PLEKHM2* | 0.554 | 4.19E-02 |
| NM_200389 | Dr.84295 | *obfc1* | *OBFC1* | 0.554 | 1.18E-02 |
| NM_001030154 | Dr.94171 | *abcc9* | *ABCC9* | 0.554 | 3.01E-02 |
| NM_130918 | Dr.32845 | *fzd8a* | *FZD8* | 0.554 | 4.13E-02 |
| NM_001077282 | Dr.35680 | *nedd9* | *NEDD9* | 0.553 | 2.36E-03 |
| NM_212800 | Dr.48573 | *disp1* | *DISP1* | 0.552 | 4.58E-02 |
| NM_001110404 | Dr.78834 | *rnf180* | *RNF180* | 0.552 | 2.65E-02 |
| NM_001201463 | Dr.22359 | *chic1* | *CHIC1* | 0.551 | 2.91E-02 |
| NM_001020728 | Dr.45793 | *fahd1* | *FAHD1* | 0.551 | 4.41E-02 |
| NM_001159584 | Dr.117747 | *itln3* | *ITLN1* | 0.550 | 2.33E-02 |
| NM_001202459 | Dr.108454 | *jarid2b* | *JARID2* | 0.550 | 4.77E-02 |
| NM_001100093 | Dr.38615 | *ercc6l* | *ERCC6L* | 0.548 | 3.96E-02 |
| NM_001004579 | Dr.132813 | *pip5k1bb* | *PIP5K1B* | 0.547 | 7.96E-05 |
| NM_200642 | Dr.87301 | *zgc:64201* | *Prorsd1* | 0.547 | 5.00E-02 |
| NM_001100147 | Dr.88502 | *zgc:158376* | *FBXL19* | 0.546 | 4.69E-02 |
| NM_001193541 | Dr.75528 | *smc5* | *SMC5* | 0.545 | 3.52E-03 |
| NM_200629 | Dr.80610 | *zgc:63489* | *N.A.* | 0.545 | 1.98E-02 |
| NM_131218 | Dr.75068 | *lhx5* | *LHX5* | 0.543 | 3.47E-02 |
| NM_131406 | Dr.193 | *raraa* | *RARA* | 0.542 | 1.17E-02 |
| NM_001003417 | Dr.17702 | *b4galt7* | *B4GALT7* | 0.542 | 4.83E-02 |
| NM_001082823 | Dr.4479 | *si:ch211-244o22.2* | *CDCA2* | 0.541 | 4.96E-02 |
| NM_001130595 | Dr.89707 | *tlr5b* | *TLR5* | 0.540 | 6.93E-03 |
| NM_001256633 | Dr.103965 | *atox1* | *ATOX1* | 0.540 | 5.87E-03 |
| NM_001008620 | Dr.83537 | *tsta3* | *TSTA3* | 0.539 | 4.50E-02 |
| NM_001114432 | Dr.93146 | *ramp2* | *RAMP2* | 0.539 | 1.33E-02 |
| NM_001004001 | Dr.34238 | *zgc:101016* | *C9orf85* | 0.539 | 5.19E-03 |
| NM_001110102 | Dr.83128 | *chtf18* | *CHTF18* | 0.539 | 2.75E-02 |
| NM_001007388 | Dr.37357 | *ttc36* | *TTC36* | 0.538 | 4.04E-02 |
| NM_200422 | Dr.83940 | *btr30* | *BTR30* | 0.538 | 4.63E-02 |
| NM_001080753 | Dr.81117 | *zgc:153243* | *N.A.* | 0.538 | 2.42E-02 |
| NM_001079990 | Dr.87827 | *dync1i1* | *DYNC1I1* | 0.538 | 4.18E-02 |
| NM_001080562 | Dr.7668 | *zgc:158605* | *N.A.* | 0.537 | 1.23E-02 |
| NM_001201408 | Dr.88279 | *LOC564844* | *N.A.* | 0.537 | 3.44E-02 |
| NM_200623 | Dr.4658 | *wdr46* | *WDR46* | 0.536 | 2.77E-03 |
| NM_001014337 | Dr.89430 | *zgc:113176* | *N.A.* | 0.536 | 2.91E-02 |
| NM_173226 | Dr.76718 | *thbs4b* | *THBS4* | 0.536 | 2.78E-02 |
| NM_131843 | Dr.10736 | *tnfsf10l* | *TNFSF10L* | 0.536 | 2.72E-02 |
| NM_200305 | Dr.82400 | *dnajb6b* | *DNAJB6* | 0.536 | 3.10E-02 |
| NM_001030210 | Dr.94164 | *cx32.2* | *CX32.2* | 0.535 | 4.37E-02 |
| NM_200118 | Dr.26707 | *poc1b* | *POC1B* | 0.534 | 1.39E-02 |
| NM_001105527 | Dr.114568 | *zgc:171711* | *C19orf66* | 0.534 | 2.99E-02 |
| NM_199792 | Dr.26610 | *znf710a* | *ZNF710* | 0.533 | 1.21E-03 |
| NM_001126397 | Dr.77748 | *LOC560826* | *CSGALNACT2* | 0.533 | 3.39E-02 |
| NM_001002638 | Dr.10354 | *zgc:92203* | *PREB* | 0.533 | 8.02E-03 |
| NM_001079861 | Dr.77089 | *apoa4* | *APOA4* | 0.533 | 4.17E-02 |
| NM_001122755 | Dr.56035 | *bnc2* | *BNC2* | 0.533 | 4.03E-02 |
| NM_001130791 | Dr.109685 | *plbd1* | *PLBD1* | 0.532 | 4.96E-03 |
| NM_131251 | Dr.334 | *otx2* | *OTX2* | 0.532 | 3.56E-02 |
| NM_001130767 | Dr.18791 | *prmt10* | *PRMT10* | 0.531 | 3.57E-02 |
| NM_001025524 | Dr.83278 | *zc3h14* | *EML5* | 0.530 | 9.52E-05 |
| NM_200916 | Dr.118099 | *panx1a* | *PANX1* | 0.529 | 2.78E-02 |
| NM_001080038 | Dr.37679 | *zgc:158343* | *N.A.* | 0.529 | 3.86E-02 |
| NM_001077160 | Dr.83672 | *zgc:152954* | *KBTBD2* | 0.528 | 1.93E-02 |
| NM_198921 | Dr.80542 | *scarb1* | *SCARB1* | 0.526 | 3.44E-02 |
| NM_001079974 | Dr.75979 | *haus6* | *HAUS6* | 0.525 | 1.57E-02 |
| NM_001114313 | Dr.85033 | *zgc:172359* | *HEATR6* | 0.524 | 4.30E-02 |
| NM_001146149 | Dr.134092 | *pax7b* | *PAX7* | 0.524 | 4.23E-02 |
| NM_001128541 | Dr.114985 | *LOC567472* | *N.A.* | 0.524 | 3.31E-03 |
| NM_001045096 | Dr.85472 | *recql* | *RECQL* | 0.523 | 4.40E-02 |
| NM_001109712 | Dr.89295 | *zgc:171731* | *CASP1* | 0.522 | 2.34E-02 |
| NM_001037392 | Dr.92067 | *trmt6* | *TRMT6* | 0.522 | 2.40E-02 |
| NM_001005934 | Dr.80185 | *gpd1l* | *GPD1L* | 0.522 | 4.73E-02 |
| NM_213205 | Dr.77980 | *m6pr* | *M6PR* | 0.521 | 1.60E-02 |
| NM_199581 | Dr.77094 | *pola2* | *POLA2* | 0.518 | 3.59E-03 |
| NM_001002429 | Dr.80944 | *wdr83* | *WDR83* | 0.517 | 3.87E-02 |
| NM_001045100 | Dr.74757 | *snx27a* | *SNX27* | 0.517 | 5.80E-03 |
| NM_001017715 | Dr.81352 | *zgc:112183* | *RAB37* | 0.517 | 4.14E-04 |
| NM_001077161 | Dr.108194 | *znf438* | *ZNF438* | 0.517 | 1.91E-02 |
| NM_001082821 | Dr.76194 | *atpaf2* | *ATPAF2* | 0.516 | 4.72E-02 |
| NM_001030199 | Dr.76365 | *sat1a* | *SAT1* | 0.516 | 3.59E-02 |
| NM_001079982 | Dr.75501 | *aggf1* | *AGGF1* | 0.515 | 4.65E-02 |
| NM_201182 | Dr.80335 | *bcs1l* | *BCS1L* | 0.515 | 4.17E-02 |
| NM_001003521 | Dr.77429 | *hsd17b14* | *HSD17B14* | 0.514 | 6.75E-03 |
| NM_001045031 | Dr.74235 | *si:ch211-154o6.3* | *SI:CH211-154O6.3* | 0.513 | 4.22E-02 |
| NM_001002204 | Dr.78187 | *prpf18* | *PRPF18* | 0.512 | 5.95E-03 |
| NM_001006046 | Dr.86992 | *cutc* | *CUTC* | 0.510 | 1.25E-02 |
| NM_001020710 | Dr.79121 | *pskh1* | *PSKH1* | 0.510 | 4.40E-02 |
| NM_001077252 | Dr.37700 | *ces2* | *CES2* | 0.509 | 2.84E-02 |
| NM_001122858 | Dr.77212 | *masp2* | *MASP2* | 0.508 | 4.92E-02 |
| NM_001044996 | Dr.108349 | *camsap2* | *CAMSAP2* | 0.508 | 4.78E-02 |
| NM_205670 | Dr.89035 | *tmem88a* | *TMEM88* | 0.508 | 4.45E-02 |
| NM_001030062 | Dr.76570 | *apobl* | *APOBL* | 0.507 | 3.99E-02 |
| NM_001080051 | Dr.115399 | *cntn4* | *CNTN3* | 0.506 | 4.85E-02 |
| NM_001045279 | Dr.92622 | *crfb9* | *IL22RA2* | 0.504 | 3.97E-02 |
| NM_001126456 | Dr.41215 | *irg1* | *IRG1* | 0.503 | 1.02E-02 |
| NM_001003998 | Dr.34241 | *bet1l* | *BET1L* | 0.503 | 2.40E-02 |
| NM_001005985 | Dr.78666 | *rwdd* | *RWDD4* | 0.503 | 3.22E-02 |
| NM_001037375 | Dr.47591 | *etv2* | *ETV2* | 0.503 | 3.21E-02 |
| NM_212624 | Dr.75843 | *chpt1* | *CHPT1* | 0.503 | 4.09E-02 |
| NM_001008583 | Dr.78424 | *cd2ap* | *CD2AP* | 0.502 | 3.48E-02 |
| NM_001033752 | Dr.151704 | *cwf19l2* | *CWF19L2* | 0.502 | 1.89E-02 |
| NM_131461 | Dr.75820 | *tie2* | *TEK* | 0.501 | 2.46E-02 |
| NM_001012366 | Dr.33521 | *krcp* | *KRCP* | 0.501 | 3.94E-03 |
| NM_001015041 | Dr.42794 | *ctgfa* | *CTGFA* | 0.498 | 3.63E-02 |
| NM_200270 | Dr.82812 | *zgc:56576* | *C14orf166* | 0.497 | 2.14E-02 |
| NM_200949 | Dr.6001 | *zfand5b* | *ZFAND5* | 0.497 | 3.73E-04 |
| NM_212747 | Dr.7400 | *csnk1e* | *CSNK1D* | 0.496 | 4.90E-02 |
| NM_001110372 | Dr.142520 | *lrrc47* | *LRRC47* | 0.496 | 1.41E-02 |
| NM_001004538 | Dr.36508 | *gyltl1b* | *LARGE* | 0.496 | 4.78E-02 |
| NM_001076586 | Dr.133461 | *zgc:153663* | *ABI1* | 0.496 | 4.60E-02 |
| NM_205590 | Dr.81780 | *pane1* | *CENPM* | 0.496 | 1.71E-02 |
| NM_213184 | Dr.79912 | *nfkbiaa* | *NFKBIA* | 0.495 | 3.28E-02 |
| NM_001278819 |  |  | *GAS7* | 0.495 | 4.84E-02 |
| NM_201012 | Dr.83796 | *nek4* | *NEK4* | 0.495 | 4.79E-02 |
| NM_001045125 | Dr.67361 | *cdc42bpb* | *CDC42BPB* | 0.494 | 4.90E-02 |
| NM_001014320 | Dr.86354 | *ppp1r13l* | *PPP1R13L* | 0.494 | 4.61E-02 |
| NM_199768 | Dr.78679 | *mynn* | *MYNN* | 0.494 | 9.98E-04 |
| NM_001002369 | Dr.77102 | *zgc:92502* | *AKR7A3* | 0.494 | 6.91E-03 |
| NM_001024811 | Dr.78829 | *gtpbp1l* | *GTPBP1* | 0.493 | 2.19E-02 |
| NM_001077625 | Dr.91624 | *il10ra* | *IL10RA* | 0.492 | 1.01E-03 |
| NM_199875 | Dr.80704 | *elovl7a* | *ELOVL7* | 0.490 | 4.47E-02 |
| NM_212940 | Dr.30341 | *lnx2b* | *LNX2B* | 0.490 | 4.54E-02 |
| NM_001076742 | Dr.43835 | *enosf1* | *TYMS* | 0.490 | 4.55E-02 |
| NM_001127516 | Dr.78853 | *hmox1* | *HMOX1* | 0.490 | 6.11E-04 |
| NM_001172308 | Dr.162139 | *LOC100003647* | *N.A.* | 0.489 | 2.65E-03 |
| NM_001030111 | Dr.91502 | *fstl3* | *FSTL3* | 0.488 | 4.89E-02 |
| NM_001278254 | N.A. | *N.A.* | *N.A.* | 0.484 | 3.49E-02 |
| NM_212690 | Dr.4036 | *plaa* | *PLAA* | 0.484 | 2.84E-02 |
| NM_001017633 | Dr.39081 | *zgc:110239* | *N.A.* | 0.484 | 4.28E-02 |
| NM_001111162 | Dr.23936 | *si:rp71-1g18.1* | *SI:RP71-1G18.1* | 0.483 | 1.33E-02 |
| NM_001098766 | Dr.85599 | *zgc:165573* | *CYSTM1* | 0.483 | 2.49E-03 |
| NM_212798 | Dr.82374 | *slc2a8* | *SLC2A8* | 0.481 | 3.03E-02 |
| NM_001114580 | Dr.162097 | *march7* | *MARCH7* | 0.481 | 3.82E-03 |
| NM_201144 | Dr.78565 | *rad54l* | *RAD54L* | 0.478 | 3.41E-02 |
| NM_205728 | Dr.99315 | *gli3* | *GLI3* | 0.478 | 4.78E-02 |
| NM_001017626 | Dr.42999 | *pcyox1* | *PCYOX1* | 0.478 | 1.36E-03 |
| NM_201071 | Dr.84976 | *mcts1* | *MCTS1* | 0.478 | 6.96E-04 |
| NM_001013519 | Dr.26870 | *slc25a44b* | *SLC25A44* | 0.477 | 4.54E-02 |
| NM_001270973 |  |  | *TMEM209* | 0.477 | 1.78E-03 |
| NM_001001841 | Dr.86023 | *rel* | *REL* | 0.475 | 3.13E-04 |
| NM_200221 | Dr.119477 | *stx4* | *STX4* | 0.472 | 3.18E-02 |
| NM_200317 | Dr.14012 | *chuk* | *CHUK* | 0.472 | 1.34E-02 |
| NM_001045215 | Dr.154223 | *foxg1c* | *FOXG1C* | 0.471 | 2.78E-02 |
| NM_214686 | Dr.3583 | *sult6b1* | *SULT6B1* | 0.469 | 1.89E-05 |
| NM_131140 | Dr.12607 | *fzd2* | *FZD2* | 0.465 | 4.64E-02 |
| NM_001135986 | Dr.118009 | *zgc:194246* | *N.A.* | 0.465 | 2.72E-02 |
| NM_001077722 | Dr.133033 | *zgc:152898* | *TPCN2* | 0.465 | 3.11E-02 |
| NM_001002433 | Dr.123434 | *dnajc9* | *DNAJC9* | 0.463 | 8.32E-03 |
| NM_212844 | Dr.30443 | *il1b* | *IL1B* | 0.463 | 1.37E-02 |
| NM_201181 | Dr.76592 | *acadl* | *ACADL* | 0.462 | 2.55E-03 |
| NM_200964 | Dr.77523 | *atf3* | *ATF3* | 0.461 | 9.39E-03 |
| NM_001034980 | Dr.134790 | *vti1a* | *VTI1A* | 0.460 | 1.98E-02 |
| NM_001037704 | Dr.39150 | *nenf* | *NENF* | 0.459 | 1.59E-02 |
| NM_001042695 | Dr.162020 | *casp2* | *CASP2* | 0.457 | 8.31E-03 |
| NM_212983 | Dr.26997 | *zgc:66427* | *N.A.* | 0.456 | 3.03E-04 |
| NM_001044855 | Dr.79865 | *vegfab* | *VEGFA* | 0.455 | 3.64E-02 |
| NM_153663 | Dr.76573 | *spns1* | *SPNS1* | 0.455 | 7.43E-04 |
| NM_001145607 | Dr.31772 | *si:dkey-20i10.6* | *ATP10D* | 0.455 | 1.40E-02 |
| NM_001037708 | Dr.128959 | *vps11* | *VPS11* | 0.454 | 9.65E-03 |
| NM_001020700 | Dr.77702 | *dbnlb* | *DBNL* | 0.452 | 2.40E-03 |
| NM_200515 | Dr.159924 | *pank4* | *PANK4* | 0.449 | 3.36E-02 |
| NM_205681 | Dr.84266 | *amdhd2* | *AMDHD2* | 0.448 | 1.27E-02 |
| NM_199629 | Dr.77409 | *nfkbiab* | *NFKBIA* | 0.447 | 1.32E-02 |
| NM_001045442 | Dr.83804 | *zgc:153247* | *EARS2* | 0.446 | 2.62E-02 |
| NM_001003469 | Dr.85497 | *zgc:91862* | *KXD1* | 0.446 | 4.51E-02 |
| NM_212565 | Dr.77989 | *dusp5* | *DUSP5* | 0.445 | 1.38E-03 |
| NM_001008628 | Dr.78644 | *pink1* | *PINK1* | 0.445 | 4.23E-02 |
| NM_001017656 | Dr.78018 | *pdss1* | *PDSS1* | 0.445 | 2.34E-02 |
| NM_207062 | Dr.76277 | *klf12a* | *KLF12* | 0.444 | 2.39E-03 |
| NM_001082994 | Dr.84225 | *tbl1x* | *TBL1X* | 0.443 | 2.61E-03 |
| NM_212740 | Dr.76172 | *fuca1* | *FUCA1* | 0.442 | 1.58E-02 |
| NM_001122856 | Dr.43382 | *glb1l* | *GLB1L* | 0.441 | 1.78E-02 |
| NM_001144813 | Dr.79952 | *LOC799527* | *N.A.* | 0.441 | 4.96E-02 |
| NM_213373 | Dr.76569 | *znf330* | *ZNF330* | 0.438 | 9.86E-03 |
| NM_001003997 | Dr.76199 | *flad1* | *FLAD1* | 0.437 | 4.08E-03 |
| NM_001020500 | Dr.88697 | *cybrd1* | *CYBRD1* | 0.436 | 2.88E-03 |
| NM_131539 | Dr.78102 | *pim1* | *PIM2* | 0.434 | 6.76E-03 |
| NM_194388 | Dr.11310 | *tuba1* | *TUBA1B* | 0.432 | 1.49E-02 |
| NM_001076631 | Dr.83081 | *zgc:153696* | *Zfp740* | 0.429 | 3.81E-02 |
| NM_001013521 | Dr.14149 | *zgc:103638* | *N.A.* | 0.428 | 3.18E-02 |
| NM_001130606 | Dr.78779 | *wdtc1* | *WDTC1* | 0.427 | 4.31E-02 |
| NM_200020 | Dr.159465 | *btg1* | *BTG1* | 0.426 | 8.33E-03 |
| NM_001020465 | Dr.34351 | *zgc:113271* | *RNF130* | 0.425 | 5.25E-04 |
| NM_001077212 | Dr.77671 | *dctn1a* | *DCTN1* | 0.424 | 3.46E-02 |
| NM_001100039 | Dr.106046 | *arid3a* | *ARID3A* | 0.422 | 4.86E-02 |
| NM_213153 | Dr.31936 | *dnase1l3* | *DNASE1L3* | 0.422 | 2.99E-04 |
| NM_001083850 | Dr.8355 | *rilpl2* | *RILPL2* | 0.421 | 2.23E-02 |
| NM_199603 | Dr.77223 | *zgc:65788* | *CHIA* | 0.418 | 2.08E-02 |
| NM_001123373 |  |  | *ASPM* | 0.418 | 1.75E-02 |
| NM_001040040 | Dr.92586 | *illr4* | *ILLR4* | 0.416 | 4.30E-02 |
| NM_001045396 | Dr.37156 | *zgc:153172* | *MRPS9* | 0.413 | 2.01E-02 |
| NM_001002631 | Dr.82562 | *dffa* | *DFFA* | 0.413 | 1.70E-02 |
| NM_001030148 | Dr.37134 | *paqr3b* | *PAQR3* | 0.413 | 4.17E-02 |
| NM_001029968 | Dr.81203 | *nif3l1* | *NIF3L1* | 0.408 | 1.80E-02 |
| NM_001111178 | Dr.74542 | *abhd16a* | *ABHD16A* | 0.407 | 9.12E-03 |
| NM_201292 | Dr.75513 | *ap3m1* | *AP3M1* | 0.404 | 1.58E-02 |
| NM_001077575 | Dr.87177 | *trip4* | *TRIP4* | 0.404 | 4.74E-02 |
| NM_001002215 | Dr.76590 | *zgc:92357* | *MGST1* | 0.403 | 4.21E-02 |
| NM_001077785 | Dr.40449 | *zgc:153996* | *VPS37C* | 0.403 | 1.96E-02 |
| NM_001114412 | Dr.83105 | *zgc:172352* | *NCKAP1L* | 0.403 | 2.10E-02 |
| NM_001002105 | Dr.3719 | *cdk5rap3* | *CDK5RAP3* | 0.402 | 4.55E-02 |
| NM_001025485 | Dr.81608 | *ccdc22* | *CCDC22* | 0.400 | 3.54E-03 |
| NM_001002315 | Dr.107920 | *ccm2* | *CCM2* | 0.399 | 4.08E-03 |
| NM_001142563 | Dr.17394 | *lmf2b* | *LMF2* | 0.397 | 2.36E-02 |
| NM_001080046 | Dr.5887 | *gpr107* | *GPR107* | 0.396 | 2.07E-02 |
| NM_001045552 | Dr.80740 | *mapk6* | *MAPK6* | 0.395 | 7.77E-03 |
| NM_212961 | Dr.3533 | *cdk7* | *CDK7* | 0.395 | 3.22E-02 |
| NM_001076672 | Dr.87599 | *ptk6b* | *PTK6* | 0.395 | 3.01E-02 |
| NM_001003761 | Dr.6946 | *zgc:101127* | *TMEM167B* | 0.395 | 4.44E-02 |
| NM_001146304 | Dr.60457 | *ralgapa1* | *RALGAPA1* | 0.393 | 3.59E-03 |
| NM_001030114 | Dr.75742 | *mrps18a* | *MRPS18A* | 0.393 | 4.47E-02 |
| NM_001007363 | Dr.37364 | *dhrs13a.1* | *DHRS13* | 0.392 | 3.74E-02 |
| NM_200092 | Dr.78455 | *bcap31* | *BCAP31* | 0.391 | 3.54E-03 |
| NM_001044334 | Dr.14745 | *ipo8* | *IPO8* | 0.390 | 3.33E-02 |
| NM_001007317 | Dr.79749 | *zgc:92177* | *GEMIN8P4* | 0.387 | 4.22E-02 |
| NM_001098777 | Dr.81449 | *sap130a* | *SAP130* | 0.387 | 4.99E-02 |
| NM_212910 | Dr.83170 | *stx11a* | *STX11* | 0.386 | 3.43E-02 |
| NM_194395 | Dr.77093 | *birc2* | *BIRC3* | 0.385 | 5.94E-03 |
| NM_201327 | Dr.77292 | *ssr1* | *SSR1* | 0.385 | 3.82E-02 |
| NM_175042 | Dr.80755 | *ttk* | *TTK* | 0.384 | 1.89E-02 |
| NM_205654 | Dr.6046 | *zgc:77262* | *N.A.* | 0.383 | 1.44E-02 |
| NM_001013316 | Dr.78557 | *tmem222a* | *TMEM222* | 0.382 | 4.30E-02 |
| NM_200912 | Dr.83728 | *mad1l1* | *MAD1L1* | 0.382 | 2.45E-02 |
| NM_001020489 | Dr.133578 | *zgc:110591* | *TMEM179B* | 0.378 | 3.37E-02 |
| NM_131587 | Dr.77675 | *gabpa* | *GABPA* | 0.377 | 1.56E-02 |
| NM_001013309 | Dr.77002 | *htatip* | *HTATIP* | 0.377 | 3.43E-02 |
| NM_201150 | Dr.75611 | *rhoae* | *RHOA* | 0.377 | 3.03E-03 |
| NM_001113616 | Dr.133035 | *zgc:172049* | *N.A.* | 0.376 | 2.26E-02 |
| NM_200173 | Dr.82394 | *ccdc53* | *CCDC53* | 0.376 | 3.22E-02 |
| NM_001278600 | N.A. | *N.A.* | *TIMELESS* | 0.376 | 1.73E-02 |
| NM_200759 | Dr.82407 | *eif4e2rs1* | *EIF4E2* | 0.373 | 3.35E-03 |
| NM_001128523 | Dr.40806 | *pole* | *POLE* | 0.373 | 3.98E-02 |
| NM_001020547 | Dr.84063 | *zgc:109995* | *ABT1* | 0.372 | 3.82E-02 |
| NM_207182 | Dr.134075 | *itsn1* | *ITSN1* | 0.372 | 4.25E-02 |
| NM_001098189 | Dr.39353 | *zgc:162584* | *VPS16* | 0.371 | 2.50E-02 |
| NM_212886 | Dr.87100 | *pgm2* | *PGM2* | 0.370 | 1.63E-02 |
| NM_001127520 | Dr.86802 | *si:dkeyp-69e1.8* | *N.A.* | 0.368 | 2.36E-02 |
| NM_201465 | Dr.105519 | *calrl* | *CALRL* | 0.366 | 1.83E-02 |
| NM_001045403 | Dr.41413 | *ccdc126* | *CCDC126* | 0.364 | 3.72E-02 |
| NM_200926 | Dr.5152 | *ercc2* | *ERCC2* | 0.363 | 3.20E-02 |
| NM_001003570 | Dr.3619 | *mfsd2ab* | *MFSD2A* | 0.362 | 7.79E-03 |
| NM_212657 | Dr.78440 | *gtf2a1* | *GTF2A1* | 0.361 | 1.70E-02 |
| NM_200473 | Dr.78529 | *ube3c* | *UBE3C* | 0.359 | 2.22E-02 |
| NM_001079994 | Dr.76563 | *gfm1* | *GFM1* | 0.358 | 6.56E-03 |
| NM_212989 | Dr.77291 | *rangap1* | *CHADL* | 0.357 | 2.40E-02 |
| NM_001118891 | Dr.27086 | *s1pr4* | *S1PR4* | 0.356 | 4.64E-02 |
| NM_001110486 | Dr.133614 | *zgc:158292* | *FILIP1L* | 0.355 | 3.88E-02 |
| NM_001013308 | Dr.81793 | *ndufaf1* | *NDUFAF1* | 0.354 | 2.04E-02 |
| NM_001017400 | Dr.79761 | *tmem11* | *TMEM11* | 0.352 | 1.46E-02 |
| NM_201133 | Dr.26559 | *stk10* | *STK10* | 0.352 | 7.54E-03 |
| NM_001012259 | Dr.85427 | *xkr9* | *XKR9* | 0.351 | 1.12E-02 |
| NM_001077355 | Dr.76050 | *atp5a1* | *ATP5A1* | 0.350 | 1.60E-03 |
| NM_001077732 | Dr.30677 | *agpat2* | *AGPAT1* | 0.349 | 2.77E-02 |
| NM_001002715 | Dr.19030 | *zgc:92360* | *ADAP1* | 0.348 | 3.51E-02 |
| NM_001089539 | Dr.82547 | *hps5* | *HPS5* | 0.348 | 2.96E-02 |
| NM_200747 | Dr.76085 | *ndufv2* | *NDUFV2* | 0.348 | 1.78E-02 |
| NM_001076774 | Dr.43779 | *zgc:153739* | *ABI1* | 0.347 | 5.49E-03 |
| NM_001020466 | Dr.4121 | *nup88* | *NUP88* | 0.343 | 1.12E-02 |
| NM_205724 | Dr.84666 | *fbxo25* | *FBXO25* | 0.343 | 1.58E-02 |
| NM_001130610 | Dr.81201 | *rbl1* | *RBL1* | 0.341 | 3.80E-02 |
| NM_001044783 | Dr.12501 | *xab2* | *XAB2* | 0.337 | 2.74E-02 |
| NM_131246 | Dr.4212 | *ldha* | *LDHB* | 0.335 | 3.11E-04 |
| NM_214692 | Dr.111730 | *mpp1* | *MPP1* | 0.334 | 1.41E-02 |
| NM_001007323 | Dr.104263 | *pgam5* | *PGAM5* | 0.333 | 1.92E-02 |
| NM_199772 | Dr.113807 | *dcun1d1* | *DCUN1D1* | 0.332 | 4.01E-02 |
| NM_001030074 | Dr.407 | *kcnk6* | *KCNK6* | 0.332 | 1.66E-02 |
| NM_201491 | Dr.203 | *ivd* | *IVD* | 0.329 | 1.14E-02 |
| NM_001077274 | Dr.74454 | *zgc:153129* | *CSTB* | 0.329 | 7.94E-03 |
| NM_200196 | Dr.745 | *pdlim5b* | *PDLIM5* | 0.328 | 2.52E-02 |
| NM_213301 | Dr.76704 | *aldh2b* | *ALDH2B* | 0.327 | 4.09E-02 |
| NM_199560 | Dr.150282 | *nt5c3* | *NT5C3A* | 0.327 | 3.40E-02 |
| NM_200426 | Dr.81560 | *ctssa* | *CTSS* | 0.325 | 4.76E-02 |
| NM_001109704 | Dr.148836 | *trim35-28* | *TRIM35* | 0.325 | 2.99E-02 |
| NM_001020621 | Dr.75443 | *cpsf3l* | *CPSF3L* | 0.324 | 3.82E-02 |
| NM_131733 | Dr.5294 | *eif4ea* | *EIF4E1B* | -0.325 | 3.66E-02 |
| NM_001003540 | Dr.30089 | *otud5b* | *OTUD5* | -0.327 | 4.80E-02 |
| NM_213374 | Dr.31694 | *zgc:56304* | *G3BP2* | -0.329 | 4.20E-02 |
| NM_001076656 | Dr.88985 | *zgc:153723* | *Sult5a1* | -0.330 | 2.35E-02 |
| NM_213530 | Dr.79349 | *ube2g1b* | *UBE2G1* | -0.330 | 9.97E-03 |
| NM_200258 | Dr.78389 | *zgc:55558* | *N.A.* | -0.330 | 3.24E-02 |
| NM_212633 | Dr.132358 | *appbp2* | *APPBP2* | -0.331 | 4.06E-02 |
| NM_213333 | Dr.76719 | *sltm* | *SLTM* | -0.334 | 3.83E-03 |
| NM_001004120 | Dr.2043 | *nfil3* | *ECHDC2* | -0.335 | 3.96E-03 |
| NM_200034 | Dr.76296 | *zgc:56419* | *N.A.* | -0.335 | 1.82E-02 |
| NM_200817 | Dr.85766 | *znf593* | *ZNF593* | -0.337 | 3.68E-02 |
| NM_001025502 | Dr.75936 | *h2afy2* | *H2AFY2* | -0.337 | 2.93E-02 |
| NM_001030161 | Dr.11432 | *gigfy2* | *GIGYF2* | -0.337 | 4.71E-02 |
| NM_214712 | Dr.75447 | *aip* | *AIP* | -0.339 | 2.11E-02 |
| NM_001002300 | Dr.4758 | *si:ch211-114c12.2* | *N.A.* | -0.339 | 4.62E-03 |
| NM_001080030 | Dr.161769 | *si:ch211-216l23.1* | *ZNF423* | -0.339 | 4.96E-02 |
| NM_199809 | Dr.32972 | *hsd3b7* | *HSD3B7* | -0.341 | 8.95E-03 |
| NM_001001844 | Dr.53867 | *ctcf* | *CTCF* | -0.341 | 1.11E-02 |
| NM_001030203 | Dr.77560 | *nde1* | *NDE1* | -0.342 | 1.40E-02 |
| NM_199563 | Dr.2393 | *dohh* | *DOHH* | -0.342 | 3.45E-02 |
| NM_201313 | Dr.114177 | *dnaja3a* | *DNAJA3* | -0.343 | 4.69E-02 |
| NM_001017907 | Dr.43219 | *atpaf1* | *ATPAF1* | -0.343 | 3.03E-02 |
| NM_200560 | Dr.108101 | *pdhx* | *PDHX* | -0.343 | 4.23E-02 |
| NM_001013349 | Dr.39143 | *zgc:112982* | *N.A.* | -0.344 | 2.88E-02 |
| NM_131002 | Dr.75473 | *lmnb2* | *LMNB2* | -0.347 | 4.90E-02 |
| NM_199630 | Dr.77415 | *stx5al* | *STX5* | -0.347 | 2.99E-02 |
| NM_198878 | Dr.19492 | *rbpja* | *RBPJ* | -0.349 | 4.15E-02 |
| NM_001012304 | Dr.38353 | *rbm39a* | *RBM39* | -0.350 | 9.02E-03 |
| NM_001199871 | Dr.35651 | *usp3* | *USP3* | -0.350 | 4.84E-02 |
| NM_212777 | Dr.80311 | *brd8* | *BRD8* | -0.350 | 3.62E-02 |
| NM_199691 | Dr.75956 | *cul3a* | *CUL3* | -0.350 | 4.74E-03 |
| NM_001089574 | Dr.74865 | *zgc:162952* | *N.A.* | -0.352 | 1.25E-02 |
| NM_001002333 | Dr.31674 | *stx5a* | *STX5* | -0.353 | 1.95E-02 |
| NM_199692 | Dr.1709 | *rhogc* | *RHOGC* | -0.353 | 1.95E-02 |
| NM_001017651 | Dr.85481 | *gosr1* | *GOSR1* | -0.354 | 2.34E-02 |
| NM_001002517 | Dr.75251 | *arl6ip5b* | *ARL6IP5* | -0.354 | 2.04E-02 |
| NM_201003 | Dr.82352 | *ubtfl* | *UBTF* | -0.357 | 9.02E-03 |
| NM_001025538 | Dr.40248 | *tspan3a* | *TSPAN3A* | -0.359 | 3.23E-03 |
| NM_201325 | Dr.24562 | *mvp* | *MVP* | -0.360 | 2.25E-03 |
| NM_001077370 | Dr.75360 | *prkar2aa* | *PRKAR2B* | -0.362 | 2.23E-02 |
| NM_001199755 | Dr.76543 | *raf1b* | *RAF1* | -0.365 | 3.94E-02 |
| NM_200100 | Dr.76367 | *hig1* | *HIGD1A* | -0.368 | 2.79E-02 |
| NM_212766 | Dr.21188 | *ranbp1* | *RANBP1* | -0.369 | 1.59E-03 |
| NM_001006076 | Dr.17359 | *tbc1d16* | *TBC1D16* | -0.370 | 3.10E-02 |
| NM_200497 | Dr.82480 | *gmppab* | *GMPPA* | -0.372 | 2.08E-02 |
| NM_001034176 | Dr.116299 | *zgc:109953* | *KANSL3* | -0.372 | 3.96E-02 |
| NM_001007392 | Dr.76132 | *pgrmc1* | *PGRMC1* | -0.374 | 1.10E-02 |
| NM_001099239 | Dr.75673 | *nucks1b* | *NUCKS1B* | -0.375 | 6.08E-03 |
| NM_199740 | Dr.80033 | *ccnl1b* | *CCNL2* | -0.375 | 3.56E-02 |
| NM_131374 | Dr.76266 | *psme2* | *PSME2* | -0.377 | 1.07E-02 |
| NM_001089415 | Dr.80634 | *si:ch211-155m12.3* | *SI:CH211-155M12.3* | -0.378 | 4.55E-02 |
| NM_200409 | Dr.82789 | *fibpa* | *FIBP* | -0.378 | 3.09E-02 |
| NM_199982 | Dr.5866 | *kidins220b* | *KIDINS220* | -0.379 | 1.09E-03 |
| NM_199649 | Dr.78307 | *zfp36l1b* | *ZFP36L1* | -0.380 | 2.94E-02 |
| NM_001110127 | Dr.80564 | *rbm38* | *RBM38* | -0.382 | 1.13E-02 |
| NM_001077247 | Dr.78060 | *si:dkey-180p18.9* | *AKR1B1* | -0.385 | 2.77E-02 |
| NM_200879 | Dr.79763 | *slc30a1a* | *SLC30A1* | -0.386 | 3.05E-03 |
| NM_001002211 | Dr.28994 | *ppapdc1b* | *PPAPDC1A* | -0.386 | 1.20E-02 |
| NM_213099 | Dr.81874 | *wdr5* | *N.A.* | -0.387 | 2.93E-02 |
| NM_001044932 | Dr.15113 | *ints3* | *INTS3* | -0.388 | 2.07E-02 |
| NM_213451 | Dr.8006 | *eif4a2* | *EIF4A1* | -0.390 | 2.72E-02 |
| NM_001130592 | Dr.107078 | *nr1d2a* | *NR1D2* | -0.390 | 3.81E-02 |
| NM_199544 | Dr.5116 | *ctdsp2* | *CTDSPL* | -0.391 | 2.26E-02 |
| NM_130941 | Dr.75779 | *pl10* | *D1Pas1* | -0.393 | 3.93E-02 |
| NM_214707 | Dr.132844 | *zdhhc18b* | *ZDHHC18* | -0.394 | 2.22E-02 |
| NM_001172683 | Dr.41669 | *zgc:113045* | *TCF3* | -0.394 | 2.25E-02 |
| NM_001082551 | Dr.78031 | *whsc1* | *WHSC1* | -0.396 | 1.85E-02 |
| NM_001020542 | Dr.13598 | *ints2* | *INTS2* | -0.396 | 4.48E-02 |
| NM_001013266 | Dr.105401 | *strn3* | *STRN3* | -0.398 | 5.52E-03 |
| NM_182967 | Dr.7638 | *calm3a* | *CALM3A* | -0.398 | 8.98E-03 |
| NM_001256603 | Dr.31547 | *asph* | *ASPH* | -0.399 | 5.33E-03 |
| NM_131145 | Dr.233 | *nras* | *NRAS* | -0.399 | 1.05E-02 |
| NM_001077389 | Dr.20000 | *tfam* | *TFAM* | -0.400 | 1.99E-02 |
| NM_001002365 | Dr.75490 | *zgc:92510* | *N.A.* | -0.402 | 1.75E-02 |
| NM_001020515 | Dr.82862 | *dpysl5b* | *DPYSL5* | -0.403 | 3.61E-02 |
| NM_200534 | Dr.24325 | *wdr77* | *WDR77* | -0.405 | 1.15E-02 |
| NM_200938 | Dr.78086 | *b4galt6* | *B4GALT5* | -0.407 | 2.75E-02 |
| NM_131266 | Dr.81307 | *cmyb* | *MYB* | -0.407 | 3.64E-02 |
| NM_201121 | Dr.3090 | *sbds* | *SBDS* | -0.408 | 6.26E-03 |
| NM_001040393 | Dr.17624 | *kalrnb* | *KALRN* | -0.408 | 3.86E-02 |
| NM_001098748 | Dr.83835 | *pacs1* | *PACS1* | -0.411 | 4.46E-02 |
| NM_001100138 | Dr.41243 | *rbm5* | *RBM5* | -0.412 | 1.14E-02 |
| NM_199590 | Dr.76997 | *rpl7l1* | *RPL7L1* | -0.414 | 2.79E-02 |
| NM_001002500 | Dr.32713 | *otub1b* | *OTUB1* | -0.414 | 2.83E-02 |
| NM_001102570 | Dr.103206 | *pum2* | *PUM1* | -0.414 | 4.53E-02 |
| NM_212715 | Dr.76581 | *exoc3* | *EXOC3* | -0.415 | 3.60E-03 |
| NM_001020481 | Dr.17901 | *gpr56* | *GPR56* | -0.415 | 6.01E-03 |
| NM_001007452 | Dr.106151 | *zgc:101614* | *GNB4* | -0.416 | 1.32E-02 |
| NM_194380 | Dr.16301 | *dusp6* | *DUSP6* | -0.418 | 2.83E-02 |
| NM_001079683 | Dr.38452 | *zgc:153683* | *ARMC8* | -0.418 | 2.85E-02 |
| NM_001083830 | Dr.84913 | *zgc:198241* | *N.A.* | -0.419 | 3.86E-02 |
| NM_001017851 | Dr.76437 | *eif4e1c* | *EIF4E* | -0.419 | 1.70E-02 |
| NM_131763 | Dr.76013 | *cldnb* | *CLDN4* | -0.420 | 1.76E-02 |
| NM_201343 | Dr.3270 | *arpc1a* | *ARPC1B* | -0.421 | 9.09E-04 |
| NM_199944 | Dr.82158 | *lmbrd2b* | *LMBRD2* | -0.423 | 1.01E-02 |
| NM_201336 | Dr.76326 | *pfn2* | *PFN2* | -0.428 | 7.86E-04 |
| NM_001161333 | Dr.88405 | *pparaa* | *PPARAA* | -0.428 | 2.51E-02 |
| NM_001006090 | Dr.683 | *fam73a* | *FAM73A* | -0.429 | 3.80E-02 |
| NM_001256179 | Dr.86533 | *mbip* | *MBIP* | -0.431 | 3.39E-02 |
| NM_001007375 | Dr.32436 | *si:dkey-42i9.4* | *N.A.* | -0.432 | 3.45E-02 |
| NM_001089363 | Dr.133317 | *ccnl1a* | *CCNL1* | -0.432 | 8.77E-03 |
| NM_214752 | Dr.35325 | *irf2bp2b* | *IRF2BP2* | -0.433 | 2.41E-02 |
| NM_213058 | Dr.67791 | *hspa5* | *HSPA5* | -0.434 | 1.26E-03 |
| NM_199617 | Dr.75369 | *dus1l* | *DUS1L* | -0.434 | 2.69E-02 |
| NM_001122708 | Dr.1042 | *hirip5* | *NFU1* | -0.434 | 4.15E-02 |
| NM_001202447 | Dr.104540 | *phf8* | *PHF8* | -0.434 | 3.79E-02 |
| NM_001037386 | Dr.76971 | *ppp1r7* | *PPP1R7* | -0.435 | 4.46E-03 |
| NM_001008585 | Dr.121016 | *slc43a2b* | *SLC43A2* | -0.438 | 3.83E-02 |
| NM_200903 | Dr.88365 | *derl3* | *DERL2* | -0.439 | 1.61E-02 |
| NM_001089497 | Dr.81126 | *acadsb* | *ACADSB* | -0.440 | 1.86E-02 |
| NM_199769 | Dr.76520 | *st13* | *ST13* | -0.442 | 1.07E-02 |
| NM_201495 | Dr.7872 | *rab14* | *RAB14* | -0.443 | 1.08E-02 |
| NM_199888 | Dr.24762 | *zc3h15* | *ZC3H15* | -0.445 | 6.73E-03 |
| NM_212696 | Dr.75185 | *dpf2l* | *DPF2* | -0.445 | 1.76E-02 |
| NM_001044809 | Dr.77149 | *smek1* | *SMEK2* | -0.446 | 1.71E-03 |
| NM_001080170 | Dr.4142 | *zgc:158452* | *TP53BP1* | -0.446 | 2.98E-02 |
| NM_001199725 | Dr.51349 | *hp1bp3* | *HP1BP3* | -0.447 | 2.06E-04 |
| NM_001045064 | Dr.78600 | *hcfc1a* | *HCFC1* | -0.448 | 1.01E-02 |
| NM_214816 | Dr.77355 | *tcf12* | *TCF12* | -0.448 | 4.31E-02 |
| NM_001045071 | Dr.73935 | *serpina10a* | *SERPINA10* | -0.449 | 2.25E-02 |
| NM_200645 | Dr.118851 | *pex13* | *PEX13* | -0.451 | 2.55E-02 |
| NM_001045300 | Dr.88991 | *lgalsla* | *LGALSL* | -0.452 | 3.31E-02 |
| NM_200157 | Dr.80685 | *fam108c1* | *FAM108C1* | -0.453 | 2.43E-02 |
| NM_201326 | Dr.76128 | *hspa9* | *HSPA9* | -0.454 | 4.05E-02 |
| NM_001159971 | Dr.70286 | *spty2d1* | *SPTY2D1* | -0.456 | 2.30E-02 |
| NM_200925 | Dr.75888 | *stau2* | *STAU2* | -0.457 | 5.36E-04 |
| NM_001044953 | Dr.75278 | *si:dkey-149j18.2* | *QPCTL* | -0.457 | 3.50E-02 |
| NM_001003755 | Dr.82163 | *immp2l* | *IMMP2L* | -0.457 | 3.82E-02 |
| NM_001020711 | Dr.140739 | *nr3c1* | *NR3C1* | -0.458 | 1.99E-02 |
| NM_200448 | Dr.80389 | *zgc:63470* | *C11orf68* | -0.460 | 4.55E-02 |
| NM_131855 | Dr.2532 | *prkci* | *PRKCI* | -0.463 | 1.96E-03 |
| NM_200054 | Dr.11469 | *setd3* | *SETD3* | -0.464 | 6.29E-04 |
| NM_001098265 | Dr.78930 | *adnp2a* | *ADNP2* | -0.464 | 6.12E-03 |
| NM_001030212 | Dr.77356 | *znf292b* | *ZNF292B* | -0.465 | 1.56E-02 |
| NM_001076648 | Dr.157101 | *smcr7b* | *SMCR7* | -0.465 | 3.45E-02 |
| NM_212745 | Dr.79191 | *tsc22d1* | *TSC22D1* | -0.465 | 3.95E-02 |
| NM_201340 | Dr.80462 | *ahcyl2* | *AHCYL2* | -0.466 | 3.12E-03 |
| NM_001045387 | Dr.16561 | *arl9* | *ARL9* | -0.466 | 2.57E-02 |
| NM_001017853 | Dr.27897 | *cbx7a* | *CBX7A* | -0.467 | 7.31E-03 |
| NM_001002297 | Dr.76519 | *gnl3* | *GNL3* | -0.468 | 7.11E-03 |
| NM_200087 | Dr.18396 | *txnipa* | *TXNIP* | -0.469 | 1.45E-02 |
| NM_213240 | Dr.10567 | *dusp16* | *DUSP16* | -0.469 | 1.84E-02 |
| NM_001007354 | Dr.85073 | *eif4ebp3* | *EIF4EBP3* | -0.472 | 3.98E-03 |
| NM_131664 | Dr.78549 | *lima1* | *LIMA1* | -0.473 | 3.96E-02 |
| NM_001002493 | Dr.10032 | *jdp2* | *JDP2* | -0.473 | 3.41E-02 |
| NM_213536 | Dr.32781 | *zfp161* | *ZFP161* | -0.473 | 2.89E-02 |
| NM_200842 | Dr.133657 | *zgc:65890* | *QKI* | -0.476 | 2.27E-02 |
| NM_198363 | Dr.79310 | *tob1a* | *TOB1* | -0.477 | 5.56E-05 |
| NM_001281461 |  |  | *BPHL* | -0.477 | 3.12E-02 |
| NM_001076705 | Dr.143230 | *zgc:153059* | *CCDC84* | -0.479 | 1.55E-02 |
| NM_212901 | Dr.30389 | *zer1* | *ZER1* | -0.479 | 2.90E-02 |
| NM_001170739 | Dr.38950 | *mga* | *MGA* | -0.479 | 2.64E-02 |
| NM_001007054 | Dr.78674 | *zgc:92590* | *N.A.* | -0.479 | 3.37E-02 |
| NM_200931 | Dr.82452 | *kdelc1* | *KDELC1* | -0.480 | 3.27E-02 |
| NM_001005951 | Dr.76900 | *dtwd1* | *DTWD1* | -0.480 | 1.96E-02 |
| NM_200517 | Dr.83931 | *zfand2a* | *ZFAND2B* | -0.482 | 3.51E-02 |
| NM_001029966 | Dr.5347 | *trnau1apl* | *TRNAU1AP* | -0.482 | 9.40E-03 |
| NM_200049 | Dr.1220 | *zdhhc4* | *ZDHHC4* | -0.482 | 9.15E-03 |
| NM_001004667 | Dr.28599 | *f11r* | *F11R* | -0.482 | 1.76E-02 |
| NM_001076743 | Dr.76073 | *zgc:153924* | *N.A.* | -0.483 | 2.21E-02 |
| NM_199271 | Dr.77201 | *cpa5* | *CPA1* | -0.484 | 8.19E-04 |
| NM_001077387 | Dr.81672 | *alkbh5* | *ALKBH5* | -0.484 | 1.76E-02 |
| NM_001080074 | Dr.79635 | *zgc:158316* | *FAM126B* | -0.485 | 3.05E-02 |
| NM_205613 | Dr.105647 | *hn1b* | *HN1* | -0.485 | 7.78E-03 |
| NM_001037569 | Dr.85108 | *zgc:123248* | *INPP5A* | -0.486 | 2.08E-03 |
| NM_001144800 | Dr.83567 | *itsn2a* | *ITSN2* | -0.486 | 3.86E-02 |
| NM_001110279 | Dr.87819 | *mll* | *MLL* | -0.488 | 9.33E-04 |
| NM_131163 | Dr.51646 | *b2m* | *B2M* | -0.489 | 4.81E-02 |
| NM_001245966 | Dr.157282 | *zeb2b* | *ZEB2B* | -0.490 | 3.66E-02 |
| NR_046387 | Dr.144326 | *si:dkey-71p21.9* | *N.A.* | -0.490 | 1.81E-02 |
| NM_001002596 | Dr.78678 | *zgc:92313* | *PRSS8* | -0.491 | 3.46E-02 |
| NM_001080029 | Dr.67666 | *arrdc3a* | *ARRDC3* | -0.492 | 1.97E-03 |
| NM_001002692 | Dr.486 | *ppp1r14bb* | *PPP1R14B* | -0.492 | 4.46E-02 |
| NM_001044918 | Dr.62863 | *znf704* | *ZNF704* | -0.493 | 1.27E-02 |
| NM_178288 | Dr.24956 | *msrb1a* | *MSRB1* | -0.493 | 4.56E-02 |
| NM_001017891 | Dr.17389 | *zgc:113019* | *SKA3* | -0.493 | 3.45E-02 |
| NM_001039973 | Dr.83565 | *inpp4aa* | *INPP4AA* | -0.494 | 4.76E-02 |
| NM_001076644 | Dr.134198 | *trim23* | *TRIM23* | -0.494 | 2.70E-02 |
| NM_200244 | Dr.82961 | *plekhf2* | *PLEKHF2* | -0.494 | 4.26E-02 |
| NM_001077257 | Dr.85892 | *foxo1a* | *FOXO1* | -0.497 | 4.96E-02 |
| NM_201128 | Dr.79185 | *sardh* | *SARDH* | -0.497 | 4.66E-03 |
| NM_001128334 | Dr.94251 | *si:dkey-119f1.1* | *SMC6* | -0.500 | 4.79E-02 |
| NM_001126383 | Dr.81479 | *si:ch211-235e18.3* | *SIK1* | -0.501 | 3.04E-02 |
| NM_001079980 | Dr.54339 | *cd99* | *CD99* | -0.502 | 1.63E-02 |
| NM_001172635 | Dr.78284 | *zgc:109987* | *ATP6V0E2* | -0.503 | 4.03E-02 |
| NM_131613 | Dr.75890 | *celf1* | *CELF1* | -0.503 | 2.58E-02 |
| NM_001080606 | Dr.150135 | *zgc:158667* | *PANK2* | -0.503 | 1.13E-02 |
| NM_212988 | Dr.18414 | *pdcd4a* | *BBIP1* | -0.504 | 1.85E-03 |
| NM_001098256 | Dr.80699 | *slc9a6a* | *SLC9A6* | -0.505 | 1.81E-02 |
| NM_001077283 | Dr.86618 | *dclre1b* | *DCLRE1B* | -0.506 | 1.42E-02 |
| NM_213450 | Dr.77852 | *ssbp3b* | *SSBP3B* | -0.507 | 1.64E-03 |
| NM_001040406 | Dr.139146 | *numb* | *NUMB* | -0.507 | 3.98E-02 |
| NM_001113477 | Dr.162180 | *keap1b* | *KEAP1* | -0.508 | 5.66E-03 |
| NM_212645 | Dr.75559 | *hnrnpa0a* | *HNRNPA0* | -0.509 | 1.00E-02 |
| NM_001004114 | Dr.2818 | *phf20b* | *PHF20* | -0.509 | 7.12E-04 |
| NM_001039921 | Dr.160831 | *pcmtd2* | *PCMTD2* | -0.509 | 2.94E-02 |
| NM_201330 | Dr.83736 | *dhcr7* | *DHCR7* | -0.509 | 2.88E-02 |
| NM_001037413 | Dr.86098 | *zgc:123272* | *DEM1* | -0.509 | 2.50E-02 |
| NM_001099419 | Dr.111213 | *zgc:165507* | *SLC16A7* | -0.509 | 4.34E-02 |
| NM_199852 | Dr.80603 | *taf5l* | *TAF5L* | -0.510 | 3.56E-02 |
| NM_201463 | Dr.75454 | *ppan* | *PPAN* | -0.510 | 1.11E-02 |
| NM_001045115 | Dr.43918 | *znf395b* | *ZNF395* | -0.511 | 1.60E-03 |
| NM_001012388 | Dr.37767 | *dlg1l* | *DLG1* | -0.514 | 3.82E-02 |
| NM_194400 | Dr.19467 | *hes6* | *HES6* | -0.515 | 2.83E-02 |
| NM_001089492 | Dr.79383 | *ncapg2* | *NCAPG2* | -0.515 | 4.23E-02 |
| NM_001115142 | Dr.13851 | *snrnp48* | *SNRNP48* | -0.517 | 2.34E-02 |
| NM_200717 | Dr.84829 | *thrap6* | *MED30* | -0.519 | 2.17E-03 |
| NM_001128729 | Dr.79877 | *klf9* | *KLF9* | -0.519 | 3.56E-03 |
| NM_199894 | Dr.9168 | *ankmy2a* | *ANKMY2* | -0.519 | 3.07E-02 |
| NM_001115057 | Dr.77211 | *zgc:174906* | *N.A.* | -0.520 | 2.22E-02 |
| NM_001202418 | Dr.75927 | *phf2* | *PHF2* | -0.520 | 1.73E-04 |
| NM_001082827 | Dr.85149 | *march5* | *MARCH5* | -0.520 | 3.49E-02 |
| NM_199623 | Dr.24816 | *dnajc5ga* | *DNAJC5GA* | -0.521 | 1.55E-02 |
| NM_001003769 | Dr.79460 | *cep57l1* | *CEP57L1* | -0.523 | 3.69E-02 |
| NM_001077319 | Dr.28311 | *zgc:152997* | *N.A.* | -0.524 | 1.00E-02 |
| NM_001004010 | Dr.86963 | *calcrla* | *CALCRL* | -0.525 | 4.35E-02 |
| NM_131671 | Dr.132414 | *atp1b1b* | *ATP1B1* | -0.525 | 4.71E-02 |
| NM_001080684 | Dr.90467 | *vhl* | *VHL* | -0.527 | 4.45E-02 |
| NM_001017618 | Dr.85453 | *icmt* | *ICMT* | -0.529 | 7.97E-03 |
| NM_001005583 | Dr.36960 | *zgc:91861* | *PTP4A2* | -0.529 | 9.55E-04 |
| NM_199764 | Dr.13180 | *uck2b* | *UCK2* | -0.529 | 6.20E-03 |
| NM_001113612 | Dr.78167 | *si:dkeyp-104h9.5* | *KIAA2013* | -0.533 | 2.99E-02 |
| NM_001001836 | Dr.4192 | *vipar* | *C14orf133* | -0.534 | 1.97E-02 |
| NM_199932 | Dr.75193 | *fam76b* | *FAM76B* | -0.534 | 2.73E-03 |
| NM_001080067 | Dr.39448 | *rai12* | *C17orf81* | -0.534 | 2.36E-02 |
| NM_001001942 | Dr.75554 | *sparc* | *SPARC* | -0.534 | 2.38E-02 |
| NM_001114923 | Dr.144792 | *arid1aa* | *ARID1AA* | -0.536 | 4.94E-02 |
| NM_001077299 | Dr.78156 | *tusc2a* | *TUSC2A* | -0.537 | 2.54E-02 |
| NM_001013485 | Dr.79137 | *zgc:110782* | *N.A.* | -0.538 | 4.32E-02 |
| NM_001012386 | Dr.83988 | *gadd45bb* | *GADD45B* | -0.539 | 3.70E-02 |
| NM_199662 | Dr.78242 | *dnaja1l* | *DNAJA1* | -0.540 | 9.97E-03 |
| NM_001020567 | Dr.66829 | *zgc:110158* | *SSBP2* | -0.540 | 1.77E-02 |
| NM_200167 | Dr.120616 | *cpne3* | *CPNE3* | -0.542 | 7.18E-03 |
| NM_131873 | Dr.78757 | *her9* | *HER9* | -0.543 | 2.73E-03 |
| NM_199998 | Dr.76325 | *utp11l* | *UTP11L* | -0.544 | 2.62E-02 |
| NM_212678 | Dr.78655 | *chsy1* | *CHSY1* | -0.544 | 1.20E-03 |
| NM_001002151 | Dr.79918 | *calua* | *CALU* | -0.545 | 3.43E-02 |
| NM_001044839 | Dr.77222 | *si:dkey-14d8.6* | *N.A.* | -0.546 | 2.71E-02 |
| NM_200418 | Dr.11398 | *fam172a* | *FAM172A* | -0.547 | 1.96E-03 |
| NM_001110396 | Dr.118484 | *snx10b* | *SNX10B* | -0.548 | 4.80E-02 |
| NM_001166015 | Dr.79363 | *si:dkeyp-106c5.3* | *FAM105B* | -0.549 | 1.45E-02 |
| NM_001002632 | Dr.84417 | *prkab1a* | *PRKAB1* | -0.550 | 1.64E-03 |
| NM_001012243 | Dr.97232 | *gna13a* | *GNA13* | -0.551 | 8.06E-03 |
| NM_001018150 | Dr.106921 | *zgc:110586* | *TSPAN2* | -0.551 | 1.77E-02 |
| NM_199783 | Dr.132553 | *klhl11* | *KLHL11* | -0.552 | 7.40E-04 |
| NM_001163106 | Dr.135603 | *chl1a* | *CHL1* | -0.553 | 3.92E-02 |
| NM_199530 | Dr.19511 | *sec31a* | *SEC31A* | -0.553 | 3.63E-02 |
| NM_001003775 | Dr.151249 | *lrrc33* | *NRROS* | -0.553 | 2.27E-03 |
| NM_200044 | Dr.883 | *dnajc21* | *DNAJC21* | -0.553 | 1.67E-02 |
| NM_001002573 | Dr.76905 | *zgc:92744* | *SERP1* | -0.554 | 2.98E-04 |
| NM_001099250 | Dr.152233 | *zgc:162913* | *PAPL* | -0.555 | 4.17E-02 |
| NM_001025464 | Dr.82686 | *pdcb* | *PDC* | -0.555 | 4.25E-02 |
| NM_001045381 | Dr.81083 | *pter* | *PTER* | -0.555 | 1.62E-02 |
| NM_001123326 | Dr.82327 | *zgc:153958* | *DALRD3* | -0.555 | 6.13E-03 |
| NM_001089466 | Dr.82297 | *srebf2* | *SREBF2* | -0.556 | 9.74E-04 |
| NM_201066 | Dr.80478 | *cbll1* | *CBLL1* | -0.556 | 2.22E-03 |
| NM_001005987 | Dr.87880 | *fam116aa* | *FAM116A* | -0.556 | 3.91E-02 |
| NM_173274 | Dr.51273 | *dag1* | *DAG1* | -0.556 | 4.29E-05 |
| NM_001080683 | Dr.152043 | *zgc:158647* | *CEP57* | -0.557 | 3.28E-02 |
| NM_001045055 | Dr.81141 | *si:ch211-132b12.7* | *N.A.* | -0.557 | 1.27E-02 |
| NM_001017800 | Dr.43229 | *zgc:110331* | *ISCU* | -0.558 | 3.24E-03 |
| NM_001083558 | Dr.151505 | *zgc:136564* | *C9orf64* | -0.559 | 3.83E-02 |
| NM_200680 | Dr.152115 | *rhoga* | *RHOGA* | -0.560 | 1.11E-02 |
| NM_212602 | Dr.2594 | *ahsa1l* | *AHSA1* | -0.560 | 1.77E-02 |
| NM_001076601 | Dr.85100 | *blvra* | *BLVRA* | -0.562 | 3.89E-02 |
| NM_001105111 | Dr.117428 | *si:ch211-121a2.2* | *SI:CH211-121A2.2* | -0.562 | 4.55E-02 |
| NM_200880 | Dr.81134 | *zgc:77593* | *DUSP8* | -0.562 | 5.21E-06 |
| NM_001123280 | Dr.90305 | *nefm* | *NEFM* | -0.563 | 1.89E-02 |
| NM_001025175 | Dr.152522 | *zgc:114108* | *CHORDC1* | -0.563 | 5.62E-03 |
| NM_213480 | Dr.11467 | *pank1a* | *PANK2* | -0.564 | 4.21E-02 |
| NM_200411 | Dr.26603 | *zgc:64090* | *N.A.* | -0.564 | 4.02E-02 |
| NM_199970 | Dr.9836 | *nme2a* | *NME2* | -0.566 | 4.35E-02 |
| NM_001007403 | Dr.36299 | *zgc:101777* | *TRIP10* | -0.570 | 4.24E-02 |
| NM_131795 | Dr.82172 | *psma6b* | *PSMA6* | -0.570 | 7.31E-03 |
| NM_001111237 | Dr.115703 | *zgc:64136* | *SNX15* | -0.572 | 6.38E-03 |
| NM_001128345 | Dr.84513 | *LOC566600* | *N.A.* | -0.572 | 3.52E-02 |
| NM_001020746 | Dr.31410 | *zgc:110425* | *N.A.* | -0.572 | 6.92E-03 |
| NM_001113505 | Dr.143306 | *il12rb2* | *IL12RB2* | -0.573 | 4.15E-02 |
| NM_001017864 | Dr.16628 | *zgc:110697* | *SPIN1* | -0.573 | 4.10E-02 |
| NM_001115070 | Dr.44056 | *zgc:174574* | *ZNF460* | -0.575 | 3.86E-02 |
| NM_001017850 | Dr.105609 | *stmn1b* | *STMN1* | -0.576 | 9.25E-03 |
| NM_001130662 | Dr.84738 | *trim105* | *TRIM105* | -0.577 | 3.71E-02 |
| NM_001114567 | Dr.148560 | *zgc:172215* | *Ankrd50* | -0.580 | 3.43E-02 |
| NM_001003580 | Dr.76011 | *klhl15* | *KLHL15* | -0.580 | 2.97E-02 |
| NM_001081692 | Dr.39148 | *kdm2aa* | *KDM2B* | -0.582 | 7.75E-06 |
| NM_213328 | Dr.39283 | *tesca* | *TESC* | -0.582 | 1.87E-02 |
| NM_201151 | Dr.118789 | *fbxo30a* | *FBXO30* | -0.584 | 3.60E-02 |
| NM_001004666 | Dr.36480 | *uck1* | *UCK1* | -0.584 | 2.99E-02 |
| NM_200216 | Dr.3155 | *trmt11* | *TRMT11* | -0.586 | 2.79E-02 |
| NM_200829 | Dr.107673 | *camk1gb* | *CAMK1G* | -0.586 | 3.71E-02 |
| NM_200052 | Dr.76398 | *tsc1a* | *TSC1* | -0.588 | 5.70E-03 |
| NM_212713 | Dr.116333 | *ythdf1* | *YTHDF1* | -0.588 | 2.36E-04 |
| NM_182885 | Dr.82621 | *pcdh10b* | *PCDH10* | -0.588 | 3.67E-02 |
| NM_001082851 | Dr.133419 | *pafah1b2* | *PAFAH1B3* | -0.589 | 3.33E-02 |
| NM_001044974 | Dr.119082 | *si:dkey-103i16.2* | *N.A.* | -0.591 | 3.07E-02 |
| NM_205538 | Dr.12744 | *rp42-pen* | *DCUN1D2* | -0.593 | 2.34E-02 |
| NM_001002660 | Dr.32020 | *sesn1* | *SESN1* | -0.595 | 7.79E-03 |
| NM_131573 | Dr.159468 | *dap1b* | *DAP1B* | -0.596 | 2.41E-03 |
| NM_001109869 | Dr.86112 | *kdm8* | *KDM8* | -0.596 | 3.21E-02 |
| NM_001034969 | Dr.79904 | *slc5a8l* | *SLC5A8* | -0.597 | 1.93E-02 |
| NM_001256200 | Dr.43460 | *si:dkey-28b4.7* | *N.A.* | -0.598 | 2.43E-02 |
| NM_001122743 | Dr.157798 | *prdm4* | *PRDM4* | -0.599 | 3.53E-03 |
| NM_131362 | Dr.616 | *six3a* | *SIX3* | -0.599 | 3.19E-02 |
| NM_213362 | Dr.79978 | *epn1* | *EPN1* | -0.599 | 6.13E-03 |
| NM_199822 | Dr.80428 | *ccdc82* | *CCDC82* | -0.604 | 3.53E-03 |
| NM_001044963 | Dr.122659 | *rabl2* | *RABL2A* | -0.606 | 2.58E-02 |
| NM_199969 | Dr.9860 | *mdh1b* | *MDH1* | -0.607 | 2.59E-02 |
| NM_200672 | Dr.84934 | *swap70a* | *SWAP70A* | -0.609 | 2.82E-02 |
| NM_001045473 | Dr.89089 | *bmf2* | *BMF2* | -0.610 | 2.72E-02 |
| NM_001013284 | Dr.79360 | *mif4gdb* | *MIF4GD* | -0.611 | 2.82E-02 |
| NM_131849 | Dr.150302 | *adh5* | *ADH5* | -0.613 | 2.26E-08 |
| NM_131411 | Dr.81299 | *aanat2* | *AANAT2* | -0.613 | 2.56E-02 |
| NM_200707 | Dr.21135 | *rdh8a* | *RDH8* | -0.613 | 2.62E-02 |
| NM_001003419 | Dr.83119 | *rab3aa* | *RAB3A* | -0.615 | 2.66E-02 |
| NM_001099981 | Dr.82936 | *stxbp5a* | *STXBP5A* | -0.616 | 9.18E-05 |
| NM_001013298 | Dr.84038 | *prnprs3* | *PRNPRS3* | -0.617 | 3.72E-04 |
| NM_198870 | Dr.75334 | *aif1l* | *AIF1L* | -0.617 | 1.28E-02 |
| NM_200526 | Dr.80642 | *spryd7b* | *SPRYD7* | -0.617 | 1.87E-02 |
| NM_200871 | Dr.117127 | *pde6c* | *PDE6C* | -0.618 | 2.44E-02 |
| NM_200770 | Dr.27145 | *fscn2b* | *FSCN2* | -0.618 | 1.68E-02 |
| NM_001077332 | Dr.84136 | *ddx28* | *DDX28* | -0.620 | 2.41E-02 |
| NM_001030259 | Dr.81450 | *ankha* | *ANKH* | -0.620 | 2.49E-02 |
| NM_001025531 | Dr.48712 | *fam60a* | *FAM60A* | -0.622 | 1.42E-02 |
| NM_001044867 | Dr.81913 | *atrip* | *ATRIP* | -0.622 | 7.23E-03 |
| NM_001033591 | Dr.78377 | *zgc:112334* | *GDI1* | -0.623 | 2.46E-02 |
| NM_001080992 | Dr.89941 | *si:dkey-231l1.6* | *ANKIB1* | -0.623 | 2.00E-03 |
| NM_131564 | Dr.79604 | *appa* | *APP* | -0.625 | 7.47E-03 |
| NM_131670 | Dr.96696 | *atp1b3b* | *ATP1B3* | -0.625 | 1.87E-02 |
| NM_001013300 | Dr.39092 | *zgc:112980* | *N.A.* | -0.625 | 2.40E-03 |
| NM_001020544 | Dr.41776 | *zgc:109985* | *WBP2* | -0.626 | 1.11E-02 |
| NM_001018120 | Dr.150297 | *cdc42* | *CDC42* | -0.628 | 1.34E-03 |
| NM_001012492 | Dr.22101 | *zgc:113436* | *N.A.* | -0.628 | 7.23E-03 |
| NM_001082936 | Dr.119557 | *cyp2ad6* | *CYP2J2* | -0.629 | 2.47E-02 |
| NM_001031840 | Dr.88842 | *arhgef11* | *ARHGEF11* | -0.629 | 1.72E-02 |
| NM_001100019 | Dr.76681 | *LOC566022* | *FDXACB1* | -0.631 | 2.17E-02 |
| NM_199979 | Dr.28183 | *slc1a2b* | *SLC1A2* | -0.632 | 1.07E-02 |
| NM_001204200 | Dr.86519 | *akap2* | *AKAP2* | -0.633 | 2.29E-02 |
| NM_001128783 | Dr.81589 | *npdc1* | *NPDC1* | -0.633 | 1.80E-02 |
| NM_198978 | Dr.77195 | *pdcd4b* | *BBIP1* | -0.635 | 1.54E-02 |
| NM_001045168 | Dr.81002 | *trim108* | *TRIM108* | -0.635 | 1.97E-02 |
| NM_001003533 | Dr.79182 | *bag3* | *BAG3* | -0.635 | 7.57E-03 |
| NM_001110526 | Dr.8167 | *srf* | *SRF* | -0.636 | 5.76E-03 |
| NM_212967 | Dr.87471 | *fat1* | *FAT1* | -0.637 | 2.16E-02 |
| NM_001109717 | Dr.120001 | *cept1b* | *CEPT1* | -0.637 | 1.85E-02 |
| NM_001127466 | Dr.105797 | *smarcal1* | *SMARCAL1* | -0.637 | 6.20E-03 |
| NM_001044754 | Dr.11520 | *si:ch211-51e12.7* | *N.A.* | -0.640 | 1.82E-03 |
| NM_001007368 | Dr.81177 | *nr2e3* | *NR2E3* | -0.641 | 1.35E-02 |
| NM_200744 | Dr.83514 | *smfn* | *RBM7* | -0.641 | 9.94E-03 |
| NM_001012378 | Dr.130482 | *dlg2* | *DLG2* | -0.641 | 1.62E-02 |
| NM_001037376 | Dr.79607 | *zgc:123304* | *TMEM55B* | -0.643 | 3.74E-05 |
| NM_001144793 | Dr.121252 | *ssna1* | *SSNA1* | -0.644 | 1.86E-02 |
| NM_001006095 | Dr.77715 | *thoc6* | *THOC6* | -0.645 | 5.52E-03 |
| NM_001017804 | Dr.134210 | *adss* | *ADSS* | -0.646 | 1.90E-02 |
| NM_001045847 | Dr.76457 | *nprl2* | *NPRL2* | -0.646 | 8.01E-03 |
| NM_001076772 | Dr.161993 | *klf13* | *KLF13* | -0.648 | 4.32E-03 |
| NM_001045069 | Dr.119094 | *si:ch211-216l23.2* | *N.A.* | -0.650 | 5.57E-05 |
| NM_001003833 | Dr.12108 | *sulf2l* | *SULF2* | -0.650 | 3.70E-03 |
| NM_001007332 | Dr.75169 | *rg9mtd1* | *RG9MTD1* | -0.653 | 3.36E-04 |
| NM_001017742 | Dr.82240 | *dusp22b* | *DUSP22* | -0.654 | 1.36E-02 |
| NM_001104940 | Dr.7295 | *si:ch211-175l6.9* | *UBN2* | -0.654 | 1.05E-03 |
| NM_001002177 | Dr.82849 | *impdh1a* | *IMPDH1P11* | -0.656 | 1.21E-02 |
| NM_001033747 | Dr.83836 | *zgc:109965* | *NCLN* | -0.658 | 1.57E-02 |
| NM_212829 | Dr.78001 | *chst14* | *CHST14* | -0.660 | 1.87E-02 |
| NM_152956 | Dr.133651 | *dachb* | *DACH2* | -0.660 | 1.85E-02 |
| NM_001003764 | Dr.91013 | *dexi* | *DEXI* | -0.661 | 1.83E-02 |
| NM_001109827 | Dr.116338 | *zgc:174931* | *N.A.* | -0.661 | 1.85E-02 |
| NM_213120 | Dr.77513 | *yy1b* | *YY1* | -0.663 | 6.08E-05 |
| NM_001020546 | Dr.89401 | *syt5b* | *SYT5B* | -0.664 | 1.59E-02 |
| NM_213008 | Dr.80808 | *cluap1* | *CLUAP1* | -0.664 | 1.27E-03 |
| NM_199959 | Dr.7206 | *nrsn1* | *NRSN1* | -0.666 | 1.58E-02 |
| NM_001007443 | Dr.84141 | *rhoua* | *RHOU* | -0.666 | 1.47E-02 |
| NM_213337 | Dr.79615 | *tmem59l* | *TMEM59L* | -0.666 | 1.52E-02 |
| NM_001034183 | Dr.78680 | *bag5* | *BAG5* | -0.667 | 5.22E-03 |
| NM_200762 | Dr.82968 | *gchfr* | *GCHFR* | -0.667 | 1.37E-02 |
| NM_001089329 | Dr.77946 | *pou2f1b* | *POU2F1B* | -0.669 | 3.57E-03 |
| NM_001004636 | Dr.78507 | *tmem237b* | *TMEM237* | -0.669 | 1.71E-03 |
| NM_001002347 | Dr.83294 | *acy3.2* | *ACY3* | -0.671 | 7.07E-04 |
| NM_001039804 | Dr.155386 | *inppl1a* | *INPPL1* | -0.672 | 1.22E-02 |
| NM_213419 | Dr.107100 | *map2k1* | *MAP2K1* | -0.672 | 1.35E-02 |
| NM_001002437 | Dr.32120 | *gnb3a* | *GNB3* | -0.673 | 1.66E-02 |
| NM_001040355 | Dr.77410 | *pmp22b* | *PMP22* | -0.673 | 1.37E-02 |
| NM_001098741 | Dr.111079 | *zgc:165464* | *RAB39A* | -0.675 | 1.29E-02 |
| NM_001025553 | Dr.92000 | *gabrr1* | *GABRR1* | -0.675 | 1.64E-02 |
| NM_001020699 | Dr.77705 | *zgc:112407* | *TTC38* | -0.677 | 1.49E-02 |
| NM_131298 | Dr.1467 | *pax9* | *PAX9* | -0.679 | 9.57E-03 |
| NM_001002627 | Dr.84455 | *lrrc57* | *LRRC57* | -0.680 | 1.15E-02 |
| NM_205627 | Dr.76381 | *dstyk* | *DSTYK* | -0.681 | 9.67E-05 |
| NM_001025458 | Dr.46022 | *tpte* | *TPTE* | -0.681 | 1.08E-02 |
| NM_214810 | Dr.106912 | *cpe* | *CPE* | -0.682 | 2.10E-03 |
| NM_001111175 | Dr.29913 | *zgc:171566* | *GGH* | -0.683 | 1.43E-02 |
| NM_001013516 | Dr.36054 | *msi1* | *MSI1* | -0.683 | 6.88E-03 |
| NM_001003866 | Dr.78835 | *gtf3aa* | *GTF3A* | -0.683 | 1.17E-02 |
| NM_205579 | Dr.83361 | *myo3a* | *MYO3A* | -0.683 | 1.33E-02 |
| NM_001077739 | Dr.79993 | *zgc:153215* | *N.A.* | -0.684 | 5.15E-03 |
| NM_001005580 | Dr.36462 | *opcml* | *OPCML* | -0.685 | 1.02E-02 |
| NM_001163443 | Dr.133432 | *LOC797202* | *N.A.* | -0.685 | 1.19E-02 |
| NM_200346 | Dr.18420 | *zgc:63938* | *DIABLO* | -0.686 | 5.87E-03 |
| NM_213634 | Dr.75704 | *ptgdsb* | *PTGDSB* | -0.690 | 1.08E-04 |
| NM_001276280 | N.A. | *N.A.* | *KCNH5* | -0.694 | 1.37E-02 |
| NM_001076662 | Dr.79653 | *sfxn4* | *SFXN4* | -0.694 | 4.34E-03 |
| NM_175082 | Dr.52521 | *smad7* | *SMAD7* | -0.695 | 4.05E-03 |
| NM_001099985 | Dr.104708 | *hipk2* | *HIPK2* | -0.696 | 1.13E-02 |
| NM_173288 | Dr.76897 | *mki67ip* | *MKI67IP* | -0.697 | 2.20E-04 |
| NM_001202512 | Dr.33404 | *ssbp4* | *SSBP4* | -0.697 | 3.07E-05 |
| NM_213402 | Dr.79569 | *grtp1a* | *GRTP1* | -0.698 | 1.30E-02 |
| NM_001044891 | Dr.80225 | *bcl2l13* | *BCL2L13* | -0.699 | 5.85E-03 |
| NM_001128270 | Dr.72351 | *LOC796180* | *N.A.* | -0.701 | 1.09E-02 |
| NM_001080613 | Dr.79041 | *napepld* | *NAPEPLD* | -0.701 | 9.58E-03 |
| NM_001039839 | Dr.89513 | *as3mt* | *AS3MT* | -0.701 | 6.92E-03 |
| NM_001287083 |  |  | *DSC1* | -0.701 | 1.07E-02 |
| NM_178131 | Dr.140308 | *scinla* | *SCINLA* | -0.701 | 4.42E-03 |
| NM_001002325 | Dr.32031 | *rdh12* | *RDH12* | -0.702 | 3.25E-04 |
| NM_001100065 | Dr.105007 | *ddah2* | *DDAH2* | -0.705 | 3.02E-03 |
| NM_201021 | Dr.85919 | *rnf11a* | *RNF11A* | -0.708 | 3.93E-04 |
| NM_199836 | Dr.4451 | *chp2* | *CHP2* | -0.709 | 1.12E-02 |
| NM_001136255 | Dr.114276 | *gch1* | *GCH1* | -0.711 | 2.35E-03 |
| NM_200532 | Dr.75861 | *rasd1* | *RASD1* | -0.712 | 1.01E-02 |
| NM_001204257 | Dr.75385 | *ppp1r13ba* | *PPP1R13B* | -0.713 | 1.05E-02 |
| NM_001083068 | Dr.79113 | *lrp12* | *LRP12* | -0.715 | 3.46E-04 |
| NM_153674 | Dr.91583 | *vangl2* | *VANGL2* | -0.717 | 1.06E-02 |
| NM_001114342 | Dr.76499 | *si:dkeyp-113d7.4* | *KRT17* | -0.718 | 3.01E-03 |
| NM_207099 | Dr.80117 | *zgc:77222* | *IGSF8* | -0.719 | 7.94E-03 |
| NM_001034175 | Dr.117033 | *etnk2* | *ETNK2* | -0.719 | 9.93E-03 |
| NM_001122705 | Dr.83889 | *abl2* | *ABL2* | -0.721 | 2.63E-03 |
| NM_001077300 | Dr.28117 | *cplx4a* | *CPLX4* | -0.722 | 6.13E-03 |
| NM_001003445 | Dr.162032 | *zgc:92533* | *KRT17* | -0.722 | 2.39E-05 |
| NM_001288775 | N.A. | *N.A.* | *N.A.* | -0.723 | 8.64E-03 |
| NM_001045423 | Dr.29209 | *zgc:153116* | *N.A.* | -0.723 | 8.89E-03 |
| NM_198917 | Dr.29102 | *foxi3a* | *FOXI1* | -0.726 | 7.32E-03 |
| NM_194421 | Dr.77382 | *hs6st2* | *HS6ST1* | -0.726 | 5.39E-03 |
| NM_001099249 | Dr.91537 | *zgc:165627* | *CEP112* | -0.727 | 9.66E-03 |
| NM_194410 | Dr.134046 | *vax1* | *VAX1* | -0.728 | 6.78E-03 |
| NM_001044944 | Dr.78487 | *si:ch211-191i18.1* | *N.A.* | -0.729 | 7.21E-06 |
| NM_001076654 | Dr.91561 | *krt1-c5* | *KRT1-C5* | -0.730 | 8.65E-03 |
| NM_131442 | Dr.81277 | *gbp* | *GBP* | -0.731 | 5.91E-06 |
| NM_205691 | Dr.116194 | *gadd45g* | *GADD45G* | -0.734 | 1.61E-03 |
| NM_001003480 | Dr.78136 | *zgc:92380* | *KRT18P55* | -0.735 | 1.97E-03 |
| NM_200454 | Dr.13952 | *ptpn11b* | *PTPN11B* | -0.736 | 1.62E-05 |
| NM_131354 | Dr.75839 | *six7* | *SIX6* | -0.737 | 8.70E-03 |
| NM_183069 | Dr.79580 | *zmynd19* | *ZMYND19* | -0.739 | 2.79E-03 |
| NM_205570 | Dr.82618 | *zgc:73226* | *N.A.* | -0.744 | 7.10E-03 |
| NM_001002323 | Dr.84836 | *nudt2* | *NUDT2* | -0.745 | 7.80E-04 |
| NM_001039926 | Dr.37692 | *hs3st1l2* | *HS3ST1* | -0.747 | 7.17E-03 |
| NM_001137660 | Dr.114423 | *wnt9b* | *WNT9B* | -0.751 | 6.87E-03 |
| NM_200501 | Dr.78126 | *parp3* | *PARP3* | -0.752 | 2.14E-03 |
| NM_001017830 | Dr.108513 | *baiap2l1b* | *BAIAP2L1* | -0.752 | 4.16E-03 |
| NM_001045198 | Dr.81349 | *hook1* | *HOOK1* | -0.754 | 5.69E-03 |
| NM_001003776 | Dr.84694 | *ncaldb* | *NCALD* | -0.756 | 5.04E-08 |
| NM_213031 | Dr.76269 | *gadd45ba* | *GADD45B* | -0.758 | 1.84E-04 |
| NM_001030248 | Dr.90388 | *zgc:114180* | *RCVRN* | -0.770 | 2.39E-03 |
| NM_001004015 | Dr.33994 | *lhx6* | *LHX6* | -0.770 | 4.61E-03 |
| NM_001130612 | Dr.99089 | *atxn10* | *ATXN10* | -0.776 | 5.77E-03 |
| NM_131214 | Dr.251 | *cdh11* | *CDH11* | -0.784 | 3.83E-03 |
| NM_001168265 | Dr.87567 | *dyrk1b* | *DYRK1B* | -0.784 | 2.00E-06 |
| NM_001017876 | Dr.150688 | *fsip1* | *FSIP1* | -0.784 | 4.19E-03 |
| NM_200417 | Dr.87870 | *gpr39* | *LYPD1* | -0.786 | 5.02E-03 |
| NM_001113636 | Dr.116564 | *zgc:172291* | *SNCAIP* | -0.787 | 4.87E-03 |
| NM_001005604 | Dr.79924 | *bcl9* | *BCL9* | -0.788 | 4.49E-03 |
| NM_001003438 | Dr.133096 | *rs1* | *RS1* | -0.794 | 1.79E-03 |
| NM_131333 | Dr.558 | *vsx1* | *VSX1* | -0.795 | 1.90E-03 |
| NM_214776 | Dr.13478 | *spryd7a* | *SPRYD7* | -0.796 | 2.88E-04 |
| NM_001037803 | Dr.52755 | *rln3a* | *RLN3A* | -0.799 | 1.07E-03 |
| NM_001030163 | Dr.79004 | *sfxn5b* | *SFXN5* | -0.799 | 4.52E-03 |
| NM_200828 | Dr.82827 | *gnao1b* | *GNAO1* | -0.800 | 3.20E-03 |
| NM_001122760 | Dr.119387 | *thsd7a* | *THSD7A* | -0.800 | 3.05E-03 |
| NM_001172661 | Dr.129884 | *si:ch211-39k3.2* | *N.A.* | -0.802 | 4.12E-03 |
| NM_213232 | Dr.76703 | *spred1* | *SPRED1* | -0.804 | 8.57E-04 |
| NM_001039983 | Dr.92028 | *ezh1* | *EZH2* | -0.807 | 3.59E-05 |
| NM_001089376 | Dr.65703 | *stxbp1b* | *STXBP1* | -0.808 | 4.00E-03 |
| NM_200757 | Dr.80934 | *scg3* | *SCG3* | -0.808 | 3.92E-03 |
| NM_001003477 | Dr.78344 | *dhrs3a* | *DHRS3* | -0.809 | 3.98E-03 |
| NM_001143933 | Dr.35961 | *si:dkeyp-118h3.6* | *C2orf49* | -0.812 | 3.19E-03 |
| NM_001045333 | Dr.89962 | *kctd7* | *KCTD7* | -0.812 | 1.79E-03 |
| NM_001017650 | Dr.75294 | *si:dkey-22a1.3* | *C3orf17* | -0.813 | 3.07E-03 |
| NM_001111244 | Dr.80441 | *zgc:171298* | *PPFIA1* | -0.815 | 3.50E-03 |
| NM_152955 | Dr.85653 | *dacha* | *DACH2* | -0.821 | 3.43E-03 |
| NM_001201403 | Dr.30088 | *si:ch211-222g5.3* | *Atp6ap1l* | -0.823 | 3.25E-03 |
| NM_001040351 | Dr.105850 | *st14a* | *ST14* | -0.830 | 2.21E-03 |
| NM_001159826 | Dr.11289 | *zgc:112320* | *N.A.* | -0.832 | 2.62E-03 |
| NM_213523 | Dr.76187 | *krt15* | *KRT17* | -0.835 | 3.07E-05 |
| NM_001161750 | Dr.16458 | *map3k5* | *MAP3K5* | -0.835 | 3.23E-04 |
| NM_001281844 | N.A. | *N.A.* | *PIK3R1* | -0.836 | 2.90E-05 |
| NM_001012389 | Dr.134827 | *tcf7* | *TCF7* | -0.839 | 2.24E-03 |
| NM_001009890 | Dr.38006 | *cyp2v1* | *CYP2J2* | -0.839 | 2.64E-03 |
| NM_001110527 | Dr.133462 | *zgc:174310* | *N.A.* | -0.843 | 2.73E-03 |
| NM_131205 | Dr.7714 | *etv5b* | *ETV5* | -0.844 | 2.05E-04 |
| NM_001128526 | Dr.88989 | *phf13* | *PHF19* | -0.845 | 2.59E-03 |
| NM_001005958 | Dr.78154 | *lgals1l1* | *LGALS1* | -0.848 | 2.02E-03 |
| NM_001118898 | Dr.80367 | *qrsl1* | *QRSL1* | -0.850 | 4.71E-04 |
| NM_001245959 | Dr.119265 | *pvrl2l* | *PVRL2* | -0.850 | 1.64E-03 |
| NM_199818 | Dr.117263 | *rhoq* | *RHOQ* | -0.852 | 2.42E-03 |
| NM_001190307 | Dr.159210 | *kcnk18* | *KCNK18* | -0.852 | 2.38E-03 |
| NM_131392 | Dr.7957 | *psmb8* | *PSMB8* | -0.854 | 2.40E-03 |
| NM_001009591 | Dr.37841 | *pcdh2ab12* | *PCDHA8* | -0.857 | 2.28E-03 |
| NM_201208 | Dr.26454 | *grina* | *GRINA* | -0.861 | 2.12E-04 |
| NM_212894 | Dr.162115 | *lgals2a* | *LGALS1* | -0.862 | 8.68E-04 |
| NM_131000 | Dr.20912 | *alcama* | *ALCAM* | -0.867 | 4.01E-04 |
| NM_001163298 | Dr.78761 | *msl2s* | *MSL2* | -0.869 | 3.08E-04 |
| NM_199429 | Dr.11595 | *pigq* | *PIGQ* | -0.879 | 3.49E-04 |
| NM_201306 | Dr.6972 | *lrpap1* | *LRPAP1* | -0.880 | 1.04E-05 |
| NM_199967 | Dr.81940 | *gngt1* | *GNGT1* | -0.889 | 1.50E-03 |
| NM_001007425 | Dr.11259 | *zgc:101716* | *ZHX1* | -0.891 | 1.43E-03 |
| NM_199816 | Dr.11252 | *zgc:56085* | *N.A.* | -0.891 | 1.37E-03 |
| NM_001039816 | Dr.79058 | *specc1la* | *SPECC1L* | -0.893 | 2.72E-04 |
| NM_001039808 | Dr.132485 | *slc2a1a* | *SLC2A3* | -0.902 | 5.41E-04 |
| NM_001110473 | Dr.85351 | *igsf21b* | *IGSF21* | -0.911 | 1.20E-03 |
| NM_001002734 | Dr.133013 | *tspan18a* | *TSPAN18* | -0.912 | 7.59E-04 |
| NM_001013518 | Dr.87101 | *faim2a* | *FAIM2* | -0.914 | 6.75E-04 |
| NM_131840 | Dr.10712 | *tnfrsfa* | *TNFRSFA* | -0.917 | 2.24E-04 |
| NM_001077731 | Dr.81779 | *zgc:154093* | *N.A.* | -0.917 | 9.71E-04 |
| NM_001098249 | Dr.74671 | *si:ch211-149p10.2* | *N.A.* | -0.919 | 2.53E-04 |
| NM_153657 | Dr.113864 | *ptgs2a* | *PTGS2* | -0.921 | 8.81E-06 |
| NM_001014304 | Dr.6690 | *pvrl3l* | *PVRL3* | -0.925 | 1.01E-03 |
| NM_213258 | Dr.6427 | *atat1* | *ATAT1* | -0.933 | 6.79E-04 |
| NM_200039 | Dr.79812 | *sdf2* | *SDF2* | -0.936 | 3.84E-04 |
| NM_001002455 | Dr.120320 | *phlda3* | *PHLDA3* | -0.942 | 6.84E-04 |
| NM_001004603 | Dr.4095 | *ass1* | *ASS1* | -0.943 | 7.85E-04 |
| NM_131175 | Dr.81279 | *opn1lw1* | *OPN1LW* | -0.947 | 7.61E-04 |
| NM_213122 | Dr.76585 | *sox4a* | *SOX4* | -0.950 | 1.95E-04 |
| NM_001077318 | Dr.52663 | *zgc:153764* | *CLCN5* | -0.956 | 2.22E-04 |
| NM_200559 | Dr.107708 | *saga* | *SAG* | -0.959 | 5.32E-04 |
| NM_201014 | Dr.9906 | *rom1b* | *ROM1B* | -0.959 | 6.63E-05 |
| NM_001002561 | Dr.84618 | *clic2* | *CLIC2* | -0.967 | 7.63E-07 |
| NM_001003534 | Dr.90943 | *mipa* | *MIP* | -0.974 | 1.49E-04 |
| NM_131868 | Dr.32527 | *gnat1* | *GNAT1* | -0.985 | 8.53E-06 |
| NM_152940 | Dr.14325 | *crx* | *OTX2* | -0.991 | 6.03E-05 |
| NM_130908 | Dr.32892 | *sox19a* | *SOX19A* | -0.993 | 2.54E-04 |
| NM_001201544 | Dr.85367 | *wdr17* | *WDR17* | -1.005 | 2.59E-04 |
| NM_194369 | Dr.75649 | *cd99l2* | *CD99L2* | -1.017 | 2.96E-04 |
| NM_200318 | Dr.82824 | *arl4ab* | *ARL4A* | -1.029 | 4.94E-07 |
| NM_131156 | Dr.162030 | *krt5* | *KRT8* | -1.037 | 2.15E-06 |
| NM_201174 | Dr.132758 | *rem1* | *GEM* | -1.050 | 1.83E-04 |
| NM_001111090 | Dr.134807 | *zgc:174224* | *ZNF729* | -1.050 | 1.41E-04 |
| NM_001039819 | Dr.104779 | *zgc:136930* | *N.A.* | -1.051 | 3.99E-05 |
| NM_001008615 | Dr.32244 | *hspb1* | *HSPB1* | -1.067 | 7.77E-05 |
| NM_001002513 | Dr.77685 | *slc1a4* | *SLC1A4* | -1.072 | 7.45E-06 |
| NM_001104649 | Dr.89850 | *flr* | *TTC30B* | -1.084 | 1.02E-04 |
| NM_001204332 | Dr.81960 | *gngt2b* | *GNGT2B* | -1.089 | 3.81E-07 |
| NM_200408 | Dr.12508 | *zgc:64085* | *TSPAN1* | -1.102 | 6.82E-05 |
| NM_213443 | Dr.75494 | *pitpnbl* | *PITPNBL* | -1.105 | 1.18E-06 |
| NM_131084 | Dr.354 | *rho* | *RHO* | -1.115 | 1.92E-08 |
| NM_131869 | Dr.81958 | *gnat2* | *GNAT2* | -1.120 | 9.35E-06 |
| NM_001145633 | Dr.17287 | *LOC100149820* | *FAM35A* | -1.127 | 5.72E-05 |
| NM_001002594 | Dr.78975 | *sgce* | *SGCE* | -1.146 | 1.92E-06 |
| NM_201048 | Dr.80443 | *pdia2* | *PDIA2* | -1.151 | 4.12E-08 |
| NM_001080701 | Dr.84809 | *slc32a1* | *SLC32A1* | -1.154 | 1.07E-05 |
| NM_001083065 | Dr.89776 | *si:dkeyp-85e10.2* | *NUGGC* | -1.158 | 2.51E-05 |
| NM_001001725 | Dr.2900 | *ift88* | *IFT88* | -1.183 | 2.28E-05 |
| NM_001009891 | Dr.76142 | *lancl1* | *LANCL1* | -1.191 | 3.04E-10 |
| NM_001128667 | Dr.105978 | *ptprn2* | *PTPRN2* | -1.224 | 5.01E-07 |
| NM_200709 | Dr.78131 | *lin7a* | *LIN7A* | -1.244 | 7.06E-08 |
| NM_200788 | Dr.84693 | *rdh8b* | *RDH8* | -1.246 | 9.49E-06 |
| NM_131762 | Dr.77880 | *cldna* | *CLDN4* | -1.259 | 3.13E-06 |
| NM_200996 | Dr.151875 | *pdk2* | *PDK2* | -1.267 | 1.90E-08 |
| NM_131691 | Dr.77753 | *s1pr1* | *S1PR1* | -1.285 | 1.45E-08 |
| NM_131319 | Dr.8194 | *opn1sw1* | *OPN1SW* | -1.337 | 3.34E-10 |
| NM_001003446 | Dr.77138 | *cpa2* | *CPA5* | -1.361 | 8.22E-07 |
| NM_173277 | Dr.132970 | *lamc1* | *LAMC1* | -1.383 | 1.53E-07 |
| NM_001045376 | Dr.90380 | *gabrr2a* | *GABRR1* | -1.446 | 1.66E-08 |
| NM_213202 | Dr.81932 | *gnb3b* | *GNB3* | -1.448 | 1.68E-11 |
| NM_131192 | Dr.81284 | *opn1sw2* | *OPN1SW2* | -1.613 | 7.93E-13 |
| NM_131253 | Dr.81282 | *opn1mw1* | *OPN1MW1* | -1.801 | 8.82E-18 |
| NM_001007459 | Dr.89620 | *slc35g2a* | *SLC35G2A* | -2.092 | 2.41E-20 |
| NM_001002443 | Dr.116975 | *opn1lw2* | *OPN1LW* | -2.196 | 1.50E-15 |

N.A. refers to not available.

**Table S3. DEGs in male adult TANs**

| Refseq | Unigene ID | Gene Symbol | Hs Gene Symbol | log2FC | pValue |
| --- | --- | --- | --- | --- | --- |
| NM_001017591 | Dr.80737 | *zgc:110307* | *CPM* | 2.161 | 1.76E-05 |
| NM_001076741 | Dr.87157 | *zgc:153027* | *ESM1* | 2.157 | 2.75E-05 |
| NM_001168267 | Dr.26022 | *cep290* | *CEP290* | 1.846 | 1.49E-04 |
| NM_212756 | Dr.67270 | *grn2* | *GRN2* | 1.821 | 3.34E-04 |
| NM_212671 | Dr.75748 | *orc6* | *ORC6* | 1.662 | 9.35E-04 |
| NM_001076622 | Dr.89419 | *itln1* | *ITLN1* | 1.650 | 3.29E-04 |
| NM_212648 | Dr.83182 | *dvl2* | *DVL2* | 1.575 | 1.30E-03 |
| NM_131873 | Dr.78757 | *her9* | *HER9* | 1.570 | 3.31E-04 |
| NM_001031840 | Dr.88842 | *arhgef11* | *ARHGEF11* | 1.523 | 1.37E-03 |
| NM_001003519 | Dr.33963 | *pltp* | *PLTP* | 1.510 | 1.83E-08 |
| NM_001100093 | Dr.38615 | *ercc6l* | *ERCC6L* | 1.474 | 2.46E-03 |
| NM_200600 | Dr.18537 | *traf3ip1* | *TRAF3IP1* | 1.454 | 2.89E-03 |
| NM_001079974 | Dr.75979 | *haus6* | *HAUS6* | 1.413 | 2.93E-03 |
| NM_001256205 | Dr.84109 | *ncapg* | *NCAPG* | 1.411 | 3.54E-03 |
| NM_001099232 | Dr.5434 | *zgc:165571* | *N.A.* | 1.395 | 4.06E-03 |
| NM_001089387 | Dr.148401 | *zgc:162964* | *ACBD3* | 1.372 | 4.52E-03 |
| NM_214816 | Dr.77355 | *tcf12* | *TCF12* | 1.366 | 6.25E-03 |
| NM_001007052 | Dr.86994 | *loh12cr1* | *LOH12CR1* | 1.316 | 5.09E-03 |
| NM_214773 | Dr.1508 | *acp5a* | *ACP5* | 1.315 | 3.94E-03 |
| NM_194414 | Dr.81778 | *mmp14b* | *MMP14* | 1.292 | 6.56E-03 |
| NM_181761 | Dr.75997 | *anxa2a* | *ANXA2* | 1.287 | 2.39E-03 |
| NM_200276 | Dr.40877 | *ncor1* | *NCOR1* | 1.267 | 1.48E-03 |
| NM_001077782 | Dr.80569 | *fgf12a* | *FGF12* | 1.263 | 7.72E-03 |
| NM_001128258 | Dr.18627 | *ttc21b* | *TTC21B* | 1.259 | 7.80E-03 |
| NM_200782 | Dr.81455 | *kcnip3* | *KCNIP3* | 1.255 | 8.03E-03 |
| NM_001089324 | Dr.77158 | *mtif2* | *MTIF2* | 1.253 | 6.09E-03 |
| NM_001033094 | Dr.94519 | *ephb2b* | *EPHB2* | 1.251 | 7.81E-03 |
| NM_001002465 | Dr.79071 | *timm13* | *TIMM13* | 1.251 | 7.85E-03 |
| NM_001080038 | Dr.37679 | *zgc:158343* | *N.A.* | 1.247 | 5.13E-03 |
| NM_199950 | Dr.6431 | *socs3a* | *SOCS3* | 1.243 | 5.35E-03 |
| NM_001037434 | Dr.79483 | *poglut1* | *POGLUT1* | 1.241 | 8.25E-03 |
| NM_001008402 | Dr.79599 | *extl3* | *EXTL3* | 1.238 | 7.98E-03 |
| NM_200964 | Dr.77523 | *atf3* | *ATF3* | 1.234 | 2.34E-03 |
| NM_213216 | Dr.75129 | *arglu1a* | *ARGLU1A* | 1.218 | 8.02E-03 |
| NM_213488 | Dr.26688 | *actr5* | *ACTR5* | 1.217 | 4.05E-03 |
| NM_001045845 | Dr.83024 | *zgc:153293* | *N.A.* | 1.209 | 3.97E-03 |
| NM_001013323 | Dr.39146 | *wars2* | *WARS2* | 1.202 | 1.02E-02 |
| NM_001044715 | Dr.81347 | *nrd1* | *NRD1* | 1.200 | 6.47E-03 |
| NM_001130612 | Dr.99089 | *atxn10* | *ATXN10* | 1.194 | 1.05E-02 |
| NM_200522 | Dr.76587 | *zc3h13* | *ZC3H13* | 1.194 | 4.58E-03 |
| NM_001044767 | Dr.77930 | *setdb1a* | *SETDB1* | 1.185 | 2.97E-03 |
| NM_001145599 | Dr.78182 | *LOC568087* | *CCAR1* | 1.172 | 8.69E-03 |
| NM_001001834 | Dr.15827 | *ift52* | *IFT52* | 1.158 | 1.14E-02 |
| NM_001136487 | Dr.149198 | *zswim6* | *ZSWIM6* | 1.154 | 8.97E-03 |
| NM_001007773 | Dr.76636 | *gna11b* | *GNA11B* | 1.147 | 3.86E-03 |
| NM_001003994 | Dr.1836 | *pddc1* | *MORC2* | 1.145 | 2.03E-03 |
| NM_001123373 | N.A. | *N.A.* | *ASPM* | 1.142 | 3.51E-02 |
| NM_200811 | Dr.82615 | *zgc:73380* | *CFAP20* | 1.129 | 6.38E-03 |
| NM_001110374 | Dr.148688 | *zgc:171630* | *SERPINH1* | 1.128 | 1.42E-02 |
| NM_212639 | Dr.77708 | *ints10* | *INTS10* | 1.126 | 1.15E-02 |
| NM_001002641 | Dr.75329 | *acss2* | *ACSS2* | 1.126 | 4.17E-03 |
| NM_201156 | Dr.160041 | *pex5* | *PEX5* | 1.122 | 4.86E-03 |
| NM_001033752 | Dr.151704 | *cwf19l2* | *CWF19L2* | 1.115 | 1.47E-02 |
| NM_001006005 | Dr.1438 | *dut* | *DUT* | 1.114 | 1.47E-02 |
| NM_001111226 | Dr.16053 | *hbegfa* | *HBEGF* | 1.106 | 1.32E-02 |
| NM_213468 | Dr.76207 | *aqp3a* | *AQP3* | 1.105 | 1.50E-02 |
| NM_001110169 | Dr.117233 | *zgc:171957* | *N.A.* | 1.098 | 1.53E-02 |
| NM_001044897 | Dr.122480 | *vtg1* | *VTG1* | 1.094 | 1.49E-02 |
| NM_198872 | Dr.78376 | *depdc1a* | *DEPDC1* | 1.094 | 1.64E-02 |
| NM_001089403 | Dr.33005 | *zgc:158281* | *PLEKHM1* | 1.090 | 1.51E-03 |
| NM_001089367 | Dr.79101 | *smarcc1b* | *SMARCC2* | 1.088 | 1.08E-02 |
| NM_001077274 | Dr.74454 | *zgc:153129* | *CSTB* | 1.088 | 4.84E-03 |
| NM_205606 | Dr.83623 | *zgc:85851* | *TADA1* | 1.085 | 1.29E-02 |
| NM_001002216 | Dr.83410 | *gadd45ab* | *GADD45A* | 1.080 | 1.68E-02 |
| NM_131882 | Dr.84258 | *cxcr4a* | *CXCR4* | 1.078 | 1.68E-02 |
| NM_001136256 | Dr.94178 | *zgc:194878* | *N.A.* | 1.074 | 1.74E-02 |
| NM_001039988 | Dr.85483 | *zgc:114169* | *C10orf32* | 1.074 | 1.32E-02 |
| NM_001160291 | Dr.82008 | *zgc:92773* | *NAA38* | 1.072 | 1.33E-02 |
| NM_001077331 | Dr.80995 | *zgc:154125* | *N.A.* | 1.070 | 1.81E-02 |
| NM_001002653 | Dr.77198 | *serpinb1* | *SERPINB1* | 1.068 | 1.32E-02 |
| NM_131461 | Dr.75820 | *tie2* | *TEK* | 1.067 | 1.33E-02 |
| NM_001076713 | Dr.87475 | *zgc:153444* | *FAM213A* | 1.065 | 1.04E-02 |
| NM_001100039 | Dr.106046 | *arid3a* | *ARID3A* | 1.063 | 1.74E-02 |
| NM_001002436 | Dr.76852 | *psmd9* | *PSMD9* | 1.062 | 4.88E-02 |
| NM_001007456 | Dr.77313 | *cdca8* | *CDCA8* | 1.058 | 1.89E-02 |
| NM_001127256 | Dr.9683 | *triqk* | *TRIQK* | 1.053 | 9.02E-03 |
| NM_001037372 | Dr.34141 | *ptcd3* | *PTCD3* | 1.052 | 8.58E-03 |
| NM_001003451 | Dr.81043 | *dusp2* | *DUSP2* | 1.052 | 1.83E-02 |
| NM_001003618 | Dr.111296 | *mphosph6* | *MPHOSPH6* | 1.051 | 1.55E-02 |
| NM_213366 | Dr.79551 | *brd7* | *BRD7* | 1.051 | 1.44E-02 |
| NM_001122946 | N.A. | *N.A.* | *N.A.* | 1.049 | 6.77E-03 |
| NM_001044339 | Dr.159723 | *zc3h18* | *ZC3H18* | 1.049 | 2.16E-02 |
| NM_001045450 | Dr.13292 | *zgc:153425* | *Anapc15* | 1.045 | 1.80E-02 |
| NM_001002189 | Dr.76497 | *bambib* | *BAMBI* | 1.043 | 1.99E-02 |
| NM_001100032 | Dr.11514 | *pleca* | *PLEC* | 1.040 | 2.71E-03 |
| NM_199276 | Dr.24246 | *h1fx* | *H1FX* | 1.039 | 1.81E-03 |
| NM_200039 | Dr.79812 | *sdf2* | *SDF2* | 1.039 | 2.01E-02 |
| NM_001002741 | Dr.14523 | *rnpep* | *RNPEP* | 1.037 | 2.04E-02 |
| NM_001037378 | Dr.82349 | *zgc:123274* | *PRIMPOL* | 1.031 | 1.80E-02 |
| NM_198807 | Dr.80157 | *pif1* | *PIF1* | 1.018 | 1.61E-02 |
| NM_001003539 | Dr.81887 | *nudcd2* | *NUDCD2* | 1.015 | 2.00E-02 |
| NM_001005772 | Dr.14847 | *atp6v1c1b* | *ATP6V1C1* | 1.003 | 1.46E-02 |
| NM_001277446 | N.A. | *N.A.* | *N.A.* | 1.001 | 1.40E-02 |
| NM_001007380 | Dr.79574 | *churc1* | *CHURC1* | 0.993 | 7.37E-03 |
| NM_001110762 | Dr.94197 | *vasnb* | *VASN* | 0.986 | 3.97E-02 |
| NM_001199735 | Dr.4773 | *zwilch* | *ZWILCH* | 0.984 | 1.23E-02 |
| NM_001256179 | Dr.86533 | *mbip* | *MBIP* | 0.983 | 2.57E-02 |
| NM_001002375 | Dr.25733 | *rdbp* | *RDBP* | 0.983 | 2.10E-02 |
| NM_001080164 | Dr.133254 | *si:ch211-212o1.2* | *UBE2V1* | 0.983 | 1.53E-02 |
| NM_001077760 | Dr.121164 | *zgc:152816* | *MATR3* | 0.982 | 1.59E-02 |
| NM_200338 | Dr.80194 | *ap4b1l* | *AP4B1* | 0.980 | 1.36E-02 |
| NM_001118891 | Dr.27086 | *s1pr4* | *S1PR4* | 0.977 | 8.16E-03 |
| NM_200658 | Dr.85117 | *ccdc43* | *CCDC43* | 0.977 | 2.29E-02 |
| NM_001040094 | Dr.78554 | *cpox* | *CPOX* | 0.975 | 2.23E-02 |
| NM_001089445 | Dr.159818 | *ccnd2a* | *CCND2* | 0.974 | 2.47E-02 |
| NM_213518 | Dr.977 | *mus81* | *MUS81* | 0.972 | 2.68E-02 |
| NM_200682 | Dr.78530 | *akap17a* | *AKAP17A* | 0.970 | 6.07E-03 |
| NM_001130628 | Dr.92348 | *btr09* | *BTR09* | 0.969 | 2.54E-02 |
| NM_001003552 | Dr.82017 | *slc16a9b* | *SLC16A9* | 0.966 | 2.50E-02 |
| NM_001034179 | Dr.115287 | *rab6b* | *RAB6B* | 0.966 | 2.69E-02 |
| NM_201073 | Dr.114938 | *h2afx* | *H2AFX* | 0.966 | 2.52E-02 |
| NM_001135143 | Dr.116869 | *znf831* | *ZNF831* | 0.965 | 2.36E-02 |
| NM_001166255 | Dr.26623 | *camsap1a* | *CAMSAP1* | 0.963 | 2.16E-02 |
| NM_001162502 | Dr.150361 | *ncapd2* | *NCAPD2* | 0.963 | 2.52E-02 |
| NM_212600 | Dr.12392 | *rchy1* | *RCHY1* | 0.962 | 1.95E-02 |
| NM_001037114 | Dr.5714 | *nubp2* | *NUBP2* | 0.959 | 2.75E-02 |
| NM_001044343 | Dr.75149 | *plod3* | *PLOD3* | 0.959 | 1.91E-02 |
| NM_001045392 | Dr.86353 | *batf3* | *BATF3* | 0.958 | 2.20E-02 |
| NM_001017739 | Dr.75744 | *mad2l1* | *MAD2L1* | 0.957 | 2.35E-02 |
| NM_001202441 | Dr.92966 | *nphp3* | *NPHP3* | 0.957 | 2.67E-02 |
| NM_001045015 | Dr.74054 | *ap3d1* | *AP3D1* | 0.955 | 2.23E-02 |
| NM_200389 | Dr.84295 | *obfc1* | *OBFC1* | 0.952 | 2.19E-02 |
| NM_001004659 | Dr.125114 | *zgc:103697* | *CXorf56* | 0.952 | 2.59E-02 |
| NM_201003 | Dr.82352 | *ubtfl* | *UBTF* | 0.950 | 5.42E-03 |
| NM_001082551 | Dr.78031 | *whsc1* | *WHSC1* | 0.949 | 1.07E-02 |
| NM_001002335 | Dr.75709 | *kpna2* | *KPNA2* | 0.948 | 1.44E-02 |
| NM_001128774 | Dr.113445 | *zgc:195152* | *N.A.* | 0.945 | 2.95E-02 |
| NM_001082849 | Dr.33010 | *mcm6* | *MCM6* | 0.942 | 1.10E-02 |
| NM_001002662 | Dr.39062 | *rgs2* | *RGS2* | 0.940 | 1.91E-02 |
| NM_001002633 | Dr.132558 | *cryzl1* | *CRYZL1* | 0.932 | 2.83E-02 |
| NM_001128818 | Dr.143348 | *tnfsf18* | *TNFSF18* | 0.932 | 1.87E-02 |
| NM_001075106 | Dr.84210 | *zgc:152785* | *CCDC9* | 0.932 | 3.12E-02 |
| NM_001111175 | Dr.29913 | *zgc:171566* | *GGH* | 0.930 | 3.10E-02 |
| NM_001003744 | Dr.20193 | *kras* | *KRAS* | 0.930 | 1.25E-02 |
| NM_200716 | Dr.117007 | *zgc:73111* | *WDR83OS* | 0.930 | 1.99E-02 |
| NM_001077295 | Dr.81555 | *ube2t* | *UBE2T* | 0.927 | 2.85E-02 |
| NM_001111197 | Dr.82691 | *si:ch211-12e1.4* | *AFG3L1P* | 0.925 | 2.95E-02 |
| NM_131859 | Dr.79292 | *klf3* | *KLF3* | 0.921 | 2.42E-02 |
| NM_001079973 | Dr.104398 | *zgc:158363* | *TAF15* | 0.919 | 3.03E-02 |
| NM_200280 | Dr.16120 | *ptgesl* | *PTGES2* | 0.917 | 2.54E-02 |
| NM_001004644 | Dr.12174 | *zgc:101095* | *KIFAP3* | 0.917 | 2.42E-02 |
| NM_001045308 | Dr.77881 | *zgc:136474* | *C1orf63* | 0.915 | 2.73E-02 |
| NM_001159912 | Dr.75482 | *lnpb* | *KIAA1715* | 0.913 | 2.92E-02 |
| NM_199794 | Dr.14259 | *memo1* | *MEMO1* | 0.913 | 2.51E-02 |
| NM_001113798 | Dr.14226 | *zgc:175088* | *FBXO2* | 0.911 | 2.87E-02 |
| NM_001128736 | Dr.113886 | *thap4* | *THAP4* | 0.909 | 2.85E-02 |
| NM_131000 | Dr.20912 | *alcama* | *ALCAM* | 0.908 | 3.35E-02 |
| NM_001008631 | Dr.86643 | *jazf1b* | *JAZF1B* | 0.907 | 3.14E-02 |
| NM_001002403 | Dr.90408 | *dnase1l3l* | *DNASE1L3* | 0.906 | 1.32E-02 |
| NM_201083 | Dr.114403 | *fus* | *FUS* | 0.906 | 7.19E-03 |
| NM_199635 | Dr.79558 | *zbtb16a* | *ZBTB16* | 0.905 | 6.31E-03 |
| NM_001128707 | Dr.159849 | *nf2a* | *NF2* | 0.902 | 3.43E-02 |
| NM_213528 | Dr.80528 | *trim36* | *TRIM36* | 0.899 | 2.75E-02 |
| NM_201503 | Dr.81475 | *mmp13a* | *MMP13* | 0.898 | 3.01E-02 |
| NM_001083823 | Dr.40360 | *cd22* | *SIGLEC1* | 0.898 | 3.54E-02 |
| NM_001098251 | Dr.43752 | *bbs1* | *BBS1* | 0.898 | 3.41E-02 |
| NM_001077789 | Dr.52561 | *zgc:154077* | *C12orf4* | 0.896 | 2.61E-02 |
| NM_001008596 | Dr.132987 | *lxn* | *LXN* | 0.896 | 2.99E-03 |
| NM_001076721 | Dr.78346 | *zgc:152925* | *N.A.* | 0.895 | 3.40E-02 |
| NM_001007353 | Dr.16501 | *rp9* | *RP9* | 0.894 | 3.48E-02 |
| NM_001004494 | Dr.5696 | *prc1a* | *PRC1A* | 0.894 | 2.55E-02 |
| NM_001193541 | Dr.75528 | *smc5* | *SMC5* | 0.893 | 1.11E-02 |
| NM_001102619 | Dr.135035 | *inpp5e* | *INPP5E* | 0.889 | 3.65E-02 |
| NM_001079658 | Dr.85202 | *zgc:158320* | *N.A.* | 0.889 | 3.66E-02 |
| NM_001002551 | Dr.18504 | *nsmce1* | *NSMCE1* | 0.889 | 3.51E-02 |
| NM_131406 | Dr.193 | *raraa* | *RARA* | 0.889 | 1.68E-02 |
| NM_001003531 | Dr.160020 | *fam151b* | *FAM151B* | 0.886 | 3.61E-02 |
| NM_001002722 | Dr.18870 | *ttc33* | *TTC33* | 0.884 | 1.87E-02 |
| NM_001161511 | Dr.1726 | *nol6* | *NOL6* | 0.884 | 3.40E-02 |
| NM_001033753 | Dr.116460 | *zgc:113314* | *N.A.* | 0.883 | 2.84E-02 |
| NM_200953 | Dr.133205 | *klc1a* | *KLC1* | 0.880 | 2.07E-02 |
| NM_001143840 | Dr.76198 | *apc* | *APC* | 0.880 | 1.92E-02 |
| NM_001017709 | Dr.88717 | *gpd1a* | *GPD1L* | 0.879 | 3.76E-02 |
| NM_212655 | Dr.150395 | *c20orf14* | *PRPF6* | 0.878 | 9.07E-03 |
| NM_001005926 | Dr.107289 | *acn9* | *ACN9* | 0.877 | 3.74E-02 |
| NM_001002107 | Dr.31100 | *nudt15* | *NUDT15* | 0.875 | 3.84E-02 |
| NM_001123060 | Dr.103330 | *camk1da* | *CAMK1G* | 0.872 | 3.05E-02 |
| NM_001128574 | Dr.110632 | *il21* | *IL21* | 0.872 | 3.74E-02 |
| NM_001033729 | Dr.41732 | *zgc:114148* | *AASDHPPT* | 0.872 | 3.90E-02 |
| NM_199924 | Dr.75357 | *fam192a* | *FAM192A* | 0.870 | 2.72E-02 |
| NM_001080600 | Dr.82412 | *shq1* | *SHQ1* | 0.869 | 2.97E-02 |
| NM_200847 | Dr.14683 | *myeov2* | *MYEOV2* | 0.868 | 3.80E-02 |
| NM_200740 | Dr.12918 | *fhit* | *FHIT* | 0.867 | 3.74E-02 |
| NM_001159820 | Dr.80522 | *triap1* | *TRIAP1* | 0.865 | 3.75E-02 |
| NM_001080037 | Dr.66579 | *atp13a2* | *ATP13A2* | 0.864 | 2.99E-02 |
| NM_173246 | Dr.77075 | *pole2* | *POLE2* | 0.863 | 4.02E-02 |
| NM_001042746 | Dr.16111 | *tshz1* | *TSHZ1* | 0.863 | 1.57E-02 |
| NM_213184 | Dr.79912 | *nfkbiaa* | *NFKBIA* | 0.862 | 2.34E-02 |
| NM_001030235 | Dr.14063 | *eml2* | *EML1* | 0.862 | 9.05E-03 |
| NM_001098181 | Dr.86090 | *zgc:163107* | *C16orf62* | 0.861 | 8.30E-03 |
| NM_001160707 | Dr.75270 | *tfpt* | *TFPT* | 0.860 | 4.02E-02 |
| NM_001080000 | Dr.11530 | *zgc:152863* | *N.A.* | 0.860 | 2.78E-02 |
| NM_213018 | Dr.76809 | *katnb1* | *KATNB1* | 0.859 | 1.62E-02 |
| NM_200201 | Dr.83003 | *mitd1* | *MITD1* | 0.858 | 3.79E-02 |
| NM_001143754 | Dr.112272 | *csf3* | *CSF3* | 0.856 | 2.65E-02 |
| NM_001003555 | Dr.120097 | *cenpp* | *CENPP* | 0.856 | 3.09E-02 |
| NM_200514 | Dr.84923 | *lgals2b* | *LGALS1* | 0.856 | 4.00E-02 |
| NM_212575 | Dr.132194 | *mtx2* | *MTX2* | 0.856 | 1.23E-02 |
| NM_001025176 | Dr.126607 | *zgc:113984* | *HIST1H3A* | 0.856 | 4.13E-02 |
| NM_001037690 | Dr.87015 | *zgc:123280* | *SDR42E1* | 0.856 | 3.62E-02 |
| NM_200954 | Dr.13796 | *upf3b* | *UPF3A* | 0.852 | 3.78E-02 |
| NM_001031837 | Dr.48541 | *acsl1* | *ACSL1* | 0.852 | 3.93E-02 |
| NM_001077788 | Dr.11461 | *rab3gap1* | *RAB3GAP1* | 0.852 | 1.42E-02 |
| NM_001080644 | Dr.128545 | *trappc12* | *TRAPPC12* | 0.852 | 3.27E-02 |
| NM_001077468 | Dr.13693 | *zgc:153377* | *MRPL21* | 0.850 | 3.83E-02 |
| NM_001004673 | Dr.48734 | *tceb1a* | *TCEB1* | 0.850 | 3.69E-02 |
| NM_001089368 | Dr.76104 | *pdcd11* | *PDCD11* | 0.849 | 9.63E-03 |
| NM_001040305 | Dr.83000 | *brat1* | *BRAT1* | 0.848 | 2.92E-02 |
| NM_001002077 | Dr.81719 | *lsm6* | *LSM6* | 0.848 | 4.21E-02 |
| NM_200786 | Dr.83748 | *xrcc4* | *XRCC4* | 0.847 | 3.46E-02 |
| NM_001045011 | Dr.77274 | *rgma* | *RGMA* | 0.847 | 3.53E-02 |
| NM_001159928 | Dr.83425 | *pih1d1* | *PIH1D1* | 0.843 | 4.22E-02 |
| NM_001159825 | Dr.13705 | *zgc:112305* | *POLR2L* | 0.843 | 4.32E-02 |
| NM_001002134 | Dr.79838 | *tmed4* | *TMED9* | 0.842 | 3.28E-02 |
| NM_001007375 | Dr.32436 | *si:dkey-42i9.4* | *N.A.* | 0.842 | 1.17E-02 |
| NM_001045215 | Dr.154223 | *foxg1c* | *FOXG1C* | 0.840 | 4.25E-02 |
| NM_201082 | Dr.1956 | *calub* | *CALUB* | 0.840 | 3.14E-02 |
| NM_199930 | Dr.29131 | *larp7* | *LARP7* | 0.840 | 4.14E-02 |
| NM_001014301 | Dr.134841 | *tcp11l1* | *TCP11L1* | 0.839 | 4.21E-02 |
| NM_001002412 | Dr.126015 | *zgc:92683* | *ZFYVE19* | 0.837 | 1.56E-02 |
| NM_001077574 | Dr.8155 | *med17* | *MED17* | 0.835 | 1.18E-02 |
| NM_131597 | Dr.7209 | *nme6* | *NME6* | 0.834 | 4.44E-02 |
| NM_200260 | Dr.7975 | *ppp4r1* | *PPP4R1* | 0.831 | 9.60E-03 |
| NM_200728 | Dr.76327 | *ssu72* | *SSU72* | 0.831 | 3.53E-02 |
| NM_001004531 | Dr.85982 | *znf511* | *ZNF511* | 0.831 | 3.31E-02 |
| NM_001045325 | Dr.79792 | *krr1* | *KRR1* | 0.830 | 3.79E-02 |
| NM_212702 | Dr.75964 | *brd3b* | *BRD4* | 0.829 | 2.77E-02 |
| NM_001111242 | Dr.9682 | *zgc:172053* | *N.A.* | 0.828 | 4.55E-02 |
| NM_001204157 | Dr.33003 | *zdhhc6* | *ZDHHC6* | 0.828 | 2.15E-02 |
| NM_001099237 | Dr.80174 | *eif4ba* | *EIF4B* | 0.828 | 1.53E-02 |
| NM_201291 | Dr.32819 | *id2a* | *ID2* | 0.828 | 3.05E-02 |
| NM_001002433 | Dr.123434 | *dnajc9* | *DNAJC9* | 0.828 | 3.08E-02 |
| NM_001045013 | Dr.72843 | *chaf1a* | *CHAF1A* | 0.827 | 2.75E-02 |
| NM_001030212 | Dr.77356 | *znf292b* | *ZNF292B* | 0.826 | 1.67E-02 |
| NM_001110450 | Dr.91775 | *wash* | *WASH2P* | 0.826 | 4.52E-02 |
| NM_001145617 | Dr.40212 | *LOC796750* | *METTL18* | 0.825 | 4.57E-02 |
| NM_001045294 | Dr.75546 | *vtg4* | *VTG4* | 0.824 | 4.00E-02 |
| NM_001115138 | Dr.5648 | *ewsr1a* | *EWSR1* | 0.822 | 3.15E-02 |
| NM_001089553 | Dr.40317 | *rfx1a* | *RFX1* | 0.821 | 4.30E-02 |
| NM_200248 | Dr.84319 | *polm* | *DNTT* | 0.821 | 4.11E-02 |
| NM_001012491 | Dr.76472 | *zgc:113418* | *ZNF729* | 0.818 | 4.46E-02 |
| NM_001110467 | Dr.90198 | *sgk3* | *SGK3* | 0.816 | 4.35E-02 |
| NM_200890 | Dr.29094 | *naprt1* | *NAPRT1* | 0.815 | 4.07E-02 |
| NM_001045847 | Dr.76457 | *nprl2* | *NPRL2* | 0.815 | 4.74E-02 |
| NM_001002605 | Dr.80771 | *sirt5* | *SIRT5* | 0.815 | 4.44E-02 |
| NM_001126468 | Dr.155792 | *si:dkeyp-94h10.5* | *MTERF4* | 0.815 | 4.74E-02 |
| NM_001004565 | Dr.80280 | *adprhl2* | *ADPRHL2* | 0.813 | 4.76E-02 |
| NM_001002119 | Dr.75677 | *tpm2* | *TPM4* | 0.811 | 4.76E-02 |
| NM_001113501 | Dr.118320 | *nos2b* | *NOS2* | 0.810 | 3.48E-02 |
| NM_001033103 | Dr.83476 | *ndufa12* | *NDUFA12* | 0.810 | 3.99E-02 |
| NM_200270 | Dr.82812 | *zgc:56576* | *C14orf166* | 0.809 | 3.56E-02 |
| NM_001007770 | Dr.78176 | *med28* | *MED28* | 0.808 | 3.59E-02 |
| NM_001003456 | Dr.34068 | *txndc17* | *TXNDC17* | 0.807 | 3.46E-02 |
| NM_001245092 | Dr.132781 | *si:dkey-4c15.5* | *ZNF729* | 0.807 | 4.83E-02 |
| NM_213024 | Dr.28184 | *dynll1* | *BC048507* | 0.807 | 2.88E-02 |
| NM_001089492 | Dr.79383 | *ncapg2* | *NCAPG2* | 0.806 | 4.73E-02 |
| NM_001245964 | Dr.160792 | *l3mbtl3* | *L3MBTL3* | 0.805 | 4.91E-02 |
| NM_199823 | Dr.6092 | *hirip3* | *HIRIP3* | 0.805 | 4.84E-02 |
| NM_214683 | Dr.2918 | *safb* | *SAFB2* | 0.804 | 3.67E-02 |
| NM_001128751 | Dr.77563 | *fignl1* | *FIGNL1* | 0.803 | 3.59E-02 |
| NM_001037698 | Dr.81504 | *snx2* | *SNX1* | 0.802 | 2.99E-02 |
| NM_200623 | Dr.4658 | *wdr46* | *WDR46* | 0.802 | 3.19E-02 |
| NM_201461 | Dr.77265 | *copeb* | *KLF6* | 0.801 | 3.00E-02 |
| NM_001076614 | Dr.80765 | *zgc:152927* | *PIGS* | 0.800 | 2.23E-02 |
| NM_001126461 | Dr.11545 | *etv5a* | *ETV5* | 0.797 | 4.30E-02 |
| NM_001077281 | Dr.82557 | *zgc:153341* | *RG9MTD3* | 0.796 | 2.49E-02 |
| NM_001089521 | Dr.51212 | *atpif1* | *ATPIF1* | 0.795 | 4.43E-02 |
| NM_213000 | Dr.107123 | *chn1* | *CHN1* | 0.794 | 3.56E-02 |
| NM_205709 | Dr.78635 | *bub3* | *BUB3* | 0.793 | 3.62E-02 |
| NM_001122619 | Dr.160046 | *sc:d217* | *SC:D217* | 0.792 | 4.37E-02 |
| NM_153657 | Dr.113864 | *ptgs2a* | *PTGS2* | 0.792 | 3.32E-02 |
| NM_001105128 | Dr.81885 | *msrb1b* | *MSRB1B* | 0.792 | 4.90E-02 |
| NM_001102671 | Dr.135804 | *vtg7* | *VTG7* | 0.791 | 3.93E-02 |
| NM_131355 | Dr.80609 | *kif23* | *KIF23* | 0.791 | 4.46E-02 |
| NM_200754 | Dr.11058 | *hsbp1* | *HSBP1* | 0.791 | 3.82E-02 |
| NM_001004651 | Dr.84954 | *ndufs6* | *NDUFS6* | 0.789 | 4.92E-02 |
| NM_001045090 | Dr.119416 | *si:ch211-199g17.1* | *FAM83H* | 0.788 | 1.85E-02 |
| NM_001030102 | Dr.109638 | *brms1la* | *BRMS1L* | 0.788 | 4.37E-02 |
| NM_200653 | Dr.5568 | *atrx* | *ATRX* | 0.786 | 2.03E-02 |
| NM_201206 | Dr.75878 | *cox15* | *COX15* | 0.785 | 4.10E-02 |
| NM_001271801 | N.A. | *N.A.* | *N.A.* | 0.785 | 4.91E-02 |
| NM_200735 | Dr.82894 | *vamp4* | *VAMP4* | 0.783 | 3.61E-02 |
| NM_001172556 | Dr.132390 | *bxdc2* | *RAD1* | 0.782 | 4.79E-02 |
| NM_131269 | Dr.79155 | *tcf7l1a* | *TCF7L1* | 0.782 | 3.16E-02 |
| NM_001128328 | Dr.39037 | *mrps31* | *MRPS31* | 0.781 | 3.45E-02 |
| NM_001202418 | Dr.75927 | *phf2* | *PHF2* | 0.781 | 3.35E-02 |
| NM_001017573 | Dr.84168 | *psmd5* | *PSMD5* | 0.781 | 4.82E-02 |
| NM_001044968 | Dr.11512 | *plcg2* | *PLCG2* | 0.779 | 1.53E-02 |
| NM_001030065 | Dr.78735 | *ilf3a* | *ILF3* | 0.779 | 3.47E-02 |
| NM_213219 | Dr.105980 | *akap8l* | *AKAP8L* | 0.779 | 2.11E-02 |
| NM_200454 | Dr.13952 | *ptpn11b* | *PTPN11B* | 0.778 | 3.90E-02 |
| NM_001128699 | Dr.52204 | *papss1* | *PAPSS1* | 0.778 | 4.08E-02 |
| NM_201064 | Dr.76607 | *zgc:66475* | *N.A.* | 0.775 | 4.56E-02 |
| NM_199792 | Dr.26610 | *znf710a* | *ZNF710* | 0.768 | 3.99E-02 |
| NM_213064 | Dr.151869 | *zgc:55943* | *C1orf21* | 0.767 | 4.43E-02 |
| NM_001017679 | Dr.81863 | *zgc:112466* | *STARD4* | 0.760 | 4.70E-02 |
| NM_001082805 | Dr.81759 | *kdm4b* | *KDM4B* | 0.758 | 4.32E-02 |
| NM_213304 | Dr.81607 | *socs3b* | *SOCS3* | 0.757 | 1.54E-02 |
| NM_001002081 | Dr.83576 | *snrpd3* | *SNRPD3* | 0.756 | 2.81E-02 |
| NM_200891 | Dr.81650 | *zgc:77880* | *N.A.* | 0.756 | 4.55E-02 |
| NM_001080074 | Dr.79635 | *zgc:158316* | *FAM126B* | 0.755 | 3.88E-02 |
| NM_001098189 | Dr.39353 | *zgc:162584* | *VPS16* | 0.755 | 2.29E-02 |
| NM_199729 | Dr.75435 | *gtf2f1* | *GTF2F1* | 0.754 | 4.73E-02 |
| NM_001002316 | Dr.84279 | *ltv1* | *LTV1* | 0.753 | 3.74E-02 |
| NM_001077717 | Dr.80994 | *zgc:154024* | *GGACT* | 0.753 | 4.64E-02 |
| NM_001126436 | Dr.17352 | *pigx* | *PIGX* | 0.752 | 4.86E-02 |
| NM_201103 | Dr.80178 | *cwc27* | *CWC27* | 0.751 | 4.54E-02 |
| NM_001007774 | Dr.4101 | *bcas2* | *BCAS2* | 0.749 | 1.83E-02 |
| NM_001002385 | Dr.31778 | *zgc:92052* | *IFT74* | 0.748 | 4.79E-02 |
| NM_200436 | Dr.83760 | *znf800b* | *ZNF800* | 0.747 | 4.33E-02 |
| NM_131018 | Dr.75813 | *htt* | *HTT* | 0.745 | 2.74E-02 |
| NM_001003545 | Dr.77868 | *entpd1* | *ENTPD2* | 0.745 | 2.92E-02 |
| NM_199834 | Dr.80343 | *zgc:55794* | *VWA9* | 0.744 | 3.91E-02 |
| NM_001105527 | Dr.114568 | *zgc:171711* | *C19orf66* | 0.743 | 3.69E-02 |
| NM_212844 | Dr.30443 | *il1b* | *IL1B* | 0.743 | 4.49E-02 |
| NM_001204250 | Dr.80816 | *zgc:194167* | *N.A.* | 0.742 | 4.57E-02 |
| NM_200218 | Dr.79519 | *atp5s* | *ATP5S* | 0.742 | 4.60E-02 |
| NM_001007352 | Dr.88655 | *rnf24* | *RNF24* | 0.740 | 4.49E-02 |
| NM_001034980 | Dr.134790 | *vti1a* | *VTI1A* | 0.739 | 3.39E-02 |
| NM_001044914 | Dr.73064 | *trim8* | *TRIM8* | 0.738 | 1.65E-02 |
| NM_213133 | Dr.75946 | *marcksl1a* | *MARCKSL1A* | 0.737 | 4.12E-02 |
| NM_001082825 | Dr.41190 | *ddit3* | *DDIT3* | 0.735 | 3.79E-03 |
| NM_205645 | Dr.6091 | *pigf* | *PIGF* | 0.734 | 4.63E-02 |
| NM_201088 | Dr.78689 | *parn* | *PARN* | 0.733 | 2.05E-02 |
| NM_001039639 | Dr.40859 | *abcg2c* | *ABCG2C* | 0.732 | 3.58E-02 |
| NM_001006042 | Dr.36945 | *fip1l1b* | *LNX1* | 0.731 | 3.18E-02 |
| NM_213471 | Dr.77368 | *stxbp3* | *STXBP3* | 0.731 | 2.99E-02 |
| NM_130929 | Dr.73782 | *nme7* | *NME7* | 0.724 | 4.94E-02 |
| NM_200823 | Dr.78768 | *zgc:66125* | *KIF4A* | 0.723 | 3.41E-02 |
| NM_001114681 | Dr.5022 | *cpsf1* | *CPSF1* | 0.719 | 3.48E-02 |
| NM_174863 | Dr.1706 | *rock2a* | *ROCK2A* | 0.719 | 2.94E-02 |
| NM_198913 | Dr.76603 | *mcm4* | *MCM4* | 0.718 | 2.93E-02 |
| NM_001006072 | Dr.90096 | *acot8* | *ACOT8* | 0.718 | 4.61E-02 |
| NM_001017763 | Dr.83238 | *zgc:112084* | *HEXA* | 0.716 | 1.68E-02 |
| NM_001122784 | Dr.76817 | *matr3l* | *MATR3* | 0.712 | 3.58E-02 |
| NM_201160 | Dr.14021 | *hibadhb* | *HIBADH* | 0.711 | 3.58E-02 |
| NM_001024448 | Dr.78058 | *myh11a* | *MYH11* | 0.711 | 2.54E-02 |
| NM_212810 | Dr.76942 | *smc1al* | *SMC1A* | 0.709 | 4.40E-02 |
| NM_199611 | Dr.77297 | *arg2* | *ARG1* | 0.700 | 4.63E-02 |
| NM_200372 | Dr.84358 | *sft2d1* | *SFT2D1* | 0.699 | 3.60E-02 |
| NM_001037688 | Dr.88180 | *hps1* | *HPS1* | 0.697 | 3.75E-02 |
| NM_200141 | Dr.119861 | *zw10* | *ZW10* | 0.695 | 4.73E-02 |
| NM_201344 | Dr.23557 | *ndel1a* | *NDEL1* | 0.695 | 2.60E-02 |
| NM_212869 | Dr.30396 | *trib3* | *TRIB3* | 0.693 | 2.99E-02 |
| NM_001024395 | Dr.79788 | *derl2* | *DERL2* | 0.689 | 4.58E-02 |
| NM_205605 | Dr.18243 | *wdr91* | *WDR91* | 0.687 | 3.10E-02 |
| NM_001076667 | Dr.38804 | *dync1i2a* | *DYNC1I1* | 0.683 | 2.28E-02 |
| NM_001002373 | Dr.85466 | *galm* | *GALM* | 0.679 | 4.57E-02 |
| NM_001017660 | Dr.13267 | *histh1l* | *HISTH1L* | 0.674 | 2.31E-02 |
| NM_200499 | Dr.26720 | *nae1* | *NAE1* | 0.670 | 4.02E-02 |
| NM_001077734 | Dr.76652 | *zgc:153012* | *N.A.* | 0.670 | 4.39E-02 |
| NM_001014819 | Dr.83220 | *nbn* | *NBN* | 0.670 | 4.97E-02 |
| NM_199550 | Dr.77236 | *naa35* | *NAA35* | 0.665 | 2.48E-02 |
| NM_001126404 | Dr.148312 | *LOC562468* | *ARHGAP25* | 0.663 | 2.42E-02 |
| NM_212777 | Dr.80311 | *brd8* | *BRD8* | 0.662 | 2.78E-02 |
| NM_131885 | Dr.78568 | *cebpa* | *CEBPA* | 0.656 | 3.83E-02 |
| NM_212763 | Dr.6507 | *mpzl2* | *MPZL2* | 0.653 | 4.17E-02 |
| NM_001110480 | Dr.66947 | *klhl8* | *KLHL8* | 0.647 | 3.25E-02 |
| NM_212873 | Dr.106930 | *lgals3bpb* | *LGALS3BP* | 0.643 | 2.01E-02 |
| NM_198357 | Dr.3018 | *tcerg1a* | *TCERG1* | 0.640 | 4.84E-02 |
| NM_001025504 | Dr.48719 | *ptgs2b* | *PTGS2* | 0.619 | 4.74E-02 |
| NM_001037115 | Dr.85323 | *apooa* | *APOO* | 0.618 | 4.09E-02 |
| NM_200508 | Dr.132598 | *eftud2* | *EFTUD2* | 0.618 | 4.35E-02 |
| NM_205661 | Dr.83905 | *zgc:77817* | *N.A.* | 0.617 | 4.75E-02 |
| NM_001079969 | Dr.85674 | *spoplb* | *SPOPL* | 0.613 | 4.81E-02 |
| NM_200342 | Dr.114603 | *ube2nb* | *UBE2NB* | 0.613 | 4.74E-02 |
| NM_213077 | Dr.77703 | *uhrf1* | *UHRF1* | 0.612 | 4.80E-02 |
| NM_001037667 | Dr.75286 | *ckap5* | *CKAP5* | 0.611 | 2.72E-02 |
| NM_001017831 | Dr.77711 | *zgc:110188* | *GM2A* | 0.607 | 4.32E-02 |
| NM_199730 | Dr.79871 | *dgat1a* | *DGAT1* | 0.606 | 4.81E-02 |
| NM_001089541 | Dr.133147 | *cyp4f3* | *CYP4F2* | 0.603 | 2.81E-02 |
| NM_001045006 | Dr.82226 | *hcls1* | *HCLS1* | 0.595 | 3.94E-02 |
| NM_001003497 | Dr.77249 | *gltpd1* | *GLTPD1* | 0.594 | 4.64E-02 |
| NM_001030167 | Dr.48434 | *nup107* | *NUP107* | 0.593 | 3.54E-02 |
| NM_213339 | Dr.80819 | *ppt1* | *PPT1* | 0.588 | 4.38E-02 |
| NM_001243323 | Dr.12252 | *selp* | *SELP* | 0.584 | 4.37E-02 |
| NM_001003473 | Dr.77924 | *zgc:91844* | *GPALPP1* | 0.583 | 4.46E-02 |
| NM_001128415 | Dr.26703 | *erlin2* | *ERLIN2* | 0.582 | 4.25E-02 |
| NM_200948 | Dr.25959 | *psmd13* | *PSMD13* | 0.579 | 4.17E-02 |
| NM_001080653 | Dr.2410 | *bckdhb* | *BCKDHB* | 0.574 | 4.20E-02 |
| NM_001089404 | Dr.106159 | *zgc:162730* | *N.A.* | 0.574 | 3.91E-02 |
| NM_001002715 | Dr.19030 | *zgc:92360* | *ADAP1* | 0.573 | 4.73E-02 |
| NM_001039924 | Dr.42344 | *graspl* | *GRASP* | 0.572 | 4.21E-02 |
| NM_199714 | Dr.1798 | *rraga* | *RRAGB* | 0.570 | 4.95E-02 |
| NM_213076 | Dr.24528 | *rps6kb1b* | *RPS6KB1* | 0.569 | 3.45E-02 |
| NM_001083833 | Dr.152514 | *zgc:162148* | *MICU1* | 0.569 | 4.70E-02 |
| NM_001044848 | Dr.81327 | *arhgef1b* | *LIPE* | 0.568 | 3.70E-02 |
| NM_001002676 | Dr.75512 | *bcat2* | *BCAT1* | 0.559 | 4.82E-02 |
| NM_212593 | Dr.76803 | *kiaa0907* | *KIAA0907* | 0.553 | 4.76E-02 |
| NM_212621 | Dr.77112 | *uap1* | *UAP1L1* | 0.552 | 4.61E-02 |
| NM_001037388 | Dr.82169 | *zgc:123333* | *NAGA* | 0.545 | 3.58E-02 |
| NM_198980 | Dr.83115 | *ldlrap1a* | *LDLRAP1* | 0.544 | 4.53E-02 |
| NM_199663 | Dr.10904 | *add3a* | *ADD3* | 0.541 | 3.48E-02 |
| NM_001004601 | Dr.76746 | *nucks1a* | *NUCKS1* | 0.541 | 4.46E-02 |
| NM_001001944 | Dr.10637 | *xrn2* | *XRN2* | 0.468 | 2.35E-02 |
| NM_213397 | Dr.78868 | *pfkfb3* | *PFKFB3* | 0.465 | 2.13E-02 |
| NM_173252 | Dr.76122 | *snrpd1* | *SNRPD1* | 0.440 | 1.93E-02 |
| NM_001083812 | Dr.77474 | *ell2* | *ELL2* | 0.435 | 1.11E-03 |
| NM_001089437 | Dr.29120 | *hadhab* | *MFAP5* | 0.420 | 3.82E-02 |
| NM_001145555 | Dr.73 | *rrbp1a* | *RRBP1* | 0.405 | 3.60E-02 |
| NM_001007133 | Dr.9145 | *zgc:152791* | *N.A.* | 0.390 | 6.08E-05 |
| NM_001045485 | Dr.48536 | *pi4k2b* | *PI4K2B* | 0.387 | 4.44E-02 |
| NM_001037117 | Dr.86778 | *zgc:123218* | *N.A.* | 0.380 | 2.01E-02 |
| NM_001177936 | Dr.143290 | *si:dkey-25o1.3* | *N.A.* | -0.370 | 3.99E-02 |
| NM_001002569 | Dr.162158 | *zgc:109981* | *TPMT* | -0.395 | 3.48E-02 |
| NM_213278 | Dr.32607 | *sfpq* | *SFPQ* | -0.421 | 4.01E-02 |
| NM_153669 | Dr.35540 | *tram1* | *TRAM1* | -0.446 | 2.32E-02 |
| NM_199886 | Dr.75684 | *ela2l* | *CELA2A* | -0.473 | 4.33E-02 |
| NM_001099420 | Dr.88698 | *ptpn22* | *PTPN22* | -0.542 | 3.36E-02 |
| NM_131452 | Dr.75059 | *elavl1* | *ELAVL1* | -0.554 | 4.92E-02 |
| NM_131390 | Dr.75529 | *gsk3ab* | *GSK3B* | -0.556 | 4.26E-02 |
| NM_213444 | Dr.1920 | *derl1* | *DERL1* | -0.569 | 3.42E-02 |
| NM_198806 | Dr.1388 | *gspt1l* | *GSPT2* | -0.581 | 3.76E-02 |
| NM_001077537 | Dr.84256 | *clcn7* | *CLCN7* | -0.587 | 3.77E-02 |
| NM_199697 | Dr.78599 | *gtf2b* | *GTF2B* | -0.589 | 3.18E-02 |
| NM_173237 | Dr.18323 | *adssl* | *ADSS* | -0.591 | 4.72E-02 |
| NM_001009892 | Dr.82062 | *serpinb1l1* | *SERPINB6* | -0.600 | 4.56E-02 |
| NM_001145604 | Dr.104627 | *kpnb3* | *RANBP6* | -0.607 | 3.43E-02 |
| NM_212808 | Dr.132507 | *zgc:85696* | *N.A.* | -0.609 | 4.35E-02 |
| NM_200266 | Dr.15571 | *sybl1* | *VAMP7* | -0.617 | 4.33E-02 |
| NM_001080013 | Dr.76423 | *adnp2b* | *ADNP2* | -0.617 | 4.43E-02 |
| NM_001202492 | Dr.74777 | *si:dkey-11p10.10* | *SH2D1B* | -0.622 | 4.79E-02 |
| NM_001110462 | Dr.66415 | *ncf2* | *NCF2* | -0.639 | 2.53E-02 |
| NM_001037390 | Dr.92721 | *fut7* | *FUT7* | -0.641 | 4.22E-02 |
| NM_212690 | Dr.4036 | *plaa* | *PLAA* | -0.644 | 3.60E-02 |
| NM_001033751 | Dr.115449 | *zgc:112435* | *FAM210B* | -0.649 | 4.61E-02 |
| NM_001033108 | Dr.76529 | *tmed9* | *TMED9* | -0.649 | 3.17E-02 |
| NM_213051 | Dr.76901 | *abce1* | *ABCE1* | -0.649 | 3.11E-02 |
| NM_001123289 | Dr.116234 | *si:dkey-56i24.1* | *N.A.* | -0.652 | 4.16E-02 |
| NM_001089479 | Dr.118315 | *hspa13* | *HSPA13* | -0.655 | 3.98E-02 |
| NM_001089525 | Dr.5554 | *ide* | *IDE* | -0.656 | 3.35E-02 |
| NM_001004116 | Dr.80294 | *elf2a* | *ELF2A* | -0.657 | 4.34E-05 |
| NM_001020614 | Dr.15106 | *eri1* | *ERI1* | -0.658 | 3.33E-02 |
| NM_001040348 | Dr.77273 | *rab40c* | *RAB40C* | -0.662 | 4.78E-02 |
| NM_212641 | Dr.76539 | *pa2g4b* | *PA2G4* | -0.675 | 1.90E-02 |
| NM_213448 | Dr.32760 | *canx* | *CANX* | -0.682 | 2.59E-02 |
| NM_001045295 | Dr.80095 | *farsa* | *FARSA* | -0.682 | 4.27E-02 |
| NM_001077603 | Dr.33396 | *atf7ip* | *ATF7IP* | -0.683 | 3.23E-02 |
| NM_001030239 | Dr.46539 | *zgc:112356* | *MAF1* | -0.685 | 1.81E-02 |
| NM_199563 | Dr.2393 | *dohh* | *DOHH* | -0.686 | 4.73E-02 |
| NM_131374 | Dr.76266 | *psme2* | *PSME2* | -0.690 | 4.19E-02 |
| NM_001037702 | Dr.77142 | *kpnb1* | *KPNB1* | -0.692 | 2.93E-02 |
| NM_175082 | Dr.52521 | *smad7* | *SMAD7* | -0.693 | 4.45E-02 |
| NM_200093 | Dr.27136 | *ormdl1* | *ORMDL1* | -0.696 | 3.47E-02 |
| NM_131834 | Dr.75485 | *cxcr4b* | *CXCR4* | -0.697 | 2.23E-02 |
| NM_201498 | Dr.76989 | *abat* | *ABAT* | -0.703 | 4.43E-02 |
| NM_199994 | Dr.11529 | *tcea1* | *TCEA1* | -0.709 | 3.83E-02 |
| NM_201459 | Dr.77264 | *arcn1a* | *ARCN1* | -0.711 | 2.37E-02 |
| NM_001006000 | Dr.74237 | *nutf2* | *NUTF2* | -0.713 | 3.88E-02 |
| NM_001044799 | Dr.81385 | *stim1a* | *STIM1* | -0.718 | 4.81E-02 |
| NM_001004583 | Dr.36537 | *slc35b1* | *SLC35B1* | -0.721 | 4.51E-02 |
| NM_001114570 | Dr.119979 | *ch25hl2* | *CH25HL2* | -0.723 | 3.04E-02 |
| NM_001089378 | Dr.84032 | *si:ch211-117l16.1* | *TMEM63A* | -0.731 | 3.10E-02 |
| NM_001256248 | Dr.86053 | *tbccl* | *TBCC* | -0.739 | 4.43E-02 |
| NM_201090 | Dr.81062 | *ppm1da* | *PPM1D* | -0.739 | 4.98E-02 |
| NM_001008609 | Dr.26140 | *armc1* | *ARMC1* | -0.741 | 4.19E-02 |
| NM_001004123 | Dr.116180 | *grid2* | *GRID2* | -0.744 | 3.29E-02 |
| NM_001110476 | Dr.41899 | *tesk2* | *TESK2* | -0.745 | 4.98E-02 |
| NM_200977 | Dr.84840 | *sigmar1* | *SIGMAR1* | -0.745 | 3.99E-02 |
| NM_001004001 | Dr.34238 | *zgc:101016* | *C9orf85* | -0.751 | 4.75E-02 |
| NM_001245988 | Dr.133813 | *LOC566907* | *N.A.* | -0.751 | 4.01E-02 |
| NM_001083570 | Dr.132661 | *errfi1* | *ERRFI1* | -0.753 | 4.91E-02 |
| NM_001201558 | Dr.8471 | *wu:fb39e12* | *MKL1* | -0.756 | 2.91E-02 |
| NM_001033111 | Dr.159286 | *pygo2* | *PYGO2* | -0.761 | 4.69E-02 |
| NM_001123008 | Dr.79454 | *zgc:158220* | *SPSB3* | -0.763 | 3.40E-02 |
| NM_001013544 | Dr.78636 | *pgap2* | *PGAP2* | -0.763 | 3.65E-02 |
| NM_001017853 | Dr.27897 | *cbx7a* | *CBX7A* | -0.768 | 3.30E-02 |
| NM_001083849 | Dr.77756 | *zgc:161969* | *N.A.* | -0.769 | 4.48E-02 |
| NM_001109718 | Dr.113248 | *mhc1ze* | *HLA-A* | -0.769 | 4.11E-02 |
| NM_001083080 | Dr.22573 | *si:dkeyp-59a8.1* | *N.A.* | -0.770 | 4.98E-02 |
| NM_131371 | Dr.81275 | *mtmr2* | *MTMR2* | -0.771 | 3.00E-02 |
| NM_212790 | Dr.76875 | *ypel3* | *YPEL3* | -0.772 | 4.94E-02 |
| NM_213173 | Dr.76246 | *sqstm1* | *TMSB4X* | -0.775 | 3.57E-02 |
| NM_131867 | Dr.12595 | *hsf2* | *HSF2* | -0.776 | 2.27E-02 |
| NM_001044316 | Dr.117435 | *snx11* | *SNX11* | -0.777 | 4.86E-02 |
| NM_001039719 | Dr.75870 | *ftr67* | *FTR67* | -0.778 | 3.71E-02 |
| NM_001110380 | Dr.18950 | *acp6* | *ACP6* | -0.778 | 2.19E-02 |
| NM_001190465 | Dr.117215 | *ifit5* | *IFIT5* | -0.780 | 4.91E-02 |
| NM_001002639 | Dr.10109 | *zgc:92202* | *N.A.* | -0.783 | 1.94E-02 |
| NM_001017547 | Dr.80770 | *glb1* | *GLB1* | -0.785 | 2.05E-02 |
| NM_001080992 | Dr.89941 | *si:dkey-231l1.6* | *ANKIB1* | -0.787 | 4.32E-02 |
| NM_001163298 | Dr.78761 | *msl2s* | *MSL2* | -0.787 | 2.23E-02 |
| NM_001256652 | Dr.75138 | *nolc1* | *NOLC1* | -0.788 | 3.24E-02 |
| NM_213011 | Dr.77109 | *amy2a* | *AMY2B* | -0.788 | 3.09E-02 |
| NM_001002623 | Dr.84875 | *zgc:92249* | *RARRES3* | -0.789 | 3.92E-02 |
| NM_200691 | Dr.75617 | *tuba8l2* | *TUBA4A* | -0.792 | 2.85E-02 |
| NM_213411 | Dr.79972 | *golga5* | *GOLGA5* | -0.792 | 3.94E-02 |
| NM_001017561 | Dr.80173 | *zgc:110567* | *TOR4A* | -0.795 | 4.88E-02 |
| NM_200596 | Dr.116470 | *tmem30c* | *TMEM30C* | -0.796 | 4.91E-02 |
| NM_001076710 | Dr.81338 | *zgc:152891* | *ALOX15B* | -0.797 | 4.98E-02 |
| NM_001030251 | Dr.79427 | *zgc:114103* | *TPRA1* | -0.797 | 3.98E-02 |
| NM_001115057 | Dr.77211 | *zgc:174906* | *N.A.* | -0.798 | 4.96E-02 |
| NM_001004607 | Dr.26104 | *zgc:92599* | *PXMP2* | -0.801 | 4.96E-02 |
| NM_001007354 | Dr.85073 | *eif4ebp3* | *EIF4EBP3* | -0.804 | 4.44E-02 |
| NM_001252649 | Dr.76461 | *si:ch211-117m20.5* | *SI:CH211-117M20.5* | -0.806 | 1.02E-02 |
| NM_213031 | Dr.76269 | *gadd45ba* | *GADD45B* | -0.807 | 3.93E-02 |
| NM_001004114 | Dr.2818 | *phf20b* | *PHF20* | -0.809 | 1.26E-02 |
| NM_001002646 | Dr.88169 | *ada* | *ADA* | -0.810 | 3.77E-02 |
| NM_001166238 | Dr.76962 | *psmd11a* | *PSMD11* | -0.810 | 9.96E-03 |
| NM_001080702 | Dr.91506 | *zgc:158643* | *NAPB* | -0.812 | 4.81E-02 |
| NM_200520 | Dr.108160 | *zgc:66337* | *LBH* | -0.812 | 4.15E-02 |
| NM_001076615 | Dr.77452 | *ctdsplb* | *CTDSPL* | -0.816 | 3.15E-02 |
| NM_200080 | Dr.75900 | *krt8* | *KRT8* | -0.817 | 2.88E-02 |
| NM_131710 | Dr.19238 | *ctsd* | *CTSD* | -0.817 | 1.99E-02 |
| NM_205565 | Dr.47431 | *zgc:77151* | *N.A.* | -0.819 | 4.09E-02 |
| NM_001003876 | Dr.79779 | *ddx56* | *DDX56* | -0.819 | 3.08E-02 |
| NM_001258235 | Dr.118519 | *dicp3.1* | *DICP3.1* | -0.822 | 4.63E-02 |
| NM_131613 | Dr.75890 | *celf1* | *CELF1* | -0.825 | 3.04E-02 |
| NM_200843 | Dr.9342 | *rabgef1* | *RABGEF1* | -0.827 | 1.31E-02 |
| NM_213482 | Dr.3614 | *abcd3a* | *ABCD3* | -0.833 | 2.55E-02 |
| NM_213536 | Dr.32781 | *zfp161* | *ZFP161* | -0.833 | 2.71E-02 |
| NM_199931 | Dr.412 | *yif1a* | *YIF1B* | -0.836 | 4.19E-02 |
| NM_200996 | Dr.151875 | *pdk2* | *PDK2* | -0.838 | 4.24E-02 |
| NM_001122613 | Dr.88423 | *si:ch211-165d12.4* | *SI:CH211-165D12.4* | -0.840 | 3.30E-02 |
| NM_001004014 | Dr.76049 | *smad9* | *SMAD9* | -0.840 | 4.36E-02 |
| NM_001172678 | Dr.83373 | *cox7a1* | *COX7A1* | -0.841 | 4.12E-02 |
| NM_199271 | Dr.77201 | *cpa5* | *CPA1* | -0.844 | 2.35E-02 |
| NM_001020638 | Dr.76067 | *cnot6l* | *CNOT6L* | -0.846 | 3.21E-02 |
| NM_001077601 | Dr.141435 | *peli1b* | *PELI1* | -0.849 | 2.30E-02 |
| NM_200206 | Dr.81669 | *zgc:56194* | *CAST* | -0.849 | 3.99E-02 |
| NM_001080028 | Dr.101972 | *slc16a10* | *SLC16A10* | -0.849 | 3.97E-02 |
| NM_131840 | Dr.10712 | *tnfrsfa* | *TNFRSFA* | -0.852 | 3.75E-02 |
| NM_199544 | Dr.5116 | *ctdsp2* | *CTDSPL* | -0.855 | 1.79E-02 |
| NM_001014367 | Dr.135387 | *zgc:110783* | *MARC1* | -0.857 | 2.86E-02 |
| NM_001044790 | Dr.149232 | *march2* | *MARCH2* | -0.865 | 4.67E-03 |
| NM_001037703 | Dr.120145 | *cfhl1* | *CFH* | -0.868 | 3.46E-02 |
| NM_001193525 | Dr.75659 | *fam213a* | *FAM213A* | -0.868 | 3.84E-02 |
| NM_001190382 | Dr.151255 | *sell* | *SELL* | -0.869 | 3.53E-02 |
| NM_199528 | Dr.28593 | *rbp1a* | *RBP1A* | -0.869 | 3.94E-02 |
| NM_001194988 | Dr.122474 | *rltgr* | *RLTGR* | -0.870 | 2.26E-03 |
| NM_001013307 | Dr.38105 | *ppm1k* | *PPM1K* | -0.871 | 2.44E-02 |
| NM_001126480 | Dr.159497 | *LOC100006238* | *ALDH9A1* | -0.879 | 2.92E-02 |
| NM_001045381 | Dr.81083 | *pter* | *PTER* | -0.880 | 3.17E-02 |
| NM_214751 | Dr.37928 | *pck1* | *PCK1* | -0.883 | 1.98E-02 |
| NM_001039839 | Dr.89513 | *as3mt* | *AS3MT* | -0.885 | 3.14E-02 |
| NM_199872 | Dr.11569 | *osbpl2* | *OSBPL2* | -0.885 | 1.40E-02 |
| NM_001020609 | Dr.107924 | *trnau1ap* | *TRNAU1AP* | -0.886 | 2.97E-02 |
| NM_131081 | Dr.75081 | *cdh2* | *CDH2* | -0.886 | 1.96E-02 |
| NM_001008634 | Dr.37874 | *adoa* | *ADO* | -0.886 | 3.14E-02 |
| NM_131826 | Dr.32057 | *spry4* | *SPRY4* | -0.891 | 3.42E-02 |
| NM_001163106 | Dr.135603 | *chl1a* | *CHL1* | -0.892 | 1.88E-02 |
| NM_001003466 | Dr.14775 | *fam122b* | *FAM122A* | -0.892 | 3.11E-02 |
| NM_001115114 | Dr.35640 | *gapdh* | *GAPDH* | -0.893 | 3.59E-02 |
| NM_201310 | Dr.78612 | *fbxw11a* | *FBXW11* | -0.893 | 1.69E-02 |
| NM_001111244 | Dr.80441 | *zgc:171298* | *PPFIA1* | -0.897 | 3.55E-02 |
| NM_200734 | Dr.28218 | *bcl6a* | *BCL6* | -0.898 | 1.20E-02 |
| NM_001077143 | Dr.74223 | *nt5dc2* | *NT5DC2* | -0.900 | 2.20E-02 |
| NM_199845 | Dr.80559 | *gclm* | *GCLM* | -0.903 | 1.33E-02 |
| NM_001020687 | Dr.88062 | *b3galnt2* | *B3GALNT2* | -0.906 | 2.48E-02 |
| NM_212693 | Dr.52859 | *bnip4* | *BNIP4* | -0.907 | 3.38E-02 |
| NM_001110475 | Dr.103307 | *exoc8* | *EXOC8* | -0.908 | 2.11E-02 |
| NM_131266 | Dr.81307 | *cmyb* | *MYB* | -0.909 | 3.35E-02 |
| NM_213475 | Dr.75321 | *gtpbp1* | *GTPBP1* | -0.911 | 2.00E-02 |
| NM_131364 | Dr.75764 | *mdm2* | *MDM2* | -0.911 | 2.99E-03 |
| NM_205549 | Dr.78884 | *slc25a16* | *SLC25A16* | -0.913 | 3.31E-02 |
| NM_001020554 | Dr.83699 | *gsr* | *GSR* | -0.923 | 5.60E-03 |
| NM_213102 | Dr.75932 | *thap11* | *THAP11* | -0.923 | 3.14E-02 |
| NM_001020608 | Dr.18113 | *gdi1* | *GDI1* | -0.924 | 2.97E-02 |
| NM_200318 | Dr.82824 | *arl4ab* | *ARL4A* | -0.924 | 2.86E-02 |
| NM_213110 | Dr.76342 | *gmfb* | *GMFB* | -0.926 | 3.04E-02 |
| NM_200865 | Dr.82469 | *scp2a* | *SCP2* | -0.928 | 2.42E-02 |
| NM_200633 | Dr.27095 | *kcnk5b* | *KCNK5* | -0.933 | 3.03E-02 |
| NM_001003481 | Dr.16545 | *zgc:91811* | *TMEM60* | -0.935 | 3.06E-02 |
| NM_001128789 | Dr.115683 | *LOC794943* | *LOC794943* | -0.936 | 2.94E-02 |
| NM_001042775 | Dr.52856 | *abcg2a* | *ABCG2* | -0.940 | 1.67E-02 |
| NM_200169 | Dr.77995 | *zgc:55634* | *FAM59A* | -0.942 | 2.98E-02 |
| NM_213246 | Dr.75607 | *vdac3* | *VDAC2* | -0.945 | 2.50E-02 |
| NM_001047190 | Dr.81875 | *rdh14b* | *RDH14B* | -0.950 | 2.67E-02 |
| NM_212604 | Dr.76994 | *cth* | *CTH* | -0.952 | 2.89E-02 |
| NM_200186 | Dr.107323 | *zgc:56005* | *OSGIN2* | -0.953 | 2.31E-02 |
| NM_207068 | Dr.80045 | *ehhadh* | *EHHADH* | -0.958 | 2.80E-02 |
| NM_199869 | Dr.115835 | *insig1* | *INSIG1* | -0.959 | 2.42E-02 |
| NM_001030179 | Dr.158989 | *slc35e3* | *SLC35E3* | -0.961 | 2.80E-02 |
| NM_181559 | Dr.76758 | *glula* | *GLUL* | -0.964 | 1.56E-02 |
| NM_001199372 | Dr.82600 | *trac* | *TRAC* | -0.967 | 1.53E-02 |
| NM_001123326 | Dr.82327 | *zgc:153958* | *DALRD3* | -0.967 | 1.46E-02 |
| NM_001136254 | Dr.111383 | *zgc:195195* | *N.A.* | -0.973 | 2.36E-02 |
| NM_199606 | Dr.21116 | *lpar5a* | *LPAR5A* | -0.975 | 7.37E-03 |
| NM_001113656 | Dr.114964 | *si:dkey-58f10.10* | *N.A.* | -0.979 | 2.61E-02 |
| NM_001002458 | Dr.31557 | *dgat1b* | *DGAT1* | -0.984 | 1.94E-02 |
| NM_001002660 | Dr.32020 | *sesn1* | *SESN1* | -0.985 | 1.90E-02 |
| NM_001098265 | Dr.78930 | *adnp2a* | *ADNP2* | -0.986 | 1.73E-02 |
| NM_001130599 | Dr.93351 | *si:ch211-198d18.2* | *USP6NL* | -0.987 | 1.35E-03 |
| NM_199942 | Dr.10070 | *fbp1a* | *FBP2* | -0.988 | 1.89E-02 |
| NM_001003865 | Dr.31536 | *rcl1* | *RCL1* | -0.997 | 2.44E-02 |
| NM_201471 | Dr.104770 | *aldh9a1a* | *ALDH9A1A* | -0.998 | 1.50E-02 |
| NM_214767 | Dr.82326 | *ube2j1* | *UBE2J1* | -1.003 | 6.46E-03 |
| NM_001045357 | Dr.76124 | *cox5b2* | *COX5B2* | -1.006 | 2.34E-02 |
| NM_001113510 | Dr.124927 | *il21r* | *IL21R* | -1.007 | 1.80E-02 |
| NM_001003426 | Dr.34133 | *zgc:92745* | *CELA1* | -1.007 | 1.26E-02 |
| NM_001045315 | Dr.80837 | *zgc:136759* | *DGKA* | -1.010 | 1.93E-02 |
| NM_001143904 | Dr.117120 | *irf3* | *IRF3* | -1.016 | 2.20E-02 |
| NM_201173 | Dr.80049 | *cyp7a1a* | *CYP7A1* | -1.017 | 2.25E-02 |
| NM_001045219 | Dr.80965 | *si:ch211-107o23.1* | *N.A.* | -1.019 | 1.80E-02 |
| NM_131248 | Dr.144146 | *egr1* | *EGR1* | -1.024 | 2.19E-02 |
| NM_201464 | Dr.2636 | *impdh2* | *IMPDH1P11* | -1.026 | 9.64E-03 |
| NM_001111229 | Dr.115208 | *phospho2* | *PHOSPHO2* | -1.027 | 2.16E-02 |
| NM_001037557 | Dr.26098 | *zgc:123007* | *AGXT2L2* | -1.027 | 1.59E-02 |
| NM_131111 | Dr.79808 | *lmo2* | *LMO2* | -1.030 | 1.96E-02 |
| NM_001089430 | Dr.89563 | *cxcr3.1* | *CXCR3* | -1.037 | 1.30E-02 |
| NM_001007284 | Dr.151654 | *mxc* | *MX2* | -1.041 | 1.98E-02 |
| NM_001045060 | Dr.105075 | *zgc:162356* | *N.A.* | -1.042 | 2.04E-02 |
| NM_213122 | Dr.76585 | *sox4a* | *SOX4* | -1.055 | 9.15E-03 |
| NM_212723 | Dr.6680 | *mknk2a* | *MKNK2* | -1.058 | 1.79E-02 |
| NM_205692 | Dr.83239 | *scamp1* | *SCAMP1* | -1.060 | 9.40E-03 |
| NM_001145567 | Dr.53954 | *exosc1* | *EXOSC1* | -1.061 | 1.83E-02 |
| NM_001111147 | Dr.80362 | *hpx* | *HPX* | -1.069 | 3.42E-02 |
| NM_200461 | Dr.84970 | *zgc:63602* | *CYP46A1* | -1.069 | 7.67E-03 |
| NM_001002205 | Dr.32174 | *dhrs1* | *DHRS1* | -1.077 | 1.71E-02 |
| NM_152961 | Dr.6814 | *fabp3* | *FABP3* | -1.084 | 1.72E-02 |
| NM_001002215 | Dr.76590 | *zgc:92357* | *MGST1* | -1.091 | 1.47E-02 |
| NM_001122747 | Dr.52983 | *si:ch211-106a19.2* | *ATP6V0A2* | -1.096 | 7.14E-03 |
| NM_131849 | Dr.150302 | *adh5* | *ADH5* | -1.101 | 1.16E-02 |
| NM_001007328 | Dr.81655 | *klhl12* | *KLHL12* | -1.101 | 1.16E-02 |
| NM_001244716 | Dr.89951 | *ccr9a* | *CCR9* | -1.108 | 7.43E-03 |
| NM_001083840 | Dr.74016 | *smcr7a* | *MIEF1* | -1.115 | 1.02E-02 |
| NM_001076637 | Dr.121047 | *pmt* | *PMT* | -1.125 | 8.40E-04 |
| NM_001013526 | Dr.85151 | *c6ast3* | *C6AST3* | -1.125 | 1.34E-02 |
| NM_001013471 | Dr.81710 | *prdx1* | *PRDX1* | -1.127 | 6.85E-03 |
| NM_001017629 | Dr.83299 | *nmnat1* | *NMNAT1* | -1.128 | 3.00E-03 |
| NM_001012490 | Dr.83712 | *zgc:113313* | *CCDC106* | -1.134 | 1.32E-02 |
| NM_200261 | Dr.78071 | *klhdc10* | *KLHDC10* | -1.137 | 1.26E-02 |
| NM_001007309 | Dr.78429 | *ctdnep1a* | *CTDNEP1* | -1.137 | 2.45E-03 |
| NM_001170836 | Dr.118434 | *znf574* | *ZNF574* | -1.151 | 1.24E-02 |
| NM_001089418 | Dr.105157 | *tmem135* | *TMEM135* | -1.155 | 1.25E-02 |
| NM_199605 | Dr.77127 | *zgc:66382* | *PRSS2* | -1.185 | 1.55E-33 |
| NM_200973 | Dr.79670 | *gps2* | *GPS2* | -1.190 | 1.98E-03 |
| NM_001001815 | Dr.78546 | *igf2b* | *IGF2B* | -1.200 | 1.03E-02 |
| NM_001012312 | Dr.37072 | *gsna* | *GSN* | -1.201 | 7.13E-03 |
| NM_001020692 | Dr.151025 | *rpp14* | *RPP14* | -1.213 | 9.43E-03 |
| NM_199672 | Dr.24253 | *stk3* | *STK3* | -1.216 | 3.40E-04 |
| NM_131573 | Dr.159468 | *dap1b* | *DAP1B* | -1.223 | 5.13E-03 |
| NM_001123287 | Dr.83260 | *rabep1* | *RABEP1* | -1.230 | 1.50E-03 |
| NM_001098186 | Dr.48823 | *suv420h2* | *SUV420H2* | -1.233 | 6.86E-03 |
| NM_001130667 | Dr.132816 | *zgc:194125* | *FTMT* | -1.234 | 8.83E-03 |
| NM_001114559 | Dr.119909 | *serpine1* | *SERPINE1* | -1.240 | 5.70E-03 |
| NR_023334 | Dr.132835 | *wu:fj39h09* | *NHLRC3* | -1.241 | 8.22E-03 |
| NM_001003446 | Dr.77138 | *cpa2* | *CPA5* | -1.242 | 3.73E-03 |
| NM_001030225 | Dr.48488 | *dse* | *DSE* | -1.247 | 3.66E-03 |
| NM_199215 | Dr.77009 | *ca2* | *CA13* | -1.250 | 6.05E-03 |
| NM_001281994 |  |  | *PRSS2* | -1.256 | 2.32E-33 |
| NM_001144786 | Dr.79872 | *si:ch1073-126c3.2* | *SI:CH1073-126C3.2* | -1.258 | 7.48E-03 |
| NM_001037661 | Dr.105841 | *slc6a6b* | *SLC6A6* | -1.261 | 1.24E-03 |
| NM_212584 | Dr.104499 | *ctsl1a* | *CTSV* | -1.262 | 1.90E-02 |
| NM_001256719 | Dr.90214 | *dhrsx* | *DHRSX* | -1.263 | 7.67E-03 |
| NM_001004646 | Dr.82956 | *osbpl3a* | *OSBPL3* | -1.268 | 6.85E-03 |
| NM_182864 | Dr.52260 | *keap1a* | *KEAP1* | -1.277 | 1.66E-03 |
| NM_001077567 | Dr.80342 | *eif2ak1* | *EIF2AK1* | -1.283 | 7.05E-03 |
| NM_001017724 | Dr.29122 | *zgc:112160* | *CTRB1* | -1.286 | 1.30E-08 |
| NM_001258225 | Dr.135610 | *irig* | *IRIG* | -1.291 | 2.46E-03 |
| NM_001003533 | Dr.79182 | *bag3* | *BAG3* | -1.292 | 6.48E-03 |
| NM_200006 | Dr.81246 | *slc48a1b* | *SLC48A1* | -1.296 | 6.25E-03 |
| NM_001100105 | Dr.161867 | *rnasel2* | *Ang4* | -1.312 | 3.71E-03 |
| NM_152960 | Dr.104721 | *fabp10a* | *FABP10A* | -1.328 | 3.12E-02 |
| NM_199963 | Dr.79522 | *rogdi* | *ROGDI* | -1.334 | 1.12E-03 |
| NM_001109862 | Dr.161821 | *zgc:173573* | *ZNF729* | -1.336 | 5.00E-03 |
| NM_001256212 | Dr.148186 | *fxyd1* | *FXYD1* | -1.337 | 4.96E-03 |
| NM_200428 | Dr.14647 | *zgc:66313* | *AMY2A* | -1.342 | 8.52E-03 |
| NM_001122858 | Dr.77212 | *masp2* | *MASP2* | -1.342 | 3.45E-03 |
| NM_001082930 | Dr.114622 | *si:ch211-240l19.8* | *SI:CH211-240L19.8* | -1.345 | 4.47E-16 |
| NM_001128254 | Dr.116285 | *mfn2* | *MFN2* | -1.346 | 4.20E-03 |
| NM_001007336 | Dr.84075 | *mettl8* | *METTL8* | -1.348 | 2.93E-03 |
| NM_213276 | Dr.76876 | *zgc:85829* | *DDAH1* | -1.348 | 4.34E-03 |
| NM_200789 | Dr.78050 | *zgc:73324* | *N.A.* | -1.351 | 6.34E-04 |
| NM_198063 | Dr.3585 | *agt* | *AGT* | -1.367 | 4.59E-03 |
| NM_173223 | Dr.123802 | *sdc2* | *SDC2* | -1.374 | 1.07E-03 |
| NM_001037673 | Dr.78705 | *prg4b* | *PRG4B* | -1.374 | 4.46E-03 |
| NM_001005981 | Dr.80177 | *kng1* | *KNG1* | -1.385 | 1.43E-02 |
| NM_200551 | Dr.76274 | *pah* | *PAH* | -1.391 | 4.29E-02 |
| NM_200570 | Dr.18416 | *selenbp1* | *SELENBP1* | -1.409 | 2.92E-03 |
| NM_001044941 | Dr.78042 | *klf11a* | *KLF11* | -1.415 | 2.00E-03 |
| NM_001109854 | Dr.114377 | *zgc:173594* | *FTMT* | -1.418 | 2.67E-03 |
| NM_001114704 | Dr.114900 | *zgc:174260* | *SERPINA9* | -1.423 | 3.44E-03 |
| NM_201046 | Dr.26801 | *shmt1* | *SHMT1* | -1.428 | 3.00E-03 |
| NM_001083000 | Dr.75398 | *slc43a1b* | *SLC43A1* | -1.453 | 2.24E-03 |
| NM_001002331 | Dr.82558 | *agxta* | *AGXT* | -1.459 | 1.72E-03 |
| NM_001163313 | Dr.77262 | *msl2b* | *MSL2* | -1.460 | 6.37E-04 |
| NM_131708 | Dr.75547 | *try* | *TRY* | -1.461 | 2.74E-10 |
| NM_001128743 | Dr.85087 | *zgc:194314* | *N.A.* | -1.477 | 2.64E-03 |
| NM_001099426 | Dr.71947 | *si:busm1-266f07.2* | *N.A.* | -1.489 | 2.44E-03 |
| NM_001030168 | Dr.78641 | *enpp1* | *ENPP1* | -1.490 | 2.47E-03 |
| NM_212619 | Dr.27131 | *cd9a* | *CD9* | -1.492 | 2.40E-03 |
| NM_001045363 | Dr.92789 | *wnt9a* | *WNT9A* | -1.501 | 1.07E-03 |
| NM_201289 | Dr.77543 | *pklr* | *PKLR* | -1.508 | 2.24E-03 |
| NM_200334 | Dr.13140 | *hyi* | *HYI* | -1.519 | 2.09E-03 |
| NM_001044806 | Dr.103690 | *prkchb* | *PRKCH* | -1.519 | 1.29E-03 |
| NM_212893 | Dr.161770 | *dpydb* | *DPYD* | -1.531 | 1.84E-03 |
| NM_212770 | Dr.1128 | *cpn1* | *CPN1* | -1.532 | 1.78E-03 |
| NM_001200010 | Dr.104697 | *LOC557507* | *AIFM2* | -1.533 | 1.39E-03 |
| NM_001025173 | Dr.47389 | *cel.2* | *CEL* | -1.534 | 1.12E-03 |
| NM_001114435 | Dr.120358 | *sla2* | *SLA2* | -1.536 | 2.62E-04 |
| NM_001109705 | Dr.108155 | *zgc:173593* | *FTMT* | -1.539 | 1.40E-03 |
| NM_001113477 | Dr.162180 | *keap1b* | *KEAP1* | -1.551 | 1.99E-04 |
| NM_001077554 | Dr.2455 | *tat* | *TAT* | -1.572 | 1.51E-03 |
| NM_001002550 | Dr.81357 | *pir* | *PIR* | -1.592 | 1.25E-03 |
| NM_001079967 | Dr.83718 | *n4bp2* | *N4BP2* | -1.592 | 1.85E-04 |
| NM_001002658 | Dr.79291 | *frs2b* | *FRS2* | -1.599 | 1.10E-03 |
| NM_001198772 | Dr.115503 | *aldh1l1* | *ALDH1L1* | -1.607 | 3.62E-03 |
| NM_001007360 | Dr.91675 | *cetp* | *CETP* | -1.609 | 1.16E-03 |
| NM_001256723 | Dr.113783 | *c8b* | *C8B* | -1.668 | 5.44E-04 |
| NM_203460 | Dr.16130 | *adh8b* | *ADH8B* | -1.680 | 7.79E-04 |
| NM_001007406 | Dr.89586 | *xkrx* | *XKRX* | -1.681 | 2.15E-05 |
| NM_212815 | Dr.11393 | *akt2l* | *AKT2* | -1.699 | 7.37E-04 |
| NM_001114915 | Dr.79754 | *crp3* | *APCS* | -1.732 | 3.28E-02 |
| NM_152980 | Dr.118210 | *mst1* | *MST1* | -1.732 | 6.02E-04 |
| NM_213507 | Dr.75549 | *zgc:55420* | *FAXDC2* | -1.737 | 4.24E-04 |
| NM_130912 | Dr.1079 | *cat* | *CAT* | -1.749 | 5.22E-04 |
| NM_001024408 | Dr.77514 | *ela3l* | *ELA3L* | -1.753 | 4.12E-19 |
| NM_178297 | Dr.9483 | *sepp1a* | *SEPP1* | -1.769 | 4.67E-04 |
| NM_001113651 | Dr.92011 | *LOC100003911* | *N.A.* | -1.781 | 3.70E-04 |
| NM_001020531 | Dr.132802 | *zgc:109934* | *FTMT* | -1.787 | 3.89E-04 |
| NM_200172 | Dr.78260 | *mycb* | *MYC* | -1.797 | 4.14E-04 |
| NM_212640 | Dr.76752 | *slc1a3a* | *SLC1A3* | -1.871 | 1.53E-04 |
| NM_001034182 | Dr.77654 | *sid4* | *HSPG2* | -1.883 | 4.21E-02 |
| NM_001002332 | Dr.106461 | *uox* | *UOX* | -1.903 | 2.01E-04 |
| NM_001080010 | Dr.85614 | *serpinf2b* | *SERPINF2* | -1.911 | 3.10E-02 |
| NM_001040306 | Dr.79892 | *kmo* | *KMO* | -1.914 | 1.80E-04 |
| NM_001002378 | Dr.31771 | *zgc:92066* | *FTMT* | -1.921 | 2.41E-04 |
| NM_205643 | Dr.80068 | *pnp4b* | *PNP* | -1.980 | 1.97E-02 |
| NM_199218 | Dr.935 | *ahcy* | *VAPB* | -2.003 | 2.00E-02 |
| NM_213192 | Dr.132490 | *pck2* | *PCK1* | -2.058 | 6.87E-05 |
| NM_001024435 | Dr.51148 | *c9* | *C9* | -2.079 | 4.92E-02 |
| NM_213054 | Dr.77175 | *fgg* | *FGG* | -2.103 | 2.45E-02 |
| NM_001194989 | Dr.33271 | *fga* | *FGA* | -2.105 | 2.58E-02 |
| NM_001113659 | Dr.132816 | *zgc:194125* | *FTMT* | -2.107 | 4.73E-05 |
| NM_199607 | Dr.159118 | *cel.1* | *CEL* | -2.130 | 3.56E-05 |
| NM_001105595 | Dr.28451 | *gamt* | *GAMT* | -2.169 | 3.65E-02 |
| NM_001110021 | Dr.77173 | *cpb1* | *CPB1* | -2.177 | 7.08E-06 |
| NM_213162 | Dr.1970 | *agxtb* | *AGXT* | -2.178 | 1.82E-02 |
| NM_001007282 | Dr.76793 | *gpx4a* | *GPX4A* | -2.232 | 2.64E-02 |
| NM_001077758 | Dr.75688 | *serpina1* | *SERPINA1* | -2.238 | 3.80E-02 |
| NM_001025474 | Dr.77210 | *zgc:112368* | *CELA1* | -2.246 | 1.26E-05 |
| NM_001003737 | Dr.31902 | *zgc:92041* | *CELA1* | -2.260 | 3.11E-36 |
| NM_001130586 | Dr.104720 | *apoa2* | *APOA2* | -2.265 | 2.25E-02 |
| NM_199523 | Dr.77108 | *ucp1* | *UCP3* | -2.274 | 4.68E-02 |
| NM_001100020 | Dr.78847 | *si:ch211-140f21.1* | *N.A.* | -2.311 | 9.59E-06 |
| NM_194367 | Dr.76309 | *aldob* | *ALDOB* | -2.319 | 3.36E-02 |
| NM_001199728 | Dr.21117 | *apom* | *APOM* | -2.323 | 3.65E-02 |
| NM_001013261 | Dr.24233 | *fn1b* | *FN1* | -2.327 | 4.45E-02 |
| NM_212622 | Dr.29757 | *ahsg* | *AHSG* | -2.332 | 4.00E-02 |
| NM_201472 | Dr.77342 | *plg* | *PLG* | -2.435 | 2.03E-02 |
| NM_001002494 | Dr.86222 | *rergla* | *RERGL* | -2.441 | 3.26E-06 |
| NM_001139479 | Dr.75455 | *a2ml* | *A2M* | -2.485 | 2.65E-06 |
| NM_131887 | Dr.1280 | *cebpd* | *CEBPD* | -2.521 | 1.42E-06 |
| NM_205685 | Dr.105154 | *grhprb* | *GRHPRB* | -2.521 | 1.96E-06 |
| NM_200235 | Dr.134613 | *cyp2k22* | *Cyp2ac1* | -2.588 | 8.33E-07 |
| NM_001100056 | Dr.13436 | *LOC796447* | *C6orf58* | -2.594 | 2.03E-02 |
| NM_131338 | Dr.75096 | *cfb* | *CFB* | -2.599 | 3.41E-02 |
| NM_001100144 | Dr.3564 | *apoa1b* | *APOA1B* | -2.599 | 1.75E-02 |
| NM_131127 | Dr.75099 | *lpl* | *LPL* | -2.674 | 4.55E-05 |
| NM_201118 | Dr.76990 | *ambpl* | *AMBP* | -2.707 | 2.26E-02 |
| NM_212774 | Dr.8505 | *fgb* | *FGB* | -2.707 | 2.05E-02 |
| NM_001123285 | Dr.36011 | *si:dkeyp-110g5.2* | *SERPING1* | -2.716 | 1.27E-02 |
| NM_130920 | Dr.1400 | *rbp4* | *RBP4* | -2.731 | 2.46E-07 |
| NM_182863 | Dr.4199 | *serpinc1* | *SERPINC1* | -2.768 | 1.92E-02 |
| NM_001077252 | Dr.37700 | *ces2* | *CES2* | -2.800 | 2.36E-02 |
| NM_131068 | Dr.473 | *inhbb* | *INHBB* | -2.850 | 9.51E-08 |
| NM_131128 | Dr.75775 | *apoa1a* | *APOA1* | -2.861 | 3.33E-02 |
| NM_201167 | Dr.77434 | *hpda* | *HPD* | -2.873 | 2.11E-02 |
| NM_001015057 | Dr.75658 | *tfa* | *TF* | -2.882 | 1.03E-02 |
| NM_001037236 | Dr.88584 | *c3c* | *C3* | -2.889 | 1.76E-02 |
| NM_001020482 | Dr.4244 | *cpa1* | *CPA1* | -2.930 | 4.62E-08 |
| NM_213203 | Dr.162096 | *zgc:77778* | *N.A.* | -2.940 | 1.83E-05 |
| NM_213390 | Dr.26855 | *f2* | *F2* | -2.969 | 2.32E-02 |
| NM_173283 | Dr.76315 | *igfbp1a* | *IGFBP1* | -3.057 | 1.93E-02 |
| NM_200346 | Dr.18420 | *zgc:63938* | *DIABLO* | -3.060 | 3.24E-02 |
| NM_001030062 | Dr.76570 | *apobl* | *APOBL* | -3.108 | 1.68E-02 |
| NM_001079861 | Dr.77089 | *apoa4* | *APOA4* | -3.199 | 2.04E-02 |
| NM_131243 | Dr.21006 | *c3b* | *C3* | -3.537 | 1.19E-02 |
| NM_131242 | Dr.77174 | *c3a* | *C3* | -3.569 | 1.20E-02 |
| NM_001012480 | Dr.75610 | *bhmt* | *BHMT* | -4.338 | 2.43E-03 |

N.A. refers to not available.

**Table S4. DEGs in female adult TANs**

| Refseq | Unigene ID | Gene Symbol | Hs Gene Symbol | log2FC | pvalue |
| --- | --- | --- | --- | --- | --- |
| NM_001003519 | Dr.33963 | *pltp* | *PLTP* | 4.399 | 2.47E-31 |
| NM_001040310 | Dr.83709 | *f9b* | *F9* | 4.373 | 2.36E-32 |
| NM_001076741 | Dr.87157 | *zgc:153027* | *ESM1* | 4.284 | 5.04E-32 |
| NM_178437 | Dr.39128 | *krt18* | *KRT18* | 3.705 | 4.72E-44 |
| NM_001002653 | Dr.77198 | *serpinb1* | *SERPINB1* | 3.333 | 1.49E-31 |
| NM_001099232 | Dr.5434 | *zgc:165571* | *N.A.* | 3.217 | 2.46E-20 |
| NM_001110128 | Dr.89561 | *zgc:171500* | *N.A.* | 3.212 | 6.81E-16 |
| NM_198063 | Dr.3585 | *agt* | *AGT* | 3.130 | 9.51E-19 |
| NM_001007282 | Dr.76793 | *gpx4a* | *GPX4A* | 2.870 | 2.03E-17 |
| NM_001194989 | Dr.33271 | *fga* | *FGA* | 2.822 | 3.97E-16 |
| NM_152960 | Dr.104721 | *fabp10a* | *FABP10A* | 2.661 | 8.42E-17 |
| NM_001003744 | Dr.20193 | *kras* | *KRAS* | 2.619 | 1.74E-34 |
| NM_001040251 | Dr.58304 | *zgc:136872* | *N.A.* | 2.608 | 7.05E-13 |
| NM_001080010 | Dr.85614 | *serpinf2b* | *SERPINF2* | 2.353 | 6.28E-12 |
| NM_200514 | Dr.84923 | *lgals2b* | *LGALS1* | 2.308 | 1.32E-12 |
| NM_214707 | Dr.132844 | *zdhhc18b* | *ZDHHC18* | 2.253 | 1.77E-20 |
| NM_198801 | Dr.75931 | *ckmt1* | *CKMT1* | 2.213 | 3.14E-08 |
| NM_130971 | Dr.1831 | *lfng* | *LFNG* | 2.140 | 6.73E-08 |
| NM_001017750 | Dr.75118 | *acta1a* | *ACTA1* | 2.027 | 3.53E-07 |
| NM_001045127 | Dr.33839 | *si:dkey-44g23.5* | *FAM195A* | 1.976 | 8.32E-07 |
| NM_173283 | Dr.76315 | *igfbp1a* | *IGFBP1* | 1.940 | 2.53E-07 |
| NM_001256257 | Dr.39823 | *cideb* | *CIDEB* | 1.919 | 1.25E-06 |
| NM_213428 | Dr.50843 | *cd9b* | *CD9* | 1.887 | 1.12E-09 |
| NM_001105117 | Dr.85476 | *si:ch211-137i24.10* | *SI:CH211-137I24.10* | 1.850 | 3.94E-06 |
| NM_001005981 | Dr.80177 | *kng1* | *KNG1* | 1.848 | 2.39E-06 |
| NM_001033751 | Dr.115449 | *zgc:112435* | *FAM210B* | 1.821 | 5.46E-13 |
| NM_001077758 | Dr.75688 | *serpina1* | *SERPINA1* | 1.802 | 1.09E-06 |
| NM_001080161 | Dr.76232 | *s100v2* | *S100V2* | 1.789 | 7.57E-06 |
| NM_001020596 | Dr.78179 | *phlda2* | *PHLDA2* | 1.768 | 8.42E-06 |
| NM_213150 | Dr.75640 | *slc22a2* | *SLC22A2* | 1.749 | 3.90E-06 |
| NM_001114915 | Dr.79754 | *crp3* | *APCS* | 1.740 | 1.23E-05 |
| NM_001014320 | Dr.86354 | *ppp1r13l* | *PPP1R13L* | 1.727 | 1.63E-05 |
| NM_001002190 | Dr.76383 | *rgcc* | *RGCC* | 1.721 | 7.90E-06 |
| NM_001282050 | N.A. | *N.A.* | *N.A.* | 1.719 | 4.91E-06 |
| NM_001020674 | Dr.23036 | *gda* | *GDA* | 1.715 | 4.37E-06 |
| NM_001104936 | Dr.83082 | *alg2* | *ALG2* | 1.688 | 4.21E-11 |
| NM_199523 | Dr.77108 | *ucp1* | *UCP3* | 1.687 | 2.80E-06 |
| NM_001007400 | Dr.22212 | *cyp3c1l2* | *CYP3A7* | 1.658 | 2.47E-05 |
| NM_200408 | Dr.12508 | *zgc:64085* | *TSPAN1* | 1.635 | 4.55E-05 |
| NM_181761 | Dr.75997 | *anxa2a* | *ANXA2* | 1.629 | 1.19E-09 |
| NM_001024404 | Dr.78590 | *marcksa* | *MARCKS* | 1.627 | 4.64E-05 |
| NM_001020588 | Dr.48047 | *zgc:110377* | *ITIH3* | 1.609 | 9.74E-06 |
| NM_177984 | Dr.107376 | *lmo4a* | *LMO4* | 1.587 | 1.78E-05 |
| NM_001002651 | Dr.76343 | *limk2* | *LIMK2* | 1.586 | 6.60E-05 |
| NM_001080001 | Dr.87836 | *tuft1a* | *TUFT1* | 1.569 | 9.52E-06 |
| NM_131098 | Dr.23502 | *apoeb* | *APOE* | 1.552 | 2.10E-05 |
| NM_001144786 | Dr.79872 | *si:ch1073-126c3.2* | *SI:CH1073-126C3.2* | 1.544 | 1.14E-04 |
| NM_001100020 | Dr.78847 | *si:ch211-140f21.1* | *SI:CH211-140F21.1* | 1.541 | 1.09E-04 |
| NM_001002617 | Dr.88692 | *tspan33* | *TSPAN33* | 1.530 | 4.30E-05 |
| NM_200243 | Dr.4501 | *gde1* | *GDE1* | 1.525 | 8.38E-06 |
| NM_131189 | Dr.25162 | *dnmt1* | *DNMT1* | 1.524 | 1.72E-07 |
| NM_201503 | Dr.81475 | *mmp13a* | *MMP13* | 1.519 | 9.09E-12 |
| NM_001122619 | Dr.160046 | *sc:d217* | *SC:D217* | 1.519 | 2.80E-06 |
| NM_001013261 | Dr.24233 | *fn1b* | *FN1* | 1.502 | 9.58E-05 |
| NM_001013259 | Dr.150316 | *serpina1l* | *SERPINA1* | 1.501 | 1.37E-04 |
| NM_001004554 | Dr.77190 | *rimkla* | *RIMKLB* | 1.477 | 6.40E-05 |
| NM_001076656 | Dr.88985 | *zgc:153723* | *Sult5a1* | 1.475 | 1.92E-06 |
| NM_001005393 | Dr.151204 | *boc* | *BOC* | 1.464 | 1.88E-04 |
| NM_001017881 | Dr.81511 | *sqrdl* | *SQRDL* | 1.460 | 6.61E-05 |
| NM_199277 | Dr.80394 | *gclc* | *GCLC* | 1.442 | 6.37E-06 |
| NM_212893 | Dr.161770 | *dpydb* | *DPYD* | 1.440 | 3.30E-04 |
| NM_200600 | Dr.18537 | *traf3ip1* | *TRAF3IP1* | 1.437 | 2.11E-05 |
| NM_001078168 | Dr.86501 | *sult2st3* | *SULT2B1* | 1.436 | 1.14E-04 |
| NM_001034977 | Dr.48538 | *pi4kb* | *PI4KB* | 1.424 | 2.50E-07 |
| NM_213181 | Dr.119316 | *ptp4a3* | *PTP4A3* | 1.415 | 1.81E-04 |
| NM_001037235 | Dr.87531 | *itgb3a* | *ITGB3* | 1.390 | 4.10E-05 |
| NM_001136255 | Dr.114276 | *gch1* | *GCH1* | 1.388 | 4.41E-04 |
| NM_001080172 | Dr.87169 | *prhoxnb* | *PRHOXNB* | 1.386 | 3.35E-04 |
| NM_001024415 | Dr.86891 | *cbx8b* | *CBX8* | 1.378 | 2.19E-04 |
| NM_200405 | Dr.11978 | *hif1al* | *HIF3A* | 1.371 | 4.87E-08 |
| NM_001017833 | Dr.44230 | *ppa1* | *PPA1* | 1.371 | 1.19E-07 |
| NM_199798 | Dr.80392 | *rrad* | *RRAD* | 1.351 | 4.97E-04 |
| NM_199737 | Dr.150554 | *zgc:56622* | *N.A.* | 1.350 | 8.63E-05 |
| NM_131217 | Dr.348 | *rxrga* | *RXRG* | 1.339 | 5.78E-11 |
| NM_200789 | Dr.78050 | *zgc:73324* | *N.A.* | 1.332 | 2.47E-04 |
| NM_201307 | Dr.7294 | *ndel1b* | *NDEL1* | 1.325 | 1.75E-07 |
| NM_001014327 | Dr.79654 | *zgc:113255* | *N.A.* | 1.311 | 1.86E-07 |
| NM_173259 | Dr.75656 | *scarb2* | *SCARB2* | 1.306 | 4.20E-09 |
| NM_001025177 | Dr.75706 | *dusp12* | *DUSP12* | 1.304 | 5.23E-05 |
| NM_001126472 | Dr.21225 | *olfml2a* | *OLFML2A* | 1.304 | 9.68E-05 |
| NM_001006036 | Dr.86834 | *zgc:103438* | *N.A.* | 1.297 | 1.19E-03 |
| NM_001008617 | Dr.14525 | *ttc26* | *TTC26* | 1.291 | 1.09E-03 |
| NM_213141 | Dr.76394 | *zgc:64098* | *TMEM263* | 1.290 | 5.65E-07 |
| NM_001017873 | Dr.159430 | *fibinb* | *FIBINB* | 1.277 | 1.43E-03 |
| NM_001128743 | Dr.85087 | *zgc:194314* | *N.A.* | 1.274 | 1.48E-03 |
| NM_200488 | Dr.107346 | *zgc:63759* | *N.A.* | 1.271 | 1.52E-03 |
| NM_213468 | Dr.76207 | *aqp3a* | *AQP3* | 1.270 | 1.47E-03 |
| NM_001076660 | Dr.42037 | *foxred2* | *FOXRED2* | 1.268 | 3.60E-05 |
| NM_001113376 | Dr.93001 | *lepr* | *LEPR* | 1.260 | 9.05E-04 |
| NM_001020728 | Dr.45793 | *fahd1* | *FAHD1* | 1.259 | 1.53E-03 |
| NM_131664 | Dr.78549 | *lima1* | *LIMA1* | 1.253 | 7.59E-04 |
| NM_001077600 | Dr.80460 | *ppm1la* | *PPM1L* | 1.236 | 1.39E-03 |
| NM_198814 | Dr.83881 | *zhx2* | *ZHX2* | 1.232 | 7.12E-08 |
| NM_199628 | Dr.77486 | *cers5* | *CERS5* | 1.226 | 8.70E-08 |
| NM_001076617 | Dr.74191 | *cish* | *CISH* | 1.223 | 1.53E-04 |
| NM_001139479 | Dr.75455 | *a2ml* | *A2M* | 1.214 | 1.69E-03 |
| NM_131406 | Dr.193 | *raraa* | *RARA* | 1.213 | 1.28E-05 |
| NM_212774 | Dr.8505 | *fgb* | *FGB* | 1.211 | 2.14E-03 |
| NM_214819 | Dr.82666 | *zgc:85746* | *RCAN2* | 1.204 | 1.75E-03 |
| NM_201508 | Dr.23802 | *aldh9a1b* | *ALDH9A1B* | 1.202 | 2.70E-03 |
| NM_001172629 | Dr.76117 | *nr4a3* | *NR4A3* | 1.201 | 1.74E-07 |
| NM_131370 | Dr.813 | *acat2* | *ACAT2* | 1.186 | 2.75E-03 |
| NM_200782 | Dr.81455 | *kcnip3* | *KCNIP3* | 1.172 | 1.02E-04 |
| NM_001089582 | Dr.89681 | *slco2a1* | *SLCO2A1* | 1.162 | 3.59E-03 |
| NM_001037376 | Dr.79607 | *zgc:123304* | *TMEM55B* | 1.162 | 1.35E-03 |
| NM_001037236 | Dr.88584 | *c3c* | *C3* | 1.155 | 3.96E-03 |
| NM_001089386 | Dr.86238 | *gfer* | *GFER* | 1.147 | 3.33E-03 |
| NM_001003773 | Dr.106937 | *cdkl1* | *CDKL1* | 1.142 | 4.30E-03 |
| NM_001146014 | Dr.12380 | *sall1a* | *SALL1A* | 1.132 | 1.59E-04 |
| NM_001044813 | Dr.77404 | *tmcc3* | *TMCC3* | 1.127 | 7.46E-06 |
| NM_205646 | Dr.82524 | *zgc:77147* | *STARD10* | 1.125 | 2.88E-03 |
| NM_194384 | Dr.19223 | *aldocb* | *ALDOC* | 1.121 | 1.92E-04 |
| NM_001045347 | Dr.49036 | *zgc:153788* | *RAB43* | 1.116 | 3.92E-04 |
| NM_001014324 | Dr.86374 | *ippk* | *IPPK* | 1.114 | 9.48E-07 |
| NM_001024435 | Dr.51148 | *c9* | *C9* | 1.109 | 5.58E-03 |
| NM_001033586 | Dr.85410 | *zgc:101684* | *TPMT* | 1.109 | 8.18E-04 |
| NM_214686 | Dr.3583 | *sult6b1* | *SULT6B1* | 1.108 | 8.52E-04 |
| NM_213076 | Dr.24528 | *rps6kb1b* | *RPS6KB1* | 1.106 | 5.94E-06 |
| NM_001099739 | Dr.82396 | *acsl4l* | *ACSL4* | 1.103 | 4.06E-06 |
| NM_001076718 | Dr.88444 | *adam28* | *ADAM28* | 1.101 | 3.78E-04 |
| NM_001045564 | Dr.75860 | *psmb7* | *PSMB10* | 1.088 | 4.70E-05 |
| NM_201320 | Dr.78859 | *kpna3* | *KPNA3* | 1.081 | 6.66E-08 |
| NM_001013472 | Dr.76395 | *zgc:110340* | *C8orf4* | 1.074 | 4.04E-03 |
| NM_001100005 | Dr.51213 | *uhrf1bp1l* | *UHRF1BP1L* | 1.073 | 8.66E-04 |
| NM_199273 | Dr.132741 | *zgc:66433* | *N.A.* | 1.070 | 3.04E-03 |
| NM_200186 | Dr.107323 | *zgc:56005* | *OSGIN2* | 1.068 | 1.34E-04 |
| NM_001030262 | Dr.77358 | *plin2* | *PLIN2* | 1.064 | 2.48E-06 |
| NM_001017717 | Dr.43244 | *bbox1* | *BBOX1* | 1.060 | 5.99E-04 |
| NM_198876 | Dr.76667 | *b3gnt5a* | *MCF2L2* | 1.059 | 4.79E-05 |
| NM_001100074 | Dr.80855 | *bcl6ab* | *BCL6AB* | 1.046 | 5.41E-05 |
| NM_001130784 | Dr.77088 | *wu:fk11d03* | *WU:FK11D03* | 1.045 | 7.22E-04 |
| NM_201295 | Dr.76386 | *coro1ca* | *CORO1CA* | 1.045 | 3.65E-04 |
| NM_001123272 | Dr.151761 | *LOC564424* | *SOAT1* | 1.041 | 2.54E-05 |
| NM_212726 | Dr.77576 | *pgp* | *PGP* | 1.040 | 1.25E-04 |
| NM_201171 | Dr.132891 | *dusp4* | *DUSP4* | 1.039 | 9.46E-03 |
| NM_001077153 | Dr.76498 | *zfp36l1a* | *ZFP36L1* | 1.038 | 2.32E-06 |
| NM_199835 | Dr.1483 | *agfg1a* | *AGFG1A* | 1.035 | 6.63E-07 |
| NM_001007352 | Dr.88655 | *rnf24* | *RNF24* | 1.029 | 8.79E-04 |
| NM_213054 | Dr.77175 | *fgg* | *FGG* | 1.028 | 6.20E-03 |
| NM_001045055 | Dr.81141 | *si:ch211-132b12.7* | *N.A.* | 1.027 | 7.01E-04 |
| NM_131299 | Dr.1307 | *foxa3* | *FOXA3* | 1.027 | 6.92E-03 |
| NM_001102624 | Dr.135758 | *zgc:171965* | *N.A.* | 1.023 | 9.08E-03 |
| NM_201009 | Dr.25204 | *aacs* | *AACS* | 1.019 | 7.73E-04 |
| NM_001024223 | Dr.106042 | *mlx* | *MLX* | 1.018 | 5.36E-04 |
| NM_001082813 | Dr.134250 | *si:dkey-102f14.5* | *C16orf70* | 1.018 | 5.27E-03 |
| NM_213312 | Dr.12990 | *gng12a* | *GNG12* | 1.016 | 4.29E-04 |
| NM_001110374 | Dr.148688 | *zgc:171630* | *SERPINH1* | 1.008 | 6.06E-03 |
| NM_213469 | Dr.17858 | *exo1* | *EXO1* | 1.005 | 1.20E-02 |
| NM_213252 | Dr.9278 | *hk1* | *HK2* | 1.004 | 1.20E-03 |
| NM_001100144 | Dr.3564 | *apoa1b* | *APOA1B* | 0.993 | 1.28E-02 |
| NM_001102616 | Dr.105118 | *tdo2a* | *TDO2* | 0.991 | 1.21E-02 |
| NM_200233 | Dr.75612 | *hif1ab* | *HIF1AB* | 0.991 | 1.20E-06 |
| NM_213038 | Dr.76747 | *pik3r3a* | *PIK3R3A* | 0.991 | 1.24E-05 |
| NM_212640 | Dr.76752 | *slc1a3a* | *SLC1A3* | 0.989 | 4.87E-03 |
| NM_200051 | Dr.6223 | *c1galt1b* | *C1GALT1B* | 0.987 | 1.45E-04 |
| NM_200173 | Dr.82394 | *ccdc53* | *CCDC53* | 0.986 | 1.31E-03 |
| NM_001007200 | Dr.96224 | *ptpn4a* | *MRAS* | 0.984 | 1.99E-04 |
| NM_001256212 | Dr.148186 | *fxyd1* | *FXYD1* | 0.983 | 1.42E-02 |
| NM_001002331 | Dr.82558 | *agxta* | *AGXT* | 0.980 | 1.39E-02 |
| NM_001199728 | Dr.21117 | *apom* | *APOM* | 0.980 | 1.06E-02 |
| NM_201473 | Dr.77323 | *slc26a5* | *SLC26A3* | 0.980 | 4.64E-05 |
| NM_212855 | Dr.79857 | *nfe2l1a* | *NFE2L1* | 0.979 | 6.21E-04 |
| NM_001128797 | Dr.118194 | *lpar5b* | *LPAR5B* | 0.977 | 1.07E-05 |
| NM_001080638 | Dr.110551 | *slc25a15a* | *SLC25A15* | 0.973 | 7.77E-06 |
| NM_205659 | Dr.29783 | *cnksr1* | *CNKSR1* | 0.973 | 1.56E-04 |
| NM_001080178 | Dr.81232 | *crtac1a* | *CRTAC1* | 0.971 | 1.27E-02 |
| NM_212876 | Dr.24266 | *cyb5b* | *CYB5B* | 0.970 | 2.50E-04 |
| NM_199706 | Dr.79210 | *gpd1b* | *GPD1L* | 0.969 | 1.56E-02 |
| NM_199811 | Dr.76790 | *rpa1* | *RPA1* | 0.967 | 6.25E-05 |
| NM_001040036 | Dr.94487 | *adora2ab* | *ADORA2B* | 0.961 | 8.43E-03 |
| NM_001002093 | Dr.83963 | *tubd1* | *TUBD1* | 0.960 | 5.05E-05 |
| NM_001114584 | Dr.90487 | *zgc:172295* | *GK* | 0.960 | 1.17E-02 |
| NM_200311 | Dr.86827 | *ctage5* | *CTAGE5* | 0.960 | 3.45E-05 |
| NM_001002746 | Dr.76657 | *pcca* | *PCCA* | 0.959 | 2.27E-04 |
| NM_001024207 | Dr.76153 | *ugcg* | *UGCG* | 0.951 | 4.29E-05 |
| NM_200350 | Dr.86356 | *ero1l* | *ERO1LB* | 0.951 | 1.77E-02 |
| NM_201076 | Dr.133499 | *sh2d3cb* | *BCAR3* | 0.951 | 3.12E-03 |
| NM_212604 | Dr.76994 | *cth* | *CTH* | 0.946 | 1.50E-02 |
| NM_200589 | Dr.24063 | *abcc2* | *ABCC2* | 0.943 | 1.81E-02 |
| NM_001002687 | Dr.152639 | *zgc:91849* | *N.A.* | 0.942 | 8.40E-03 |
| NM_001201443 | Dr.78512 | *gmeb1* | *GMEB1* | 0.940 | 9.07E-05 |
| NM_213527 | Dr.132412 | *hiat1b* | *HIAT1* | 0.940 | 7.38E-05 |
| NM_131337 | Dr.77148 | *sox11b* | *SOX11* | 0.937 | 1.33E-02 |
| NM_001076648 | Dr.157101 | *smcr7b* | *SMCR7* | 0.935 | 2.90E-03 |
| NM_001004628 | Dr.88161 | *pnp5b* | *PNP* | 0.934 | 6.03E-05 |
| NM_131513 | Dr.121261 | *ccnb1* | *CCNB2* | 0.930 | 2.03E-02 |
| NM_001044859 | Dr.14195 | *si:ch211-15d5.5* | *ATPIF1* | 0.930 | 3.18E-03 |
| NM_212921 | Dr.81190 | *msrb2* | *MSRB2* | 0.926 | 1.59E-02 |
| NM_205762 | Dr.81750 | *traf4a* | *TRAF4* | 0.926 | 2.10E-02 |
| NM_001040406 | Dr.139146 | *numb* | *NUMB* | 0.925 | 6.33E-04 |
| NM_213123 | Dr.76275 | *mmp9* | *MMP9* | 0.925 | 1.08E-05 |
| NM_001013301 | Dr.83978 | *upp1* | *UPP1* | 0.923 | 1.42E-04 |
| NM_201180 | Dr.26674 | *gys1* | *GYS2* | 0.923 | 2.35E-03 |
| NM_199212 | Dr.78523 | *sgk1* | *SGK2* | 0.921 | 1.83E-04 |
| NM_214716 | Dr.78661 | *hspa4a* | *HSPA4* | 0.920 | 4.39E-03 |
| NM_200251 | Dr.13039 | *slc43a2a* | *SLC43A2A* | 0.917 | 2.21E-02 |
| NM_131807 | Dr.79623 | *bcl2l1* | *BCL2L1* | 0.917 | 8.54E-05 |
| NM_001080684 | Dr.90467 | *vhl* | *VHL* | 0.916 | 1.09E-02 |
| NM_001014345 | Dr.76887 | *cbsb* | *CBS* | 0.916 | 1.10E-05 |
| NM_001076665 | Dr.107584 | *zgc:154045* | *RAB21* | 0.915 | 3.22E-05 |
| NM_182880 | Dr.57073 | *serpind1* | *SERPIND1* | 0.914 | 2.25E-02 |
| NM_001044944 | Dr.78487 | *si:ch211-191i18.1* | *N.A.* | 0.911 | 2.79E-03 |
| NM_001161750 | Dr.16458 | *map3k5* | *MAP3K5* | 0.911 | 5.49E-04 |
| NM_181757 | Dr.75805 | *anxa5b* | *ANXA5* | 0.907 | 1.38E-02 |
| NM_213193 | Dr.132662 | *adob* | *ADO* | 0.906 | 8.25E-03 |
| NM_212955 | Dr.30382 | *esrra* | *ESRRA* | 0.906 | 1.03E-03 |
| NM_001003474 | Dr.105859 | *spsb4b* | *SPSB4* | 0.905 | 2.08E-04 |
| NM_001044865 | Dr.108026 | *chka* | *CHKA* | 0.905 | 1.18E-02 |
| NM_001003887 | Dr.16862 | *ticrr* | *C15orf42* | 0.905 | 2.31E-02 |
| NM_214816 | Dr.77355 | *tcf12* | *TCF12* | 0.904 | 7.46E-03 |
| NM_199764 | Dr.13180 | *uck2b* | *UCK2* | 0.902 | 1.31E-03 |
| NM_212886 | Dr.87100 | *pgm2* | *PGM2* | 0.901 | 2.18E-03 |
| NM_001020544 | Dr.41776 | *zgc:109985* | *WBP2* | 0.899 | 1.99E-03 |
| NM_001099259 | Dr.151937 | *rab8b* | *RAB8B* | 0.898 | 1.82E-04 |
| NM_199707 | Dr.150483 | *ell* | *ELL* | 0.893 | 4.66E-05 |
| NM_001130780 | Dr.94105 | *fam20a* | *FAM20A* | 0.891 | 2.62E-02 |
| NM_200772 | Dr.79691 | *wars* | *WARS* | 0.887 | 4.88E-03 |
| NM_001025506 | Dr.10050 | *adipor2* | *ADIPOR2* | 0.887 | 7.01E-05 |
| NM_001080064 | Dr.84668 | *ipmkb* | *IPMKB* | 0.880 | 5.58E-03 |
| NM_001081691 | Dr.23431 | *zgc:158742* | *SH3GL3* | 0.879 | 3.06E-04 |
| NM_001098485 | Dr.81377 | *pgm2l1* | *PGM2* | 0.878 | 4.24E-03 |
| NM_001029970 | Dr.90978 | *rab24* | *RAB24* | 0.876 | 4.11E-03 |
| NM_001105697 | Dr.115331 | *gpr84* | *GPR84* | 0.872 | 3.02E-05 |
| NM_001006032 | Dr.84635 | *tspo* | *TSPO* | 0.867 | 6.33E-03 |
| NM_131564 | Dr.79604 | *appa* | *APP* | 0.864 | 4.46E-03 |
| NM_001082983 | Dr.90037 | *tmem120a* | *TMEM120A* | 0.861 | 2.30E-04 |
| NM_001100006 | Dr.62003 | *snx18a* | *SNX18* | 0.860 | 1.48E-04 |
| NM_001042775 | Dr.52856 | *abcg2a* | *ABCG2* | 0.858 | 3.06E-02 |
| NM_001076555 | Dr.74180 | *ifrd1* | *IFRD1* | 0.854 | 2.53E-04 |
| NM_131774 | Dr.11764 | *anxa13* | *ANXA13* | 0.844 | 2.25E-02 |
| NM_199550 | Dr.77236 | *naa35* | *NAA35* | 0.842 | 5.38E-04 |
| NM_001017882 | Dr.79739 | *sar1a* | *SAR1B* | 0.841 | 3.14E-02 |
| NM_201486 | Dr.20376 | *clic4* | *CLIC1* | 0.841 | 2.84E-02 |
| NM_199481 | Dr.76134 | *ccng1* | *CCNG1* | 0.841 | 1.22E-04 |
| NM_213358 | Dr.79494 | *pi4k2a* | *PI4K2A* | 0.840 | 1.97E-02 |
| NM_199664 | Dr.76236 | *ube2d1* | *UBE2D1* | 0.839 | 1.07E-04 |
| NM_001003555 | Dr.120097 | *cenpp* | *CENPP* | 0.837 | 1.00E-02 |
| NM_001013284 | Dr.79360 | *mif4gdb* | *MIF4GD* | 0.835 | 2.32E-02 |
| NM_001002625 | Dr.108054 | *supt3h* | *SUPT3H* | 0.830 | 2.42E-02 |
| NM_212816 | Dr.76276 | *gnmt* | *GNMT* | 0.830 | 9.04E-04 |
| NM_200538 | Dr.28449 | *slc2a12* | *SLC2A12* | 0.830 | 1.22E-02 |
| NM_001001843 | Dr.15819 | *tph1b* | *TPH1* | 0.829 | 1.64E-02 |
| NM_001002182 | Dr.83427 | *zgc:91890* | *N.A.* | 0.829 | 7.77E-03 |
| NM_001003503 | Dr.84029 | *elac1* | *ELAC1* | 0.827 | 1.44E-02 |
| NM_200034 | Dr.76296 | *zgc:56419* | *N.A.* | 0.827 | 5.47E-04 |
| NM_001080587 | Dr.82221 | *dytn* | *DYTN* | 0.826 | 2.18E-02 |
| NM_001024445 | Dr.37774 | *agtpbp1* | *AGTPBP1* | 0.822 | 6.53E-03 |
| NM_199601 | Dr.44058 | *fah* | *FAH* | 0.821 | 1.24E-02 |
| NM_001030181 | Dr.80811 | *si:ch211-237l4.5* | *PCOLCE* | 0.821 | 1.56E-02 |
| NM_001044862 | Dr.12216 | *rasgrp4* | *RASGRP1* | 0.819 | 4.74E-04 |
| NM_001077564 | Dr.75277 | *foxp1a* | *FOXP1A* | 0.818 | 5.82E-03 |
| NM_001008630 | Dr.14023 | *zgc:101722* | *NXNL2* | 0.816 | 2.94E-02 |
| NM_001113641 | Dr.82576 | *si:ch211-170d8.5* | *N.A.* | 0.814 | 3.89E-02 |
| NM_001077287 | Dr.89219 | *afap1l1b* | *AFAP1L1* | 0.814 | 4.96E-03 |
| NM_001130785 | Dr.88548 | *zgc:195062* | *C2orf43* | 0.814 | 7.81E-03 |
| NM_182857 | Dr.159689 | *per2* | *PER2* | 0.814 | 2.26E-02 |
| NM_200008 | Dr.78272 | *ppdpfb* | *PPDPF* | 0.812 | 1.05E-02 |
| NM_200340 | Dr.80998 | *plekhf1* | *PLEKHF1* | 0.809 | 9.22E-03 |
| NM_200208 | Dr.117193 | *desi1b* | *DESI1B* | 0.808 | 2.29E-03 |
| NM_131134 | Dr.81286 | *fzd5* | *FZD5* | 0.806 | 4.42E-02 |
| NM_199610 | Dr.77134 | *dnajc3* | *DNAJC3* | 0.806 | 8.37E-03 |
| NM_200076 | Dr.77842 | *mars* | *MARS* | 0.805 | 1.27E-03 |
| NM_001271308 | N.A. | *N.A.* | *ACACA* | 0.804 | 1.18E-02 |
| NM_131787 | Dr.80555 | *cry4* | *CRY4* | 0.804 | 1.70E-02 |
| NM_001017667 | Dr.76666 | *zgc:112042* | *MFSD10* | 0.803 | 2.96E-03 |
| NM_201303 | Dr.76847 | *mapkapk2a* | *MAPKAPK2* | 0.801 | 2.19E-04 |
| NM_001110402 | Dr.78436 | *ugdh* | *UGDH* | 0.801 | 1.62E-02 |
| NM_131412 | Dr.1 | *myca* | *MYC* | 0.800 | 1.92E-02 |
| NM_001079994 | Dr.76563 | *gfm1* | *GFM1* | 0.798 | 1.05E-03 |
| NM_001077731 | Dr.81779 | *zgc:154093* | *N.A.* | 0.797 | 4.00E-02 |
| NM_001080799 | Dr.108294 | *si:ch211-244b2.1* | *SCYL2* | 0.797 | 8.93E-04 |
| NM_200258 | Dr.78389 | *zgc:55558* | *N.A.* | 0.795 | 2.67E-04 |
| NM_199792 | Dr.26610 | *znf710a* | *ZNF710* | 0.789 | 1.13E-02 |
| NM_200417 | Dr.87870 | *gpr39* | *LYPD1* | 0.787 | 4.94E-02 |
| NM_213390 | Dr.26855 | *f2* | *F2* | 0.786 | 4.60E-02 |
| NM_001017809 | Dr.84969 | *bdh2* | *BDH2* | 0.786 | 5.03E-03 |
| NM_001199366 | Dr.104840 | *cfhl4* | *CFHL4* | 0.785 | 3.96E-02 |
| NM_152961 | Dr.6814 | *fabp3* | *FABP3* | 0.781 | 2.87E-02 |
| NM_200677 | Dr.82280 | *irf7* | *IRF7* | 0.781 | 5.88E-04 |
| NM_001007432 | Dr.37150 | *galk2* | *GALK2* | 0.780 | 2.75E-02 |
| NM_001045315 | Dr.80837 | *zgc:136759* | *DGKA* | 0.778 | 2.74E-02 |
| NM_001017558 | Dr.98888 | *ets1a* | *ETS1* | 0.776 | 3.56E-04 |
| NM_199219 | Dr.2328 | *elf2b* | *ELF2B* | 0.774 | 2.27E-03 |
| NM_183341 | Dr.86414 | *egr2a* | *EGR2* | 0.774 | 2.42E-02 |
| NM_001039810 | Dr.49022 | *pold1* | *POLD1* | 0.774 | 4.80E-04 |
| NM_212896 | Dr.86893 | *zgc:77828* | *RFFL* | 0.774 | 1.15E-02 |
| NM_213058 | Dr.67791 | *hspa5* | *HSPA5* | 0.774 | 8.30E-03 |
| NM_001111175 | Dr.29913 | *zgc:171566* | *GGH* | 0.774 | 4.92E-02 |
| NM_001024393 | Dr.91282 | *zgc:110447* | *N.A.* | 0.773 | 6.58E-03 |
| NM_001020500 | Dr.88697 | *cybrd1* | *CYBRD1* | 0.773 | 7.73E-04 |
| NM_212772 | Dr.75912 | *tuba8l* | *TUBA3C* | 0.773 | 2.11E-04 |
| NM_198877 | Dr.75938 | *mibp2* | *MIBP2* | 0.772 | 7.56E-03 |
| NM_200547 | Dr.83952 | *purb* | *PURB* | 0.771 | 1.37E-02 |
| NM_001110412 | Dr.120351 | *ppp1r3da* | *PPP1R3D* | 0.770 | 3.95E-02 |
| NM_212689 | Dr.78922 | *lmo4b* | *LMO4* | 0.770 | 4.27E-03 |
| NM_001083026 | Dr.87642 | *rps6ka3b* | *RPS6KA3* | 0.768 | 3.36E-02 |
| NM_001002688 | Dr.32749 | *cdc34b* | *CDC34* | 0.767 | 1.70E-03 |
| NM_001123255 | Dr.76641 | *rnf10* | *RNF10* | 0.767 | 2.02E-04 |
| NM_212637 | Dr.75135 | *mat2aa* | *MAT1A* | 0.766 | 1.88E-04 |
| NM_214693 | Dr.32351 | *lgals3l* | *LGALS3L* | 0.764 | 1.73E-03 |
| NM_198804 | Dr.945 | *pgam1a* | *PGAM2* | 0.761 | 2.00E-02 |
| NM_001076629 | Dr.14931 | *manf* | *MANF* | 0.759 | 1.29E-02 |
| NM_001007363 | Dr.37364 | *dhrs13a.1* | *DHRS13* | 0.759 | 5.54E-03 |
| NM_213039 | Dr.3123 | *zfand5a* | *ZFAND5* | 0.758 | 2.02E-04 |
| NM_001100045 | Dr.36673 | *b3gnt5b* | *MCF2L2* | 0.757 | 5.08E-04 |
| NM_001172680 | Dr.78145 | *zgc:136826* | *RPS27L* | 0.756 | 3.83E-03 |
| NM_194377 | Dr.61171 | *aldoaa* | *ALDOC* | 0.754 | 1.13E-02 |
| NM_212904 | Dr.143624 | *chmp2bb* | *CHMP2B* | 0.754 | 6.65E-04 |
| NM_001039813 | Dr.141253 | *adora2b* | *ADORA2B* | 0.753 | 1.26E-03 |
| NM_199897 | Dr.106021 | *slc43a1a* | *SLC43A1* | 0.752 | 1.75E-02 |
| NM_001007303 | Dr.106742 | *zgc:92804* | *ARFGAP1* | 0.751 | 7.18E-03 |
| NM_001044928 | Dr.86837 | *ptpn23b* | *PTPN23* | 0.747 | 5.25E-03 |
| NM_131059 | Dr.10259 | *ctnnb1* | *CTNNB1* | 0.746 | 1.54E-02 |
| NM_001082825 | Dr.41190 | *ddit3* | *DDIT3* | 0.746 | 3.45E-04 |
| NM_198813 | Dr.82149 | *zhx3* | *ZHX3* | 0.744 | 2.71E-02 |
| NM_001082838 | Dr.40650 | *ubap2* | *UBAP2* | 0.739 | 2.90E-03 |
| NM_213360 | Dr.75510 | *pdcd6ip* | *PDCD6IP* | 0.739 | 1.68E-03 |
| NM_001105693 | Dr.115914 | *zgc:171682* | *NAGK* | 0.737 | 4.97E-02 |
| NM_001007375 | Dr.32436 | *si:dkey-42i9.4* | *N.A.* | 0.734 | 1.65E-03 |
| NM_001020824 | Dr.21703 | *zgc:110680* | *TXNDC12* | 0.733 | 3.59E-03 |
| NM_198140 | Dr.75674 | *tpt1* | *TPT1* | 0.733 | 2.50E-03 |
| NM_201587 | Dr.17431 | *ical1* | *ICA1L* | 0.731 | 5.26E-03 |
| NM_212756 | Dr.67270 | *grn2* | *GRN2* | 0.730 | 4.87E-03 |
| NM_001002178 | Dr.32183 | *zgc:91909* | *RAB7A* | 0.728 | 2.46E-03 |
| NM_200078 | Dr.75365 | *cited4b* | *CITED4B* | 0.728 | 3.63E-03 |
| NM_001007372 | Dr.80846 | *gsto2* | *GSTO1* | 0.726 | 4.67E-03 |
| NM_130924 | Dr.8290 | *ptpn1* | *PTPN1* | 0.725 | 7.83E-04 |
| NM_213246 | Dr.75607 | *vdac3* | *VDAC2* | 0.724 | 3.06E-02 |
| NM_194394 | Dr.26893 | *mcl1b* | *MCL1* | 0.724 | 6.74E-03 |
| NM_131585 | Dr.6496 | *fth1a* | *FTH1P3* | 0.719 | 3.65E-04 |
| NM_131246 | Dr.4212 | *ldha* | *LDHB* | 0.718 | 2.69E-02 |
| NM_001017629 | Dr.83299 | *nmnat1* | *NMNAT1* | 0.718 | 4.73E-02 |
| NM_001098766 | Dr.85599 | *zgc:165573* | *CYSTM1* | 0.717 | 1.10E-03 |
| NM_001077380 | Dr.87907 | *lrrc8db* | *LRRC8D* | 0.716 | 4.72E-02 |
| NM_001080600 | Dr.82412 | *shq1* | *SHQ1* | 0.716 | 3.41E-02 |
| NM_199794 | Dr.14259 | *memo1* | *MEMO1* | 0.716 | 1.98E-02 |
| NM_001003997 | Dr.76199 | *flad1* | *FLAD1* | 0.709 | 4.76E-02 |
| NM_213456 | Dr.18403 | *stk38a* | *STK38L* | 0.708 | 1.55E-03 |
| NM_200889 | Dr.77727 | *heatr3* | *HEATR3* | 0.707 | 3.93E-02 |
| NM_001122654 | Dr.75263 | *ptbp1b* | *PTBP1* | 0.705 | 3.65E-02 |
| NM_200035 | Dr.76297 | *ggps1* | *GGPS1* | 0.704 | 7.07E-03 |
| NM_001100054 | Dr.74501 | *metap2a* | *METAP2A* | 0.702 | 2.40E-02 |
| NM_001045552 | Dr.80740 | *mapk6* | *MAPK6* | 0.702 | 2.23E-02 |
| NM_001004288 | Dr.80637 | *itga5* | *ITGA5* | 0.700 | 4.64E-02 |
| NM_001003569 | Dr.80184 | *zgc:101071* | *ACSL1* | 0.700 | 1.51E-02 |
| NM_001037681 | Dr.91652 | *tcf21* | *TCF21* | 0.699 | 4.87E-02 |
| NM_001006052 | Dr.91108 | *tmem189* | *UBE2V1* | 0.699 | 1.69E-02 |
| NM_001003595 | Dr.81537 | *cant1b* | *CANT1B* | 0.699 | 3.34E-02 |
| NM_001007325 | Dr.118860 | *zgc:91976* | *SGMS1* | 0.698 | 1.22E-03 |
| NM_212647 | Dr.935 | *ahcy* | *VAPB* | 0.697 | 1.42E-03 |
| NM_131688 | Dr.10713 | *atp1a1a.3* | *ATP1A1A.3* | 0.696 | 2.92E-02 |
| NM_001077780 | Dr.42121 | *rb1* | *RB1* | 0.696 | 5.97E-03 |
| NM_001034981 | Dr.94310 | *gfpt1* | *GFPT2* | 0.696 | 2.09E-02 |
| NM_001110126 | Dr.76600 | *chac1* | *CHAC1* | 0.696 | 9.65E-04 |
| NM_200949 | Dr.6001 | *zfand5b* | *ZFAND5* | 0.694 | 2.57E-03 |
| NM_001017728 | Dr.90156 | *f3b* | *F3* | 0.693 | 4.00E-02 |
| NM_001003730 | Dr.51929 | *sdf2l1* | *SDF2L1* | 0.691 | 3.56E-02 |
| NM_213135 | Dr.5040 | *cyb5a* | *CYB5A* | 0.689 | 2.40E-03 |
| NM_001008583 | Dr.78424 | *cd2ap* | *CD2AP* | 0.689 | 4.20E-02 |
| NM_194395 | Dr.77093 | *birc2* | *BIRC3* | 0.687 | 3.91E-03 |
| NM_001014289 | Dr.29749 | *eif4g2a* | *EIF4G2* | 0.687 | 4.37E-03 |
| NM_212866 | Dr.16684 | *plscr3b* | *PLSCR1* | 0.686 | 9.93E-04 |
| NM_001037384 | Dr.5829 | *grpel1* | *GRPEL1* | 0.685 | 2.93E-02 |
| NM_200366 | Dr.79342 | *prelid1a* | *PRELID1* | 0.684 | 2.46E-02 |
| NM_199990 | Dr.76677 | *vps37a* | *VPS37A* | 0.683 | 5.39E-03 |
| NM_131176 | Dr.21244 | *ucp2* | *UCP2* | 0.681 | 1.79E-03 |
| NM_001002745 | Dr.102158 | *impa1* | *IMPA1* | 0.679 | 6.03E-03 |
| NM_001163293 | Dr.53262 | *snx25* | *SNX25* | 0.676 | 3.60E-03 |
| NM_001083014 | Dr.116844 | *zgc:158327* | *C14orf49* | 0.674 | 1.50E-02 |
| NM_200928 | Dr.12260 | *rab7* | *RAB7A* | 0.673 | 3.11E-03 |
| NM_001034983 | Dr.83706 | *aco1* | *ACO1* | 0.671 | 8.63E-03 |
| NM_001089568 | Dr.89004 | *zgc:162724* | *N.A.* | 0.671 | 7.05E-03 |
| NM_001002649 | Dr.79934 | *aclya* | *ACLY* | 0.670 | 6.29E-03 |
| NM_001030074 | Dr.407 | *kcnk6* | *KCNK6* | 0.670 | 3.10E-02 |
| NM_212708 | Dr.23391 | *slc16a3* | *SLC16A3* | 0.670 | 1.14E-02 |
| NM_001034982 | Dr.48619 | *por* | *POR* | 0.670 | 3.13E-03 |
| NM_001017763 | Dr.83238 | *zgc:112084* | *HEXA* | 0.668 | 5.27E-03 |
| NM_001025475 | Dr.79806 | *idi1* | *IDI2* | 0.668 | 4.14E-02 |
| NM_200978 | Dr.84921 | *prkcbb* | *PRKCB* | 0.667 | 1.64E-02 |
| NM_001278842 | N.A. | *N.A.* | *NFE2L1* | 0.667 | 3.23E-02 |
| NM_001195613 | Dr.6419 | *abcc5* | *ABCC5* | 0.664 | 4.04E-03 |
| NM_131364 | Dr.75764 | *mdm2* | *MDM2* | 0.664 | 1.00E-02 |
| NM_199608 | Dr.21056 | *dbi* | *DBI* | 0.660 | 3.32E-03 |
| NM_199824 | Dr.70549 | *arl8* | *ARL5B* | 0.657 | 3.13E-02 |
| NM_213639 | Dr.76190 | *cfl1* | *CFL1* | 0.657 | 9.14E-03 |
| NM_001256603 | Dr.31547 | *asph* | *ASPH* | 0.656 | 4.07E-02 |
| NM_201291 | Dr.32819 | *id2a* | *ID2* | 0.654 | 8.49E-03 |
| NM_001089535 | Dr.87479 | *ube2j2* | *UBE2J2* | 0.654 | 9.41E-03 |
| NM_198142 | Dr.2447 | *bsg* | *BSG* | 0.652 | 2.12E-02 |
| NM_199719 | Dr.78153 | *zgc:55512* | *UBE2A* | 0.652 | 7.35E-03 |
| NM_200196 | Dr.745 | *pdlim5b* | *PDLIM5* | 0.649 | 2.29E-03 |
| NM_212615 | Dr.4850 | *rnf128a* | *RNF128* | 0.649 | 4.99E-02 |
| NM_131518 | Dr.75855 | *cd81* | *CD81* | 0.646 | 7.20E-03 |
| NM_201340 | Dr.80462 | *ahcyl2* | *AHCYL2* | 0.645 | 4.17E-02 |
| NM_001017854 | Dr.77665 | *atg16l1* | *ATG16L1* | 0.645 | 3.50E-03 |
| NM_001128529 | Dr.7501 | *mepce* | *MEPCE* | 0.642 | 2.25E-02 |
| NM_001025537 | Dr.132634 | *herpud1* | *HERPUD1* | 0.641 | 3.61E-03 |
| NM_201163 | Dr.25168 | *asns* | *ASNS* | 0.640 | 9.92E-03 |
| NM_200160 | Dr.81636 | *zbtb2a* | *ZBTB2* | 0.637 | 6.88E-03 |
| NM_131347 | Dr.2166 | *urod* | *UROD* | 0.635 | 2.38E-02 |
| NM_001009898 | Dr.81568 | *cdk2ap2* | *CDK2AP2* | 0.634 | 8.15E-03 |
| NM_001114704 | Dr.114900 | *zgc:174260* | *SERPINA9* | 0.633 | 4.41E-02 |
| NM_001004583 | Dr.36537 | *slc35b1* | *SLC35B1* | 0.632 | 3.91E-02 |
| NM_001005293 | Dr.32139 | *ric8b* | *RIC8B* | 0.631 | 2.09E-02 |
| NM_200581 | Dr.77072 | *ammecr1* | *AMMECR1* | 0.630 | 1.40E-02 |
| NM_001079987 | Dr.27905 | *tmem41b* | *TMEM41B* | 0.629 | 2.40E-02 |
| NM_201507 | Dr.75913 | *mapk3* | *MAPK3* | 0.627 | 3.52E-03 |
| NM_214724 | Dr.84135 | *rgs12* | *RGS12* | 0.627 | 4.11E-02 |
| NM_200053 | Dr.297 | *ssr3* | *SSR3* | 0.626 | 2.16E-02 |
| NM_001044933 | Dr.74201 | *asap2b* | *ASAP2* | 0.625 | 9.46E-03 |
| NM_200904 | Dr.80315 | *fermt3b* | *FERMT3* | 0.623 | 2.02E-03 |
| NM_001277234 |  |  | *LONRF1* | 0.623 | 4.90E-03 |
| NM_131012 | Dr.659 | *gro2* | *GRO2* | 0.622 | 1.08E-02 |
| NM_001012259 | Dr.85427 | *xkr9* | *XKR9* | 0.622 | 2.81E-02 |
| NM_001030164 | Dr.89852 | *hivep2* | *HIVEP2* | 0.621 | 2.31E-02 |
| NM_213109 | Dr.24327 | *cmpk* | *CMPK1* | 0.621 | 7.10E-03 |
| NM_001045019 | Dr.73871 | *tiparp* | *TIPARP-AS1* | 0.620 | 1.83E-02 |
| NM_001020663 | Dr.91614 | *bach1* | *BACH1* | 0.619 | 3.18E-02 |
| NM_001110486 | Dr.133614 | *zgc:158292* | *FILIP1L* | 0.619 | 4.68E-02 |
| NM_205561 | Dr.7422 | *stau1* | *STAU1* | 0.619 | 1.19E-02 |
| NM_200935 | Dr.12713 | *pitpnaa* | *PITPNAA* | 0.618 | 3.27E-02 |
| NM_001002662 | Dr.39062 | *rgs2* | *RGS2* | 0.617 | 3.52E-02 |
| NM_213154 | Dr.3570 | *pdhb* | *PDHB* | 0.614 | 7.67E-03 |
| NM_001045144 | Dr.114606 | *cog5* | *COG5* | 0.614 | 1.37E-02 |
| NM_001030086 | Dr.32933 | *kin* | *KIN* | 0.611 | 4.30E-02 |
| NM_001083025 | Dr.106889 | *pcsk7* | *PCSK7* | 0.611 | 4.14E-03 |
| NM_001017747 | Dr.16371 | *coq10b* | *COQ10B* | 0.610 | 4.28E-03 |
| NM_212963 | Dr.81989 | *dip2ba* | *DIP2B* | 0.610 | 7.57E-03 |
| NM_001075110 | Dr.81817 | *otud5a* | *OTUD5* | 0.609 | 4.94E-03 |
| NM_199630 | Dr.77415 | *stx5al* | *STX5* | 0.609 | 4.61E-02 |
| NM_199603 | Dr.77223 | *zgc:65788* | *CHIA* | 0.608 | 2.53E-03 |
| NM_001128530 | Dr.31764 | *ap1b1* | *AP1B1* | 0.608 | 3.39E-02 |
| NM_213330 | Dr.75230 | *sgsm3* | *SGSM3* | 0.607 | 4.29E-02 |
| NM_001145585 | Dr.66855 | *osbp* | *OSBP* | 0.606 | 3.79E-02 |
| NM_001044755 | Dr.18195 | *si:dkey-11e23.5* | *KIAA0247* | 0.605 | 7.01E-03 |
| NM_205759 | Dr.30522 | *eepd1* | *EEPD1* | 0.602 | 1.26E-02 |
| NM_200487 | Dr.81406 | *tbc1d23* | *TBC1D23* | 0.601 | 5.57E-03 |
| NM_173240 | Dr.76448 | *actl6a* | *ACTL6B* | 0.600 | 1.68E-02 |
| NM_001045158 | Dr.81066 | *sbf1* | *SBF1* | 0.600 | 2.49E-02 |
| NM_001003451 | Dr.81043 | *dusp2* | *DUSP2* | 0.600 | 2.01E-02 |
| NM_001126454 | Dr.78262 | *fam222a* | *C12orf34* | 0.598 | 1.85E-02 |
| NM_001013443 | Dr.36043 | *eif4g2b* | *EIF4G2* | 0.597 | 2.80E-03 |
| NM_212916 | Dr.75201 | *gatad2ab* | *GATAD2AB* | 0.594 | 1.31E-02 |
| NM_001172625 | Dr.77623 | *tbc1d15* | *TBC1D15* | 0.594 | 2.42E-02 |
| NM_001115138 | Dr.5648 | *ewsr1a* | *EWSR1* | 0.593 | 4.97E-02 |
| NM_001020684 | Dr.76726 | *clpp* | *CLPP* | 0.593 | 4.18E-02 |
| NM_199934 | Dr.370 | *atp6v1g1* | *ATP6V1G1* | 0.593 | 1.16E-02 |
| NM_001002181 | Dr.76909 | *mthfd2* | *MTHFD2* | 0.593 | 1.77E-02 |
| NM_199697 | Dr.78599 | *gtf2b* | *GTF2B* | 0.592 | 9.23E-03 |
| NM_212602 | Dr.2594 | *ahsa1l* | *AHSA1* | 0.591 | 1.70E-02 |
| NM_131877 | Dr.11726 | *casp3a* | *CASP3* | 0.589 | 2.76E-02 |
| NM_001089510 | Dr.90354 | *zgc:163064* | *TNFRSF1B* | 0.589 | 9.98E-03 |
| NM_001190755 | Dr.83979 | *slc1a5* | *SLC1A5* | 0.588 | 1.01E-02 |
| NM_200649 | Dr.78711 | *acsl4a* | *ACSL4* | 0.586 | 5.95E-03 |
| NM_198910 | Dr.9751 | *fmo5* | *FMO5* | 0.585 | 3.26E-02 |
| NM_001002527 | Dr.75889 | *sec61b* | *SEC61B* | 0.582 | 1.13E-02 |
| NM_131514 | Dr.81267 | *psen2* | *PSEN2* | 0.582 | 1.43E-02 |
| NM_001172308 | Dr.162139 | *LOC100003647* | *LOC100003647* | 0.581 | 1.09E-02 |
| NM_001002870 | Dr.16482 | *trim24* | *TRIM24* | 0.580 | 3.86E-02 |
| NM_001080989 | Dr.87366 | *dennd4a* | *DENND4A* | 0.578 | 3.92E-02 |
| NM_001110124 | Dr.79973 | *rpz5* | *RPZ5* | 0.578 | 3.52E-02 |
| NM_001003627 | Dr.34030 | *me2* | *ME2* | 0.578 | 3.17E-02 |
| NM_001128381 | Dr.134981 | *traf1* | *TRAF1* | 0.578 | 3.95E-02 |
| NM_199633 | Dr.33037 | *wsb1* | *WSB1* | 0.577 | 8.82E-03 |
| NM_199956 | Dr.7036 | *g3bp1* | *G3BP2* | 0.573 | 5.07E-03 |
| NM_001003776 | Dr.84694 | *ncaldb* | *NCALD* | 0.573 | 7.97E-03 |
| NM_001002655 | Dr.69449 | *atxn7l2b* | *ATXN7L2* | 0.572 | 2.05E-02 |
| NM_001039816 | Dr.79058 | *specc1la* | *SPECC1L* | 0.571 | 3.91E-02 |
| NM_213415 | Dr.1692 | *zbtb2b* | *ZBTB2* | 0.568 | 4.66E-02 |
| NM_001044829 | Dr.152163 | *si:dkey-239i20.2* | *FMO5* | 0.567 | 3.77E-02 |
| NM_001002453 | Dr.118048 | *zgc:92335* | *MMADHC* | 0.566 | 4.52E-02 |
| NM_001017781 | Dr.40351 | *arhgap1* | *ARHGAP1* | 0.565 | 1.60E-02 |
| NM_001037114 | Dr.5714 | *nubp2* | *NUBP2* | 0.564 | 3.55E-02 |
| NM_001045100 | Dr.74757 | *snx27a* | *SNX27* | 0.564 | 4.50E-02 |
| NM_001114563 | Dr.93039 | *zgc:172106* | *SIGLEC1* | 0.563 | 3.08E-02 |
| NM_213066 | Dr.80401 | *hk2* | *HK2* | 0.562 | 2.63E-02 |
| NM_200269 | Dr.3685 | *ostc* | *OSTC* | 0.561 | 2.72E-02 |
| NM_001008590 | Dr.84283 | *slc35c1* | *SLC35C1* | 0.560 | 1.75E-02 |
| NM_212983 | Dr.26997 | *zgc:66427* | *N.A.* | 0.559 | 1.87E-02 |
| NM_213408 | Dr.75253 | *slc25a22* | *SLC25A22* | 0.557 | 1.20E-02 |
| NM_199715 | Dr.79146 | *chp1* | *CHP* | 0.556 | 1.09E-02 |
| NM_213157 | Dr.76663 | *slc25a33* | *SLC25A36* | 0.554 | 9.54E-03 |
| NM_198980 | Dr.83115 | *ldlrap1a* | *LDLRAP1* | 0.553 | 2.37E-02 |
| NM_200357 | Dr.25864 | *zgc:63992* | *C20orf111* | 0.553 | 2.69E-02 |
| NM_131339 | Dr.23544 | *rarga* | *RARG* | 0.553 | 1.96E-02 |
| NM_001020669 | Dr.150731 | *zgc:112255* | *C1orf50* | 0.550 | 4.24E-02 |
| NM_001126465 | Dr.88290 | *sort1b* | *SORT1* | 0.549 | 4.67E-02 |
| NM_213368 | Dr.8647 | *arfip1* | *ARFIP1* | 0.547 | 1.68E-02 |
| NM_001099238 | Dr.159284 | *zgc:158564* | *N.A.* | 0.547 | 2.54E-02 |
| NM_199777 | Dr.3854 | *sec23b* | *SEC23A* | 0.546 | 7.58E-03 |
| NM_001123048 | Dr.119244 | *rnf139* | *RNF139* | 0.545 | 4.38E-02 |
| NM_001127475 | Dr.155224 | *zgc:175175* | *N.A.* | 0.541 | 1.70E-02 |
| NM_214682 | Dr.76915 | *cyth1a* | *CYTH1* | 0.540 | 1.47E-02 |
| NM_001002175 | Dr.79252 | *zgc:92432* | *N.A.* | 0.539 | 3.55E-02 |
| NM_001128825 | Dr.53940 | *rab11fip4b* | *RAB11FIP4B* | 0.539 | 2.77E-02 |
| NM_131859 | Dr.79292 | *klf3* | *KLF3* | 0.536 | 5.00E-02 |
| NM_001007378 | Dr.37361 | *pptc7* | *PPTC7* | 0.534 | 2.13E-02 |
| NM_199781 | Dr.35557 | *zgc:66160* | *ZP3* | 0.534 | 2.16E-02 |
| NM_001144040 | Dr.82136 | *cog3* | *COG3* | 0.534 | 3.30E-02 |
| NM_001128669 | Dr.36926 | *samsn1a* | *SAMSN1A* | 0.533 | 9.65E-03 |
| NM_200734 | Dr.28218 | *bcl6a* | *BCL6* | 0.533 | 4.28E-02 |
| NM_201315 | Dr.104616 | *abcf2a* | *ABCF2* | 0.533 | 1.34E-02 |
| NM_001003420 | Dr.84835 | *zgc:92251* | *SLC10A7* | 0.533 | 3.02E-02 |
| NM_212700 | Dr.35945 | *degs1* | *DEGS2* | 0.533 | 3.95E-02 |
| NM_001089478 | Dr.79929 | *fam114a1* | *FAM114A1* | 0.531 | 4.96E-02 |
| NM_200023 | Dr.76185 | *zgc:56493* | *TXN* | 0.530 | 9.14E-03 |
| NM_200020 | Dr.159465 | *btg1* | *BTG1* | 0.528 | 1.11E-02 |
| NM_200971 | Dr.14593 | *gnai1* | *GNAI3* | 0.527 | 1.59E-02 |
| NM_131710 | Dr.19238 | *ctsd* | *CTSD* | 0.526 | 3.28E-02 |
| NM_001144803 | Dr.9166 | *blzf1* | *BLZF1* | 0.525 | 3.05E-02 |
| NM_131087 | Dr.81254 | *jak2b* | *JAK2* | 0.524 | 2.49E-02 |
| NM_001002419 | Dr.51881 | *entpd4* | *ENTPD4* | 0.524 | 4.13E-02 |
| NM_199903 | Dr.6975 | *larp6* | *LARP6* | 0.524 | 1.99E-02 |
| NM_001002096 | Dr.81815 | *yipf6* | *YIPF6* | 0.524 | 3.48E-02 |
| NM_198821 | Dr.9667 | *dnajb11* | *DNAJB11* | 0.523 | 4.52E-02 |
| NM_001077785 | Dr.40449 | *zgc:153996* | *VPS37C* | 0.521 | 2.71E-02 |
| NM_199602 | Dr.76484 | *zgc:77849* | *C16orf72* | 0.518 | 1.56E-02 |
| NM_201454 | Dr.76947 | *rab2a* | *RAB2A* | 0.517 | 1.57E-02 |
| NM_200787 | Dr.11214 | *gnao1a* | *GNAO1* | 0.516 | 2.18E-02 |
| NM_199734 | Dr.115452 | *slmo2* | *SLMO2* | 0.513 | 3.55E-02 |
| NM_001007775 | Dr.28550 | *ptp4a1* | *PTP4A1* | 0.512 | 2.46E-02 |
| NM_200910 | Dr.105233 | *sdha* | *SDHAP3* | 0.511 | 1.88E-02 |
| NM_001099988 | Dr.80038 | *gltpa* | *GLTP* | 0.507 | 3.59E-02 |
| NM_207061 | Dr.79855 | *tollip* | *TOLLIP* | 0.507 | 1.49E-02 |
| NM_001003497 | Dr.77249 | *gltpd1* | *GLTPD1* | 0.506 | 4.59E-02 |
| NM_212621 | Dr.77112 | *uap1* | *UAP1L1* | 0.505 | 3.93E-02 |
| NM_131686 | Dr.75307 | *atp1a1* | *ATP1A1* | 0.503 | 2.37E-02 |
| NM_200092 | Dr.78455 | *bcap31* | *BCAP31* | 0.500 | 3.92E-02 |
| NM_001114741 | Dr.76446 | *zgc:175221* | *SPTLC2* | 0.500 | 1.88E-02 |
| NM_181603 | Dr.2414 | *smarca4* | *SMARCA4* | 0.499 | 2.60E-02 |
| NM_199584 | Dr.77007 | *hiat1a* | *HIAT1* | 0.498 | 1.94E-02 |
| NM_001003645 | Dr.83746 | *carm1* | *CARM1* | 0.496 | 4.55E-02 |
| NM_213137 | Dr.32765 | *rhoaa* | *RHOA* | 0.496 | 3.47E-02 |
| NM_001006016 | Dr.79955 | *atl3* | *ATL3* | 0.493 | 4.27E-02 |
| NM_212588 | Dr.5307 | *slc20a1b* | *SLC20A1* | 0.493 | 4.17E-02 |
| NM_001077775 | Dr.104270 | *rps6ka1* | *RPS6KA3* | 0.493 | 1.50E-02 |
| NM_001002064 | Dr.31059 | *psmc3* | *PSMC3* | 0.492 | 3.57E-02 |
| NM_199845 | Dr.80559 | *gclm* | *GCLM* | 0.492 | 4.60E-02 |
| NM_201331 | Dr.76149 | *rdh10b* | *RDH10* | 0.492 | 3.54E-02 |
| NM_200767 | Dr.80153 | *chchd2* | *CHCHD2* | 0.490 | 2.48E-02 |
| NM_001079702 | Dr.22015 | *pisd* | *PISD* | 0.486 | 4.12E-02 |
| NM_001077583 | Dr.77694 | *zmynd11* | *ZMYND11* | 0.484 | 3.90E-02 |
| NM_200441 | Dr.79637 | *fam91a1* | *FAM91A1* | 0.481 | 4.62E-02 |
| NM_001030070 | Dr.24921 | *gpx4b* | *GPX4* | 0.481 | 3.58E-02 |
| NM_001033718 | Dr.78229 | *znf598* | *ZNF598* | 0.479 | 2.65E-02 |
| NM_001079826 | Dr.162190 | *bida* | *BIDA* | 0.478 | 4.00E-02 |
| NM_199682 | Dr.78760 | *ap1g1* | *AP1G1* | 0.478 | 3.76E-02 |
| NM_001177932 | Dr.92993 | *sec24d* | *SEC24D* | 0.478 | 3.25E-02 |
| NM_199275 | Dr.24221 | *arf5* | *ARF5* | 0.478 | 2.36E-02 |
| NM_194378 | Dr.76631 | *arpc5a* | *ARPC5* | 0.477 | 1.93E-02 |
| NM_001145555 | Dr.73 | *rrbp1a* | *RRBP1* | 0.476 | 4.82E-02 |
| NM_131264 | Dr.79593 | *ahr2* | *AHR* | 0.475 | 4.33E-02 |
| NM_201452 | Dr.4828 | *arf1* | *ARF1* | 0.475 | 2.38E-02 |
| NM_213189 | Dr.81839 | *pla2g7* | *PLA2G7* | 0.474 | 3.33E-02 |
| NM_001001403 | Dr.78072 | *atp13a* | *ATP13A1* | 0.472 | 4.03E-02 |
| NM_213296 | Dr.81512 | *timp2b* | *TIMP2B* | 0.470 | 4.05E-02 |
| NM_001045412 | Dr.79622 | *tspan14* | *TSPAN14* | 0.466 | 4.89E-02 |
| NM_001003763 | Dr.150579 | *eif3ha* | *EIF3H* | 0.459 | 3.11E-02 |
| NM_001002461 | Dr.79021 | *txn* | *TXN* | 0.458 | 3.08E-02 |
| NM_199842 | Dr.80461 | *gnai2* | *GNAI3* | 0.458 | 2.43E-02 |
| NM_001077159 | Dr.132240 | *hectd3* | *HECTD3* | 0.457 | 2.91E-02 |
| NM_212669 | Dr.22923 | *sypl2b* | *SYPL1* | 0.456 | 3.54E-02 |
| NM_199928 | Dr.75260 | *cnppd1* | *CNPPD1* | 0.454 | 3.12E-02 |
| NM_001044967 | Dr.144132 | *si:dkey-222f8.3* | *N.A.* | 0.453 | 4.51E-02 |
| NM_001166628 | Dr.78670 | *ppp3cca* | *PPP3CA* | 0.451 | 4.91E-02 |
| NM_200952 | Dr.13584 | *kcmf1* | *KCMF1* | 0.444 | 3.92E-02 |
| NM_001256176 | Dr.76604 | *tmem214* | *TMEM214* | 0.442 | 4.35E-02 |
| NM_001103194 | Dr.76290 | *uqcrfs1* | *UQCRFS1* | 0.441 | 4.21E-02 |
| NM_001005592 | Dr.7906 | *cox6a1* | *COX6A1* | 0.441 | 3.76E-02 |
| NM_001002705 | Dr.21400 | *zgc:92610* | *TMC6* | 0.436 | 4.41E-02 |
| NM_001030277 | Dr.76755 | *atp2a2b* | *ATP2A2* | 0.431 | 3.97E-02 |
| NM_213495 | Dr.7761 | *cul1b* | *CUL1* | 0.431 | 4.62E-02 |
| NM_199710 | Dr.8749 | *wbp2* | *WBP2* | 0.430 | 3.83E-02 |
| NM_213311 | Dr.105888 | *pnp5a* | *PNP* | 0.428 | 3.29E-02 |
| NM_213532 | Dr.79921 | *creb3l3l* | *CREB3* | 0.426 | 4.67E-02 |
| NM_213457 | Dr.16859 | *chmp4b* | *CHMP4B* | 0.426 | 3.90E-02 |
| NM_201287 | Dr.4829 | *alas1* | *ALAS1* | 0.426 | 3.15E-02 |
| NM_001145244 | Dr.87835 | *scpp8* | *SCPP8* | 0.424 | 4.16E-02 |
| NM_001045208 | Dr.78796 | *letm1* | *LETM1* | 0.422 | 4.93E-02 |
| NM_198805 | Dr.77804 | *gnaia* | *GNAI3* | 0.417 | 4.96E-02 |
| NM_198809 | Dr.32367 | *tubb4b* | *TUBB2B* | 0.413 | 3.50E-02 |
| NM_001083858 | Dr.78007 | *zgc:158446* | *CFB* | -0.437 | 3.71E-02 |
| NM_001045308 | Dr.77881 | *zgc:136474* | *C1orf63* | -0.455 | 3.06E-02 |
| NM_213126 | Dr.77427 | *b2ml* | *B2ML* | -0.467 | 4.89E-02 |
| NM_212750 | Dr.737 | *junbb* | *JUNB* | -0.468 | 4.58E-02 |
| NM_199996 | Dr.6963 | *calm2a* | *CALM2A* | -0.476 | 3.80E-02 |
| NM_213556 | Dr.10326 | *junba* | *JUNB* | -0.477 | 4.08E-02 |
| NM_001082997 | Dr.79376 | *si:dkey-23c22.6* | *TNFRSF14* | -0.486 | 3.85E-02 |
| NM_001199725 | Dr.51349 | *hp1bp3* | *HP1BP3* | -0.499 | 4.09E-02 |
| NM_001105110 | Dr.152825 | *hephl1* | *HEPHL1* | -0.499 | 4.12E-02 |
| NM_001115095 | Dr.81316 | *cdc25* | *CDC25B* | -0.505 | 3.72E-02 |
| NM_001020642 | Dr.44139 | *rhof* | *LOC338799* | -0.507 | 1.36E-02 |
| NM_213190 | Dr.27758 | *tnfrsf1a* | *TNFRSF1A* | -0.510 | 2.00E-02 |
| NM_001040390 | Dr.159454 | *zgc:136929* | *CTBP1* | -0.514 | 2.71E-02 |
| NM_001030138 | Dr.5195 | *apol1* | *APOL1* | -0.518 | 4.34E-02 |
| NM_152963 | Dr.10644 | *fmr1* | *FMR1* | -0.519 | 4.29E-02 |
| NM_001045392 | Dr.86353 | *batf3* | *BATF3* | -0.522 | 4.89E-02 |
| NM_194376 | Dr.105146 | *ptmaa* | *PTMAA* | -0.527 | 1.56E-02 |
| NM_001077552 | Dr.83785 | *dcaf5* | *DCAF5* | -0.529 | 4.61E-02 |
| NM_001002573 | Dr.76905 | *zgc:92744* | *SERP1* | -0.531 | 2.38E-02 |
| NM_001004601 | Dr.76746 | *nucks1a* | *NUCKS1* | -0.531 | 4.51E-02 |
| NM_001001846 | Dr.145037 | *pura* | *PURA* | -0.532 | 4.11E-02 |
| NM_001002706 | Dr.134016 | *lygl1* | *LYG2* | -0.534 | 3.34E-02 |
| NM_199621 | Dr.77334 | *lnpep* | *LNPEP* | -0.534 | 1.43E-02 |
| NM_001099261 | Dr.83064 | *zgc:162641* | *SLC25A17* | -0.536 | 4.86E-02 |
| NM_001126455 | Dr.106452 | *zgc:162944* | *N.A.* | -0.536 | 1.60E-02 |
| NM_200164 | Dr.16249 | *slc25a14* | *SLC25A14* | -0.536 | 4.86E-02 |
| NM_001079952 | Dr.150673 | *tnip1* | *TNIP1* | -0.538 | 3.14E-02 |
| NM_131465 | Dr.372 | *pcmt* | *PCMT1* | -0.543 | 3.91E-02 |
| NM_001161336 | Dr.75621 | *fb06f03* | *FB06F03* | -0.547 | 2.03E-02 |
| NM_213351 | Dr.76175 | *calm1a* | *CALM1A* | -0.547 | 1.82E-02 |
| NM_212869 | Dr.30396 | *trib3* | *TRIB3* | -0.552 | 2.19E-02 |
| NM_200179 | Dr.105353 | *cdc14b* | *CDC14B* | -0.554 | 3.39E-02 |
| NM_212937 | Dr.79747 | *mycl1a* | *MYCL1* | -0.554 | 4.36E-02 |
| NM_001004604 | Dr.76622 | *zgc:92481* | *VWA5A* | -0.557 | 3.68E-02 |
| NM_199638 | Dr.77699 | *tomm34* | *TOMM34* | -0.558 | 3.58E-02 |
| NM_001080034 | Dr.75902 | *zgc:158387* | *N.A.* | -0.564 | 2.44E-02 |
| NM_001123326 | Dr.82327 | *zgc:153958* | *DALRD3* | -0.565 | 4.83E-02 |
| NM_001200015 | Dr.32270 | *si:ch73-178d14.1* | *TOR1B* | -0.566 | 2.09E-02 |
| NM_200419 | Dr.87570 | *tpst2* | *TPST2* | -0.566 | 2.34E-02 |
| NM_213223 | Dr.10261 | *marcksl1b* | *MARCKSL1B* | -0.573 | 2.42E-02 |
| NM_198870 | Dr.75334 | *aif1l* | *AIF1L* | -0.573 | 3.69E-02 |
| NM_001098746 | Dr.82671 | *coq9* | *COQ9* | -0.577 | 4.35E-02 |
| NM_001001817 | Dr.78702 | *zgc:92326* | *LGALS9* | -0.579 | 9.22E-03 |
| NM_205739 | Dr.78415 | *zgc:101841* | *DGCR6L* | -0.580 | 4.62E-02 |
| NM_001017662 | Dr.82544 | *zgc:112056* | *C21orf33* | -0.584 | 4.96E-02 |
| NM_001201535 | Dr.75736 | *gon4l* | *YY1AP1* | -0.587 | 4.89E-02 |
| NM_001009892 | Dr.82062 | *serpinb1l1* | *SERPINB6* | -0.590 | 1.54E-02 |
| NM_201044 | Dr.80141 | *rnf13* | *RNF13* | -0.594 | 1.10E-02 |
| NM_198912 | Dr.79690 | *pepd* | *PEPD* | -0.596 | 1.81E-02 |
| NM_001165918 | Dr.74463 | *wu:fb15g10* | *WU:FB15G10* | -0.599 | 6.98E-03 |
| NM_214815 | Dr.75204 | *thrap3b* | *THRAP3B* | -0.603 | 9.31E-03 |
| NM_001089515 | Dr.42370 | *zgc:162228* | *TRAFD1* | -0.604 | 2.38E-02 |
| NM_001102636 | Dr.151019 | *zgc:165582* | *N.A.* | -0.609 | 4.16E-02 |
| NM_200133 | Dr.78030 | *ftsjd2* | *FTSJD2* | -0.613 | 2.80E-02 |
| NM_001003993 | Dr.80929 | *zgc:91930* | *AK1* | -0.614 | 3.29E-02 |
| NM_213130 | Dr.75392 | *ak3* | *AK3* | -0.615 | 3.63E-02 |
| NM_001007406 | Dr.89586 | *xkrx* | *XKRX* | -0.618 | 3.52E-02 |
| NM_001017853 | Dr.27897 | *cbx7a* | *CBX7A* | -0.618 | 2.53E-02 |
| NM_001030077 | Dr.49097 | *slc9a7* | *SLC9A7* | -0.619 | 4.67E-02 |
| NM_001025471 | Dr.77233 | *fdps* | *FDPS* | -0.622 | 4.82E-02 |
| NM_001002560 | Dr.76489 | *zgc:92763* | *GSTK1* | -0.623 | 3.86E-02 |
| NM_199619 | Dr.2490 | *csrnp1b* | *CSRNP1* | -0.623 | 5.89E-03 |
| NM_213450 | Dr.77852 | *ssbp3b* | *SSBP3B* | -0.623 | 1.23E-02 |
| NM_199891 | Dr.80698 | *ecd* | *ECD* | -0.625 | 4.23E-02 |
| NM_201471 | Dr.104770 | *aldh9a1a* | *ALDH9A1A* | -0.628 | 2.62E-03 |
| NM_199950 | Dr.6431 | *socs3a* | *SOCS3* | -0.633 | 5.82E-03 |
| NM_001128395 | Dr.134371 | *si:ch211-71m22.1* | *PLSCR1* | -0.635 | 3.22E-02 |
| NM_001130400 | Dr.105819 | *si:dkey-67c22.2* | *N.A.* | -0.638 | 2.88E-02 |
| NM_213104 | Dr.75881 | *pgrmc2* | *PGRMC2* | -0.640 | 7.41E-03 |
| NM_001082938 | Dr.77146 | *si:ch211-13c6.3* | *SI:CH211-13C6.3* | -0.641 | 3.22E-02 |
| NM_199987 | Dr.1064 | *jun* | *JUN* | -0.643 | 4.42E-02 |
| NM_213304 | Dr.81607 | *socs3b* | *SOCS3* | -0.644 | 8.56E-03 |
| NM_199917 | Dr.75926 | *synj2bp* | *SYNJ2BP* | -0.647 | 2.13E-02 |
| NM_200710 | Dr.80988 | *paqr3a* | *PAQR3* | -0.648 | 2.46E-02 |
| NM_001002646 | Dr.88169 | *ada* | *ADA* | -0.650 | 4.92E-02 |
| NM_213194 | Dr.76786 | *usf1l* | *USF1* | -0.653 | 4.89E-03 |
| NM_001083829 | Dr.94833 | *pcsk5b* | *PCSK5* | -0.653 | 2.59E-02 |
| NM_001003467 | Dr.79974 | *socs1* | *SOCS1* | -0.654 | 3.36E-02 |
| NM_001089404 | Dr.106159 | *zgc:162730* | *N.A.* | -0.658 | 1.56E-02 |
| NM_001079956 | Dr.76670 | *pbxip1b* | *PBXIP1* | -0.658 | 2.47E-02 |
| NM_001080074 | Dr.79635 | *zgc:158316* | *FAM126B* | -0.659 | 3.06E-02 |
| NM_001114571 | Dr.17331 | *zgc:174863* | *N.A.* | -0.660 | 3.40E-03 |
| NM_001030208 | Dr.84802 | *gnsa* | *GNS* | -0.662 | 2.59E-02 |
| NM_001126480 | Dr.159497 | *LOC100006238* | *ALDH9A1* | -0.663 | 2.33E-02 |
| NM_213147 | Dr.150756 | *acox3* | *ACOX3* | -0.665 | 4.37E-02 |
| NM_213385 | Dr.77494 | *vrk1* | *VRK1* | -0.666 | 2.34E-02 |
| NM_200748 | Dr.83261 | *pin1* | *PIN1* | -0.667 | 1.17E-02 |
| NM_201208 | Dr.26454 | *grina* | *GRINA* | -0.669 | 2.78E-03 |
| NM_213432 | Dr.77272 | *e2f4* | *E2F4* | -0.671 | 3.87E-02 |
| NM_001004114 | Dr.2818 | *phf20b* | *PHF20* | -0.672 | 1.64E-02 |
| NM_001128702 | Dr.120543 | *pip4k2aa* | *PIP4K2A* | -0.672 | 3.34E-02 |
| NM_131601 | Dr.79263 | *slc3a2a* | *SLC3A2* | -0.672 | 3.33E-02 |
| NM_199942 | Dr.10070 | *fbp1a* | *FBP2* | -0.678 | 1.60E-02 |
| NM_001099239 | Dr.75673 | *nucks1b* | *NUCKS1B* | -0.679 | 2.97E-02 |
| NM_001045297 | Dr.78091 | *zgc:136560* | *ABI2* | -0.684 | 1.89E-02 |
| NM_199822 | Dr.80428 | *ccdc82* | *CCDC82* | -0.687 | 4.73E-02 |
| NM_212706 | Dr.79316 | *cul5a* | *CUL5A* | -0.691 | 2.32E-02 |
| NM_001004116 | Dr.80294 | *elf2a* | *ELF2A* | -0.691 | 2.21E-02 |
| NM_001089346 | Dr.952 | *zgc:162544* | *MPST* | -0.694 | 2.16E-02 |
| NM_001083875 | Dr.83525 | *mgat2* | *MGAT2* | -0.695 | 4.75E-02 |
| NM_213014 | Dr.151164 | *slc37a2* | *SLC37A2* | -0.698 | 3.08E-02 |
| NM_001278180 |  |  | *CASD1* | -0.699 | 2.37E-02 |
| NM_001040348 | Dr.77273 | *rab40c* | *RAB40C* | -0.702 | 1.55E-02 |
| NM_001114570 | Dr.119979 | *ch25hl2* | *CH25HL2* | -0.704 | 1.76E-02 |
| NM_131719 | Dr.105878 | *cdk5* | *CDK5* | -0.704 | 2.63E-02 |
| NM_001199754 | Dr.76939 | *nmt2* | *NMT2* | -0.710 | 4.38E-02 |
| NM_001113510 | Dr.124927 | *il21r* | *IL21R* | -0.711 | 1.08E-02 |
| NM_205683 | Dr.85211 | *tcea3* | *TCEA3* | -0.712 | 2.65E-03 |
| NM_001115052 | Dr.74758 | *si:dkey-281i8.1* | *N.A.* | -0.713 | 2.88E-02 |
| NM_131724 | Dr.150491 | *map2k6* | *MAP2K6* | -0.713 | 1.72E-03 |
| NM_001014323 | Dr.108377 | *accs* | *ACCS* | -0.714 | 4.34E-02 |
| NM_001080007 | Dr.149115 | *capn5a* | *CAPN5A* | -0.714 | 1.47E-02 |
| NM_001044774 | Dr.11495 | *trit1* | *TRIT1* | -0.716 | 3.12E-02 |
| NM_001017661 | Dr.132688 | *ctssb.2* | *CTSS* | -0.719 | 3.53E-02 |
| NM_001039637 | Dr.106330 | *foxp1b* | *Foxp1* | -0.719 | 2.24E-02 |
| NM_001025539 | Dr.81522 | *fgl2* | *FGL2* | -0.719 | 3.38E-02 |
| NM_001030238 | Dr.135313 | *zgc:114045* | *N.A.* | -0.720 | 9.25E-03 |
| NM_205747 | Dr.119956 | *irf1b* | *IRF1* | -0.721 | 3.54E-02 |
| NM_001037118 | Dr.1836 | *pddc1* | *MORC2* | -0.722 | 2.99E-02 |
| NM_153671 | Dr.12608 | *cers2a* | *SETDB1* | -0.724 | 1.25E-02 |
| NM_001006090 | Dr.683 | *fam73a* | *FAM73A* | -0.725 | 2.57E-02 |
| NM_130922 | Dr.76624 | *btg2* | *BTG2* | -0.726 | 5.52E-04 |
| NM_001017804 | Dr.134210 | *adss* | *ADSS* | -0.727 | 4.68E-02 |
| NM_001080163 | Dr.90403 | *snapc5* | *SNAPC5* | -0.727 | 5.84E-03 |
| NM_001257157 | Dr.93597 | *LOC100148871* | *DHX58* | -0.728 | 1.80E-02 |
| NM_001002444 | Dr.15687 | *decr1* | *DECR1* | -0.728 | 3.93E-02 |
| NM_001079967 | Dr.83718 | *n4bp2* | *N4BP2* | -0.730 | 3.44E-02 |
| NM_001163313 | Dr.77262 | *msl2b* | *MSL2* | -0.730 | 2.48E-02 |
| NM_200414 | Dr.81013 | *cybb* | *CYBB* | -0.732 | 7.73E-03 |
| NM_200680 | Dr.152115 | *rhoga* | *RHOGA* | -0.733 | 1.15E-02 |
| NM_181758 | Dr.59 | *anxa1a* | *ANXA1* | -0.733 | 3.25E-02 |
| NM_001114468 | Dr.81197 | *zgc:175094* | *MSL1* | -0.734 | 6.49E-03 |
| NM_001044342 | Dr.74181 | *mov10b.1* | *MOV10* | -0.734 | 2.93E-03 |
| NM_213005 | Dr.8018 | *ggctb* | *GGCT* | -0.736 | 3.97E-02 |
| NM_001113585 | Dr.86245 | *LOC558402* | *LOC558402* | -0.738 | 3.61E-02 |
| NM_001079950 | Dr.85548 | *glcci1* | *GLCCI1* | -0.738 | 1.95E-02 |
| NM_200111 | Dr.85725 | *arhgap29b* | *ARHGAP29* | -0.739 | 1.96E-03 |
| NM_205723 | Dr.82530 | *gabarapl2* | *GABARAPL2* | -0.739 | 8.98E-04 |
| NM_194417 | Dr.80611 | *sepw2a* | *MIEN1* | -0.741 | 6.90E-04 |
| NM_001044756 | Dr.78874 | *cmtm6* | *CMTM6* | -0.741 | 1.46E-03 |
| NM_001080670 | Dr.61397 | *hs2st1a* | *HS2ST1* | -0.743 | 8.89E-03 |
| NM_130974 | Dr.159695 | *tapbp* | *TAPBP* | -0.744 | 7.42E-03 |
| NM_001045213 | Dr.143668 | *znf592* | *ZNF592* | -0.748 | 3.72E-02 |
| NM_001039983 | Dr.92028 | *ezh1* | *EZH2* | -0.749 | 2.81E-02 |
| NM_001123011 | Dr.79176 | *fam13b* | *FAM13B* | -0.751 | 8.26E-03 |
| NM_001199491 | Dr.97225 | *foxp4* | *FOXP4* | -0.752 | 1.78E-02 |
| NM_213001 | Dr.1761 | *klhl20* | *KLHL20* | -0.757 | 1.79E-02 |
| NM_199544 | Dr.5116 | *ctdsp2* | *CTDSPL* | -0.758 | 1.12E-02 |
| NM_001128792 | Dr.19805 | *vhll* | *VHLL* | -0.761 | 1.96E-02 |
| NM_001089480 | Dr.82019 | *zgc:162310* | *KLHDC2* | -0.763 | 1.07E-02 |
| NM_001129894 | Dr.143343 | *LOC100170201* | *N.A.* | -0.767 | 3.73E-02 |
| NM_194406 | Dr.82549 | *cbr1* | *CBR1* | -0.767 | 3.96E-02 |
| NM_001017552 | Dr.77551 | *zgc:112962* | *TOR1AIP1* | -0.769 | 4.98E-03 |
| NM_213167 | Dr.79731 | *kdm2ba* | *KDM2B* | -0.770 | 1.24E-02 |
| NM_001089372 | Dr.83530 | *zgc:162634* | *PYURF* | -0.771 | 1.53E-02 |
| NM_201301 | Dr.76768 | *fxr1* | *FXR1* | -0.773 | 2.74E-03 |
| NM_001077391 | Dr.67670 | *zgc:153997* | *PIM3* | -0.773 | 1.22E-03 |
| NM_001077733 | Dr.37007 | *znf362* | *ZNF362* | -0.777 | 3.57E-02 |
| NM_001166156 | Dr.82408 | *pkig* | *PKIG* | -0.777 | 6.62E-03 |
| NM_200066 | Dr.5851 | *nup35* | *NUP35* | -0.778 | 3.54E-02 |
| NM_001083852 | Dr.86171 | *wu:fu71h07* | *WU:FU71H07* | -0.780 | 1.16E-02 |
| NM_131573 | Dr.159468 | *dap1b* | *DAP1B* | -0.781 | 4.04E-02 |
| NM_001003531 | Dr.160020 | *fam151b* | *FAM151B* | -0.781 | 3.37E-02 |
| NM_001110278 | Dr.41373 | *acvr2a* | *ACVR2A* | -0.782 | 8.91E-03 |
| NM_201068 | Dr.106084 | *ephx1* | *EPHX1* | -0.783 | 2.52E-02 |
| NM_200486 | Dr.132778 | *slc25a39* | *SLC25A40* | -0.787 | 4.05E-02 |
| NM_212737 | Dr.23608 | *mpeg1* | *MPEG1* | -0.788 | 2.92E-02 |
| NM_001114703 | Dr.154576 | *zgc:172170* | *N.A.* | -0.789 | 1.94E-02 |
| NM_199735 | Dr.18138 | *zgc:66447* | *N.A.* | -0.790 | 2.68E-02 |
| NM_001130655 | Dr.86775 | *si:ch211-122l24.4* | *N.A.* | -0.790 | 3.36E-02 |
| NM_201145 | Dr.118394 | *nudt3a* | *NUDT3A* | -0.791 | 1.33E-02 |
| NM_001007788 | Dr.36704 | *mfng* | *MFNG* | -0.794 | 4.78E-04 |
| NM_001020721 | Dr.91144 | *mtmr1a* | *MTMR2* | -0.797 | 8.01E-03 |
| NM_001002134 | Dr.79838 | *tmed4* | *TMED9* | -0.797 | 2.28E-02 |
| NM_001076662 | Dr.79653 | *sfxn4* | *SFXN4* | -0.797 | 4.65E-02 |
| NM_001083830 | Dr.84913 | *zgc:198241* | *N.A.* | -0.798 | 4.14E-03 |
| NM_001044918 | Dr.62863 | *znf704* | *ZNF704* | -0.802 | 1.94E-02 |
| NM_200097 | Dr.84963 | *brms1* | *BRMS1* | -0.802 | 1.57E-02 |
| NM_001039838 | Dr.162322 | *nefl* | *NEFL* | -0.803 | 2.77E-02 |
| NM_001077606 | Dr.42522 | *amotl2b* | *AMOTL2* | -0.803 | 1.57E-02 |
| NM_213081 | Dr.76977 | *prkrira* | *PRKRIR* | -0.805 | 1.81E-02 |
| NM_001166134 | Dr.93052 | *btr29* | *BTR29* | -0.805 | 2.14E-02 |
| NM_199650 | Dr.77853 | *srpk1a* | *SRPK1* | -0.805 | 2.80E-03 |
| NM_001136242 | Dr.104771 | *ptprja* | *PTPRJ* | -0.808 | 1.47E-03 |
| NM_205707 | Dr.79798 | *phc1* | *PHC1* | -0.808 | 2.70E-02 |
| NM_001109718 | Dr.113248 | *mhc1ze* | *HLA-A* | -0.811 | 7.68E-03 |
| NM_131604 | Dr.82593 | *runx3* | *RUNX3* | -0.812 | 1.26E-03 |
| NM_001089548 | Dr.90794 | *zgc:162623* | *ZNF362* | -0.815 | 1.87E-02 |
| NM_001114573 | Dr.107500 | *zgc:171837* | *ZNF341* | -0.820 | 4.06E-02 |
| NM_173223 | Dr.123802 | *sdc2* | *SDC2* | -0.820 | 6.05E-03 |
| NM_201032 | Dr.82465 | *xpnpep2* | *XPNPEP2* | -0.823 | 1.87E-04 |
| NM_199814 | Dr.80413 | *zgc:66473* | *N.A.* | -0.823 | 3.61E-02 |
| NM_001040376 | Dr.18486 | *pkz* | *PKZ* | -0.824 | 3.37E-02 |
| NM_212668 | Dr.116955 | *zgc:55764* | *NAMPT* | -0.824 | 8.64E-03 |
| NM_001200059 | Dr.124820 | *LOC100151110* | *OPLAH* | -0.824 | 2.06E-02 |
| NM_173273 | Dr.75940 | *diexf* | *DIEXF* | -0.824 | 3.83E-02 |
| NM_001020532 | Dr.77244 | *cfdl* | *CFD* | -0.831 | 3.79E-02 |
| NM_213022 | Dr.107091 | *ywhag1* | *YWHAG1* | -0.832 | 3.77E-02 |
| NM_200192 | Dr.80589 | *scpep1* | *SCPEP1* | -0.832 | 1.08E-04 |
| NM_131795 | Dr.82172 | *psma6b* | *PSMA6* | -0.834 | 2.48E-03 |
| NM_200172 | Dr.78260 | *mycb* | *MYC* | -0.835 | 1.07E-02 |
| NM_001045848 | Dr.76979 | *zgc:153225* | *TSSC4* | -0.843 | 1.76E-02 |
| NM_001002725 | Dr.86298 | *zdhhc3* | *ZDHHC3* | -0.843 | 6.10E-03 |
| NM_001012480 | Dr.75610 | *bhmt* | *BHMT* | -0.845 | 2.32E-02 |
| NM_001278831 | N.A. | *N.A.* | *N.A.* | -0.846 | 1.31E-02 |
| NM_200916 | Dr.118099 | *panx1a* | *PANX1* | -0.847 | 2.35E-02 |
| NM_001030210 | Dr.94164 | *cx32.2* | *CX32.2* | -0.848 | 1.13E-02 |
| NM_001005934 | Dr.80185 | *gpd1l* | *GPD1L* | -0.851 | 3.29E-02 |
| NM_001017796 | Dr.43294 | *esd* | *ESD* | -0.853 | 4.32E-03 |
| NM_001202436 | Dr.76632 | *si:dkey-88p24.9* | *N.A.* | -0.854 | 3.18E-02 |
| NR_036574 | N.A. | *N.A.* | *N.A.* | -0.854 | 1.84E-02 |
| NM_200482 | Dr.9222 | *jkamp* | *JKAMP* | -0.857 | 3.09E-03 |
| NM_001166228 | Dr.135321 | *dock4b* | *DOCK4* | -0.858 | 2.70E-02 |
| NM_001001834 | Dr.15827 | *ift52* | *IFT52* | -0.859 | 2.36E-02 |
| NM_001002094 | Dr.77956 | *msrb3* | *MSRB3* | -0.864 | 4.34E-03 |
| NM_001007354 | Dr.85073 | *eif4ebp3* | *EIF4EBP3* | -0.866 | 1.30E-02 |
| NM_001014334 | Dr.18102 | *casp8ap2* | *CASP8AP2* | -0.874 | 2.81E-02 |
| NM_001001844 | Dr.53867 | *ctcf* | *CTCF* | -0.874 | 8.34E-05 |
| NM_131163 | Dr.51646 | *b2m* | *B2M* | -0.876 | 3.00E-04 |
| NM_001002748 | Dr.77360 | *zgc:100919* | *N.A.* | -0.876 | 2.53E-03 |
| NM_131596 | Dr.10204 | *adar* | *ADAR* | -0.877 | 5.52E-03 |
| NM_001123324 | Dr.17214 | *zgc:194839* | *N.A.* | -0.883 | 2.11E-02 |
| NM_001083068 | Dr.79113 | *lrp12* | *LRP12* | -0.884 | 4.37E-03 |
| NM_199978 | Dr.80253 | *gas6* | *GAS6* | -0.885 | 2.62E-02 |
| NM_001002168 | Dr.81856 | *rbck1* | *RBCK1* | -0.886 | 4.28E-04 |
| NM_194396 | Dr.77503 | *xiap* | *XIAP* | -0.886 | 2.15E-04 |
| NM_131375 | Dr.81309 | *psme1* | *PSME1* | -0.888 | 3.82E-04 |
| NM_212607 | Dr.77070 | *ubxn1* | *UBXN1* | -0.888 | 9.08E-03 |
| NM_001003462 | Dr.73072 | *trim35-36* | *TRIM35* | -0.889 | 1.13E-02 |
| NM_001007199 | Dr.88054 | *gpr161* | *GPR161* | -0.890 | 2.41E-02 |
| NM_212900 | Dr.119953 | *zgc:77058* | *N.A.* | -0.892 | 2.14E-02 |
| NM_212688 | Dr.14176 | *ctsh* | *CTSH* | -0.894 | 5.23E-03 |
| NM_194367 | Dr.76309 | *aldob* | *ALDOB* | -0.895 | 1.99E-02 |
| NM_001002727 | Dr.74633 | *rnf144ab* | *RNF144AB* | -0.896 | 8.00E-03 |
| NM_001003774 | Dr.78088 | *zgc:101000* | *N.A.* | -0.896 | 2.21E-02 |
| NM_001089445 | Dr.159818 | *ccnd2a* | *CCND2* | -0.901 | 1.97E-04 |
| NM_001110285 | Dr.84556 | *card9* | *CARD9* | -0.905 | 1.58E-04 |
| NM_001100954 | Dr.108637 | *LOC566993* | *N.A.* | -0.905 | 2.38E-02 |
| NM_001045081 | Dr.35714 | *fan1* | *FAN1* | -0.911 | 1.57E-02 |
| NM_001076647 | Dr.89531 | *zgc:153893* | *XAF1* | -0.912 | 7.31E-03 |
| NM_001045115 | Dr.43918 | *znf395b* | *ZNF395* | -0.913 | 2.57E-03 |
| NM_001128794 | Dr.86835 | *crp6* | *APCS* | -0.917 | 1.47E-02 |
| NM_199701 | Dr.20919 | *sephs1* | *SEPHS1* | -0.917 | 8.67E-03 |
| NM_001044820 | Dr.65799 | *mdm1* | *MDM1* | -0.921 | 7.06E-03 |
| NM_199579 | Dr.115559 | *usp1* | *USP1* | -0.923 | 3.44E-03 |
| NM_001002699 | Dr.15223 | *csrp2bp* | *PET117* | -0.924 | 1.85E-02 |
| NM_001013269 | Dr.82491 | *tlr3* | *TLR3* | -0.924 | 2.12E-02 |
| NM_001100008 | Dr.148566 | *zbtb33* | *ZBTB33* | -0.928 | 8.82E-03 |
| NM_001001947 | Dr.79081 | *dfna5* | *DFNA5* | -0.931 | 2.03E-02 |
| NM_001039922 | Dr.88446 | *slc43a3b* | *SLC43A3* | -0.931 | 1.35E-02 |
| NM_001017734 | Dr.76505 | *steap4* | *STEAP4* | -0.933 | 1.58E-02 |
| NM_001089332 | Dr.66850 | *zgc:162198* | *N.A.* | -0.937 | 2.36E-03 |
| NM_199623 | Dr.24816 | *dnajc5ga* | *DNAJC5GA* | -0.941 | 4.01E-03 |
| NM_001109850 | Dr.86370 | *mfap4* | *MFAP4* | -0.941 | 1.29E-02 |
| NM_001007436 | Dr.78776 | *c3orf58a* | *C3orf58* | -0.942 | 1.85E-02 |
| NM_131374 | Dr.76266 | *psme2* | *PSME2* | -0.945 | 7.48E-06 |
| NM_001257192 | Dr.72271 | *tfeb* | *TFEB* | -0.945 | 4.34E-03 |
| NM_001002368 | Dr.85174 | *ctsl.1* | *CTSL.1* | -0.947 | 7.77E-03 |
| NM_214810 | Dr.106912 | *cpe* | *CPE* | -0.947 | 8.06E-05 |
| NM_212919 | Dr.86120 | *zgc:85676* | *TMEM243* | -0.954 | 7.00E-03 |
| NM_001113651 | Dr.92011 | *LOC100003911* | *N.A.* | -0.962 | 1.63E-02 |
| NM_130944 | Dr.81314 | *dlc* | *DLC* | -0.965 | 1.16E-02 |
| NM_212736 | Dr.82151 | *mecp2* | *MECP2* | -0.965 | 2.71E-03 |
| NM_001037401 | Dr.86649 | *kcnk5a* | *KCNK5* | -0.968 | 5.26E-03 |
| NM_001256222 | Dr.89588 | *LOC553228* | *GGH* | -0.968 | 6.15E-03 |
| NM_001008642 | Dr.30620 | *ephx2* | *EPHX2* | -0.970 | 5.65E-05 |
| NM_001105523 | Dr.91240 | *znf711* | *ZNF711* | -0.970 | 1.10E-02 |
| NM_212693 | Dr.52859 | *bnip4* | *BNIP4* | -0.972 | 7.77E-03 |
| NM_001044340 | Dr.94434 | *mov10b.2* | *MOV10* | -0.975 | 4.96E-03 |
| NM_200570 | Dr.18416 | *selenbp1* | *SELENBP1* | -0.976 | 2.43E-03 |
| NM_200586 | Dr.76034 | *mb* | *MB* | -0.978 | 1.47E-02 |
| NM_200087 | Dr.18396 | *txnipa* | *TXNIP* | -0.979 | 4.22E-06 |
| NM_201128 | Dr.79185 | *sardh* | *SARDH* | -0.984 | 7.94E-03 |
| NM_200052 | Dr.76398 | *tsc1a* | *TSC1* | -0.988 | 1.37E-02 |
| NM_001166122 | Dr.48610 | *pcp4a* | *PCP4A* | -0.993 | 1.32E-02 |
| NM_001002658 | Dr.79291 | *frs2b* | *FRS2* | -0.994 | 9.00E-03 |
| NM_212701 | Dr.30302 | *hlx1* | *HLX* | -1.003 | 2.10E-05 |
| NM_001076607 | Dr.88400 | *tnfsf12* | *TNFSF12* | -1.007 | 9.72E-03 |
| NM_001161470 | Dr.132711 | *LOC325449* | *CDC23* | -1.009 | 1.18E-02 |
| NM_001204373 | Dr.77996 | *si:ch211-226m16.2* | *N.A.* | -1.013 | 3.89E-05 |
| NM_201116 | Dr.96738 | *sh3gl2* | *SH3GL1* | -1.017 | 1.10E-02 |
| NM_001083012 | Dr.29846 | *ms4a17a.1* | *MS4A17A.1* | -1.017 | 6.17E-03 |
| NM_001160126 | Dr.159631 | *si:dkeyp-22b2.3* | *CFP* | -1.023 | 5.09E-06 |
| NM_205710 | Dr.133138 | *irf9* | *IRF9* | -1.024 | 4.62E-04 |
| NM_001128785 | Dr.111674 | *zgc:194655* | *N.A.* | -1.024 | 1.04E-02 |
| NM_001126456 | Dr.41215 | *irg1* | *IRG1* | -1.025 | 2.26E-04 |
| NM_201199 | Dr.26647 | *pik3cd* | *PIK3CD* | -1.028 | 2.23E-05 |
| NM_001098245 | Dr.118332 | *si:dkey-24p1.1* | *N.A.* | -1.037 | 9.52E-03 |
| NM_200712 | Dr.12908 | *zgc:73100* | *C20orf11* | -1.047 | 8.66E-03 |
| NM_001045474 | Dr.82255 | *pmaip1* | *PMAIP1* | -1.051 | 2.32E-04 |
| NM_001044984 | Dr.85103 | *ptplad1* | *PTPLAD1* | -1.059 | 1.23E-03 |
| NM_213519 | Dr.5129 | *sesn3* | *SESN1* | -1.059 | 5.34E-05 |
| NM_001020741 | Dr.117434 | *calcoco2* | *CALCOCO2* | -1.061 | 6.64E-04 |
| NM_200520 | Dr.108160 | *zgc:66337* | *LBH* | -1.061 | 7.84E-03 |
| NM_001017778 | Dr.76224 | *ctsk* | *CTSK* | -1.064 | 5.42E-04 |
| NM_001202512 | Dr.33404 | *ssbp4* | *SSBP4* | -1.068 | 4.91E-03 |
| NM_178297 | Dr.9483 | *sepp1a* | *SEPP1* | -1.070 | 9.27E-05 |
| NM_001080690 | Dr.78083 | *zgc:158288* | *CREG1* | -1.076 | 5.91E-04 |
| NM_130957 | Dr.81289 | *clock* | *CLOCK* | -1.080 | 6.78E-03 |
| NM_200562 | Dr.78003 | *carhsp1* | *CARHSP1* | -1.080 | 2.78E-04 |
| NM_001007295 | Dr.37609 | *zgc:92107* | *N.A.* | -1.080 | 6.82E-03 |
| NM_001002502 | Dr.86419 | *zgc:92834* | *N6AMT1* | -1.083 | 2.20E-03 |
| NM_001040247 | Dr.79177 | *zgc:136881* | *POLH* | -1.083 | 1.17E-03 |
| NM_001126401 | Dr.114392 | *LOC561627* | *SLC16A3* | -1.085 | 1.00E-03 |
| NM_200568 | Dr.76027 | *zgc:77517* | *KRT17* | -1.091 | 3.26E-06 |
| NM_001245999 | Dr.162548 | *LOC100536817* | *N.A.* | -1.093 | 3.06E-03 |
| NM_001100055 | Dr.88360 | *si:dkey-71l1.7* | *N.A.* | -1.095 | 7.70E-04 |
| NM_001076576 | Dr.151464 | *zgc:153722* | *FBXO6* | -1.096 | 5.38E-04 |
| NM_001245987 | Dr.15615 | *LOC100005232* | *N.A.* | -1.115 | 3.03E-03 |
| NM_001009891 | Dr.76142 | *lancl1* | *LANCL1* | -1.116 | 2.54E-03 |
| NM_001113656 | Dr.114964 | *si:dkey-58f10.10* | *N.A.* | -1.124 | 3.69E-03 |
| NM_212879 | Dr.120285 | *irf10* | *IRF10* | -1.124 | 2.99E-03 |
| NM_131113 | Dr.75078 | *pou6f1* | *POU6F1* | -1.134 | 2.31E-03 |
| NM_001076757 | Dr.78844 | *pdgfab* | *PDGFAB* | -1.138 | 7.39E-04 |
| NM_001126475 | Dr.114958 | *LOC100003654* | *N.A.* | -1.163 | 1.47E-03 |
| NM_001045160 | Dr.58973 | *zbtb37* | *ZBTB37* | -1.169 | 2.23E-03 |
| NM_001285536 | N.A. | *N.A.* | *N.A.* | -1.169 | 5.17E-04 |
| NM_001110761 | Dr.89429 | *si:ch211-114l13.9* | *N.A.* | -1.173 | 3.02E-03 |
| NM_001044323 | Dr.107658 | *zgc:152753* | *N.A.* | -1.176 | 3.27E-03 |
| NM_213634 | Dr.75704 | *ptgdsb* | *PTGDSB* | -1.178 | 1.77E-04 |
| NM_131854 | Dr.83331 | *efna3b* | *EFNA3* | -1.180 | 2.56E-03 |
| NM_001114447 | Dr.87350 | *si:ch211-219a4.3* | *PARP14* | -1.182 | 6.78E-04 |
| NM_001128541 | Dr.114985 | *LOC567472* | *LOC567472* | -1.187 | 1.95E-04 |
| NM_199522 | Dr.106133 | *rnd3a* | *RND3* | -1.189 | 2.17E-03 |
| NM_131629 | Dr.81317 | *slc40a1* | *SLC40A1* | -1.189 | 9.48E-07 |
| NM_001002547 | Dr.2219 | *psmf1* | *PSMF1* | -1.197 | 6.80E-05 |
| NM_001204169 | Dr.114892 | *isg15* | *ISG15* | -1.206 | 4.29E-04 |
| NM_001007302 | Dr.162086 | *anxa3b* | *ANXA3* | -1.217 | 3.11E-07 |
| NM_001123289 | Dr.116234 | *si:dkey-56i24.1* | *N.A.* | -1.223 | 9.85E-05 |
| NM_001113579 | Dr.22056 | *LOC100149189* | *CFP* | -1.227 | 1.25E-05 |
| NM_001014815 | Dr.83304 | *eif4e2* | *EIF4E2* | -1.229 | 9.11E-04 |
| NM_001077614 | Dr.83960 | *sgms1* | *SGMS1* | -1.247 | 1.30E-04 |
| NM_131372 | Dr.75719 | *cd74b* | *CD74B* | -1.249 | 2.58E-04 |
| NM_131245 | Dr.75570 | *id1* | *ID1* | -1.249 | 6.41E-04 |
| NM_001045060 | Dr.105075 | *zgc:162356* | *N.A.* | -1.250 | 2.85E-05 |
| NM_001136254 | Dr.111383 | *zgc:195195* | *N.A.* | -1.251 | 1.82E-03 |
| NM_001005394 | Dr.162084 | *gypc* | *GYPC* | -1.262 | 1.61E-03 |
| NM_200091 | Dr.87745 | *stat1b* | *STAT1B* | -1.285 | 3.06E-04 |
| NM_001130662 | Dr.84738 | *trim105* | *TRIM105* | -1.289 | 3.45E-04 |
| NM_001171066 | Dr.151469 | *si:ch211-283h6.6* | *N.A.* | -1.300 | 2.06E-04 |
| NM_001002343 | Dr.79870 | *gbp1* | *GBP1* | -1.308 | 5.04E-06 |
| NM_001002493 | Dr.10032 | *jdp2* | *JDP2* | -1.309 | 3.83E-08 |
| NM_001098774 | Dr.111246 | *zgc:165583* | *N.A.* | -1.320 | 2.86E-04 |
| NM_001007430 | Dr.82534 | *zgc:101699* | *PLA2G4C* | -1.327 | 3.29E-05 |
| NM_212661 | Dr.78399 | *cd82a* | *CD82* | -1.329 | 1.45E-04 |
| NM_001128345 | Dr.84513 | *LOC566600* | *LOC566600* | -1.336 | 1.94E-05 |
| NM_001012386 | Dr.83988 | *gadd45bb* | *GADD45B* | -1.341 | 9.99E-07 |
| NM_001020481 | Dr.17901 | *gpr56* | *GPR56* | -1.345 | 1.21E-04 |
| NM_001004120 | Dr.2043 | *nfil3* | *ECHDC2* | -1.346 | 1.61E-07 |
| NM_200006 | Dr.81246 | *slc48a1b* | *SLC48A1* | -1.358 | 3.39E-08 |
| NM_001002561 | Dr.84618 | *clic2* | *CLIC2* | -1.359 | 4.53E-10 |
| NM_200112 | Dr.78704 | *padi2* | *PADI2* | -1.365 | 4.33E-04 |
| NM_001100441 | Dr.123450 | *zgc:165621* | *SYNGR3* | -1.377 | 3.93E-04 |
| NM_001139462 | Dr.13606 | *snx10a* | *SNX10A* | -1.388 | 3.73E-05 |
| NM_001098249 | Dr.74671 | *si:ch211-149p10.2* | *N.A.* | -1.403 | 3.48E-05 |
| NM_001122971 | Dr.88631 | *mxg* | *MXG* | -1.412 | 6.24E-05 |
| NM_001123052 | Dr.90318 | *erap2* | *ERAP2* | -1.413 | 1.64E-05 |
| NM_001045381 | Dr.81083 | *pter* | *PTER* | -1.421 | 3.56E-04 |
| NM_001030176 | Dr.74381 | *enpp5* | *ENPP5* | -1.424 | 3.93E-05 |
| NM_001252630 | Dr.94200 | *si:ch73-191k20.2* | *N.A.* | -1.431 | 1.16E-04 |
| NM_001110127 | Dr.80564 | *rbm38* | *RBM38* | -1.440 | 6.49E-05 |
| NM_001126460 | Dr.76334 | *si:dkey-169i5.4* | *N.A.* | -1.457 | 8.17E-06 |
| NM_200206 | Dr.81669 | *zgc:56194* | *CAST* | -1.468 | 3.08E-06 |
| NM_205569 | Dr.12986 | *fos* | *FOS* | -1.475 | 4.83E-11 |
| NM_001113590 | Dr.90160 | *tnfsf13b* | *TNFSF13B* | -1.494 | 6.02E-06 |
| NM_001083584 | Dr.126047 | *il-15ra* | *IL-15RA* | -1.499 | 1.01E-04 |
| NM_001128804 | Dr.81802 | *si:ch211-217k17.10* | *N.A.* | -1.547 | 6.22E-05 |
| NM_001075109 | Dr.90976 | *zgc:152774* | *MORC3* | -1.581 | 5.92E-09 |
| NM_001002623 | Dr.84875 | *zgc:92249* | *RARRES3* | -1.616 | 8.38E-07 |
| NM_001001849 | Dr.81752 | *trpv6* | *TRPV6* | -1.656 | 2.56E-05 |
| NM_001045118 | Dr.77857 | *scin* | *SCIN* | -1.672 | 1.60E-08 |
| NM_001020586 | Dr.88891 | *zgc:110354* | *MPEG1* | -1.706 | 1.88E-05 |
| NM_130920 | Dr.1400 | *rbp4* | *RBP4* | -1.719 | 1.22E-05 |
| NM_212894 | Dr.162115 | *lgals2a* | *LGALS1* | -1.719 | 3.07E-06 |
| NM_001001946 | Dr.159487 | *adh8a* | *ADH8A* | -1.730 | 1.23E-05 |
| NM_001258225 | Dr.135610 | *irig* | *IRIG* | -1.771 | 2.47E-07 |
| NM_001099430 | Dr.82089 | *aste1* | *ASTE1* | -1.777 | 7.08E-06 |
| NM_001083024 | Dr.93275 | *etv7* | *ETV6* | -1.780 | 8.65E-08 |
| NM_199515 | Dr.77773 | *prdm1a* | *PRDM1* | -1.795 | 5.32E-13 |
| NM_001130593 | Dr.160679 | *tlr1* | *TLR1* | -1.867 | 5.36E-08 |
| NM_001013555 | Dr.104643 | *zgc:113006* | *AOC3* | -1.869 | 3.96E-07 |
| NM_001099426 | Dr.71947 | *si:busm1-266f07.2* | *N.A.* | -1.945 | 5.61E-09 |
| NM_001007284 | Dr.151654 | *mxc* | *MX2* | -2.066 | 1.32E-07 |
| NM_001113588 | Dr.6471 | *dnajc18* | *DNAJC18* | -2.076 | 2.12E-08 |
| NM_001007428 | Dr.83801 | *cadm4* | *CADM4* | -2.156 | 2.05E-08 |
| NM_001042686 | Dr.134294 | *hspa12b* | *HSPA12B* | -2.250 | 1.27E-12 |
| NM_001020802 | Dr.80831 | *grn1* | *GRN1* | -2.313 | 3.30E-16 |

N.A. refers to not available.

**Table S5. List of the top 50 GSEA leading edge genes between the zebrafish TAN_L and the mouse TANs**

| **No.** | **Symbol** | **UGCluster** | **Name** |
| --- | --- | --- | --- |
| 1 | *Ch25h* | Mm.30824 | cholesterol 25-hydroxylase |
| 2 | ***Egr2*** | Mm.290421 | early growth response 2 |
| 3 | ***Lgals1*** | Mm.43831 | lectin, galactose binding, soluble 1 |
| 4 | *Egr1* | Mm.181959 | early growth response 1 |
| 5 | ***Pltp*** | Mm.6105 | phospholipid transfer protein |
| 6 | *Ctss* | Mm.3619 | cathepsin S |
| 7 | ***Lgals3bp*** | Mm.3152 | lectin, galactoside-binding, soluble, 3 binding protein |
| 8 | ***Dnase1l3*** | Mm.272258 | deoxyribonuclease 1-like 3 |
| 9 | ***Il1b*** | Mm.222830 | interleukin 1 beta |
| 10 | *Cryl1* | Mm.25539 | crystallin, lambda 1 |
| 11 | ***Cstb*** | Mm.6095 | cystatin B |
| 12 | *Ambp* | Mm.2197 | alpha 1 microglobulin/bikunin |
| 13 | *Rel* | Mm.4869 | reticuloendotheliosis oncogene |
| 14 | ***Atf3*** | Mm.2706 | activating transcription factor 3 |
| 15 | *Vegfa* | Mm.282184 | vascular endothelial growth factor A |
| 16 | *Nenf* | Mm.46444 | neuron derived neurotrophic factor |
| 17 | *Stab1* | Mm.220821 | stabilin 1 |
| 18 | *Il10* | Mm.874 | interleukin 10 |
| 19 | ***Apom*** | Mm.2161 | apolipoprotein M |
| 20 | *Hspa1a* | Mm.15514 | heat shock protein family A (Hsp70) member 1A |
| 21 | *Spata6* | Mm.275526 | spermatogenesis associated 6 |
| 22 | *Rtn4ip1* | Mm.390253 | reticulon 4 interacting protein 1 |
| 23 | ***Pgm2*** | Mm.18665 | Phosphoglucomutase 2 |
| 24 | *Top1mt* | Mm.182401 | DNA topoisomerase 1, mitochondrial |
| 25 | *Mrps18a* | Mm.287443 | mitochondrial ribosomal protein S18A |
| 26 | *Large* | Mm.324371 | like-glycosyltransferase |
| 27 | *Slc2a8* | Mm.305754 | solute carrier family 2, (facilitated glucose transporter), member 8 |
| 28 | *Cdk5rap3* | Mm.28297 | CDK5 regulatory subunit associated protein 3 |
| 29 | *Vps11* | Mm.295013 | vacuolar protein sorting 11 (yeast) |
| 30 | *Grn* | Mm.1568 | granulin |
| 31 | *Agxt2l2* | Mm.477511 | Alanine Glyoxylate Aminotransferase 2-Like 2 |
| 32 | *Panx1* | Mm.142253 | pannexin 1 |
| 33 | *Tnni2* | Mm.39469 | troponin I, skeletal, fast 2 |
| 34 | ***Birc3*** | Mm.2026 | baculoviral IAP repeat-containing 3 |
| 35 | *Preb* | Mm.272414 | prolactin regulatory element binding |
| 36 | *Il10ra* | Mm.379327 | interleukin 10 receptor, alpha |
| 37 | *Cdc42bpb* | Mm.93839 | CDC42 Binding Protein Kinase Beta |
| 38 | *Dhrsx* | Mm.305345 | dehydrogenase/reductase (SDR family) X chromosome |
| 39 | *Mrps9* | Mm.252982 | mitochondrial ribosomal protein S9 |
| 40 | *Pola2* | Mm.209931 | polymerase (DNA directed), alpha 2 |
| 41 | *Pdss1* | Mm.249752 | prenyl (solanesyl) diphosphate synthase, subunit 1 |
| 42 | *Slc39a1* | Mm.13536 | Solute Carrier Family 39 (Zinc Transporter), Member 1 |
| 43 | *Coro1b* | Mm.276859 | coronin, actin binding protein 1B |
| 44 | *Gsto1* | Mm.378931 | glutathione S-transferase omega 1 |
| 45 | *Atpaf2* | Mm.41651 | ATP synthase mitochondrial F1 complex assembly factor 2 |
| 46 | *Atox1* | Mm.217759 | ATX1 (antioxidant protein 1) homolog 1 (yeast) |
| 47 | *F9* | Mm.391283 | coagulation factor IX |
| 48 | *Tsta3* | Mm.22596 | tissue specific transplantation antigen P35B |
| 49 | *Cdkal1* | Mm.212227 | CDK5 regulatory subunit associated protein 1-like 1 |
| 50 | *Plaa* | Mm.490101 | phospholipase A2, activating protein |

The genes commonly enriched in at least two zebrafish TAN groups are highlighted in bold.

**Table S6. List of the top GSEA leading edge genes between the zebrafish TAN_M and the mouse TANs**

| **No.** | **Symbol** | **UGCluster** | **Name** |
| --- | --- | --- | --- |
| 1 | *Mmp14* | Mm.280175 | matrix metallopeptidase 14 (membrane-inserted) |
| 2 | *Socs3* | Mm.3468 | suppressor of cytokine signaling 3 |
| 3 | ***Pltp*** | Mm.6105 | phospholipid transfer protein |
| 4 | *Ptgs2* | Mm.292547 | prostaglandin-endoperoxide synthase 2 |
| 5 | ***Lgals3bp*** | Mm.3152 | lectin, galactoside-binding, soluble, 3 binding protein |
| 6 | ***Dnase1l3*** | Mm.272258 | deoxyribonuclease 1-like 3 |
| 7 | ***Ddit3*** | Mm.110220 | DNA-damage inducible transcript 3 |
| 8 | ***Il1b*** | Mm.222830 | interleukin 1 beta |
| 9 | ***Cstb*** | Mm.6095 | cystatin B |
| 10 | *Ccnd2* | Mm.333406 | cyclin D2 |
| 11 | ***Atf3*** | Mm.2706 | activating transcription factor 3 |
| 12 | *Lxn* | Mm.2632 | latexin |
| 13 | *Grasp* | Mm.276573 | GRP1 (general receptor for phosphoinositides 1)-associated scaffold protein |
| 14 | *Kifap3* | Mm.4651 | kinesin-associated protein 3 |
| 15 | ***Uap1l1*** | Mm.33797 | UDP-N-acteylglucosamine pyrophosphorylase 1-like 1 |
| 16 | *Arg1* | Mm.154144 | arginase, liver |
| 17 | *Eml1* | Mm.236645 | echinoderm microtubule associated protein like 1 |
| 18 | *Acp5* | Mm.46354 | acid phosphatase 5, tartrate resistant |
| 19 | *Atp6v1c1* | Mm.276618 | ATPase, H+ transporting, lysosomal V1 subunit C1 |
| 20 | *Plod3* | Mm.251003 | procollagen-lysine, 2-oxoglutarate 5-dioxygenase 3 |
| 21 | *H1fx* | Mm.33796 | H1 histone family, member X |
| 22 | *Extl3* | Mm.103748 | exostoses (multiple)-like 3 |
| 23 | *Hps1* | Mm.218381 | Hermansky-Pudlak syndrome 1 homolog (human) |
| 24 | *Bckdhb* | Mm.12819 | branched chain ketoacid dehydrogenase E1, beta polypeptide |
| 25 | *Selp* | Mm.3337 | selectin, platelet |
| 26 | *Mmp13* | Mm.5022 | matrix metallopeptidase 13 |
| 27 | *Ndel1* | Mm.31979 | nuclear distribution gene E-like homolog 1 (A. nidulans) |
| 28 | *Ccdc9* | Mm.482401 | coiled-coil domain containing 9 |
| 29 | *Hexa* | Mm.2284 | hexosaminidase A |
| 30 | *Snx1* | Mm.271891 | sorting nexin 1 |
| 31 | *Mphosph6* | Mm.181836 | M phase phosphoprotein 6 |

The genes commonly enriched in at least two zebrafish TAN groups are highlighted in bold.

**Table S7. List of the top 50 GSEA leading edge genes between the zebrafish TAN_F and the mouse TANs**

| **No.** | **Symbol** | **UGCluster** | **Name** |
| --- | --- | --- | --- |
| 1 | ***Egr2*** | Mm.290421 | early growth response 2 |
| 2 | ***Lgals1*** | Mm.43831 | lectin, galactose binding, soluble 1 |
| 3 | *Zfp36l1* | Mm.235132 | zinc finger protein 36, C3H type-like 1 |
| 4 | *Cish* | Mm.4592 | cytokine inducible SH2-containing protein |
| 5 | *Uck2* | Mm.280895 | uridine-cytidine kinase 2 |
| 6 | *Psmb10* | Mm.787 | proteasome (prosome, macropain) subunit, beta type 10 |
| 7 | *Adora2b* | Mm.40740 | adenosine A2b receptor |
| 8 | *Irf7* | Mm.3233 | interferon regulatory factor 7 |
| 9 | *Traf1* | Mm.239514 | TNF receptor-associated factor 1 |
| 10 | *Atp1a1* | Mm.193670 | ATPase, Na+/K+ transporting, alpha 1 polypeptide |
| 11 | ***Ddit3*** | Mm.110220 | DNA-damage inducible transcript 3 |
| 12 | *Tuft1* | Mm.10214 | tuftelin 1 |
| 13 | *Ppa1* | Mm.28897 | pyrophosphatase (inorganic) 1 |
| 14 | *Rasgrp1* | Mm.42150 | RAS guanyl releasing protein 1 |
| 15 | *Entpd4* | Mm.489765 | ectonucleoside triphosphate diphosphohydrolase 4 |
| 16 | *Ifrd1* | Mm.168 | interferon-related developmental regulator 1 |
| 17 | *Mif4gd* | Mm.390387 | MIF4G domain containing |
| 18 | *Gfer* | Mm.28124 | growth factor, erv1 (S. cerevisiae)-like (augmenter of liver regeneration) |
| 19 | ***Apom*** | Mm.2161 | apolipoprotein M |
| 20 | *Upp1* | Mm.4610 | uridine phosphorylase 1 |
| 21 | *Plekhf1* | Mm.333798 | pleckstrin homology domain containing, family F (with FYVE domain) member 1 |
| 22 | *Scarb2* | Mm.297964 | scavenger receptor class B, member 2 |
| 23 | *Clpp* | Mm.287892 | caseinolytic mitochondrial matrix peptidase proteolytic subunit |
| 24 | *Ell* | Mm.271973 | elongation factor RNA polymerase II |
| 25 | *Aldoc* | Mm.7729 | aldolase C, fructose-bisphosphate |
| 26 | ***Pgm2*** | Mm.18665 | Phosphoglucomutase 2 |
| 27 | *Dusp4* | Mm.170276 | dual specificity phosphatase 4 |
| 28 | *Pla2g7* | Mm.9277 | phospholipase A2, group VII (platelet-activating factor acetylhydrolase, plasma) |
| 29 | *Nr4a3* | Mm.247261 | nuclear receptor subfamily 4, group A, member 3 |
| 30 | *Ap1b1* | Mm.274816 | adaptor protein complex AP-1, beta 1 subunit |
| 31 | *Rrbp1* | Mm.13705 | ribosome binding protein 1 |
| 32 | *Atp13a1* | Mm.186066 | ATPase type 13A1 |
| 33 | *Tbc1d15* | Mm.22252 | TBC1 domain family, member 15 |
| 34 | ***Uap1l1*** | Mm.33797 | UDP-N-acteylglucosamine pyrophosphorylase 1-like 1 |
| 35 | *Agtpbp1* | Mm.153008 | ATP/GTP binding protein 1 |
| 36 | *Rps27l* | Mm.30120 | ribosomal protein S27-like |
| 37 | *Coq10b* | Mm.281019 | coenzyme Q10 homolog B (S. cerevisiae) |
| 38 | *Marcks* | Mm.30059 | myristoylated alanine rich protein kinase C substrate |
| 39 | *Heatr3* | Mm.36291 | HEAT repeat containing 3 |
| 40 | *Wsb1* | Mm.56416 | WD Repeat And SOCS Box Containing 1 |
| 41 | ***Birc3*** | Mm.2026 | baculoviral IAP repeat-containing 3 |
| 42 | *Slc20a1* | Mm.272675 | solute carrier family 20, member 1 |
| 43 | *Acat2* | Mm.11414 | acetyl-CoA acetyltransferase 2 |
| 44 | *Pcca* | Mm.18522 | propionyl-Coenzyme A carboxylase, alpha polypeptide |
| 45 | *Creb3* | Mm.12407 | cAMP responsive element binding protein 3 |
| 46 | *Ptbp1* | Mm.265610 | polypyrimidine tract binding protein 1 |
| 47 | *Cfl1* | Mm.329655 | cofilin 1, non-muscle |
| 48 | *Hk2* | Mm.255848 | hexokinase 2 |
| 49 | *Traf4* | Mm.390418 | TNF receptor associated factor 4 |
| 50 | *Arl5b* | Mm.174068 | ADP-ribosylation factor-like 5B |

The genes commonly enriched in at least two zebrafish TAN groups are highlighted in bold.
